# Anion-Exchange Membrane Water Electrolyzers

**DOI:** 10.1021/acs.chemrev.1c00854

**Published:** 2022-04-20

**Authors:** Naiying Du, Claudie Roy, Retha Peach, Matthew Turnbull, Simon Thiele, Christina Bock

**Affiliations:** † National Research Council of Canada, 1200 Montreal Road, Ottawa, Ontario K1A 0R6, Canada; ‡ Energy, Mining and Environment Research Centre, 1200 Montreal Road, Ottawa, Ontario K1A 0R6, Canada; § National Research Council of Canada, 2620 Speakman Drive, Mississauga, Ontario L5K 1B1, Canada; ∥ Forschungszentrum Jülich GmbH, Helmholtz Institute Erlangen-Nürnberg for Renewable Energy (IEK-11), Cauerstaße 1, 91058 Erlangen, Germany; ⊥ Department Chemie- und Bioingenieurwesen, Friedrich-Alexander-Universität Erlangen-Nürnberg, Egerlandstr. 3, 91058 Erlangen, Germany

## Abstract

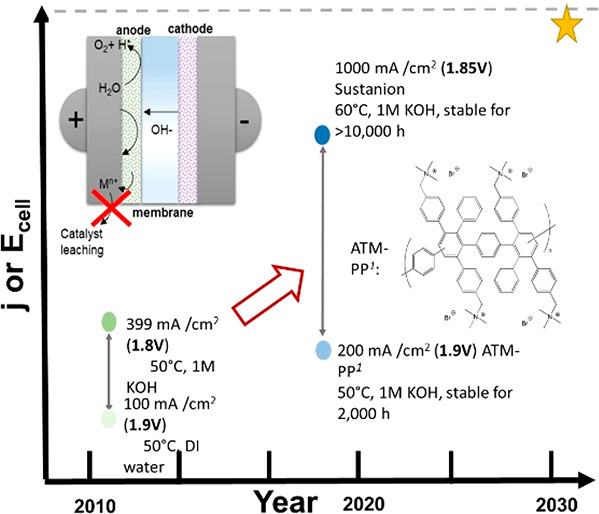

This Review provides an overview of the emerging concepts of catalysts, membranes, and membrane electrode assemblies (MEAs) for water electrolyzers with anion-exchange membranes (AEMs), also known as zero-gap alkaline water electrolyzers. Much of the recent progress is due to improvements in materials chemistry, MEA designs, and optimized operation conditions. Research on anion-exchange polymers (AEPs) has focused on the cationic head/backbone/side-chain structures and key properties such as ionic conductivity and alkaline stability. Several approaches, such as cross-linking, microphase, and organic/inorganic composites, have been proposed to improve the anion-exchange performance and the chemical and mechanical stability of AEMs. Numerous AEMs now exceed values of 0.1 S/cm (at 60–80 °C), although the stability specifically at temperatures exceeding 60 °C needs further enhancement. The oxygen evolution reaction (OER) is still a limiting factor. An analysis of thin-layer OER data suggests that NiFe-type catalysts have the highest activity. There is debate on the active-site mechanism of the NiFe catalysts, and their long-term stability needs to be understood. Addition of Co to NiFe increases the conductivity of these catalysts. The same analysis for the hydrogen evolution reaction (HER) shows carbon-supported Pt to be dominating, although PtNi alloys and clusters of Ni(OH)_2_ on Pt show competitive activities. Recent advances in forming and embedding well-dispersed Ru nanoparticles on functionalized high-surface-area carbon supports show promising HER activities. However, the stability of these catalysts under actual AEMWE operating conditions needs to be proven. The field is advancing rapidly but could benefit through the adaptation of new in situ techniques, standardized evaluation protocols for AEMWE conditions, and innovative catalyst-structure designs. Nevertheless, single AEM water electrolyzer cells have been operated for several thousand hours at temperatures and current densities as high as 60 °C and 1 A/cm^2^, respectively.

## 1 Introduction

**Figure 1:**
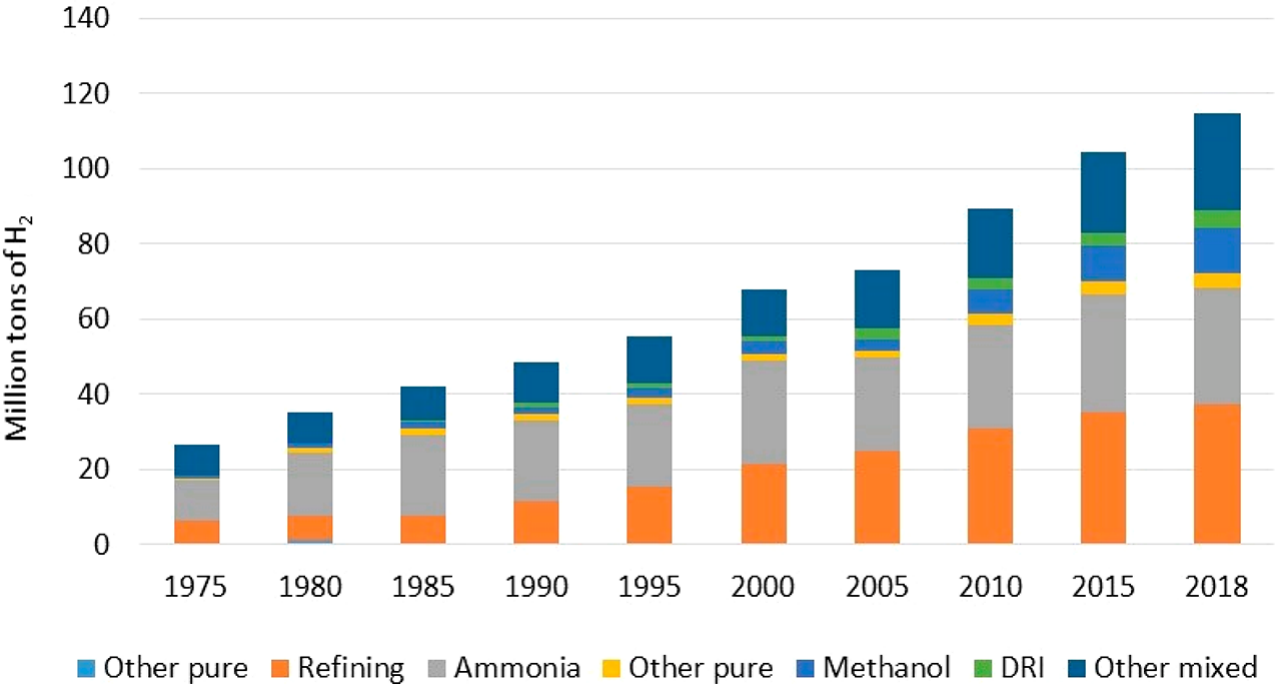
Historical trend of the global usage of H_2_ predominantly produced by utilizing a fossil fuel feedstock divided into industrial sectors. “Other pure” stands for applications needing high-purity H_2_, “DRI” stands for direct reduced iron steel production, and “Other mixed” stands for applications using H_2_ as a mixture gas, e.g., fuel or feedstock synthesis gas. Made from ref (1). Copyright 2019 U.S. Department of Energy.

Hydrogen has played a key role throughout the industrial life of humankind, and the global demand for H_2_ has continuously increased, in fact tripling since 1975.^1,2^ Today’s global H_2_ production exceeds 70 million metric tons (MMTs)/year and is consumed by the oil and gas industry and by metal refineries or turned into value-added products such as NH_3_, feedstock chemicals such as CH_3_OH, or specialty chemicals.^1^ H_2_ can be considered a commodity of increasing need, with its importance already being reflected in its 117 billion US$ global market value.^3^Figure 1 shows the trend for the global demand for H_2_ divided into end-use sectors. The actual H_2_ demand per sector depends on the country, e.g., in the United States, the oil and gas sector consumes 80% of the produced H_2_. The future demand for H_2_ may experience an additional increase due to H_2_’s physical properties such as its high gravimetric standard heat of formation (being the highest among fuels)^4^ and its standard heat of formation value (H_2_’s high heating value is 142 MJ/kg^5^), which is up to 3 times higher than that for liquid hydrocarbon fuels.^6^ Unfortunately, the volumetric density of H_2_ of 8 MJ/L is 4 times lower than the 32 MJ/L value of gasoline,^7^ thus requiring a high storage volume or significant gas compression.^2^

Since the late 1950s, steam methane reforming (SMR) has been predominantly used to produce H_2_ followed by coal gasification and water electrolysis (WE), although the latter only contributes 2–4% to today’s global H_2_ production.^8,9^ The heating value of CH_4_ is high (the HHV is 55.5 MJ/kg^5^), making the production of H_2_ via SMR, which is in the range of 2 €/kg H_2_,^9−11^ economically attractive. Today, 96% of H_2_ is produced from fossil fuel-based feeds (48% from natural gas, 30% from heavy oils and naphtha, and 18% from coal).^1,6^ H_2_ production from fossil-based sources and moreover from CH_4_, which is an ∼30 times more potent greenhouse gas than CO_2_,^12,13^ is a net emitter of CO_2_ and other air pollutants. On the basis of calculations and a large set of reported data, Sun et al.^14^ concluded average CO_2_/H_2_ values of 9 kg/kg and 75.4 kg/MJ, translating into 720 MMT/year of CO_2_ emitted for 70 MMT/year H_2_ produced from SMR. The latter suggests that H_2_ produced from SMR alone contributes 1.7% to the 43.1 billion metric tons worldwide emissions from humans in 2019.^15,16^ Furthermore, the SMR process also produces H_2_ of low purity (95–98%), requiring upgrading steps such as pressure swing absorption for many applications (e.g., fuels, specialty chemicals, and the ceramic and electronics industries).^17^ A mechanical compression step, which can be costly, also needs to be added.^18^ WEs have an advantage of generating higher-purity and already partially compressed H_2_.

H_2_ as a fuel for the H_2_ economy is still a topic of interest.^19,20^ Much of the interest is driven by our increasing demand for energy based on cleaner sources.^21^ Correspondingly, H_2_ is considered for energy storage (ES) and as a fuel.^11,21^ The driving forces for the H_2_ economy are different across the globe depending on the resources, potential for energy generation, and political landscape of a country.

To understand the feasibility for a specific H_2_ production or storage route, technical gaps need to be identified and examined while keeping the cost in mind. The complete cycle needs to be considered including the end use of H_2_, which can be manifold and may be different depending on the locations of H_2_ production and consumption. H_2_ is suitable for short-, medium-, and long-term energy storage. H_2_ could, e.g., be reelectrified and injected back into the grid considering payback options known as power to power, which is used as a grid service to balance the grid when demand is high and production is low, or that known as price arbitrage, or to avoid the building of new grid connections.^11,22−24^ In addition, H_2_ can also be transformed to a liquid organic hydrogen carrier (LOHC), enabling safer transport for later use.^25−29^

In this Review, the feasibility of H_2_ produced from electrochemical water splitting coupled with renewable energies is of interest. The anion-exchange membrane water electrolysis (AEMWE) is one of three types of low-temperature (<100 °C) WEs. The other two are proton-exchange membrane water electrolysis (PEMWE) and traditional electrolysis, which uses highly caustic KOH as the electrolyte and a porous separator. Among the three low-temperature WEs, AEMWE is the least-mature technology, and prior to implementation, significant technological hurdles need to be overcome. Many of the hurdles lie in the AEMWE components’ chemistries, which will be discussed in this Review. References are made where needed to scientific knowledge established for well-studied PEMWEs and fuel cells (FCs). This Review differs from recent publications^30−41^ as it presents a comprehensive analysis of all components of an AEMWE up to the single-cell level and reviews AEMWE single-cell performances for cells that have shown at least 100 h of operation. Performance results and needs of materials development and engineering are given based on high-level analyses.

## 2 To and from H_2_ Produced via Water Electrolysis: Sources, Cost, Conversion, and Principles

Biomass and water are renewable sources that potentially allow clean H_2_ production.^6,42^ Vast amounts of biomass are available, but the production of H_2_ from water is more advanced; hence, H_2_ derived from biomass is viewed to be implemented in the long term. H_2_ production from water splitting  can be divided into low- and high-temperature electrolysis, thermochemical water splitting, and photoelectrochemical processes. Due to the absence of carbon-based reaction fuels, water splitting offers the cleanest way of producing H_2_, provided clean sources of electricity are used.

Fossil fuel-free energy sources, such as nuclear and renewables, can be low or CO_2_-free forms of energy. Nuclear offers several advantages as vast amounts are available and excess energy during low demands can be stored via, e.g., electrolysis. The outlet temperatures of nuclear reactors are in the range of 300–950 °C. Such a range can be attractive for higher-temperature electrolyzers, e.g., solid oxide electrolysis cells and thermochemical water splitting. However, these high-temperature electrolysis methods are not yet mature and suffer material-corrosion issues above 100 °C.^6,43^

Solar and wind provide intermittent forms of energy. Solar is currently the fastest growing energy source due to the many investments made globally. Solar energy supplied just above 2% of the global electricity usage in 2018, while wind energy provided ∼5%.^44^ The global capacity and usage of wind energy may well grow, as it is not costly and the technology is continuously advancing, even though on-shore wind farms require thousands of acres of land.^44^ Electrochemical WE is best suited in combination with wind energy, which calls for storage in the MW range.^45^ WE not only is able to provide large-scale storage but also offers medium- and longer-term storage unlike, e.g., flywheels, which are low cost but only allow short-term storage.^46^ In addition, WEs can accept high-current inputs per surface area, operate in dynamic modes, and can be ramped up quickly, which are all requirements for storage of intermittent energy sources.^11,45,41^ Batteries are not suitable as a storage option for wind energy because they only accept low currents per surface area and have high self-discharge rates.^32,47,48^ Thermal molten salts are another high-energy storage density option being developed.^49^ However, it is based on exchanging heat. The heat is stored in a molten salt (which is thermally insulated) and released when needed. In addition, a WE is better suited than a battery for operations in cold climates because WEs can be heated using internal electrical currents without compromising their lifetime.^50,51^ Unfortunately, the intermittent nature of renewable energy lowers the annual operating hours, thus increasing the cost of the technology.^9^

The coupling of wind and solar energy with WEs provides many advantages; nevertheless, clear challenges exist. Table 1 provides a summary of the H_2_ production characteristics from SMR and WEs. It is seen that a challenge of H_2_ produced by WEs is the cost, which can be captured in the sum of the operating (OPEX) and capital (CAPEX) investment costs.

**Table 1:** H_2_ Production Characteristics of Steam Methane Reforming (SMR) versus Electrochemical (<100 °C) Water Electrolyzers (WEs)

characteristic	SMR	WE
feed	fossil fuel	H_2_O
estimated CO_2_ emissions per kg H_2_ (kg/kg H_2_)	9^a^	0.4–0.9^b^
global CO_2_ emissions (MMT/year) (2019)	720	32–72^b^
% of global CO_2_ emissions 2019 (est)	1.7	0.08–0.17^b^
H_2_ production cost (2019) (€/kg_H2_)	2	>3.8^c^
driving force for reaction	heat	electrical energy
catalysts	sulfur- and coke-tolerant (nickel, nanosized nickel, platinum, rhodium)	acidic: Pt (cathode), IrO_2_ (anode)
		alkaline: nickel-based or Pt (cathode), often nickel-based (anode)
H_2_ purity^d^ (%)	95–98	PEMWE: 99.9–99.999^e^
		AEMWE: 99.4

a Taken from ref (15).

b The data are for traditional alkaline water electrolyzers coupled with wind energy and estimated from refs (52−55). Fewer carbon-footprint studies are available for water electrolysis coupled with wind than from SMR processes. However, all of these studies consistently show water electrolyzers coupled with wind to be one of the lowest CO_2_ emitters.

c An estimate of 3.8 €/kg H_2_ is for coupling with wind, minimal operating hours of 7000 of 8760 per year, i.e., 80% capacity, a CAPEX of 800 €/kW, WE efficiency of 80%, and renewable electricity cost of 70 € M/Wh.^10^

d H_2_ purity without purification processes as pressure or temperature swing adsorption.

e Typically at 30 bar outlet pressure.

For low-temperature WEs the price of electricity is often taken as the OPEX value because electricity prices often dominate the cost of H_2_ production and reliable data for operating large-scale WEs are lacking.^9^ However, it is advisible to also include the cost of water (which needs to be of drinking water quality), specifically for operation at remote locations, as well as the WE maintenance costs.

The CAPEX cost is typically given as the investment cost per kW of electrical capacity and sometimes as the cost per nominal H_2_ production rate (in m^3^/h). In general, the definition of the CAPEX value in the form of cost per nominal capacity or cost per nominal H_2_ production rate is not complete.^56^ Neither of the two include relevant electrolyzer information such as the lifetime and the H_2_ production efficiency. A few approaches have been suggested to calculate the contribution of the CAPEX to the cost of H_2_ production in order to include the actual performance capabilities of different WEs.^56^ Villagra and Millet defined the CAPEX contribution to the total H_2_ cost (“CAPEX”) in €/kg of H_2_ produced as follows:^56^
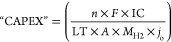
1In eq 1, *n* is the number of electrons (2 for H_2_ electrolysis), *F* is the Faraday constant (96 485 C/mol_e–_), IC is the initial WE cost, LT and *A* are the lifetime and geometrical electrode area of the WE, respectively, *M*_H2_ is the molecular H_2_ weight, and *j*_o_ is the operational current density. This definition gives a clearer indication of the WE characteristics that influence the H_2_ production cost as compared to the traditionally used CAPEX values. However, eq 1 does not include the efficiency of the WE, which could simply be introduced as a term in the dividend in eq 1.

From eq 1 it is seen that the CAPEX contribution to the cost of H_2_ produced decreases with an increase in the WE lifetime, electrode surface area, operating current density (*j*), and average efficiency of the H_2_ produced.^56^ Logically, a lower initial cost of the WE, which is influenced by materials and manufacturing costs, also reduces the cost of H_2_ production. To obtain the full cost of H_2_ production, the OPEX and CAPEX are combined. The joint OPEX and CAPEX costs define the technical targets the technology needs to achieve to be competitive for deployment. According to Table 1, WEs must produce H_2_ at a cost below 2 €/kg_H2_ to be cost competitive. The price for H_2_ production by WE coupled with wind energy could be below 3.8 €/kg_H2_ if the WE is used at an 80% annual capacity, has a CAPEX value of 800 €/kW, and has a cell efficiency of 80% at OPEX costs corresponding to renewable electricity costs of 70 €/MWh.^9^ Proost and others suggest that WEs could become more competitive to SMR as CAPEX prices of WEs are predicted to decrease with an increase in manufacturing (taking advantage of the economy of scale) and to a lesser extent also continue to decrease through additional research and development contributions.^9,52,57^ This seems reasonable considering that the CAPEX prices of PEMWEs decreased by 1 order of magnitude between 2000 and 2010 and continue to steadily decrease, as demonstrated in Table 2.^18−59^ These trends suggest that H_2_ production by WEs will become competitive if low-cost electricity is used.^9,60^

**Table 2:** Evolution of CAPEX Values for PEMWEs

year	IC^a^ per H_2_ output (M€/*t*_H2_^b^ day)	CAPEX^b^ (€/kW)
2014	8	4000
2018	3	1500
target for 2023	1.5	750

a Initial cost (IC).

b Source: refs (56 and 57).

### 2.1 H_2_ Conversion to Chemical Raw Materials or LOHCs

**Figure 2:**
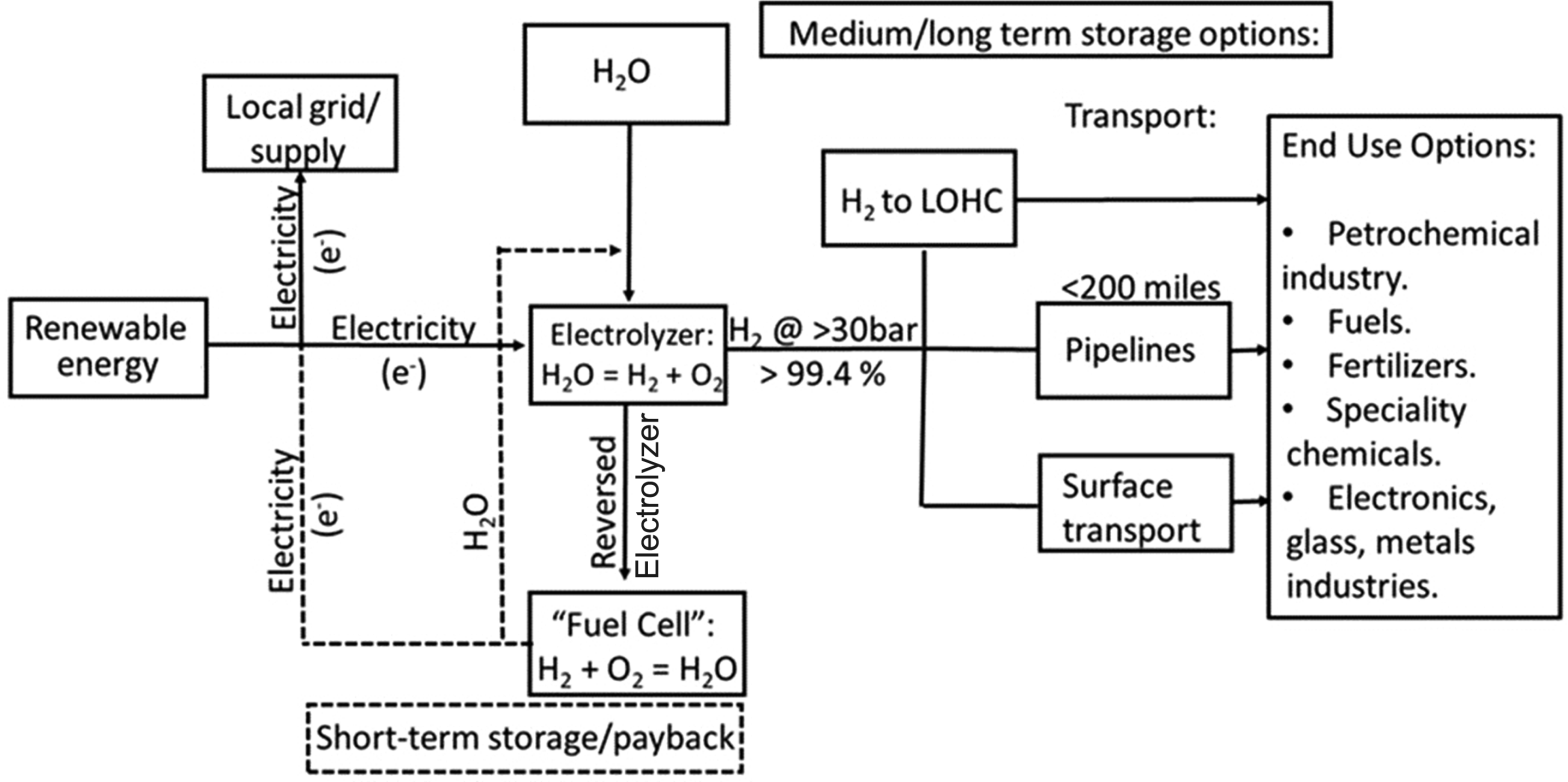
Schematic for the coupling of renewable (wind or solar) energy with water electrolysis. The figure shows options for short-, medium-, and long-term storage for the energy in the form of H_2_ and possible end uses including payback options. LOHC stands for liquid organic hydrogen carrier.

A scheme demonstrating the coupling of wind with WE and the possible uses of the stored H_2_ is shown in Figure 2. Much of the wind resources are located in remote areas, and transport of H_2_ in pipelines is only feasible over limited distances in the 100–200-mile range.

Several studies^61,62^ proposed to transform the H_2_ into a chemical raw material or LOHC (liquid organic hydrogen carrier), which are H_2_ carriers that can allow for safer transportation. The chemical raw materials could be CH_3_OH, dimethyl ether, gasoline, ammonia, and Fischer–Tropsch fuels. Examples of LOHC systems are *N*-alkylcarbazoles and derivatives.^63^ An early LOHC system was toluene/cyclohexane, but dehydrogenation in the liquid phase with easy condensation of the evaporated parts of the H_2_ carrier is also possible for higher-boiling aromatics and heteroaromatics.^27,64,65^ The LOHC is formed by a catalytic hydrogenation and a reversible dehydrogenation reaction. LOHC systems are liquids and can be used in the existing fuel infrastructure. LOHCs are also reloadable without the release of CO_2_. LOHCs offer higher volumetric energy densities than H_2_ and can be a room-temperature, long-term storage option. They could serve as a H_2_ supplier for arbitrary applications such as energy or specialty chemicals. CH_3_OH could be formed from CO_2_ concentrated from the atmosphere and H_2_ from electrolysis. CH_3_OH has a high acceptance level due to its similarity to existing fuels, although a 2014 techno-economic study showed that the cost of CH_3_OH via the route of using clean H_2_ is over the market price.^66^ Similarly, NH_3_ can be formed by electrolysis,^67^ which according to recent studies releases less CO_2_, when coupled with wind or solar energy, than the traditional Haber–Bosch process.^68^

### 2.2 Electrochemical Water Electrolyzers

**Figure 3:**
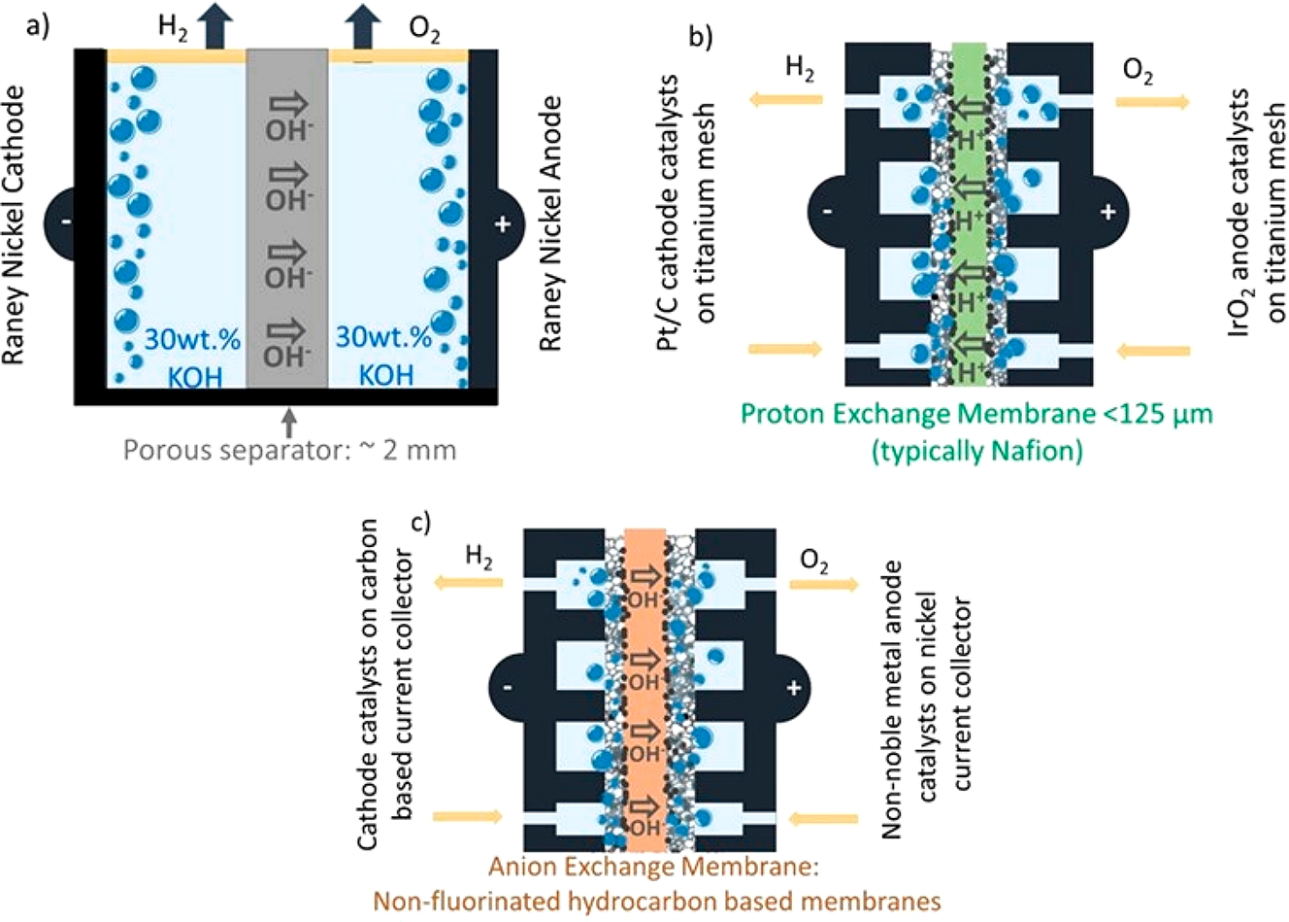
Schematic of the three types of WEs as (a) traditional alkaline finite WE (AWE), (b) zero-gap PEMWE running under acidic conditions using an H^+^ conducting membrane, and (c) zero-gap AEMWE utilizing an OH^–^ conducting membrane. The goal is to use noble metal-free catalysts for the cathode and anode of an AEMWE.

Low-temperature water electrolyzers (WEs) can be divided into alkaline and acidic systems. They are further divided into finite and zero-gap electrolyzers (Figure 3). The schematics show the principles of a single WE cell, while an actual system consists of an assembly of many cells known as a stack. The anode and the cathode in a WE are spaced using a separator to avoid mixing of the H_2_ and O_2_ gases.

The terms finite and zero gap are related to the distance of the separator between the anode and the cathode, where the O_2_ evolution reaction (OER) and the H_2_ evolution reaction (HER) take place. Finite-gap alkaline WEs employ a porous separator and aqueous, e.g., 30 wt % (5 M) KOH, conducting solutions (Figure 3a).^50,69^ This is a proven technology and has been deployed in MW scales since the late 1950s.^50,70^ A well-known advantage of alkaline conditions, specifically pH > 13, is the stability of the non-platinum group metal (non-PGM)-based catalysts for the OER and HER, unlike for acidic media needing platinum group metal catalysts.^42,44,50^ Typically, high-surface-area Raney nickel electrodes are used in an infinite-gap alkaline electrolyzer.^71,72^ The use of a porous separator, such as Zircon and Perl UTP 500,^73−75^ calls for a large distance (>2 mm) between the anode and cathode to reduce H_2_ and O_2_ gas crossover, which unfortunately is accompanied by a high ohmic resistance due to the direct dependency of ionic resistance on electrolyte thickness. The latter limits the maximum current densities (*j*_max_) that can be reached.^46,56,76^ Typically the *j*_max_ value for a finite-gap alkaline WE is 0.25 A/cm^2^, which is too low for integration with renewables, such as wind, that need ES technologies that are able to accept current densities in the several A/cm^2^ range as well as with fast dynamic responses.^24,44^ New WE designs are being developed that incorporate one electrode of minimized or even zero-gap distance to the separator.^42,76−78^ Examples explored are alkali-doped ion-solvating membranes in combination with, e.g., 24 wt % KOH electrolytes.^79−81^ Single-cell tests using a KOH-doped ion-solvating membrane and Raney nickel electrodes yielded a low cell voltage of 1.8 V at *j* values of 1.7 A/cm^2^.^80^

The zero-gap WE design reduces the internal resistance as thin polymer-based membranes of low H_2_ and O_2_ crossover are employed. Proton-exchange membranes (PEMs, also referred to as cation-exchange membranes) and anion-exchange membranes (AEMs) are used for acidic (Figure 3b) and alkaline (Figure 3c) zero-gap WEs, respectively. Consequently, zero-gap WEs are predicted to achieve higher *j* values than finite-gap electrolyzers. In the case of commercial PEMWEs, *j* values of up to 1–3 A/cm^2^ at lifetimes (LTs) of 15 000–20 000 h using membranes as thin as 50–200 μm PEMs have been demonstrated. PEMWEs are much more mature than AEMWEs. This is related to the fact that PEMs, which typically consist of a perfluorosulfonic acid that is known under the trademarks Nafion and Aquivion, have a significantly higher stability than anion-exchange membranes (AEMs), although the stability of Nafion is limited to 80 °C operations. In fact, PEMWEs using a Nafion separator are typically operated at 60 °C.^18^ Only in recent years have achievements been made to increase the stability of AEMs and single-cell AEMWEs run in the several A/cm^2^ range, although proof of extended durability and performance is still needed.^73,77,82−84^

**Figure 4:**
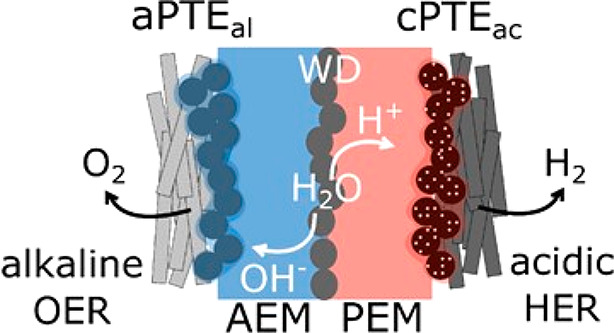
Schematic of a bipolar membrane (BPM) WE employing a solid AEM (blue) and PEM (red) with a water-dissociation (WD) catalyst layer located at the AEM|PEM interface. The OER and HER take place at the anode, indicated as aPTE_al_, and the cathode, indicated as cPTE_ac_, respectively. Reprinted with permission from Open Access article.^89^ Copyright 2021 Royal Society of Chemistry under CC Attribution 3.0 Unported License https://creativecommons.org/licenses/by/3.0/.

Recent developments in the field of bipolar membranes (BPMs) have opened new opportunities.^85^ The BPM’s principal lies in linking the advantages of the PEM and AEM system where low-cost anode materials (alkaline media) and active and durable cathode catalysts (acidic media) are used. In the BPM system, a cation-exchange membrane (CEM) and an AEM are in direct contact to form a bipolar interface (Figure 4).^85^ A water dissociation or water recombination catalyst is added between the two membranes to enhance the performance.^86−88^ Activities of such bilayer catalysts have been shown to be close to those of alkaline HER catalysts.^86^

Another difference between finite and zero-gap alkaline WEs is that zero-gap WEs operate on a pure water feed or dilute alkali electrolytes.^90^ The use of pure water theoretically eliminates issues related to the reaction of cations such as K^+^ with CO_2_ to form carbonates in OH^–^ environments but requires an OH^–^ conductive polymer, an anion-exchange ionomer (AEI), to be present in the catalyst layer.^46,91,92^ However, even at low KOH concentrations, or in pure water, the complete exclusion of CO_2_ is a challenge because CO_2_ is present in the air and can easily dissolve in water (0.75 g/L at 50 °C). Much of the research and development on zero-gap systems has focused on PEMWEs because the implementation of AEMWEs still strongly depends on the availability of AEMs, which show long-term stability at elevated temperatures, although low-power (e.g., 0.5–1 N·m_H2_^3^/h) AEMWE systems are commercially available.^39,93,94^ The commercial system from Enapter (formerly Acta) offers high-purity (99.9%) H_2_ and 99.999% H_2_ with an optional dryer.^94^ An advantage of membranes, i.e., the zero-gap WEs, is to obtain a higher-purity H_2_ directly from the cell (section 6). WEs should last >50 000 h under high *j* values and ideally also under pressure of 50–80 bar and higher (≥60 °C) temperatures. Today’s commercial PEMWEs have shown long (at least 20 000 h) lifetimes at low temperatures and 30 bar.^39,43^

### 2.3 Thermodynamics for WEs

The water splitting reactions in acidic and alkaline media are overall comparable, although in alkaline media OH^–^ is the conducting ion, while in acidic media H^+^ assumes this role. In the case of alkaline WEs, the reactions are as follows,
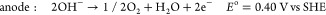
2
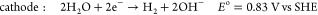
3
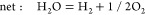
4where SHE stands for standard hydrogen electrode and *E*° is the reversible potential. In alkaline conditions, the cathode needs two water molecules per H_2_ produced, and thus the water transport from the anode to the cathode is a crucial factor to be considered in the cell design, materials selection, and operation mode of the WE. The OH^–^ needed at the anode is provided through the cathode reaction and needs to be transported through the catalyst layer and membrane to the catalyst sites in the anode layer.

The standard reversible potential (*E*°_rev_) for the water splitting reaction is −1.23 V, i.e., the reaction is endothermic and does not occur at a cell voltage (*E*_cell_) below 1.23 V. For the water splitting reaction, the standard enthalpy and entropy are 285.84 and 163.6 kJ/mol_H2_, respectively.^43^ This difference indicates a large entropy change of the reaction system when liquid H_2_O changes into the two gaseous products H_2_ and O_2_. Electrolysis at higher *T* values (>100 °C), or more precisely using a H_2_O steam reactant, reduces the energy requirements of the electrolysis as this entropy change is eliminated. Reference is also made to the thermoneutral voltage (*E*°_tn_), which for the water splitting reaction is 1.48 V, reflecting the transition point between endothermic and exothermic, i.e., the potential at which the reaction proceeds without heat input.

**Figure 5:**
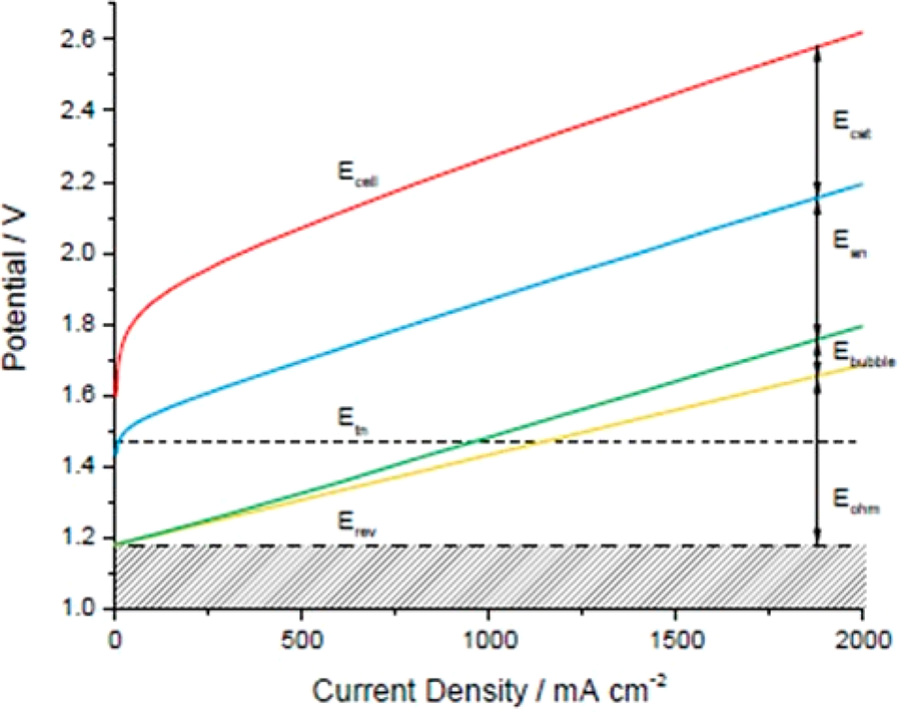
Typical potential (*E*_cell_)–current density (*j*) curves using arbitrary values demonstrating the cumulative contributions of different voltage losses. The anode and cathode voltages (*E*_an_ and *E*_cat_) can be reduced by using catalysts of higher activity and improved catalyst layers, while the ohmic voltage (*E*_ohm_) loss depends on both electrode conductivity and membrane ionic conductivity. Both,= potential losses due to gas bubble formation (*E*_bubble_) and ohmic losses (*E*_ohm_) are reflected in the *iR*_cell_ term shown in the simplified eq 5. Many factors influence the actual *E* losses thar are demonstrated in the figure. Reprinted with permission from (98). Copyright 2017 DTU Energy, Department of Energy and Energy Storage.

To understand the WE cell performance, the difference between *E*_anode_ and *E*_cathode_, i.e., the cell potential (*E*_cell_), is plotted versus *j* (Figure 5). *E*_cell_ depends on the reversible potential (*E*_rev_). However, an operating WE also experiences voltage losses as overpotentials (η) at the anode (η_an_) and cathode (η_cat_) and *iR* drops induced by the cell resistance (*R*_cell_):
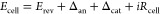
5In eq 5, *R*_cell_ is a lump resistance term made of a number of resistances including contributions from the membrane, polar plates, interfaces, system circuits, and mass-transport losses. Mass-transport losses are losses that result from the nonstoichiometric supply of reactants to the active catalyst centers.^95^ The formation of oxygen and hydrogen bubbles is one possible effect. Product gas bubbles in contact with the electrodes reduce the electrode contact with the liquid water, which in turn decreases the active electrode areas. All of the voltage loss terms in eq 5 increase with an increase in the current (*i*), i.e., *j* as shown in Figure 5. For well-designed low-temperature WEs, *iR* drops across the catalyst layers and other components such as the gas diffusion layer (GDL) and bipolar plates (BPs) are negligible.^96^ For today’s AEMWEs, the membrane resistance dominates the voltage.^97^ This of course can change with the continued development of AEMs.

**Figure 6:**
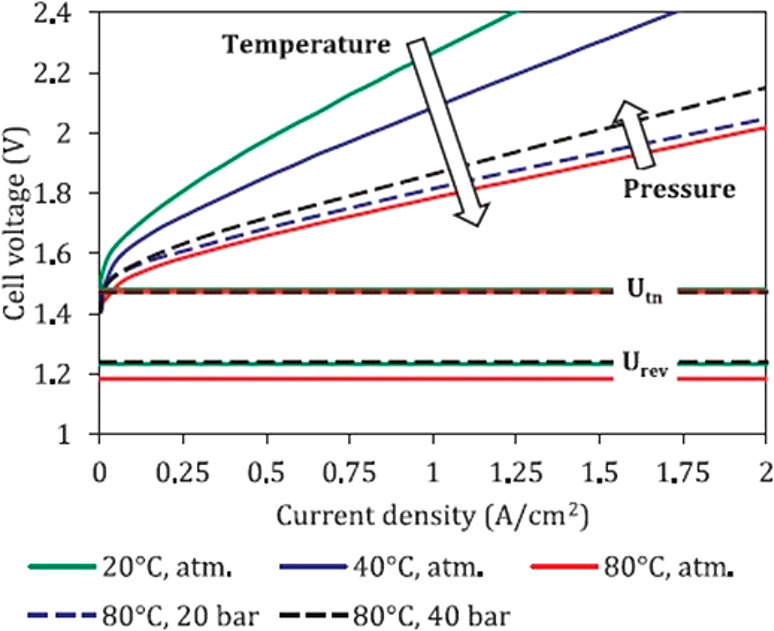
Cell voltage (*E*_cell_)–curve as a function of the applied current density for a PEMWE. The influences of *T* and *P* are shown. The thermoneutral voltage of 1.48 V, labeled as *U*_tn_, and the reversible voltage of 1.23 V, labeled as *U*_rev_, are also shown. Reprinted with permission from ref (24). Copyright 2018 Elsevier.

As seen from Figure 6, a higher temperature (*T*) lowers *E*_cell_, which is beneficial for the electrochemical reactions, increases the counterion transport, and facilitates H_2_ and O_2_ separation as the gas solubility decreases with increasing *T*.^99−101^ A higher pressure (*P*) increases *E*_cell_ according to the Nernst equation, although the increase is not pronounced. In fact, the availability of compressed H_2_ directly from a WE is a benefit, reducing the cost of mechanical H_2_ compression provided that the compression remains in the 30–50 bar range.^24,35,102^ WE operation at higher pressures can require the reinforcement of thinner membranes (e.g., in the case of PEMWEs < 125 μm)^103^ to increase their mechanical strength and achieve higher WE efficiencies.

### 2.4 Key Target Performance Characteristics

To make H_2_ production via PEMWEs economically competitive, the CAPEX cost needs to drop below 750 €/kW. This is at an electricity (OPEX) cost of <70 €/MWh.^9^ On the basis of extrapolations of experimental voltage versus current curves for single-cell PEMWEs, this goal has been suggested as feasible for WEs operated at ≥80 °C and current densities of 10 A/cm^2^, using a thin (25 μm) Nafion 212 membrane, and achieving a WE lifetime exceeding both 15 000 h and efficiencies of 75%.^56^ These data can be used as a guideline to approximate the target characteristics and potential cost savings when changing to more-abundant materials for the milder AEMWE conditions. This assumes that the economy of scaling to a fully integrated AEMWE system follows at least the same beneficial trend as observed for PEMWEs and relies on the development of AEMWE component materials matching lifetime and performance needs.^18^

**Figure 7:**
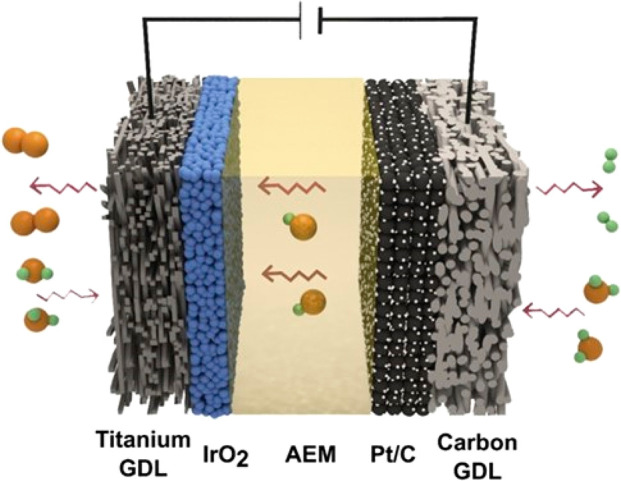
Schematic diagram of components for a single cell of an AEMWE. In this schematic, IrO_2_-based catalysts and a porous titanium transport layer (PTL) are used at the anode. (The titanium PTL is referred to as GDL in the schematic.) At the cathode (right-hand side), carbon-supported Pt (Pt/C) catalysts and a porous carbon GDL are used. H_2_O, OH^–^, and gas (H_2_ and O_2_) molecule flow are also indicated in the figure. Depending on the AEMWE, other catalyst compositions, e.g., Ni- and Fe-based anode catalysts, are often used. Reprinted with permission from ref (107). Copyright 2019 Elsevier.

Cost data for PEMWEs suggest that the stack makes up 60% of the CAPEX amount.^11^ The stack is made of the individual WE cell and the appropriate separators. The cell contains the heart of the WE: the membrane electrode assembly (MEA). The MEA is made of the anode and cathode catalyst layers, which are interfaced with the porous transport layers (PTLs) and sandwich the AEM. Typically in water electrolysis, metal-based PTLs are used at the anode; in alkaline conditions, a variety of Ni-based materials are used in single-cell tests. Because, in practice, the cathodic environment is less corrosive, more cost-efficient PTLs are in use at the cathode. These are mostly carbon-based (e.g., carbon fibers, carbon paper, or carbon cloth) and therefore often referred to as gas diffusion layers (GDLs); see, e.g., Figure 7.

The MEA, in turn, is sandwiched between two flow fields known as bipolar plates (BPs) that allow water, H_2_, and O_2_ to flow and conduct the current (Figure 7). For oxidative and acidic conditions, the BPs are typically made of costly titanium^104−106^ and dominate the cost, making up 51% of the stack costs, followed by the manufacturing costs of the MEA (10%) and the cost of the cathode (9%) and anode (8%) current collectors. The cost of the PEMWE anode catalyst and membrane are comparable at 6% and 5%, respectively, while research and development efforts resulted in cost reductions of the cathode to 1%. Changes in the BPs, e.g., by switching to stainless steel (even when noble metal coated for high potential corrosion protection),^103^ offer the potential for large reduction costs for AEMWEs compared to PEMWEs. In the case of other components such as the catalysts, the cost reduction by employing less-expensive materials alone is in the few to several % range. This indicates the need for AEM and catalyst improvements and calls for innovative material and component designs to assist in making AEMWEs viable.

## 3 HER and OER Catalysts

Enhancing the activity and stability of both the HER and OER electrocatalysts is crucial to make AEMWEs viable for large-scale deployment. OER catalyst improvements are urgently needed as the OER is a sluggish reaction, resulting in high overpotentials (ηs). A number of studies focused on gaining a detailed understanding of the HER and OER mechanisms in order to eventually create more-active catalysts. Reaction mechanistic understandings are important, but the creation of catalysts displaying high activities and stabilities in MEAs is crucial. Studies that focus on the AEMWE elecrocatalyst development often involve the preparation of catalyst powders. The catalyst powders can be subsequently transformed into catalyst layers (CLs) that can be up to several tenths of a micrometer thick. The activity of a catalyst measured in its powder form, i.e., prior to integration into a CL, and the activity of a catalyst in an actual CL of an MEA can be different (section 6). In some studies, thin catalyst films are also deposited onto solid and smooth electrode substrates such as gold foils. The latter can be valuable model catalysts, but for practical applications, porous current collectors enabling facile flow for the reactants and reaction products are needed. Therefore, the preparation of HER and OER electrocatalysts for AEMWEs often focuses on powder catalysts. However, the deposition of catalysts directly onto the porous and high-surface-area current collectors to be used in an MEA is also receiving attention, and recent literature has shown that such designs could open up AEMWE operation into high-current-density (>5 A/cm^2^) regimes.^108^

### 3.1 Metrics for Electrocatalysts

#### 3.1.1 Mass and Intrinsic Activity

Both the HER and OER are heterogeneous reactions; thus, electron transfer from and to the reactants occurs across the electrode surface. Modifications of the electrocatalyst generally have the goal to lower the energy barrier of the reaction, which in electrocatalysis is observed as a lower overpotential (η) and an overall increase in the electrochemical activity. The two main approaches used to increase an electrocatalyst’s activity are (1) increasing the number of active surface sites and (2) increasing the catalyst’s intrinsic activity.^109^ An obvious strategy lies in increasing the electrochemical surface area (ECSA) of the electrocatalysts. Many approaches involve the preparation of catalysts of nanometer dimensions to reach maximal increases in the ratio of surface to bulk atoms. However, studies in actual AEMWE cells are needed to confirm if catalysts of nanosized dimensions (specifically if they are unsupported) retain their high-surface-area benefit. In an MEA, the electrocatalysts need to form an electronically conductive network without hindering the flow of reactants and products.

The exchange current density (*j*_o_) and the current measured at a specific η are indicators of the activity of a catalyst and are often presented as mass activity (current per catalyst mass) or intrinsic activity (current per ECSA). The mass activity is of practical relevance, but as already mentioned, the intrinsic activity is a measure of the actual catalytic activity. Unfortunately, an accurate measurement of the ECSA value of many electrocatalysts other than platinum, specifically when of high surface area, can be challenging,^110,111^ and it is further discussed in the activity testing procedure section presented in the Supporting Information. Therefore, grouping catalysts according to their intrinsic activities can be difficult. In addition, there are inconsistencies in the measurement of catalyst activities. Data are extracted for different electrolytes and are often reported as η at a specific current density per electrode area (cm_geom_^2^). Such values are extremely difficult to compare because the loading of the catalysts on the electrode can be different and, of course, different catalysts can have widely different ESCA values. Another metric used in some studies is the turnover frequency (TOF), which is a function of the amount of H_2_ or O_2_ gas produced at a specific η resembling an equation as TOF in s^–1^: (the amount of gas produced at a specific η)/(*F* × *n*_e_ × *n*), where *F* is the Faraday constant, *n*_e_ is the number of electrons involved, and *n* is the number of catalyst atoms. However, there are a great deal of inconsistencies in calculating the TOF number specifically in the estimation of the amount of gas produced and the use of the number of catalyst atoms (*n*). For example, some authors use the total number of metal atoms of a catalyst, while others use the number of atoms on the catalyst surface; in some cases, the measured HER or OER current density is used as the amount of gas produced, while others measure the amount of gas produced. Therefore, the TOF values reported in the literature do not allow for an easy comparison of catalyst performances between different studies. If measured consistently, the TOF number could be a useful engineering metric. However, the consistent reporting of simply the current density (per mass and if possible per ECSA of the catalyst) at a specific η value (and preferably for the same electrolyte) rather than the TOF seems preferable for catalyst materials’ research purposes and presents fewer complications. Such an approach is consistent with a recent study by Anderson et al., who used a measurement protocol for OER catalysts, which reported current densities measured at a specific η value.^112^

**Figure 8:**
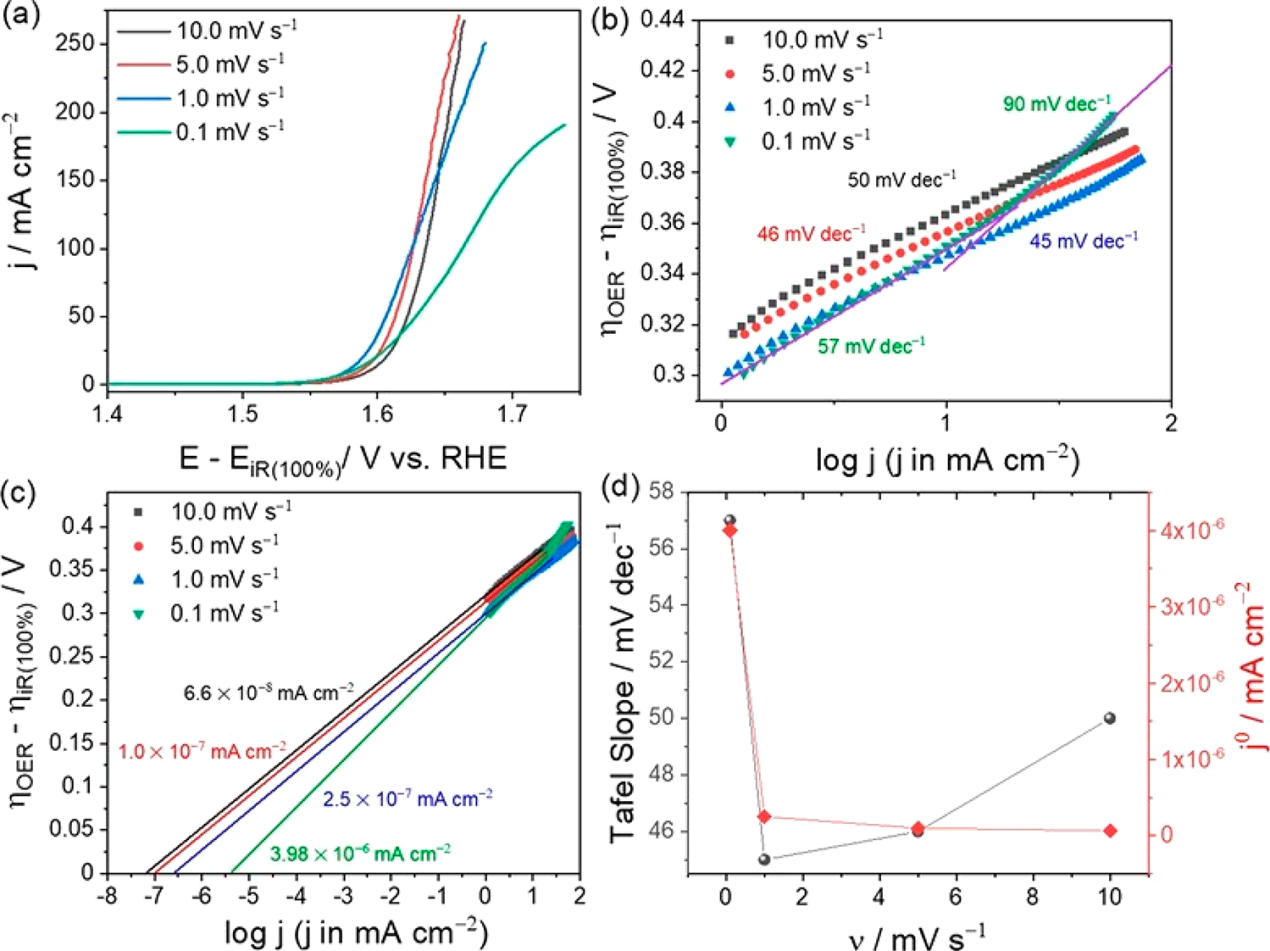
Demonstration of the erroneous impact of attempted Tafel slope measurements using slow-sweep voltage polarization, i.e., a nonsteady-state method. The data are for a Co foil measured using a 0.1 M KOH electrolyte. Reprinted with permission from ref (115). Copyright 2021 American Chemical Society.

The Tafel equation reflects kinetic information and yields the Tafel slope value (*b*) as follows: η = *a* + *b* × log(*j*). The Tafel slope yields reaction mechanistic information. To be valid, the Tafel slope needs to be determined at a η value exceeding *RT*/*F*, i.e., typically higher than at least 45–50 mV in order to neglect contributions from the back reaction.^113^ Smaller Tafel slopes are favorable as an increase in *j*, i.e., an increase in the HER and OER rates is accompanied by a smaller increase in both η and *E*_cell_ (eq 5). The η value is specific to a catalyst, indicating how the catalyst surface binds, interacts, and releases various reaction intermediates. The reaction kinetics are dependent on many experimental factors including the nature and morphology of the catalyst and the final electrode. Catalytic activities are influenced by the bulk and surface properties of a catalyst. It is well-known that catalyst activities can be tuned by means of alloying and introducing shape and ligand effects.^114^ Extrapolation of a Tafel plot to a η of 0, i.e., to the potential equaling the standard potential, yields *j*_o_. Tafel slope values need to be obtained from steady-state measurements (such as a constant-current or constant-potential experiment) because a Tafel slope depends on the surface coverage of adsorbed intermediate species. Many recent studies extracted mechanistic and Tafel slope information from slow-sweep linear voltammetry. Slow-sweep linear voltammetry does not provide steady-state conditions and hence can yield incorrect values. This has recently been emphasized by Anantharaj et al. and is demonstrated in Figure 8, which shows results for *iR*-corrected [ΔmV/Δdec] slopes extracted at different sweep rates for the example of a Co foil measured in 0.1 M KOH.^115^ It was demonstrated that the [ΔmV/Δdec] slopes depended on the sweep rate varying between 45 and 90 mV/dec, while the actual Tafel slope for this system extracted from constant-potential experiments yielded a value of 60 mV/dec.

Furthermore, the highest Tafel slope value measurable is 120 mV/dec (at 20 °C). Slopes exceeding 120 mV/dec are not Tafel slopes, i.e., their values cannot be interpreted as electrochemical reactions following Butler–Volmer behavior. Slopes higher than 120 mV/dec are observed and are a result of factors such as changes in the catalyst/electrode structure, which could be the formation of a resistive surface oxide and/or other changes in the catalyst structure.^113^

#### 3.1.2 Metrics Including the Catalyst Stability

The development of catalysts often focuses on developing a material exhibiting a high electrocatalytic activity. However, the activity of a catalyst does not always correlate with the lifetime of a catalyst. Therefore, other metrics to assess catalysts can be useful, such as the recently suggested *S*-number.^116^ The *S*-number is the ratio between the amounts of evolved H_2_ or O_2_ gas versus the amount of dissolved catalyst metal.^116,117^ The amount of gas evolved is normalized using the ECSA value. The *S*-number appears to be a good indicator providing a comparative and balanced measure of catalytic activities and stability. However, care needs to be taken with the measurement of the *S*-number because the ECSA of a catalyst can change during the course of the measurement. Other similar metrics that could be useful reflect the catalyst utilization and lifetime in CL layers and MEAs for operating AEMWE conditions.

### 3.2 HER Catalysts

The kinetic pathway of the HER generally follows the Volmer–Heyrovskey or Volmer–Tafel mechanism.^118^ Both consist of water adsorption, followed by water dissociation (Volmer step, eq 6), and then either hydrogen dissociation via chemical desorption (Tafel step, eq 7) or electrochemical desorption (Heyrovsky step, eq 8) to form H_2_:^118^
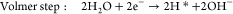
6
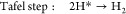
7
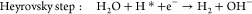
8In eqs 6–8, the * indicates a surface-bound species. Tafel slopes of −30, −40, or −120 mV/dec measured at 20 °C may be observed if the Heyrovsky, Tafel, or Volmer reaction, respectively, is the rate-determining step (rds).^118−120^ However, it is impossible to distinguish the actual reaction routes for the HER in the case of a −120 mV/dec Tafel slope.^119^ The energy barriers associated with each step play a role in determining the catalytic activity. It was suggested that the HER current density can be correlated with the calculated hydrogen-binding energy (HBE) on metal surfaces,^121^ and the HBE was shown to play a dominant role for the HER activity.^121−124^

The HER is one of the most studied electrochemical reactions, but compared to acidic conditions limited data is available in alkaline electrolytes. The HER activity decreases monotonically with increasing pH, supporting the theory of the higher HBE suppressing the catalytic activity.^125^ Furthermore, the HER takes place at more-negative potentials than the OER. Therefore, a higher number of stable materials are available for HER than for OER catalysts. These less-severe HER conditions also offer a wider range of electronically conductive and high-surface-area support materials for HER versus OER catalysts.

#### 3.2.1 Platinum Group Metal-Based Catalysts

**Figure 9:**
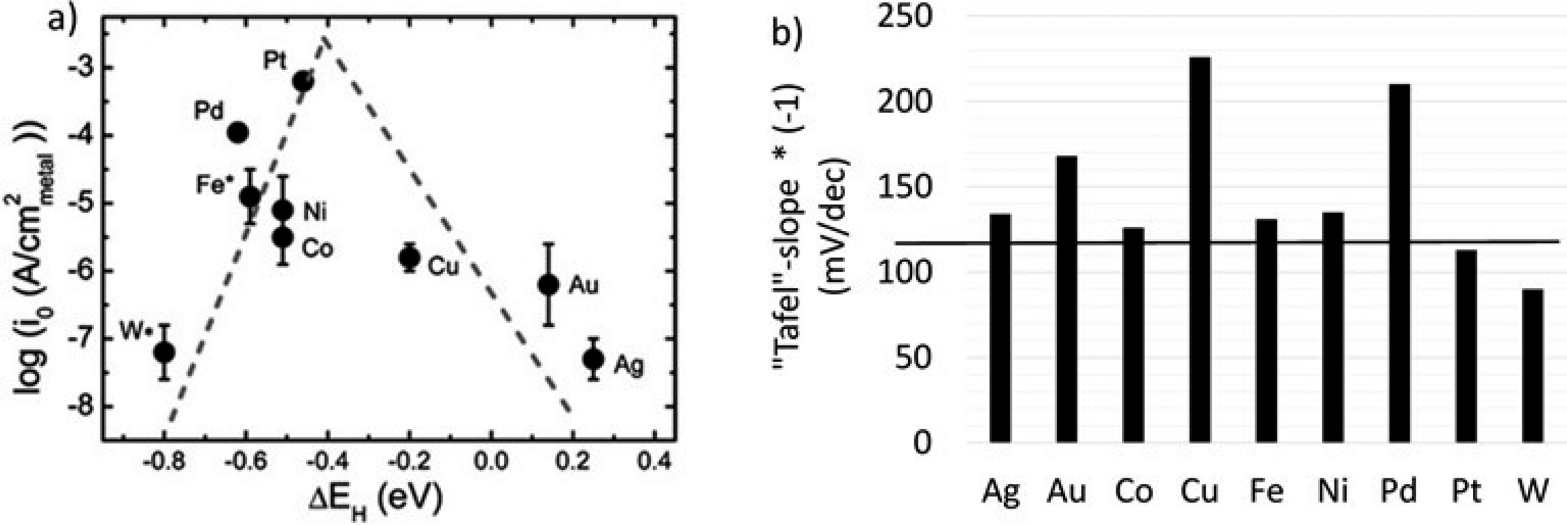
HER results measured for bulk, single-metal electrodes in 0.1 M KOH. (a) *j*_o_ versus calculated HBE (Δ*H*) values revealing a Volcano-plot relationship. (b) Tafel slope values as reported. The horizontal line at −120 mV/dec [shown in (b)] indicates the highest value a Tafel slope can display. (a, b) Reprinted with permission from ref (121). Copyright 2013 American Chemical Society.

Among many systems studied, Pt and Pt-based catalysts show the highest intrinsic HER activities in alkaline and acidic electrolytes.^126,127^ Typical *j*_o_ values for bulk and polycrystalline Pt measured in 0.1 M KOH are 0.62 ± 0.01 mA/cm_Pt_^2^, and the HER kinetics for Pt are slowed by 2 orders of magnitude in alkaline versus acidic media due to an extra water-dissociation step.^128^ Similarly, the Tafel slope of Pt is favored (i.e., lower), namely, −30 mV/dec, in acidic solutions versus approximately −120 mV/dec for alkaline solutions.^121,128^ The following order was extracted from HER measurements carried out in 0.1 M KOH using smooth, single-metal bulk catalysts: Pt ≫ Pd > Ni > Fe ≈ Co > W > Cu > Au > Ag.^121^Figure 9a shows that the exchange current density, *j*_o_, and HBE values follow a Volcano-plot dependence in alkaline electrolytes, as is the case for acidic media. The HER activities, measured as *j*_o_, of these bulk metal electrodes show up to ∼4 orders of magnitude differences. A closer inspection of the Tafel slopes (Figure 9b) reported for this series shows high slopes from −90 to −216 mV/dec and only two catalysts, namely, W and Pt, show actual Tafel slopes, i.e., values less than −120 mV/dec. In the case of W, it is questionable if the HER was actually studied on the metal surface because, in aqueous solutions, the surface of tungsten will be covered with oxides, which are difficult to reduce to the metallic surface state in this electrolyte. The same could apply to the Ni, Fe, and Co catalysts studied because surface oxides form easily on these metals, and their complete reduction to the metallic surface state can be challenging. Additionally, hydride incorporation into metals such as Ni and Pd can further complicate HER activity measurements. In fact, a recent study using ambient-pressure X-ray photoelectron spectroscopy (XPS) suggests the formation of Pt–H components and their transformation and/or H intercalation in subsurface Pt layers to possibly take place on Pt in alkaline conditions.^129^

Just as for acidic conditions, the surface orientation impacts the activity of a catalyst. The lower density and stepped surfaces of Pt are more active for the HER.^130^ Pt(110) exceeds the HER activities of Pt(100), and dense surfaces like Pt(111) show drastically lower activities.^130^ The use of single-crystal electrodes is not practical for AEMWE applications. However, the results show that tuning the catalyst’s morphology and working with nanoparticles can change the intrinsic activity in addition to increasing the surface-to-bulk atom ratio. The use and development of nanostructured and nanoengineered catalysts is important, but structural changes and agglomeration of small, specifically nanosized particles can take place during electrolysis, reducing the activity of a catalyst.^131^

The high cost of Pt is an issue for large scale applications. Correspondingly, Pt nanoparticles of <5 nm size, supported on carbon blacks such as Vulcan XC-72 and referred to as supported Pt/C catalysts, are often employed. These catalysts benefit from high ECSAs and correspondingly high mass activities. Sheng et al. carried out the careful extraction of *j*_o_ values and activation energies (*E*_act_) for the HER and H_2_ oxidation reaction (HOR) for bulk metal, polycrystalline Pt, and a commercial 46 wt % Pt/C catalyst in KOH electrolytes.^128^ The data shown in Table 3 suggest that the intrinsic exchange current density (*j*_o,intr_) and the *E*_act_ values are essentially the same for the bulk metal Pt and the 46 wt % Pt/C catalysts.

**Table 3:** Summary of Average HER/HOR Results for Polycrystalline Pt and Commercial Pt/C Catalysts^128^

	electrolyte	*j*_o_,_intr_^a^ (at 21 ± 1.5 °C) (mA/cm_Pt_^2^)	*j*_o_,_mass_^a^ (at 21 ± 1.5°C) (mA/mg_Pt_)	*E*_act_ (kJ/mol)	Tafel slope^a^ (at 21 ± 1.5°C) (mV/dec)
Pt (pc)^b^	0.1 M KOH	0.62 ± 0.01	n.a.^c^	28.9 ± 4.3	109
Pt/C^d^	0.1 M KOH	0.57 ± 0.07	0.35 ± 0.05	29.5 ± 4	n.r.^c^

a Measured at 21 ± 1.5 °C.

b Polycrystalline bulk metal Pt.

c n.a. and n.r. stand for not applicable and not reported, respectively.

d Commercial 46 wt % Pt/C (Tanaka Kikinzoku International, Inc.). Measured ECSA = 62 m^2^/g_Pt_.

**Figure 10:**
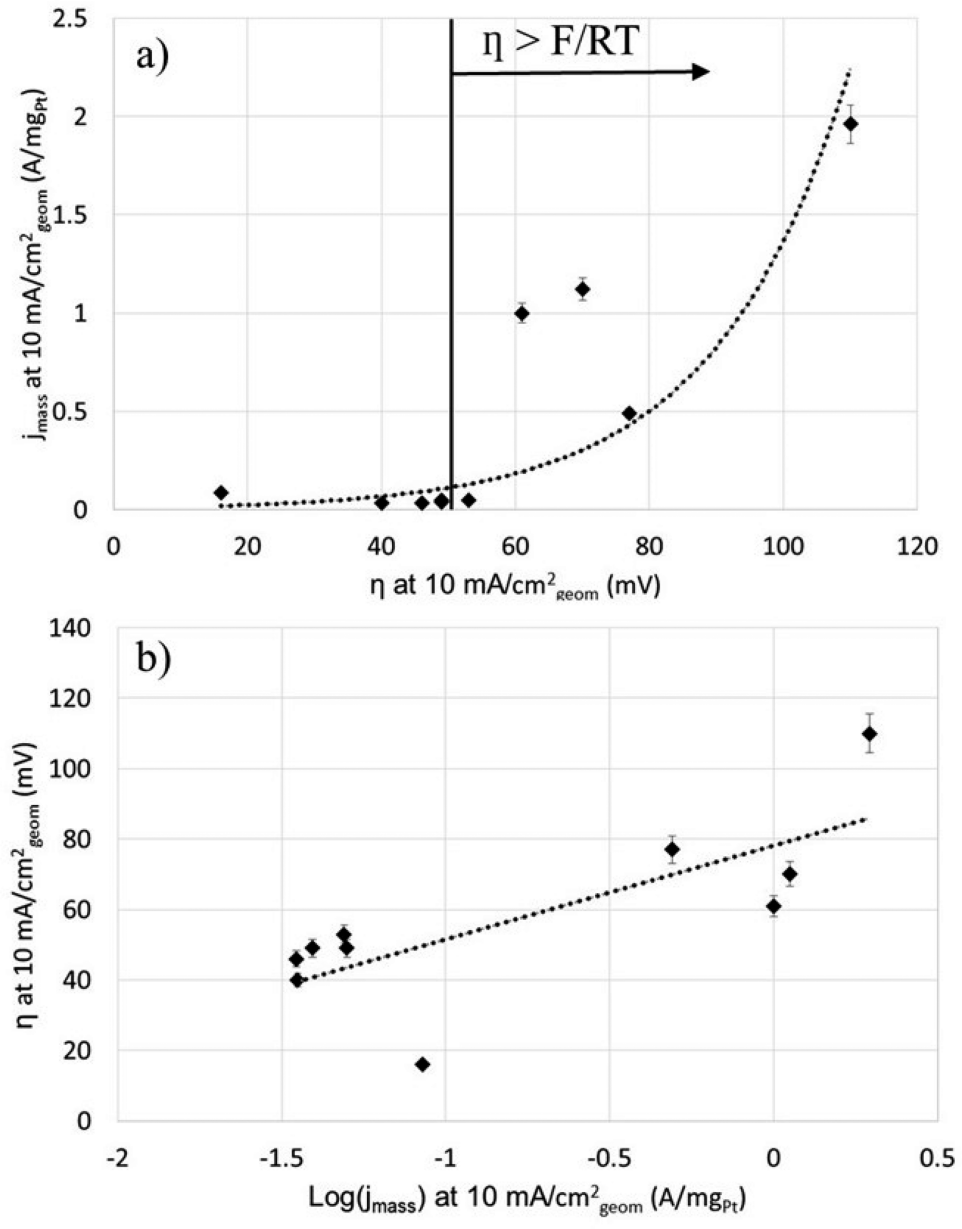
Mass current density (*j*_mass_) for Pt/C catalysts reported in the literature versus the corresponding η value, both of which were measured at 10 mA/cm_geom_^2^. Additional information about the Pt/C catalysts and the literature references are given in Table S1. (a) The data follow an exponential-type relationship, which is confirmed by (b), which shows essentially the same as (a) but as a plot of η versus the log 10 of *j*_mass_ of the Pt/C catalysts.

It is important to validate studies of new catalysts by HER activity measurements of a commercially available Pt/C catalyst. Table S1 shows a summary of literature data for Pt/C catalysts as well as for other HER catalysts. Some of the HER (and also OER) activity data tables shown in the Supporting Information were built using data made available by Kibsgaard and Chorkendorff,^132^ but many additional catalysts and other relevant metrics (when available) such as the Tafel slopes, η range used for the Tafel slope measurements, and ECSA values were added in this Review. The reported HER characteristics for the Pt/C catalysts (most are commercial catalysts from a number of suppliers) differ substantially. The Tafel slope values show a large variation among the Pt/C catalysts, the majority of which are reported as negative slopes ranging between 36 and 55 mV/dec, while the slopes of two Pt/C catalysts are close to −120 mV/dec. A closer inspection of the η range used to extract the Tafel slopes (Table S1) reveals that the two Pt/C catalysts with the higher, i.e., close to −120 mV/dec, slope were measured at a valid η (>*RT*/*F*) range of >0.05 V. Furthermore, the majority of the HER activities shown in Table S1 were measured at 10 mA/cm^2^ geometrical electrode area (cm_geom_^2^), which makes a direct comparison and validation of catalyst performances difficult because the catalyst loading on the electrode (mg/cm_geom_^2^) can differ significantly. Mass and surface area normalized HER activities measured at the same η value are better for comparison; however, data for such measurements are rare. A plot of the mass activity of the Pt/C catalysts versus the corresponding η values, i.e., both measured at 10 mA/cm_geom_^2^, is shown in Figure 10a and reveals an expected increase of *j*_mass_ with η in an exponential manner. The latter is confirmed by plotting the same data as η versus the log_10_ of *j*_mass_ (Figure 10b). Both plots demonstrate the scatter in the data, which can be at least partially ascribed to experimental variations as the majority of the studies use nonsteady-state polarization curves and different sweep rates for recording. The purpose of Figure 10b is to demonstrate the scatter in the results reported in different studies rather than suggesting the extraction of a Tafel slope, which would not be a valid approach using such data.

The measurement of intrinsic activities is needed and can be obtained for Pt-based catalysts because the ECSA of Pt can be estimated using the charge resulting from adsorption and desorption of H (H_ads/des_).^133,134^ For the 15 Pt/C catalysts shown in Figure 10, ESCA values for three catalysts are reported. Only one group reported data that allow the estimation of the intrinsic HER activity at the same η (of −70 mV), suggesting intrinsic activities of 0.88 and 1.4 mA/cm_Pt_^2^ for a commercial Pt/C and homemade Pt nanowire (NW) catalyst. The number of data points (measured at consistent conditions) is insufficient to draw conclusions and validate the activity values. However, the results emphasize the need for proper and consistent measurements and also for the establishment of a valid baseline using a Pt/C catalyst. Results reported for various HER catalysts are discussed in the following sections and will also be compared to the Pt/C activities shown in Table S1 and Figure 10.

##### 3.2.1.1 Combinations of Pt and Ni

**Figure 11:**
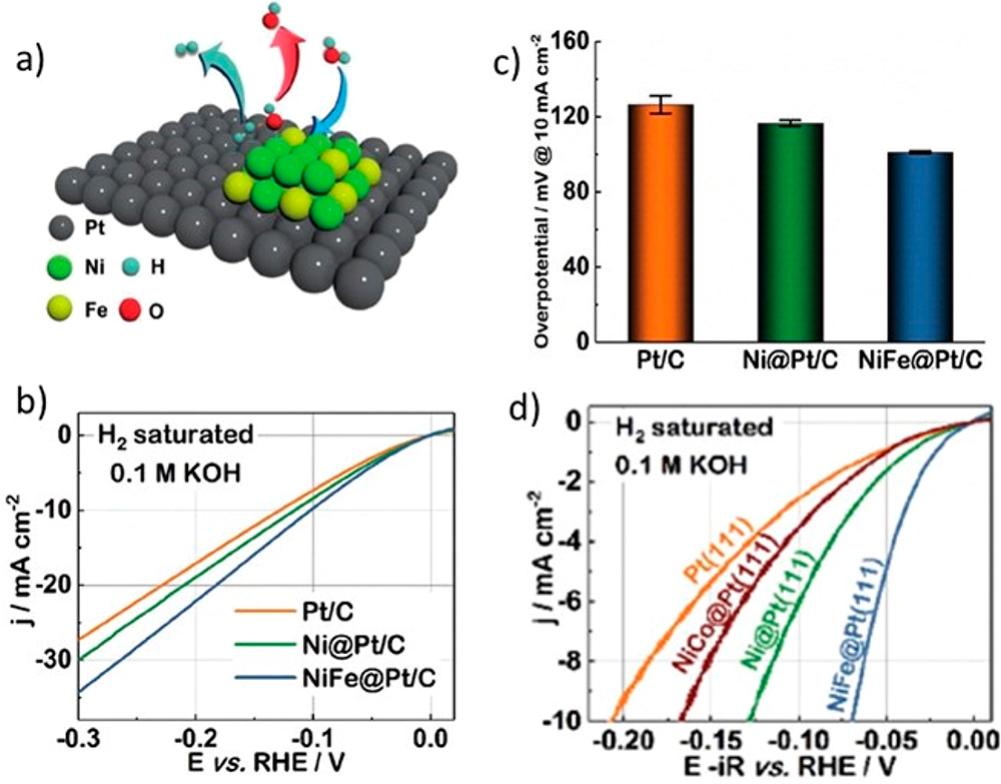
(a) H-spillover mechanism and enhancement of HER activities created by various Ni(OH)_2_-type clusters deposited on (b, c) Pt/C and (d) bulk Pt(111) crystals. A NiFe(OH)_2_ cluster on Pt is used to demonstrate the H-spillover mechanism in (a), while Ni(OH)_2_ and NiFe(OH)_2_ clusters are deposited on Pt/C powder catalysts for the polarization curves and η values shown in (b) and (c), respectively. (d) Polarization curves for NiCo(OH)_2_, Ni(OH)_2_, and NiFe(OH)_2_ clusters deposited onto bulk Pt(111). The abbreviations NiCo@, Ni@, and NiFe@ for the NiCo(OH)_2_, Ni(OH)_2_, and NiFe(OH)_2_ clusters, respectively, are used in the graphs. Reprinted with permission from ref (140). Copyright 2020 Wiley.

Combinations of Pt with Ni^135^ such as alloys and Ni deposits on Pt are recognized as being able to exceed the HER activity of Pt in alkaline media^135−139^ A synergistic effect between Pt and Ni exists, benefiting the HER. Xue et al. demonstrated this effect using a model catalyst formed by the growth of ultrathin Ni(OH)_2_ [and in subsequent work also thin NiFe(OH)_2_] clusters^140^ of 15–20% surface coverage onto Pt(111).^135^ The Ni(OH)_2_ clusters on Pt(111) demonstrated an 8-fold increase in the intrinsic activity compared to bare Pt(111), which was suggested to take place through a H-spillover mechanism from Pt to Ni(OH)_2_. The HER activity was further increased by adding cations such as Li^+^ to the electrolyte, which enhanced the formation of hydrogen intermediates. The same authors deposited such clusters on more practical Pt/C powder catalysts and also observed such a synergetic effect. Figure 11a shows a schematic demonstrating the H-spillover effect for the case of NiFe(OH)_2_ clusters on Pt. Furthermore, polarization curves (Figure 11b) and η values measured at 10 mA/cm_geom_^2^ for Pt/C and Ni(OH)_2_ or NiFe(OH)_2_ clusters on a Pt/C powder catalyst (labeled as Ni@Pt/C or NiFe@Pt/C, respectively) are shown in Figure 11c. It is seen that the NiFe(OH)_2_ clusters formed on the Pt/C powder show the highest HER activity. The same is the case for NiFe(OH)_2_ clusters formed on bulk Pt(111) crystals (Figure 11d). Furthermore, NiCo(OH)_2_ clusters on Pt(111) show the lowest HER enhancement, i.e., lower than Ni(OH)_2_ and NiFe(OH)_2_ (Figure 11d).

**Figure 12:**
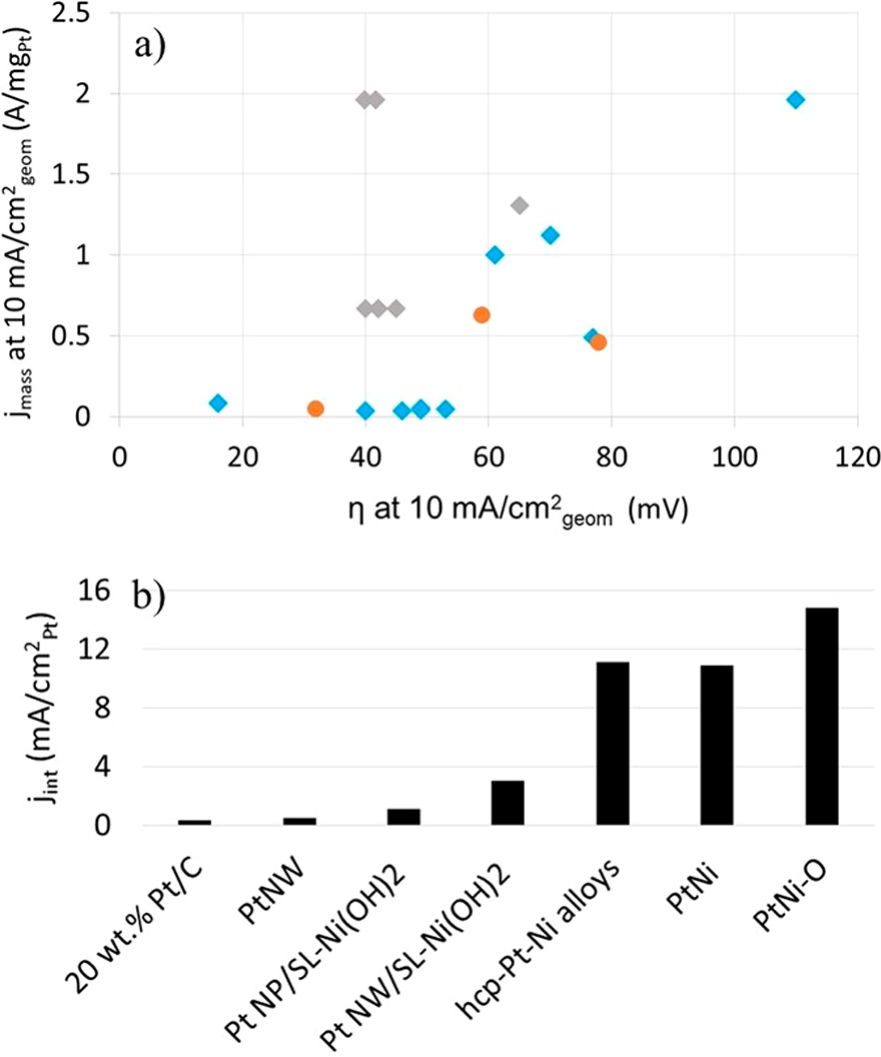
(a) Mass current activities per amount of Pt versus the corresponding η for various Pt–Ni catalysts, both measured at 10 mA/cm_geom_^2^. (b) Plot of the intrinsic activity per ECSA of Pt (*j*_int_) measured at η = 70 mV for two Pt-based and a number of Pt–Ni-based catalysts. The data used for (a) and (b) are shown in Tables S2 and S3, respectively. The blue diamonds represent Pt/C, the gray diamonds represent Pt_*x*_Ni_*y*_ alloys, and the orange circles represent the Pt nanosized catalysts wih Ni(OH) in (a).

A number of studies report the synthesis of various forms of combined Pt and Ni-based catalyst powders with the goal to produce catalysts of higher HER activities by introducing the synergetic H-spillover effect. In most cases, the mass activity per mg Pt and the η values were measured at 0.01 A/cm_geom_^2^ and are shown in Figure 12a. Data for the commercial Pt/C catalysts are also shown. Some authors also reported the intrinsic activities measured at η = 0.07 V and the ECSA values. These results are summarized in Figure 12b. Yin et al.^138^ formed Pt nanowires and also Pt nanoparticles on single-layer Ni(OH)_2_ sheets (the latter were formed by exfoliation of layered Ni(OH)_2_). At η = 0.07 V and in 1 M KOH, superior intrinsic activities (measured as *j* per Pt area) of up to approximately 8 and 3 times were reported for the two Pt catalysts formed on the single-layer Ni(OH)_2_ sheets compared to the commercial Pt/C and homemade Pt-only nanowires, respectively. The single Ni(OH)_2_ layers offer a high surface area for the dispersion of the Pt catalysts. However, a possible contribution of the high number of Ni(OH)_2_ surface sites to the HER was not considered in the *j*/cm_Pt_^2^ measurements and cannot be completely ruled out on the basis of the reported measurements. The authors further reported that the combination of the Pt nanowire with the single-layer Ni(OH)_2_ structure also increases the catalyst’s stability, although the stability experiments were carried out over a short period of 4000 s.

Abbas et al. deposited Pt nanoparticles of 1.7–3.1 nm onto nickel urchin-like structures, referred to as *x*Pt@Ni-SP.^141^ They reported that the HER mass activity per weight Pt in 1 M NaOH was up to 3.15 times higher for the *x*Pt@Ni-SP catalysts compared to a 40 wt % Pt/C commercial catalyst. Differences in the intrinsic activities, measured as A/cm_Pt_^2^, were smaller: the 0.75Pt@Ni-SP catalyst showed the highest increase of 1.3 times, while some *x*Pt@Ni-SP catalysts exhibited a lower intrinsic activity over the Pt/C catalyst. The use of the nickel-based support might be beneficial to the catalysts’ long-term performance, as the authors reported a higher stability of the *x*Pt@Ni-SP catalysts over the commercial carbon-supported Pt catalysts. Tafel slopes in the −30 mV/dec range were reported for all Pt_*x*(*x*>0.5)_@Ni-SP catalysts and the Pt/C catalysts, suggesting that the Volmer reaction was the rate-determining step. However, the reported Tafel slopes were extracted from nonsteady-state polarization curves. Chen et al. explored the deposition of Pt onto honeycomb-like NiO@Ni-film catalysts.^137^ The Ni films were actual Ni nanofoams that could also serve as current collectors in an MEA. The intrinsic HER activity per Pt surface area did not seem to vary remarkably among the catalysts. One catalyst, namely, Pt on the honeycomb-like NiO@Ni-nanofoam substrate, was reported to have a 15 times higher HER activity per mass of Pt compared to a commercial Pt/C catalyst. This increase may be at least partially due to a H-spillover effect. However, the direct deposition of the catalysts onto the current collector may also contribute to a higher mass activity by increasing the utilization of the catalyst (in this case the Pt onto honeycomb-like NiO@Ni-film) compared to a powder catalyst. Powder catalysts are typically transformed into electrodes using an ionomer and/or binder, which can block catalyst sites (see also section 6). Measurements of η at 10 mA/cm_geom_^2^ showed an increase of ∼40% for both the Pt onto honeycomb-like NiO@Ni-film and the Pt/C powder catalysts over a period of 24 h. All of these results show that the combination of finely dispersed Pt on high-surface-area nickel present as, e.g., Ni(OH)_2_ layers can potentially offer HER catalysts of higher mass activity per Pt. It is therefore not surprising that other metal additions such as Fe and Co are being explored. It was already mentioned that the deposition of NiFe(OH)_2_ clusters on Pt(111) crystals further promotes the HER activity, suggesting that Fe assists Ni in the water-dissociation step.^140^ It is also claimed that Fe increases the conductivity and the oxidation state of Ni in its vicinity. Wang et al. decorated Co nanowires grown on a Ti mesh with Pt–Co alloys.^142^ Only one of the Pt–Co catalysts exceeded the HER mass activity of the commercial Pt/C catalyst, which seemed to be measured in A per geometrical electrode area, and only the less-active catalysts showed stable catalytic activities for 50 h.

However, the highest HER activities among these types of catalysts seem to be achieved by Pt_*x*_Ni_*y*_ alloy particles, as suggested by the data also presented in Figure 12. Wang et al. prepared, by annealing, various Pt–Ni nanowire catalysts that were shown to consist of different alloy phases such as Pt_3_Ni_4_, Pt_3_Ni_3_, Pt_3_Ni_2_, Pt_3_Ni, and NiO_*x*_.^136^ They reported up to ∼12 times higher HER mass activity in 1 M KOH for their Pt–Ni nanowire catalysts than for a commercial Pt/C catalyst. The higher mass activity was assigned to the many interfaces of Pt_3_Ni and NiO_*x*_ being created upon an optimized annealing process. The NiO_*x*_ surface is proposed to accept the OH^–^ produced in the H_2_O splitting reaction, while nearby Pt sites accept the H_ads_ and produce the H_2_. The intrinsic HER activity was not measured, and the onset potential for the HER appears to be the same for all catalysts studied, including the commercial Pt/C catalyst.

##### 3.2.1.2 Ru HER Catalysts

**Figure 13:**
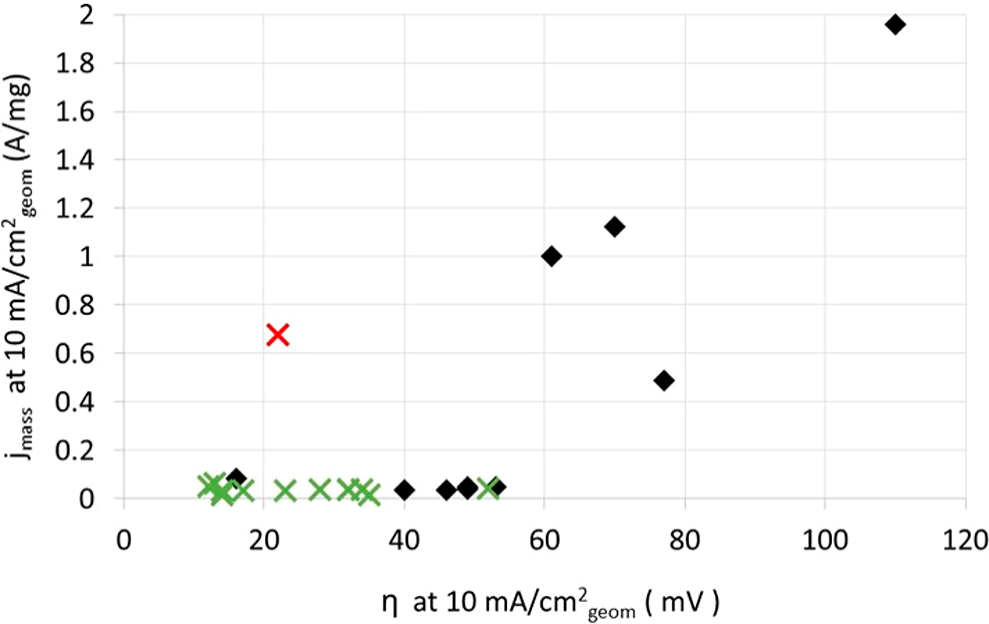
Mass activities (*j*_mass_) versus the corresponding η values of various supported catalysts, namely, Ru nanoparticles (green crosses), a 2.5 nm Pt_1_Ru_1.54_ alloy (red cross), and Pt/C (black diamonds). The *j*_mass_ and η values are measured at 10 mA/cm_geom_^2^ in 1 M KOH. The mass activities are measured in A/mg noble metal catalyst for the supported Pt_1_Ru_1.54_ alloy and the Pt/C catalysts, while in the case of the supported Ru catalysts, the mass activities are in mg per total catalyst, i.e., including the carbon support. Details about the catalysts, the actual values, and the corresponding references are given in Table S4.

Ru is another PGM that is attracting attention as apotential HER catalyst for acidic as well as alkaline electrolytes. The ∼65 kcal/mol H-bonding energy of Ru is similar to that of Pt.^141^ While not as expensive as Pt, Ru is scarce. Therefore, Ru will only become a viable candidate for large-scale AEMWEs if Ru catalysts of high HER activities and long-term stability can be made using affordable materials and routes for the synthesis of both the catalyst and the support. Recent activities on the development of Ru-based HER catalysts for AEMWEs have focused on the formation and anchoring of Ru and also PtRu alloy nanosized particles on conductive carbon-based supports. High-surface-area carbon supports such as phosphorus carbon nanosheets and N-doped holey two-dimensional carbon sheets consisting of repeat units of, e.g., C_2_N structures were synthesized to allow the anchoring of the Ru-based particles.^143^ Density functional theory (DFT) calculations suggest that the H-binding energy is lowered for Ru particles embedded into these C_2_N and C_2_N_2_ structures and that both the Ru and the adjunct carbon atoms act as catalyst sites.^141−144^ The HER activities and catalyst loadings on the electrode seem to be given as total catalyst mass, i.e., including Ru and other components such as the supports in many of these studies. Furthermore, ECSA measurements are rare, which may be due to the fact that the reliable extraction of the ECSA values for Ru-based catalysts can be challenging. Double layer capacitance values and CO_ads_ stripping measurements have been used to gain ECSA information, but Ru forms many different oxides at low potentials, each yielding a different *C*_dl_ value, and CO_ads_ only adsorbs on metallic surfaces.^110^ Similarly, the method of Cu underpotential (Cu_upd_) deposition can be applied to catalyst sites in the metal state but not to oxides.^110^ Nevertheless, according to thin-layer catalyst measurements, some of the Ru-based catalysts show promise, as shown in Figure 13. Figure 13 is a plot of the mass activities versus the corresponding η values (both measured at 10 mA/cm_geom_^2^) for Ru-based versus Pt/C powder catalysts. The results for the majority of HER activities for the Ru catalysts are underestimated due to the fact that the total catalyst weight is used for the mass activity calculation (the Ru loading for many of these catalysts does not seem to have been determined), while the activities for the Pt/C catalysts (black diamonds) and the supported Pt_1_Ru_1.54_ alloy (red cross) catalysts are per total noble metal weight. Figure 13 suggests that the Ru-based catalysts show mass activities as high as and exceeding that of Pt/C. In the case of the 2.5 nm Pt_1_Ru_1.54_ alloy catalyst formed on phosphorus carbon nanosheets, the activity per total noble metal loading seems to exceed that of the commercial Pt/C catalysts.^145^

The 2.5 nm Pt_1_Ru_1.54_ alloy catalyst reported to exceed the Pt/C catalyst and a homemade Pt catalyst supported on phosphorus carbon nanosheet were made by Li et al.^145^ The authors suggested that the observed enhancement of the Pt_1_Ru_1.54_ alloy catalysts was due to the electronic interactions between the nanosized Pt_1_Ru_1.54_ catalyst and the phosphorus carbon nanosheet, thus resulting in the enhancement of the H_2_O dissociation kinetics.

**Figure 14:**
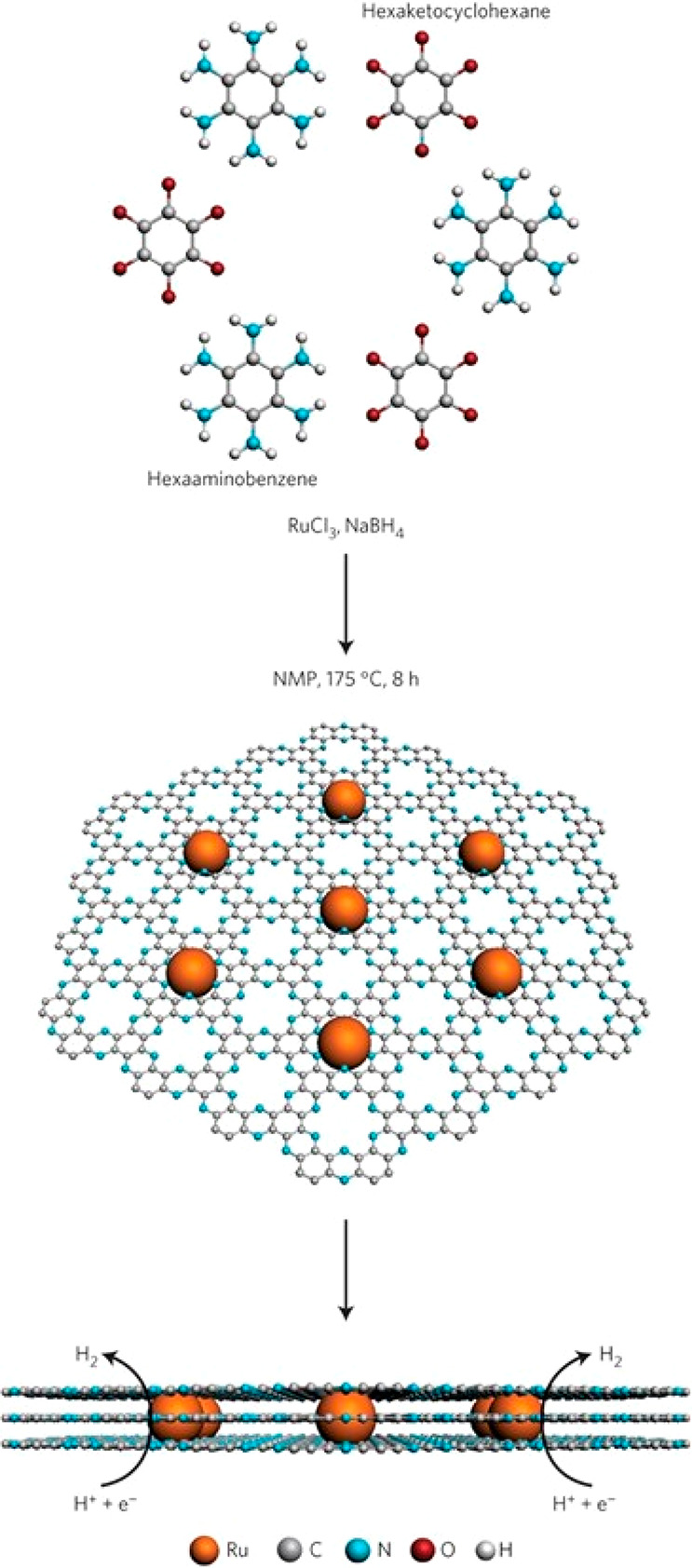
Schematic of the synthesis to form nanosized Ru catalysts embedded within holey, two-dimensional carbon nanosheets made of repeating C_2_N units. Reprinted with permission from ref (143). Copyright 2017 Springer Nature.

Mahmood et al. dispersed 1.6 nm averaged size Ru particles within holey, two-dimensional carbon nanosheets made of repeating C_2_N units.^143^Figure 14 demonstrated the formation and distribution as well as the embedment of the Ru nanoparticles within the layers of the high-surface-area nanosheets. The authors used Cu_upd_, CO_ads_ stripping voltammetry, and H_ads/des_ charges to estimate ECSA values and reported the number of active sites for the Ru/C_2_N to be ∼18% below those of the Pt/C_2_N and Pt/C catalysts. On the basis of the number of active sites estimated from these three methods, the TOF per active catalyst site (i.e., the intrinsic HER activity of the Ru/C_2_N) exceeded that of the commercial Pt/C catalysts by a factor of ∼1.7. It is assumed that the ECSA measurement for the Ru/C_2_N catalyst reflected the Ru sites in the metallic state, as discussed earlier. Only a small drop in the HER activity was reported after 10 000 potential cycles between 0.2 and −0.1 V versus the reversible hydrogen electrode in 1 M KOH. Details about the electrochemical experiments such as whether a high-surface-area Pt-free counter electrode was used were not given.

Other studies (the results of which are included in Figure 13 and Table S4) also focused on the dispersion of Ru on high-surface-area supports. Lu et al.^144^ formed Ru nanowires on N-doped carbon nanowires, Zheng et al.^146^ formed Ru particles of an average 2 nm size in C_3_N_4_ matrixes, while Xu et al.^147^ formed Ru particles of an average 1.5 nm size by pyrolysis at 350 °C using a carbon support of unspecified origin. These catalysts approached the mass activities of commercial Pt/C catalysts at comparable η values (Figure 13). As already noted, the authors appeared to give the loadings of the Ru catalysts as total catalyst loading, i.e., including the carbon support, while the mass activity for the Pt/C is per Pt metal.

Recent studies also involve the Ru–Ni system, which again often focuses on the dispersion of Ru (in the nanosized range) on Ni(OH)_2_-type matrixes, partially with the goal to take advantage of the two-dimensional high-surface-area structures that Ni-hydroxides can form. Ding et al.^148^ formed Ru–Ni nanoplates of ∼10–30 nm size, and Chen et al.^149^ formed RuNi as layered sheets (RuNi-LMH) on nickel nanofoams. Both groups reported lower η values measured at 10 mA/cm_geom_^2^ compared to Pt/C catalysts. Similar to the RuNi system, RuCo catalysts are being explored. A nitrogen-doped carbon-supported Ru–Co alloy catalyst, formed by using the optimized annealing temperature of 600 °C, was reported to show a lower η of 34 versus 49 mV (measured at 10 mA/cm_geom_^2^) versus a commercial Pt/C catalyst. The total catalyst loadings were ∼0.255 mg/cm_geom_^2^, and the RuCo loading on the carbon seemed not to have been measured.^150^ The addition of Co to Ru was proposed to enhance the H* recombination step.^150,151^ Mao et al. formed Co-substituted Ru nanosheets and reported that the ∼30 nm Co atoms distributed among the Ru lattice had kinetics (measured as TOF) comparable to those of commercial Pt/C, Ru/C, and homemade RuCo alloy catalysts.^152^ Details about the calculation of the TOF numbers and the loading of the Pt/C catalyst do not seem to be presented.

In conclusion, activities reported for Ru-based HER catalysts, focusing on the dispersion and anchoring of the Ru catalysts, have shown promise in thin-layer electrode studies. However, a full understanding will require detailed analyses of these catalysts under AEMWE conditions as well as the determination of the Ru content of the catalysts. True Tafel slope measurements carried out under steady-state conditions within a valid η region are also needed. For water electrolysis in alkaline conditions, the stability of Ru is a concern, and it has been established that Ru catalysts have poor stability in alkaline conditions within the OER potential range. Therefore, thorough long-term stability measurements of these proposed Ru-based HER catalysts under conditions reflecting real AEMWE operations, i.e., involving intermittent periods and possible potential reversals, are important.

#### 3.2.2 Ni-Based Catalysts without PGMs

##### 3.2.2.1 Ni Metal HER Catalysts

Ni is an abundant metal and is used in traditional WE electrolyzers as an HER and OER catalyst, thus making it a candidate of high interest to replace Pt- or Ru-based catalysts for alkaline conditions.^153−155^ Ni metal shows good water adsorption, albeit the hydrogen-bonding energy to Ni metal is high and in general the rate-determining step is the H* recombination reaction.^156,157^ HER activities of Ni-only catalysts are lower than those for Pt/C catalysts; for example, at 10 mA/cm^2^, metallic Ni shows an ∼0.15 V higher η than that observed for Pt/C catalysts.^157^ Also, the HER activity of metallic Ni tends to decrease with the time of electrolysis, which is often attributed to hydride incorporation into the Ni lattice in the bulk and at the electrode surface.^71,158^ This is specifically strong for catalysts of small grain size, which correspondingly possess a high number of grain boundaries, where preferential H_2_ adsorption takes place.^159,160^ Corrosion is another factor eventually reducing the HER activity during electrolysis, as are changes in alkaline concentration induced by OH^–^ adsorption.^153^ Ni metal surfaces can adsorb oxygen from the electrolyte and react to form NiO.^161^ NiO is transformed into NiOOH when cycled into positive potential regions, allowing the electrolyte to adsorb onto the surface, reacting with the NiO to form NiOOH penetrating further into the catalyst that results in lower HER activity.^161^

**Figure 15:**
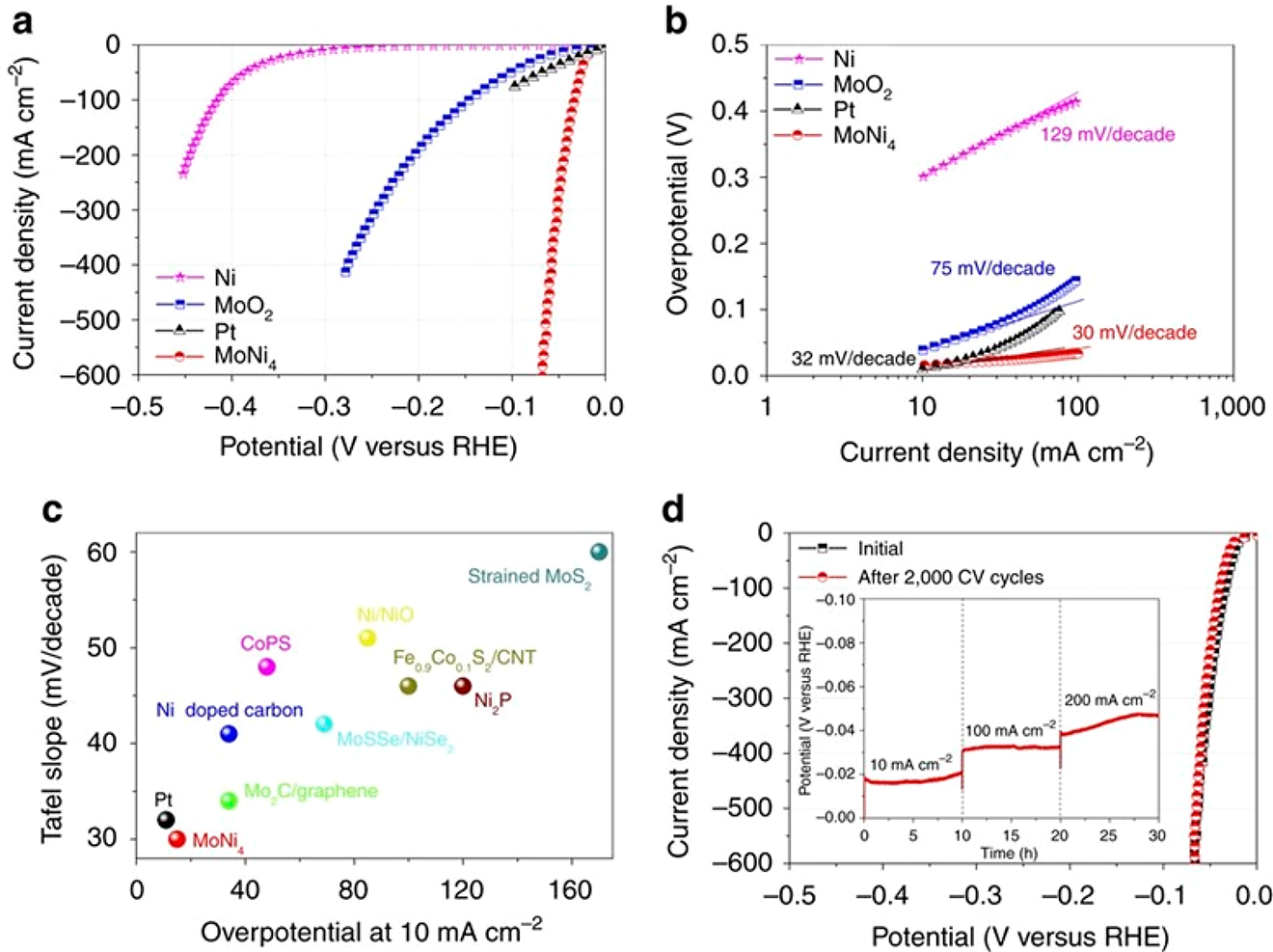
HER data extracted for Ni, Pt, and two Mo-based catalysts in 1 M KOH. (a, b) Slow-sweep polarization curves and Tafel slope values extracted from the polarization curves, respectively. (c) Comparison of Tafel slope values to other catalysts reported in the literature. (d) Results for a stabilization test carried out under potential cycling for the MoNi_4_ catalyst. Reprinted with permission from ref (162). Copyright 2017 Springer Nature.

Alloying of Ni has been shown to change the HER activity, as for, e.g., binary NiMo and ternary NiCoMo alloys. An example of alloying Ni and Mo is shown in Figure 15, where the MoNi_4_ catalyst is reported to have a higher activity than Pt, although the loading of the MoNi_4_ on the current collector does not seem to be known.^162^ Other approaches are the interstitial doping of nickel with, e.g., nitrogen. Using this concept, Ni_3_N nanoparticles (np-Ni_3_N) were prepared and activities approaching those of commercial 20 wt % Pt/C and 20 wt % PtRu/C catalysts were reported.^160^ However, the active catalyst component loading of the np-Ni_3_N catalysts was significantly higher than those for the Pt/C and PtRu/C catalysts, namely, 0.16 mg_Ni3N_/cm_geom_^2^ versus 0.01 mg_Pt_ or _PtRu_/cm_geom_^2^. The np-Ni_3_N catalyst is embedded in an N-doped graphitic support structure, which is proposed to alter the intrinsic catalytic properties of the Ni and possibly also stabilize the catalyst nanoparticles. Interstitial N-doping and embedment into the N-doped graphitic support of the Ni was carried out via a two-step reaction. The two reaction steps involved the decomposition reaction of preformed K_2_[Ni(CN)_4_] to form nanosheets made of Ni-cyano compounds at 450 °C followed by N-doping in a NH_3_ atmosphere also at 450 °C to form the 5–20 nm sized Ni_3_N nanoparticles. The N-doped graphitic lattice was formed simultaneously during this process.

CeO_2_ has been proposed as beneficial catalyst support to anchor metal catalyst sites. The system is of interest for both the HER and OER and may act as a bifunctional electrocatalyst.^163−167^ The metal-oxide interface of Ni and CeO_2_, with the latter being deposited on carbon nanotubes (CNTs), was reported to show a synergistic effect, and DFT calculations suggest that the interactions of Ni with CeO_2_ benefit the HBE, matching that of Pt/C.^163^ A lower η value for the Ni-CeO_2_/CNT catalyst compared to Ni/CNTs and CeO_2_–CNTs catalysts was reported, but the η value was higher than that measured for a commercial Pt/C catalyst of a 40% lower total metal loading. The addition of CeO_2_ was observed to enable the formation of Ni particles of 4 nm size, i.e., much smaller than the 50–100 nm Ni particles formed on CNTs free of CeO_2_. Therefore, some of the observed increases in the HER activity are likely due to an increase in the ECSA caused by the smaller Ni particle size of the Ni-CeO_2_/CNT versus Ni/CNT catalysts.

##### 3.2.2.2 Non-PGM Ni Alloys and Mixtures: The Addition of Mo

**Figure 16:**
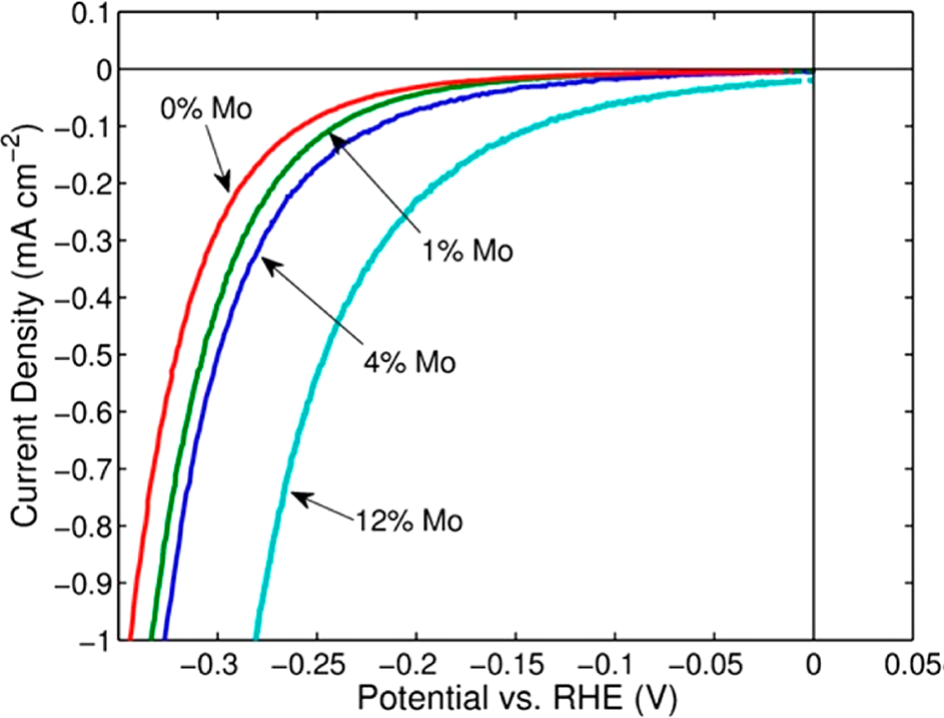
Polarization data of commercially available and metallurgically prepared Ni and Ni–Mo alloys with varying Mo content. Experiments were performed in 2 M KOH solutions. The Ni and Ni–Mo alloy samples were coin-sized samples prepared by cutting cylindrical rods and were carefully polished to create a smooth surface. Reprinted with permission from ref (178). Copyright 2013 American Chemical Society.

Other attempts to increase and stabilize the activity of bare Ni catalysts involve the formation of binary and ternary alloys of Ni with different elements such as Co, Fe, Mo, Ce, Zn, and Cu to improve the catalytic activity, prevent hydride formation, and achieve a higher stability.^168^ Many of these studies originate from the development of HER catalysts for traditional alkaline WEs and also for artificial photosynthesis devices.^169,170^ Some reports suggest that Ni–Mo alloys have the highest HER activity among non-PGM catalysts^30^ and that the activity of the Ni–Mo alloys is further improved for ternary alloys.^171−174^ While Ni–Mo alloys show high HER activities among non-PGM catalysts, the actual measurements are not always conclusive due to issues measuring the ECSA and the intrinsic activities of these catalysts accurately.^175^ These measurements are complicated by the fact that catalysts of the Ni–Mo combination can have widely different particles sizes and porous structures. In addition, the Ni–Mo system exhibits a pseudocapacitance in the potential region, typically used to estimate the ECSA value.^175^ Therefore, increases in HER activities reported for Ni–Mo catalysts have been argued to be due to higher surface areas rather than actual beneficial intrinsic catalytic effects.^176,177^ Nevertheless, it has been shown using nonporous catalysts that Ni–Mo alloys can exhibit increased HER activity over Ni-only catalysts, as demonstrated in Figure 16. Figure 16 shows polarization curves for metallurgical Ni and Ni–Mo alloys of different compositions made by cutting the metallurgical rod into disc-shaped electrodes of the same size. The surfaces of the disc-shaped electrodes were carefully polished to produce smooth surfaces. However, Ni–Mo alloys do not yield the HER activities needed for large-scale AEMWE applications. The stability of Ni–Mo alloys also needs to be proven. Ni–Mo alloy particles of 50–200 nm size have shown stable currents of 0.02 A/cm_geom_^2^ over 100 h in 2 M KOH using a catalyst loading of 1 mg/cm_geom_^2^, but currents of 0.02 A/cm_geom_^2^ are low and an analysis of dissolved metals was not performed.^178^

The addition of Mo to Ni, to form intermetallics or disorganized compounds, has been reported to improve the stability and the HER activity compared to Ni-only catalysts. In intermetallic compounds, the atomic fraction of Mo is much larger than that in disordered structures. Examples of intermetallics of Ni and Mo include Ni_7_Mo_7_, Ni_3_Mo, and Ni_4_Mo. In a recent study, polished samples of Ni_7_Mo_7_, Ni_3_Mo, and Ni_4_Mo were tested for the HER, and the metal dissolution was measured.^179^ Substantial dissolution of Mo occurred for Ni_7_Mo_7_, leading to an increase in the ECSA, while Ni_3_Mo and Ni_4_Mo did not show dissolution at potentials below 0 V versus the reversible hydrogen electrode. However, the stability range of Ni_3_Mo appears to be relatively narrow (between −0.25 and −1 V versus the reversible hydrogen electrode), and dissolution was reported to take place at open-circuit potentials.^117^ In comparison to intermetallics, in disordered structures the Mo content only needs to be a few atom % to be effective for the HER.^175,179^ For disordered structures, a Mo content of ∼10 atom % leads to the highest activity among Ni–Mo disordered catalysts.^180^

Many studies involve unsupported Ni–Mo catalysts of several hundred of nanometers in size.^178^ This is much larger than the sub-5-nm size that is typical for Pt/C catalyst,s and the larger size is at least partially responsible for the lower HER mass activities compared to Pt/C. To improve the electrical connectivity, Ni–Mo-based catalysts have been directly deposited onto Ni foam current collector substrates.^181,182^ A study of the formation of a ternary Ni_*x*_Mo_*y*_Fe_*z*_ alloy on Ni foam reported significantly lower η values than that for Ni only; however, the η values are still higher than that for Pt, and the catalyst loading is not known.^174^ Another two studies reported improved HER performances of their catalysts over commercial Pt/C powders.^182,183^ However, the loadings of the Ni–Mo catalysts were higher than the Pt/C catalyst loadings: approximately 55.8 and 44.3 mg/cm_geom_^2^ of MoNi_4_/MoO_2_@Ni versus 2 mg/cm_geom_^2^ Pt/C^182^ and 5.9 mg/cm_geom_^2^ MoNi_4_/MoO_3–*x*_ versus 1 mg/cm_geom_^2^ Pt/C.^183^ Additional studies are needed to deposit lower amounts of Ni–Mo-based catalysts directly on the porous Ni current collectors, and as already mentioned, the long-term stability of Ni–Mo catalysts under AEMWE operating conditions needs to be proven.

In conclusion, it appears that the intrinsic activity of the combined Ni and Mo catalyst system can be higher than that for Ni-only catalysts, although the dissolution of Mo under intermittent electrolyzing conditions at high pH could be an issue. Many Ni–Mo catalysts are unsupported, representing particles in the tenth of a nanometer size range, and are unlikely to be comparable to the mass activity that can be achieved for the catalytically very active and nanosized catalysts such as Pt/C, PtNi/C, and various supported Ru catalysts discussed earlier.

#### 3.2.3 Other HER Catalyst Groups

##### 3.2.3.1 Mo Carbides

Another class of HER catalysts consists of Mo carbides, borides, and sulfides. Mo_2_C, MoC, or Mo catalysts embedded in nanocarbons,^184^ carbon nanosheets, CNTs, or boron–carbon–nitrogen are examples of Mo catalysts studied for the HER. Commercial Mo_2_C has shown an ∼5 times higher η than Pt/C.^184^ Encapsulation of Mo_2_C in nitrogen-doped porous CNT was reported to benefit the HER, and a η value (measured at 10 mA/cm_geom_^2^) of 0.045 V for the embedded Mo_2_C compared to 0.033 V for the Pt/C catalyst was reported.^185^ A high loading of the embedded Mo_2_C catalyst of 0.728 mg/cm_geom_^2^ was used. Other studies also reported that encapsulation benefited the HER activity by increasing the number of active sites with better water adsorption properties.^186^ Again, these studies tend to use high catalyst loadings to achieve the low η values reported, and in some cases Pt wires were used as counter electrodes. (The use of a Pt counter electrode can result in Pt dissolution and deposition of the dissolved Pt on the working electrode, i.e., onto the catalyst, which in turn can result in incorrect high HER activities.) Encapsulation has been reported to increase the catalyst’s stability by reducing both corrosion and agglomeration of the catalyst particles.^185−187^ Other results suggest that graphene-based structures offer electronic benefits, although the anchoring of the catalyst onto the graphene will need to be addressed.^185−190^ Mo-nitrides have similar HER activities as carbides,^187,191^ and the combination of Mo_2_C and Mo_2_N has been reported to lower the η value.^187^ In all cases, stability appears to be a recurring problem. Similarly, MoB also shows unstable performance in alkaline solutions.^192^

##### 3.2.3.2 Transition Metal Phosphides and Sulfides

In acidic electrolytes, TM phosphides^193^ and sulfides^194−196^ have been reported to show notable HER activities. Therefore, Ni and cobalt phosphide catalysts such as Ni_2_P,^197^ Ni_5_P_4_, and Ni_3_P^198^ and CoP,^199,200^ Co_2_P, and CoP_2_, respectively, were also studied in alkaline media, but fast deactivation was observed.^198^ Poisoning of the catalyst surface or catalyst dissolution were suggested as possible mechanism for the observed deactivation.^197,199^ Phosphorus can perform in much the same way as N. These materials have high electrical conductivity and allow for higher catalyst loadings and high current densities, but the rapid catalyst deactivation is an issue.^198,201^

### 3.3 OER Catalysts

#### 3.3.1 OER Reaction Mechanism and Stability Consideration

The OER involves four charge-transfer steps as follows, where the * indicates surface-adsorbed species:^202^
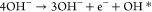
9
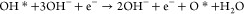
10
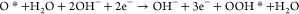
11
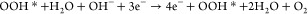
12

The OER free-energy diagram has an individual step height of 1.23 eV and a total change in free energy of 4.96 eV at standard conditions.^202^ As already discussed, the OER is a sluggish reaction. Changes in the binding energy of the reaction intermediates with the catalyst will change the overpotential. The minimal η for a catalyst surface that binds a reaction intermediate strongly is defined by the breaking of the reaction intermediate bond with the catalyst surface. Catalysts such as Mn, Co, Ir, and Ru oxides bind the reaction intermediates formed in the OER strongly and are predicted to show a minimum η of 0.37 V.^203^ NiO and TiO_2_ are examples of catalysts that show weak binding energies for OER intermediates and are defined by eqs 9 and 10.^203^

**Figure 17:**
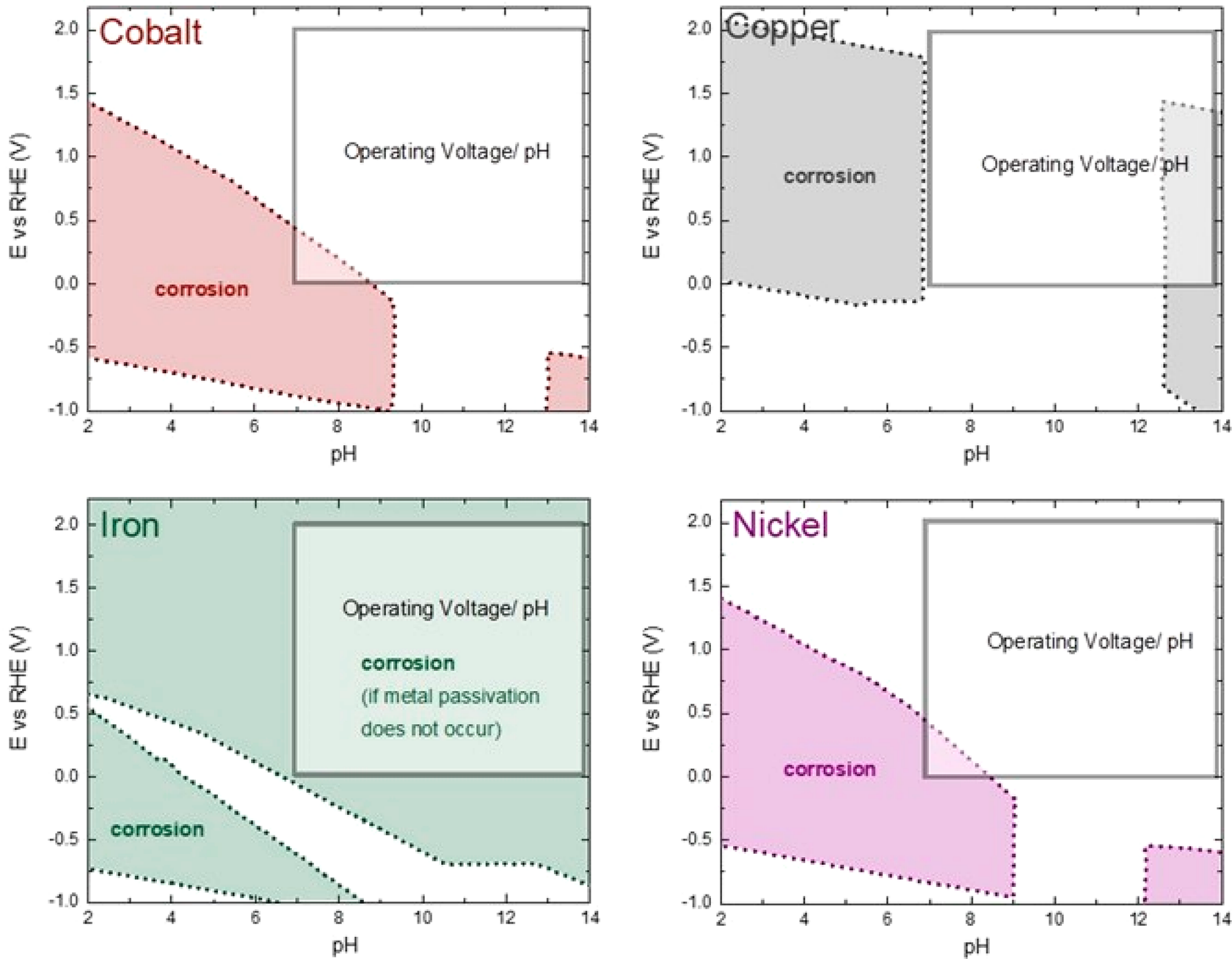
Pourbaix diagrams of cobalt, copper, iron, and nickel in aqueous electrolytes at ambient pressure and 25 °C. The inset shows the voltage–pH range that an anode catalyst may experience in an AEMWE. The diagrams were constructed from ref (204).

In the case of the acidic conditions of PEMWEs, only PGM OER catalysts have proven to show the stabilities needed for real applications. The alkaline pH of AEMWEs is viewed to offer a wide range of non-PGM OER catalysts, including transition metals such as Ni, Co, Fe, and Cu. However, this view is too simplistic, as long-term stability is an issue for many TM catalysts and also for catalyst supports. Therefore, the number of materials suitable as OER catalysts and supports is limited. Both the IrO_2_ and RuO_2_ OER catalysts, which are typically used in PEMWEs, show a lack of long-term stability in alkaline conditions. The RuO_2_ is the least stable of the two oxides, and the metallic counterparts of the oxides, i.e., Ir and Ru metal electrode catalysts, show poor stability.^204^ The stability issue is demonstrated in the Pourbaix diagrams shown in Figure 17 for Ni, Cu, Fe, and Cu, i.e., for four TMs of high interest as catalyst or catalyst components for AEMWE OER catalysts. The insets show the voltage and pH domains of relevance to AEMWEs considering different AEMWE feed modes, namely, water and dilute electrolytes. These domains are pH ∼7 for water, pH ∼9 for KHCO_3_, and a pH range of 9–12 for dilute NaOH or KOH. The colored areas suggest regions of corrosion for the corresponding element. An important point to note is that the stability of these TMs not only depends on the potential region but also on the pH and, hence, also on the nature of the solution feed to the AEMWE cell. The diagrams suggest that the stabilities of Co and Ni are jeopardized for pH 7 but are promising for a pH range exceeding 9. Indeed, NiO is known to have long-term stability at high pH and is used as an OER anode in traditional WEs. Copper is also suggested as a candidate for the pH range of ∼9–12. The diagram shown for Fe in Figure 17 ignores the formation of passivating iron oxides and shows that corrosion of iron may well occur under AEMWE operating conditions. It needs to be noted that Pourbaix diagrams are only guidelines based on thermodynamic information. They do not include reaction kinetics information, and experimental verification of the catalyst stability is needed. In addition, the stability of a catalyst is influenced by its chemical and physical structure, including its physical size. Nevertheless, Pourbaix diagrams provide insights and initial material stability guidelines.

#### 3.3.2 Challenges for OER Catalyst Supports

The harsh conditions of the OER also severely limit the number of stable support materials available for OER catalysts. The high surface area and electronically conductive carbon supports such as Vulcan XC-72, graphite, or potentially even CNTs, which are often used for the HER catalysts, are not suitable as a support for OER catalysts because carbon is easily oxidized and consumed during the OER. Therefore, non-carbon-supported OER catalysts are typical for AEMWEs, even though carbon-supported TM OER catalysts (including graphene, organic frameworks, and CNT supports) have been used, mainly at short experimental time scales. As already mentioned, the electronic conductivity of the support (or the bulk of a catalyst) also plays a role in the creation of effective OER catalysts. In fact, a conductive support can reduce electronic-conductivity limitations of certain catalysts, and, in the case of very thin (on the atomic-layer scale) catalysts, a support can alter the electronic properties of a catalyst and its lattice constants.^203^

#### 3.3.3 OER Activities of IrO_2_ and RuO_2_

**Figure 18:**
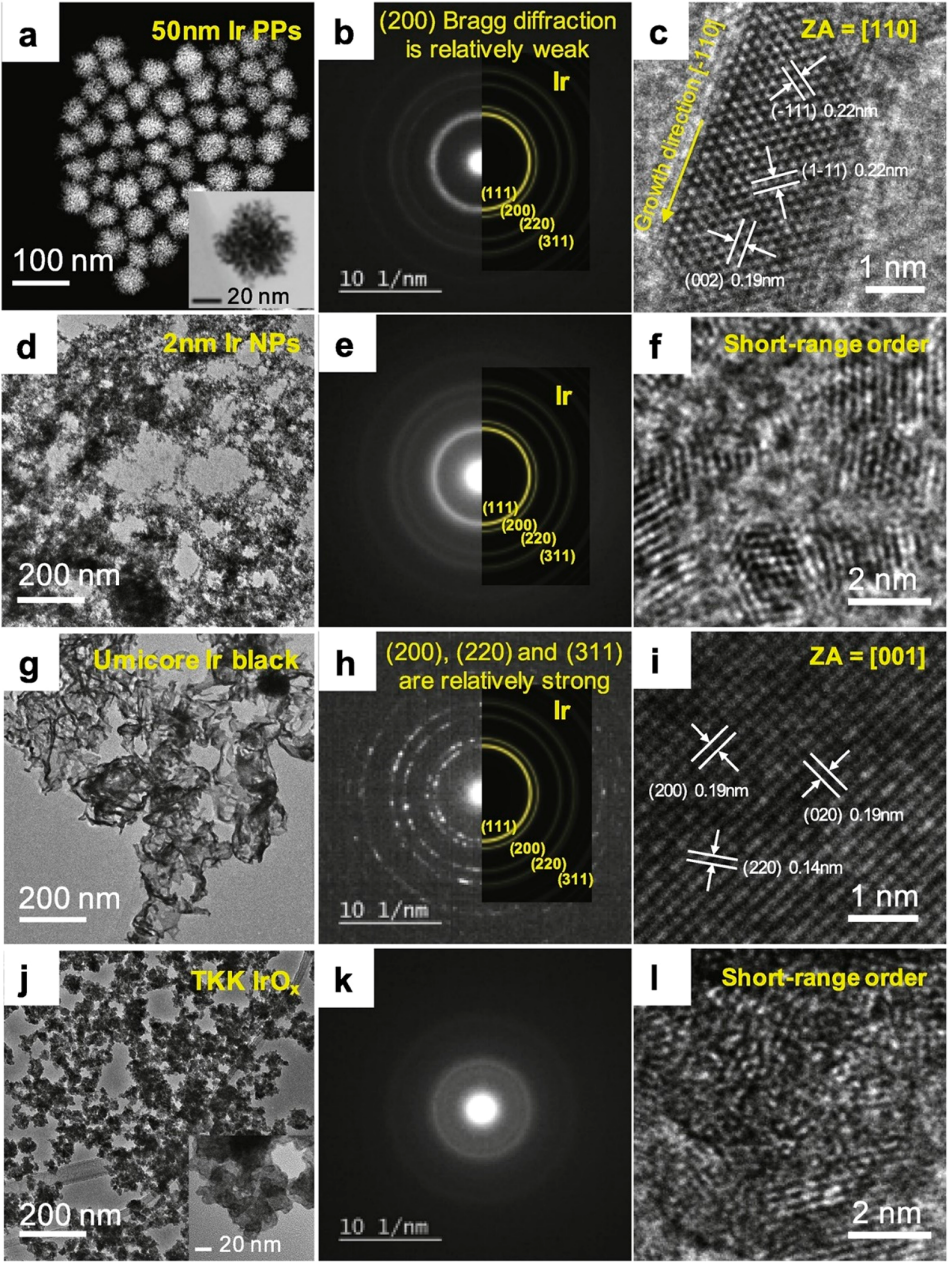
Transmission electron microscopy images for various Ir-based catalysts are as follows: (a–c) Ir particles, (d–f) nanosized Ir particles, (g–i) Ir black from Umicore, (j–l) amorphous IrO_*x*_ from (a) TKK and (b) the rutile form of IrO_2_. Reprinted with permission from ref (207). Copyright 2019 Elsevier.

Research for OER catalysts for AEMWEs has become extensive over the past decade. Many different synthesis conditions are used, and the catalysts are not always fully characterized in terms of their size and structure. As for the HER catalysts, the majority of the OER catalysts studied for AEMWE applications are powder catalysts, and the activity of newly prepared OER catalysts are often measured in thin-layer electrode setups and/or directly in a single AEMWE cell; also, the establishment of a baseline catalyst is needed. OER activity comparisons to commercial Ir-oxide catalysts, which are still considered state-of-the-art catalysts in terms of initial activity, are reported in some studies. However, the reported OER activities for Ir-oxide catalysts vary significantly. This may be partially due to the different forms and properties Ir-oxide can have depending on the method used for their preparation. Some of the possible differences are demonstrated in Figure 18, which shows transmission electron microscopy images for four different Ir-oxides. Ir-oxide powder catalysts are often made by thermal composition of a precursor salt like hydrous IrCl_3_. It is known that the annealing temperature influences the ECSA, water content, and likely the electronic conductivity and crystallinity of the resulting oxide catalysts, and a higher temperature results in a more crystalline and compact oxide.^205^ Amorphous IrO_*x*_ has a higher catalytic activity for the OER than that for the crystalline and rutile IrO_2_, which may be due to the open structure of the hydrous and amorphous IrO_*x*_ versus the compact structure of IrO_2_. However, the stability of the former is lower.^116^ A hydrous and amorphous IrO_*x*_ form can also be formed on the surface of an Ir metal, possibly assisting with the OER activity.^206^ Many commercial suppliers sell Ir-oxide powders as IrO_2_ or hydrous IrO_2_·H_2_O. Researchers should develop a practice of characterizing the as-received commercial Ir-oxides to understand the type of Ir-oxide structure being studied. At a minimum, the characterization of the as-received Ir-oxide including X-ray photon spectroscopy (XPS) of the Ir and O regions, an X-ray diffraction (XRD) pattern, and an examination of the redox chemistry of the as-received Ir-oxide by cyclic voltammetry (CV) needs to be carried out. In this Review, the labeling provided in the corresponding publications for the Ir-oxides will be used (which most commonly is IrO_2_), even though an actual proof of the structure is typically not provided.

Consistent baseline data for the OER activity of Ir-oxide catalysts in alkaline conditions are rare. In a recent study, Anderson et al. suggested baseline control studies and also reviewed published data.^112^ The literature summary suggests mass activities of approximately 11 and 60 A/g in 0.1 M KOH and at a η of 0.35 V for two IrO_2_ powders from two different suppliers. More data is available for 1 M KOH electrolytes, and mass activity data for IrO_2_ powders vary between 9 and 275 A/g at η = 0.35 V, as summarized in Table S6. Stoerzinger et al. carried out a study on single-crystal IrO_2_ (and also RuO_2_) catalysts.^205^ They reported that the (100) surface was intrinsically more active than the thermodynamically more stable (110) surface for both oxides at pH 13 and correlated these OER activities with the density of uncoordinated metal sites of the crystal faces. The results are summarized in Table 4, which also includes intrinsic OER data for commercial IrO_2_ and RuO_2_ powder catalysts reported by Anderson et al.^112^ As seen in Table 4, the RuO_2_ single-crystal surfaces show significantly higher activities over the IrO_2_ equivalents. The activities of these single-crystal IrO_2_ and RuO_2_ catalysts are significantly lower than the intrinsic activities of the commercial IrO_2_ and RuO_2_ particles measured in 1 M KOH (Table 4). The single crystals of IrO_2_ and RuO_2_ used in the study were the rutile forms of the oxides and were formed as thin layers on (001)-oriented SrTiO_3_ and MgO substrates. All of these characteristics, i.e., possible differences in the crystalline catalyst structure, thin layers, and a potential substrate effect may be responsible for some of the intrinsic OER activity differences observed between the catalyst powders and the single-crystal films. The Tafel slope values for the four single-crystal electrodes are consistent with results by Lyons and Floquet,^113^ who reported that IrO_2_ and RuO_2_ catalysts exhibit low- and high-η Tafel slope regions. As shown in Table 4, at lower η values (<0.3 V), the Tafel slopes of all four single-crystal oxide surfaces were similar in the 60 mV/dec range, and an increase to 90 and 140 mV/dec for IrO_2_ and RuO_2_, respectively, was observed for the higher-η (>0.4 V) region. As already discussed in the HER section, a true Tafel slope cannot exceed a value of 120 mV/dec. The 140 mV/dec slopes for the RuO_2_ single crystal may be due to changes in the oxide surface structure taking place at high η values.

**Table 4:** Intrinsic OER Activities for IrO_2_ and RuO_2_ in KOH Electrolytes

catalyst	electrolyte	*j*_int_^a^ (μA/cm^2^)	low-η Tafel slope^a^ (mV/dec)	high-η Tafel slope^a^ (mV/dec)	ref
IrO_2_ (100)^b^	0.1 M KOH	3	55	93	(205)
IrO_2_ (110)^b^	0.1 M KOH	5	61	85	(205)
IrO_2_ powder	1 M KOH	171^c^	n.r.^d^	106	(112)
RuO_2_ (100)^b^	0.1 M KOH	182	54	144	(205)
RuO_2_ (110)^b^	0.1 M KOH	65	56	141	(205)
RuO_2_ powder	1 M KOH	43^c^	n.r.^d^	83	(205)

a The intrinsic activities (*j*_int_) and Tafel slopes are measured at 23.5 °C in the case of the two powder catalysts. The temperature is presumably the same and/or within 3 °C for the single-crystal electrodes. Furthermore, double layer (*C*_dl_) measurements were used to estimate the surface area of the commercial IrO_2_ and RuO_2_ powders. All *j*_int_ and Tafel slopes values shown in Table 4 seem to have been measured from nonsteady-state measurements.

b All of the IrO_2_ and RuO_2_ single crystals were thin films in the rutile form.

c The *j*_int_ values were measured at a η value of 0.35 V.

d n.r. stands for not reported.

**Figure 19:**
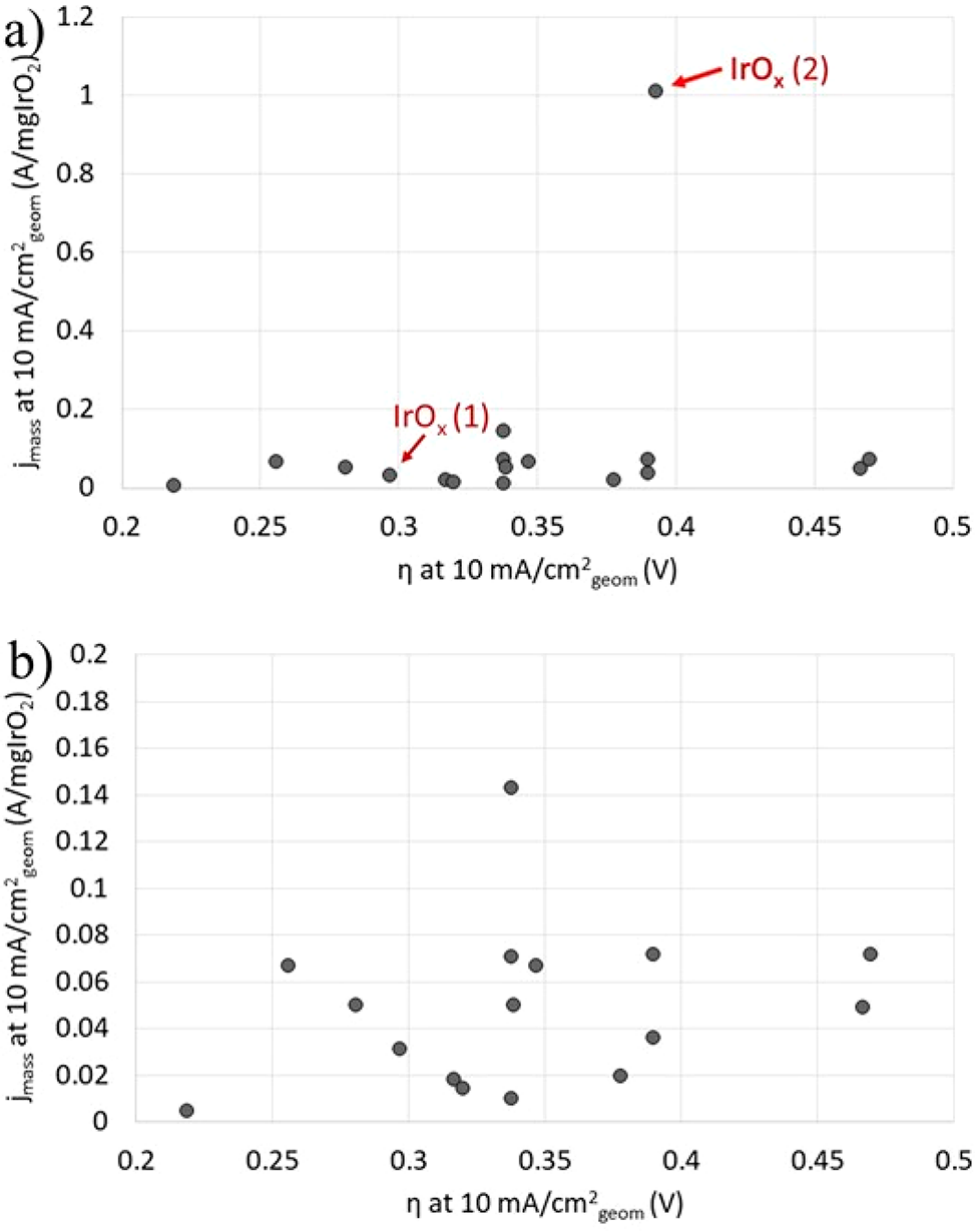
Comparison of mass activities (*j*_mass_) reported for commercial Ir-oxide powder catalysts versus the corresponding overpotential (η). Both *j*_mass_ and the corresponding η values were measured at 10 mA/cm_geom_^2^. (b) Enlarged version of (a) demonstrating the variability in the reported data for the lower η range. Details of the Ir-oxide mass loadings on the electrodes and references are given in Table S6. The majority of the Ir-oxides were reported to be IrO_2_, with the exception of two oxides that are referred to as IrO_*x*_, as indicated in (a).

OER mass activity results reported for Ir-oxide powder catalysts show a large discrepancy, as demonstrated in Figure 19. Figure 19 shows the mass activities and the corresponding η values measured at 10 mA/cm_geom_^2^. Figure 19 also illustrates the sluggishness of the OER, as the η value needed to achieve the same mass current density is substantially higher for the OER catalysts than for the HER catalysts (Figure 10). Furthermore, an exponential dependence of the mass activity on the η value is not evident. In fact, the majority of the catalysts suggest a zero (or very small) slope dependence on η. The exact reasons for this are unknown. It could be partially due to different properties, including differences in the ECSA values, of the Ir-oxide powder catalysts studied from the various suppliers, and it may be partially due to the fact that many of these results are obtained from slow-sweep polarization curves rather than from actual steady-state measurements. Among the data in the 0.35–0.4 V η range, one catalyst shows a substantially higher OER activity (red arrow and IrO_*x*_ (2) in Figure 19a). This catalyst is reported as IrO_*x*_, possibly indicating an amorphous Ir-oxide form.^208^ The OER activity for this IrO_*x*_ catalyst was extracted from steady-state Tafel slope measurements, thus adding validity to the data reported for these measurements of the catalyst. Figure 19a shows the results for one other IrO_*x*_ catalyst [red arrow and IrO_*x*_ (1)] for which the OER activity is seen to lie within the wide scatter of the other Ir-oxide catalysts.

All of these results reveal the necessity of studies establishing baselines for selected catalysts carried out as proper steady-state measurements and in reference to their preparation method and detailed physical and chemical characterization. The lack of true comparative studies including both mass and intrinsic activities for a large number of well-characterized Ir-oxide powder catalysts complicates the identification of the best-performing OER catalysts. Due to the challenges of accurately determining the ECSA values for many of these catalysts, mass activities are often reported and, as for HER catalysts, the mass activity of a catalyst is a characteristic of high practical relevance; however, it cannot be directly translated into an intrinsic activity.

#### 3.3.4 Ni-Based OER Catalysts

High-surface-area nickel and nickel alloy catalysts have been of key interest for traditional alkaline water electrolyzers that runs on high-concentration (in the 30 wt % range) KOH electrolytes, and the catalysts are directly formed on nickel mesh current collectors. Reports for the synthesis methods of unsupported and high-surface-area alloy powder OER catalysts are scarce. In fact, it is challenging to synthesize unsupported alloy powder catalysts with the high surface areas needed for AEMWE OER catalysts. It is noteworthy that Ni alloys have shown promising intrinsic OER activities that can be high specifically when freshly prepared.^209−211^ Similar to the case of HER catalysts, the most promising Ni-alloy OER catalysts seem to be Ni_*x*_Fe_*y*_ and Ni_*x*_Co_*z*_Fe_*y*_, although Ni-based alloys containing, e.g., Al and Mo have also been suggested.^212,213^ Unfortunately, Al and Mo suffer stability issues at high potentials in high pH conditions, a fact that is often neglected. In the cases of the alloy and metal catalysts, it can be assumed that an oxide and/or an (oxy)hydroxide form is involved in the OER, as at least the surface of these catalysts will be transformed into oxidized forms upon exposure to the high potentials and high pH typical for OER conditions.^204,214^

Not surprisingly, Ni-based catalysts in the nanosize range have been synthesized, and some of the highest OER mass activities (measured in thin-layer electrode cells) have been reported for these high-surface-area catalysts. For example, at η = 0.35 V and in 1 M KOH, a mass activity of 1795 A/g_catalyst_ was reported for a 5 nm Ni-shell catalyst formed on an ∼50 nm Fe core particle.^215^ In addition to the high surface area, the combination of a thin Ni shell on a Fe core appears to increase the intrinsic catalytic activity of the Ni. Studies will need to be carried out to understand if the high activity of such a nanosized catalyst will also be observed in an MEA and if nanoparticles and core–shell catalysts possess long-term stability under real AEMWE operating conditions. However, recent reports for single AEMWE cells run on 1 M KOH electrolytes used a commercial NiFe_2_O_4_ OER catalyst. Details of the catalyst are not given, but the supplier, US Research Nanomaterials, focuses on nanosized catalysts. Long-term operation of up to several thousand hours at reasonably high *j* values of 1 A/cm^2^ is shown, suggesting that at least the latter type of Fe-containing catalysts could be suitable for AEMWE applications despite the fact that the Fe-to-Ni ratio of this catalyst is very high (Table 6).

**Figure 20:**
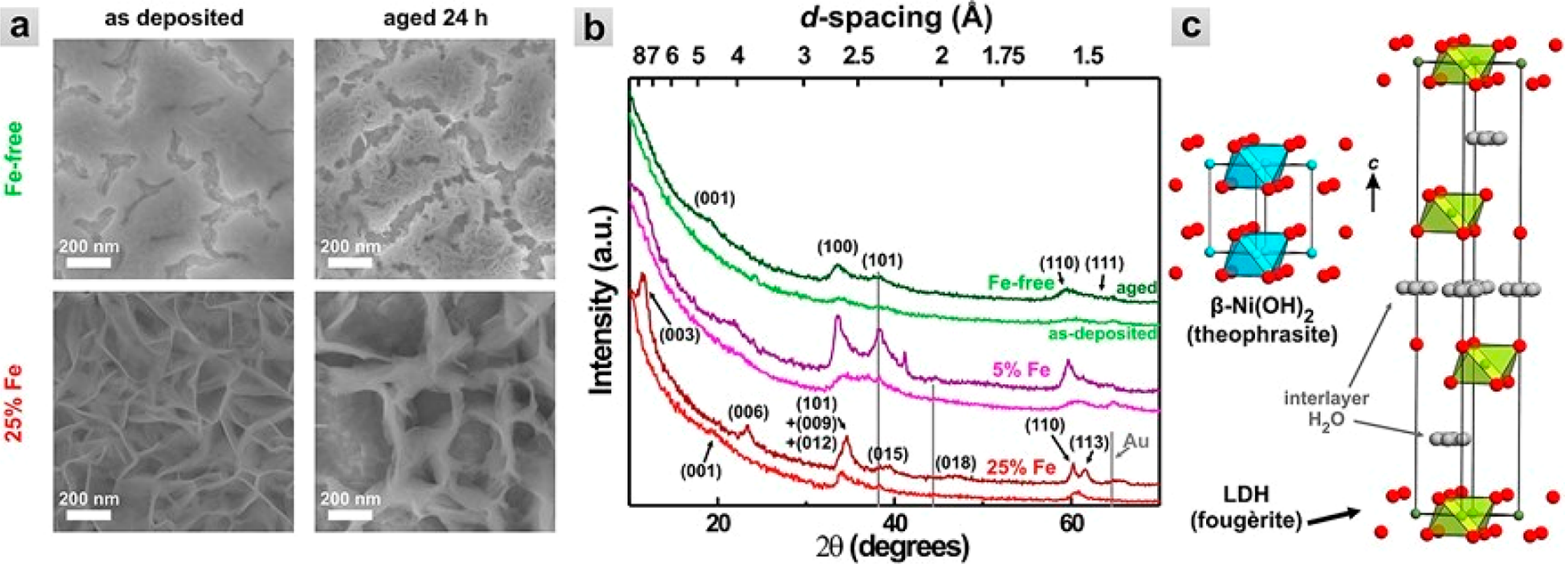
(a) Scanning electron microscopy (SEM) images, (b) XRD patterns, and (c) unit cell structures for Ni(OH)_2_ and NiFeO_*x*_H_*y*_ catalysts. (b) XRD patterns for different amounts of Fe in the NiFeO_*x*_H_*y*_ catalysts. (c) Interlayer of H_2_O in the open LDH structure of the NiFeO_*x*_H_*y*_ catalyst. Reprinted with permission from ref (169). Copyright 2014 American Chemical Society.

Much of the recent OER catalyst development for AEMWEs has focused on the oxy(hydroxide) forms of nickel. The exact nature of these oxy(hydroxides) can vary, and hence, the abbreviation MO_*x*_H_*y*_ is preferably used. It is known that nickel-based catalysts on their own have a low catalytic activity for the OER, while the incorporation of iron into the nickel lattice can substantially enhance the OER activity.^169,216^ The iron appears to play a key role for the observed enhancement and facilitates the formation of high-surface-area structures. Fe atoms easily replace Ni atoms in the oxide/(oxy)hydroxide lattice. In the as-synthesized catalysts, the Fe is present as 3+ and the Ni is present as 2+, thus creating a change in the overall charge, which is compensated by the intercalation of anions such as carbonate and also water molecules, creating layered and high-surface-area structures that can also facilitate ion transport.^217^ The layered double helix (LDH) structures are composed of Ni and Fe layers and can be formed as two-dimensional layered sheet structures. The sheets are often only a few nanometers thick. Figure 20 demonstrates the layered high-surface-area structures, experimental XRD patterns, and corresponding structures.

#### 3.3.5 Iron Contribution to OER Catalysts

**Figure 21:**
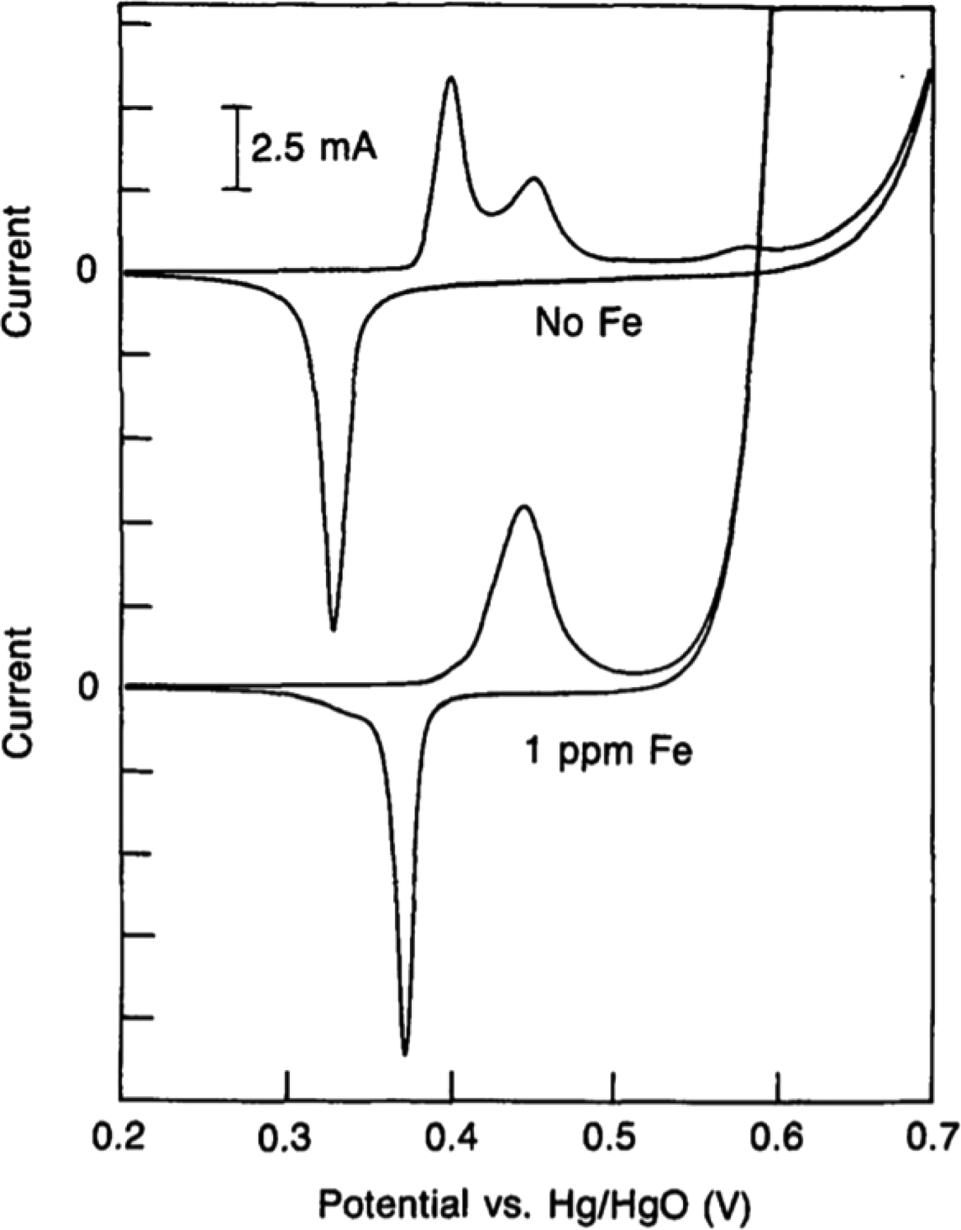
Effect of a 1 ppm Fe impurity in a 25 wt % KOH electrolyte on the cyclic voltammogram (CV) characteristics of a nickel oxide thin-film electrode. The steep increase in current at ∼0.52 V seen for the CV curve containing the Fe impurity (lower graph) is due to the Fe-impurity-facilitated OER. Reprinted with permission from ref (218). Copyright 1987 IOP Publishing.

Many past studies have ignored the fact that, due to the similarity of Fe and Ni (with atomic numbers of 28 and 26, respectively), iron is easily incorporated into the nickel (as well as the Co) lattice. Corrigan reported in 1987 that even trace Fe impurities present in the KOH electrolytes can decrease the Tafel slope and also increase the OER activity of the Ni catalysts, as shown in Figure 21.^218^ Iron is typically also present in nickel precursor salts and KOH electrolytes unless high-purity chemicals are used. Chemical and electrochemical methods have also been proposed for the removal of iron from commercial KOH electrolytes.^219,220^ Fe is easily incorporated into the nickel lattice during the synthesis and/or upon potential cycling when NiOOH is formed unless iron-free chemicals are used. Thus, reports that ignore the contribution of iron cannot be used for reliable catalyst activity data interpretation.

**Figure 22:**
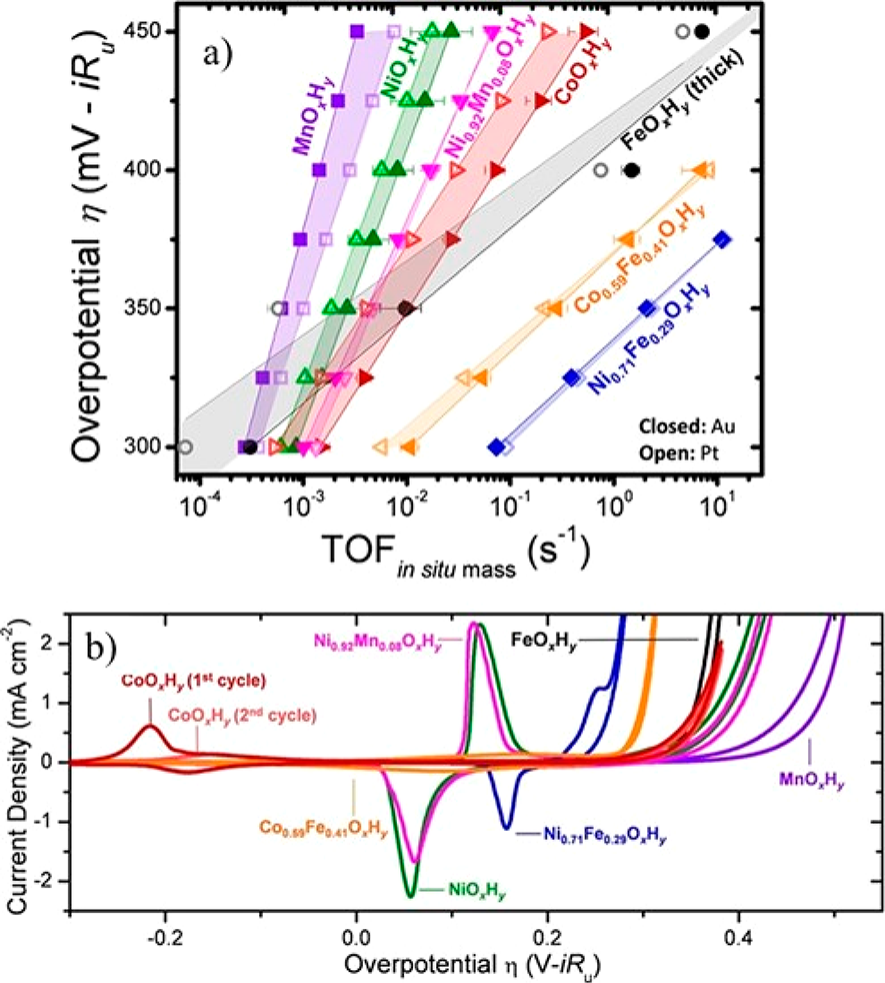
OER activity trends for various thin-film catalysts made using Fe-free precursors and Fe-free 1 M KOH electrolyte solutions. (a) η plotted versus the corresponding TOF number for the catalysts. (b) CV characteristics. The mass of the thin films was used to calculate the TOF number. Reprinted with permission from ref (221). Copyright 2015 American Chemical Society.

In the case of single-metal (oxy)hydroxides, i.e., when Fe impurities are absent, FeO_*x*_H_*y*_ on its own exhibits a higher OER activity than other single-metal (oxy)hydroxides, as follows: FeO_*x*_H_*y*_ > CoO_*x*_H_*y*_ > NiO_*x*_H_*y*_ > MnO_*x*_H_*y*_.^221^ This relatively recent study compared (oxy)hydroxides formed by electrodeposition as thin films of similar and low mass in 1 M Fe-free KOH at η values of 0.45 V and used high-purity, i.e., low Fe content, precursor salts for the synthesis. Therefore, this trend may well reflect a more accurate order for the OER activity of single-metal (oxy)hydroxides than seen in previous studies.^221^Figure 22a shows the trends of the single-metal (oxy)hydroxide catalysts evaluated as TOF calculated per total metal sites of the thin catalyst films, which were assumed to be surface sites, while Figure 22b shows the CV characteristic of the catalyst films. The beneficial effect of Fe incorporation into Ni- and Co-(oxy)hydroxides is also demonstrated in Figure 22, which shows that Ni_0.71_Fe_0.29_O_*x*_H_*y*_ has the highest TOF followed by Co_0.59_Fe_0.41_O_*x*_H_*y*_.

Activity increases of 30–1000 times have been reported due to Fe incorporation in NiO_*x*_H_*y*_ single-metal and multimetal catalysts.^222^ This large range can be assigned to the many different catalyst-synthesis procedures used, which in turn results in catalysts with many different chemical, structural, and physical properties. Dionigi and Strasser presented a review that also includes the many different synthesis routes for these oxides.^223^ Again, a thorough characterization of the as-prepared catalysts is often lacking and requires more attention. Ni_*z*_Fe_*z*–1_O_*x*_H_*y*_-type catalysts have shown high OER mass activities when tested in thin-electrode-layer setups, and recent single AEMWE cell studies are also promising. The long-term stability of iron still needs to be proven, although recent Pourbaix diagram calculations for an iron-doped Ni-NiOOH system suggest that the iron doping improves the pH stability range by ∼2.5 units for both the acidic and alkaline conditions.^224^

**Figure 23:**
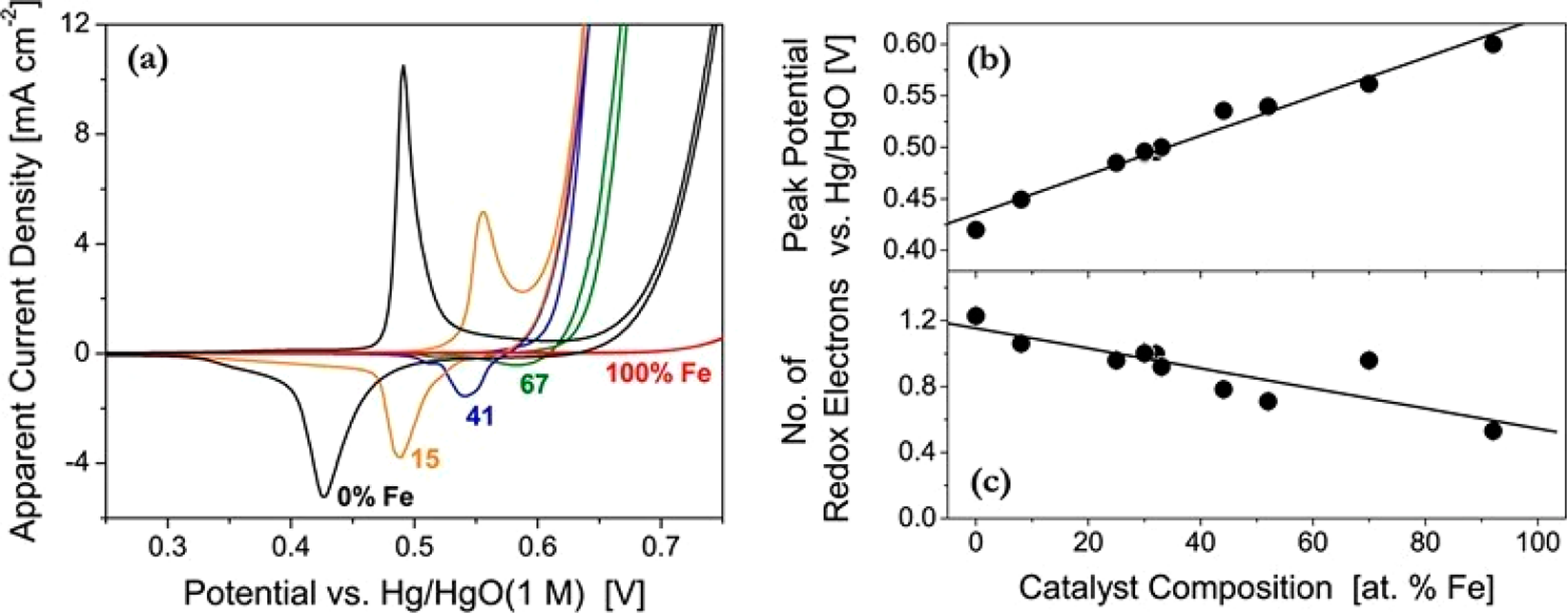
Influence of the atom % of Fe incorporated into NiO_*x*_H_*y*_ films. (a) CV characteristics of the films showing a continued shift of the redox peaks of the Ni^2+^/Ni^3+^ reaction to more-positive potentials, while the onset potential for the OER is shifted to lower values up to 15 atom % Fe, followed by an increase for the higher Fe atom % concentrations. (b) Linear increase of the Ni^2+^/Ni^3+^ redox reaction potentials with increasing atom % Fe, while the number of electrons transferred in the film shows a linear decrease. Reprinted with permission from ref (228). Copyright 2013 American Chemical Society.

An optimum OER activity at 5–10 atom % Fe, which levels off at 30–50 atom % Fe, of total TM mass has been reported for NiO_*x*_H_*y*_ films (Figure 23).^225^ Above the 30–50 atom % range, Fe forms Fe_2_O_3_ and transitions to the unstable FeOOH, which also reduces the catalytic effect of the incorporated Fe. Furthermore, the location of Fe in the structure is key to reaching high catalytic activities. It is suggested that Fe located at the surface and edges and incorporated into defects accounts for most of the activity increase as compared to Fe present in the bulk of the catalyst.^226^ This is also the case for Fe incorporation into Co catalysts.^227^ This explains why small Fe contents can lead to orders of magnitude higher activities. In addition to assisting in the formation of high-surface-area, open, two-dimensional layered structures, Fe can also affect the electronic conductivity of a catalyst. Fe added to the bulk of Ni catalysts appears to impact the redox behavior, which can be seen in a shift of the Ni^2+^/Ni^3+^ reaction to higher potentials.^228^

Single-metal Co catalysts, which can possess good electronic conductivities, have shown decent but not high OER activities.^229^ However, similar to NiO_*x*_H_*y*_, Fe impurities from the electrolyte can incorporate into the Co_*x*_O_*x*_H_*y*_ structure, which can lead to higher OER activities.^230,231^ Unlike the Ni(Fe)O_*x*_H_*y*_ system, the incorporation of Fe into the CoO_*x*_H_*y*_ structure from the electrolyte occurs at a much slower rate. Intentional incorporation of 40–60 atom % Fe into CoO_*x*_H_*y*_ yielded a 100 times increase in catalytic activity compared to Fe-free CoO_*x*_H_*y*_.^222,230^ However, the incorporation of large amounts of Fe can dilute the high electronic conductivity of the cobalt host.^222^ Furthermore, a rapid OER activity decay can be observed in CoFeO_*x*_H_*y*_ catalysts due to the formation of less-conductive Fe oxide phases. The latter is not stable under the alkaline OER conditions seen in Fe leaching from the catalyst.^222^ The high OER activity places CoFeO_*x*_H_*y*_ just behind NiFeO_*x*_H_*y*_ as the most promising bimetallic catalyst.

#### 3.3.6 Ternary M_*n*_M_*m*_O_*x*_H_*y*_ Catalysts

To further tune the intrinsic OER activity, ternary catalysts mostly including the same TM elements as discussed earlier are also explored. NiFeCoO_*x*_H_*y*_ is one of the most promising ternary catalysts. The 3+ oxidation states of both Ni and Co are electronically conductive and need to be reached for the OER (neither the Ni nor Co 2+ oxide states are conductive). The addition of Co to NiFeO_*x*_H_*y*_ increases the electrical conductivity (the electronically conductive Co^3+^ is formed at lower potentials than the Ni^3+^.^232^ Therefore, the addition of Co to Ni-based catalysts facilitates the transition to a more-conductive Ni phase.^208,219,232,233^ As an example, the electronic benefit of the Co in NiCoFeO_*x*_H_*y*_ catalysts has also been reported for thin spin-cased and subsequently annealed films that showed 22 mV/dec lower Tafel slopes (58 versus 77 mV/dec for NiCoFeO_*x*_H_*y*_ versus NiFeO_*x*_H_*y*_).^232^ However, the effect of the Co addition on the intrinsic OER activity was reported to be minimal: the most active NiCoFeO_*x*_H_*y*_ film showed only an ∼1.5 times higher intrinsic activity (measured as TOF per surface atom mass) than the Co-free NiFeO_*x*_H_*y*_ film. The increased electronic conductivity of such catalysts containing Co may have other benefits such as improving the performance of MEAs. Differences in the electronic conductivities of catalysts are often not accounted for and could well affect the performance of the catalyst when incorporated into CLs (section 6).

The positive impact of Fe on the OER activity, at least in the short term, of Ni- and Co-based catalysts has been established, but the question regarding its specific role in the catalyst structure remains unanswered. One popular theory is that Fe is the active site, and it has been suggested that Fe^3+^ acts as a Lewis acid promoting the formation of higher Ni^4+^ oxidation states.^220^ In any case, the LDH structures promote reactant access to active sites due to their open structure.

#### 3.3.7 CoCu-Based Catalysts

CoCu-based catalysts are also of interest.^234^ The use of Co makes a catalyst expensive, but based on the Pourbaix diagrams, both Cu and Co may show the stability needed for OER catalysts for AEMWEs. In fact, single AEMWE cells employing a commercial CuCoO_*x*_ (Acta 3030) anode catalyst were operated for >100 h (Table 6).^84,235^ Copper on its own is a poor OER catalyst, but its incorporation into CoOOH creates a catalyst of higher activity than both Cu(OH)_2_ and CoOOH; however, the mechanism of the enhanced OER activity is not well-understood. Early studies using Cu-incorporated Co_3_O_4_ catalysts (Cu_*x*_Co_3–*x*_O_4_, 0 ≤ *x* < 1) involved single-AEMWE-cell tests and OER catalyst loadings in the 3 mg/cm^2^ MEA area range.^90,236,237^ The substitution of Co into the spinel Co_3_O_4_ lattice shifted both the Co^3+/4+^ redox reaction and the η for the OER to lower potentials. The authors showed promising AEMWE cell performances featuring a cell voltage of 1.8 V at 1 A/cm^2^, and the composition of Cu_0.7_Co_2.3_O_4_ was reported as the most active. A thermal decomposition method was used to prepare the catalysts, and the average catalyst particle size reported was in the 20–30 nm range, although larger particles may be present according to the transmission electron microscopy (TEM) images shown. Alternative synthesis routes may result in the optimization of the catalyst particle size and increases in the ECSA, which in turn could result in higher catalyst and AEMWE cell performances. More recent studies have explored the synthesis of high-surface-area, Cu-substituted Co_3_O_4_ catalysts,^238−240^ Karmakar and Srivastava synthesized Cu_0.3_Co_2.7_O_4_ nanochains.^240^ The smallest catalyst particles were in the 10–26 nm range. Jang et al. used a low-temperature and pH-adjusted coprecipitation method to form Cu_0.5_Co_2.5_O_4_ and Co_3_O_4_ catalysts.^239^ They reported Cu_0.5_Co_2.5_O_4_ particles of 3–4 nm size to be the smallest and most-active OER catalyst. In 1 M KOH, at 10 mA/cm_geom_^2^ and for Cu_0.5_Co_2.5_O_4_ catalyst loadings of 0.5 mg/cm_geom_^2^, a η value of 285 mV was reported. The Tafel slope of 79 mV/dec for this catalyst is also lower than the 98 mV/dec slope measured for the IrO_2_ catalyst, but it is higher than the slopes for Ni_*z*_Fe_*z*–1_O_*x*_H_*y*_ catalysts. The half-cell performance measured for the Cu_0.5_Co_2.5_O_4_ catalyst on a Ni foam current collector yielded a cell voltage of 1.8 V at 1.3 A/cm^2^, and a decline of ∼15 mV was observed in the polarization curve recorded after 2000 h at 10 mA/cm_geom_^2^. However, the catalyst loading on the Ni foam was high, namely, 10 mg/cm^2^, and the current density used for such a stability test is low. Therefore, the improvement over previous AMEWE cell tests seems minor. It is possible that the coprecipitation method used by Jang et al.^239^ also formed some larger particles, as in fact particle-size control without a stabilizer using the coprecipitation method is a challenge. Park et al. used the approach to form nanostructured CuCo_2_O_4_ catalysts directly on the Ni foam current collector.^241^ A cell voltage of 1.8 V was achieved at 1 A/cm^2^, showing a higher performance at a higher current density than that for a commercial IrO_2_ powder catalyst, although the loading of the CuCo_2_O_4_ versus the IrO_2_ catalyst on the Ni foam was significantly higher, namely, 23 versus 4 mg/cm^2^, respectively. The result for Cu_*x*_Co_3–*x*_O_4_, 0 ≤ *x* < 1, makes this system interesting, but methods to form higher-ECSA catalysts need to be found.

#### 3.3.8 Perovskites

Perovskites are another class of materials studied extensively as catalysts for the OER in alkaline media. The general formula of the perovskite structure is ABO_3_, where A and B are cations of different size. Perovskite catalysts are composed of rare and alkaline earth metals at site A and 3d TM at site B. The variation in the OER activity for a perovskite is correlated with the e_g_ orbital filling, indicating an e_g_ closer to unity to be more active.^242^ A decade ago, Ba_0.5_Sr_0.5_Co_0.8_Fe_0.2_O_3−δ_ was reported with an intrinsically high OER activity,^243^ but it is unstable under the oxidative conditions that are relevant for AEMWEs. Further work showed that Ba^2+^ and Sr^2+^ leached, leaving behind a less-active Fe–Co surface.^244^ Recently, a double cubic perovskite, Pr_0.5_Ba_0.5_CoO_3−δ_, with the highest OER activity and an increased stability among perovskites was reported.^242^ According to computational studies, the position of the O p-band center relative to the Fermi level can explain the different OER activities observed for perovskite catalysts.^243,127,245^ Fermi levels closer and overlapping to the O p-band are linked to higher activities. Unfortunately, it is challenging to determine the exact binding energy of M–O as the surface is altered due to leaching and redeposition of metal cations during the OER.

#### 3.3.9 Chalcogenide, Sulfide, and Phosphide Dopants

Research has also focused on chalcogenides, sulfides, and phosphides as dopants for TM catalysts because this class of materials has shown promising HER activities.^246−248^ Many studies report that TM sulfides and phosphides are better OER catalysts than the TM-only equivalent.^246,249−251^ Metal sulfides, phosphides, and nitrides are thermodynamically unstable under oxidizing conditions;^204,252^ hence, it is expected that these catalysts are oxidized to (oxy)hydroxides. However, detailed experimental support is lacking. Researchers have acknowledged the formation of the oxide and hydroxide phases at the surface, leaving the core, if anything, as sulfides, phosphides, and chalcogenides.^253,254^ The nature of the resulting structure may well have an enhanced catalytic activity due to the creation of defect sites or a higher surface area. In operando and postmortem analyses are needed to elude the mechanism.

#### 3.3.10 Catalyst OER Activities in a Thin Layer Versus a Single AEMWE Cell

Measuring catalytic activities in a thin-layer electrode setup typically represent short-term measurements. Therefore, the subsequent evaluation of promising catalysts in single AEMWE cells or a half-cell setup are needed. The importance of this is also highlighted by the substantially higher Tafel slopes that have been measured in single-cell AEMWEs compared to slopes determined from thin-layer setups. Slopes higher than 120 mV/dec have been observed in AEMWEs, indicating that other factors than catalytic reactions determine this slope.^255^

**Figure 24:**
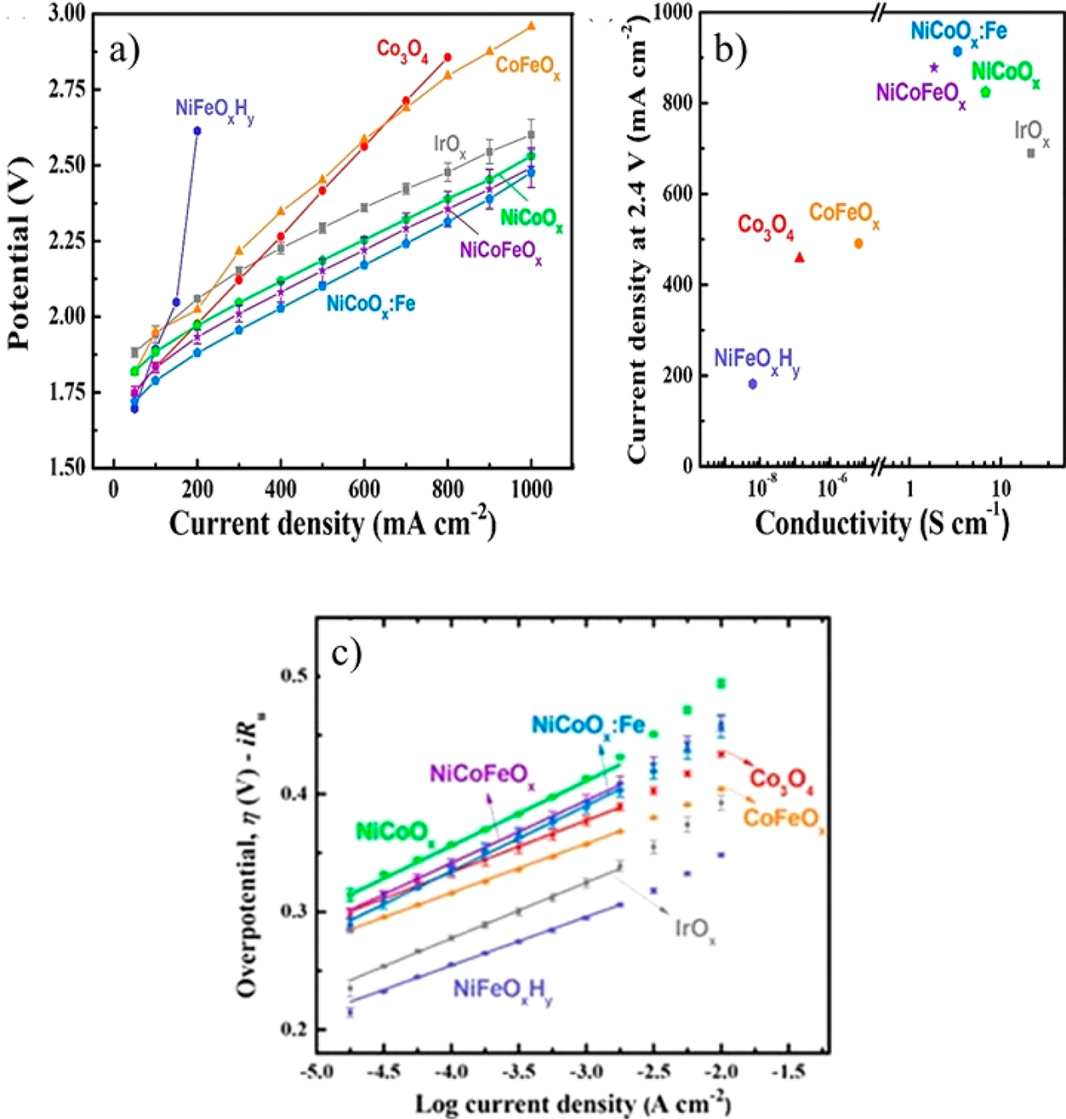
(a) Tafel slopes measured in a thin-catalyst-layer setup, (b) conductivity of the as-prepared powders, and (c) performance of the OER catalysts in a single-cell AEMWE for a range of OER catalyst powders. The Tafel slopes in (a) were extracted from steady-state measurements in 1 M KOH electrolytes at 20 °C. The AEMWE performances were measured under a pure water feed at 50 °C. Reprinted with permission from ref (208). Copyright 2019 American Chemical Society.

Xu et al.^208^ made a series of single-, bi-, and multi-metal oxide OER catalysts. Catalysts showing higher thin-layer and single-cell AEMWE performances have been reported, but their study represented the measurement of both the OER activities for thin-layer electrodes and their subsequent evaluation in single-cell AEMWE performances for a large number of catalysts. Mixed Ni-, Co-, and Fe-oxide catalysts (namely, Co_3_O_4_, CoFeO_*x*_, NiFeO_*x*_H_*y*_, NiCoO_*x*_, NiCoO_*x*_:Fe, and NiCoFeO_*x*_) were made and also compared to a commercial IrO_*x*_ (Proton OnSite) catalyst. Fe was likely incorporated into the Co_3_O_4_ and NiCoO_*x*_ catalysts from the KOH electrolyte, and the authors used the formula “IrO_*x*_” for the commercial catalyst, possibly reflecting an amorphous Ir-oxide, as already discussed in section 3.3.3. It can be seen (Figure 24) that their catalysts referred to as NiCoO_*x*_:Fe, NiCoFeO_*x*_, and NiCoO_*x*_ outperformed the commercial IrO_*x*_ catalyst in the AEMWE cell, while the OER activity in a thin-catalyst-layer setup showed a different order. The mass activity (in A/mg_cat_) measured for the thin catalyst layers at a η value of 0.35 V showed the following order: NiFeO_*x*_H_*y*_ (633 A/mg) > IrO_*x*_ (273 A/mg) > CoFeO_*x*_ (61 A/mg) > Co_3_O_4_ (30 A/mg) > NiCoFeO_*x*_ (18 A/mg) ≈ NiCoO_*x*_:Fe (17.2 A/mg) > NiCoO_*x*_ (8.8 A/mg). The order of the Tafel slopes measured in the thin-catalyst-layer setup was relatively similar to the order of mass activity as the lowest slopes in the 40–49 mV/dec range were observed for NiFeO_*x*_H_*y*_, CoFeO_*x*_, and Co_3_O_4_ as well as 47 mV/dec for IrO_*x*_, while NiCoFeO_*x*_, NiCoO_*x*_:Fe, and NiCoO_*x*_ showed slopes of 54, 55, and 53 mV/dec, respectively. The authors suggested that some of the discrepancy between the thin-catalyst-layer setup data and the single-AEMWE-cell evaluation was due to the differences in electrical conductivity of the powder catalysts, as NiFeO_*x*_H_*y*_, Co_3_O_4_, and CoFeO_*x*_ showed lower electronic conductivities than the other catalysts. Some of it may also reflect stability issues of the catalysts such as the NiFeO_*x*_H*y* catalyst, which seemed to show that one of the highest OER mass activities reported in a thin-catalyst-layer setup contained a high amount of Fe. It is possible that some of the high OER activity observed in the thin-layer and short-term experiment originated from a single Fe-oxide phase. Dissolution of Fe from a single Fe-oxide phase may well occur. The overall results clearly indicate that measurements in single AEMWE cells are needed to confirm the performance of a catalyst.

#### 3.3.11 Role of Lattice Oxygen for OER Catalysts

**Figure 25:**
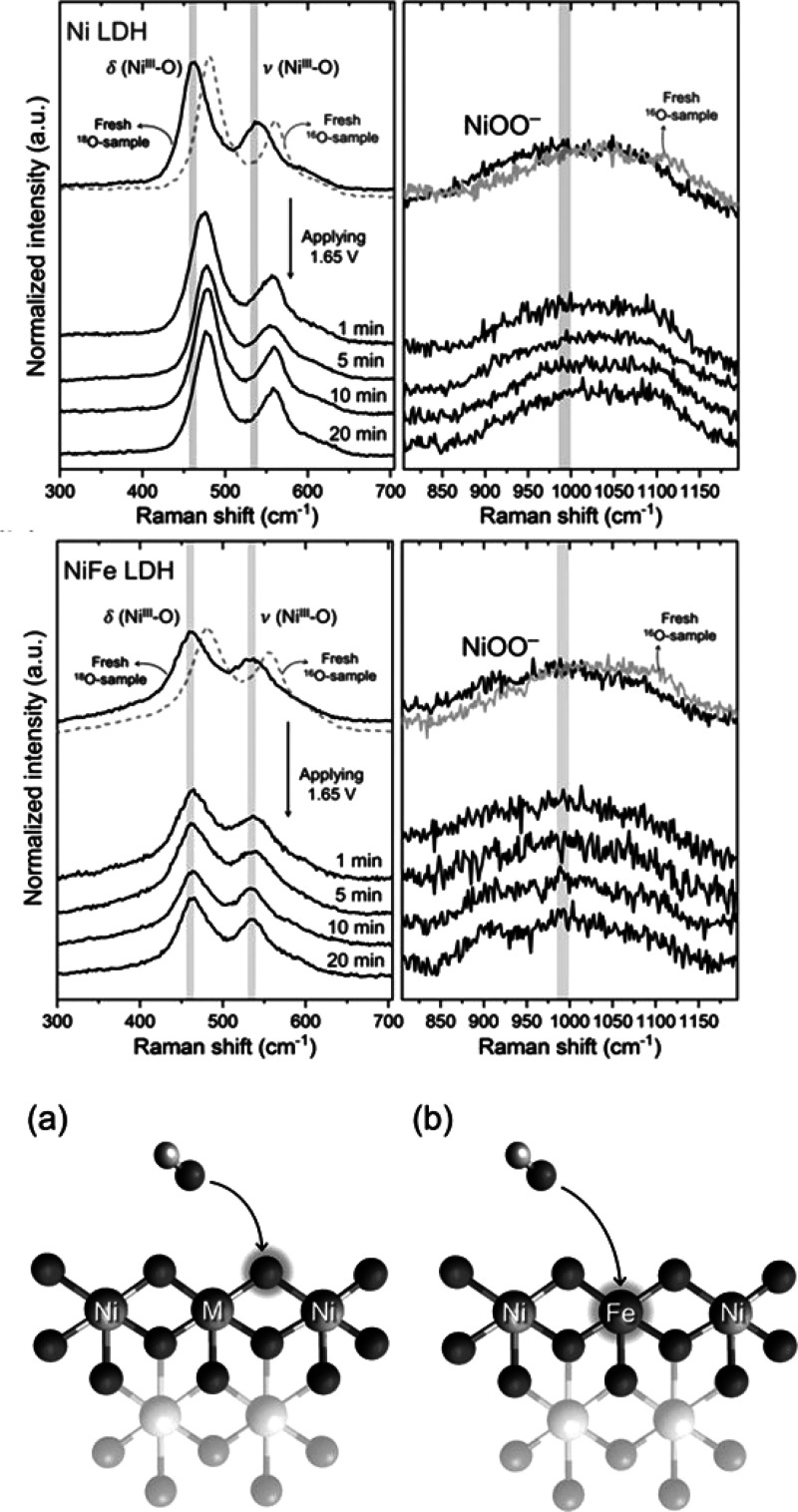
Isotope-exchange experiments and in situ Raman spectra of ^18^O-labeled (top) Ni and (middle) NiFe LDH, indicating the frequency shift and contribution of oxygen lattice for Ni, while the frequency remains constant for NiFe LDH. The figures at the bottom show the suggested scheme for O_2_ involvement (a) with and (b) without Fe. Reprinted with permission from ref (266). Copyright 2019 Wiley.

The high potentials driving the OER and the presence of lattice oxygen in most OER catalysts raise the question of what role lattice oxygen plays in the reaction and also in the stability of the catalysts. DFT calculations suggest that the release of lattice (bulk) oxygen in a system such as NiFeO_*x*_H_*y*_ is feasible because the energetics for the OER at the surface and in the bulk are comparable.^256^ One study reports a linear increase of the OER current with increasing NiFeO_*x*_H_*y*_ loadings (up to 0.1 mg/cm^2^) on a flat electrode surface.^257^ This was taken as support for activity from lattice oxygen, while others claim the opposite.^258^ Isotope-labeled water experiments coupled to a high-sensitivity detection method suggest that lattice oxygen contributes to the OER for many catalysts, including Au surface oxides,^259^ IrO_2_/Ti,^260^ Co_3_O_4_ spinel,^261^ some Ru-based catalysts,^262,263^ and perovskites including La_0:5_Sr_0:5_CoO_3−γ_, Pr_0:5_Ba_0:5_CoO_3−γ_, and SrCoO_3−γ_.^264^ Release of lattice oxygen was not observed for crystalline RuO_2_ structures^265^ nor for perovskites with low metal–oxygen bonds.^264^Figure 25 shows an example of isotope-labeling and Raman spectroscopy experiments for Ni and NiFe LDH catalysts suggesting the contribution of lattice oxygen in the case of Ni but not for the NiFe LDH catalyst.

The literature results show that bulk oxidation is observed for specific compositions and catalyst structures. The lattice oxygen activities seem to vary depending on the nature of the catalyst, crystallinity, and operating conditions, as observed for the case of NiFeO_*x*_H_*y*_ nanoparticles.^214,266^ Establishing an understanding of the factors determining the role of lattice oxygen will help in tuning the morphology, structure, and composition of the catalysts.

### 3.4 Stability of HER and OER Catalysts

As discussed throughout the catalyst section, HER and OER catalyst stability measurements relevant to AEMWE operating conditions are needed. Many different types of electrochemical methods such as chronoamperometric, chronopotentiometric, potential steps, and/or potential cycling have been applied to probe the stability of HER and OER catalysts. Therefore, consistent measurement procedures are needed, and suggested stability-evaluation protocols are shown in the Supporting Information.

Relevant factors that are potentially responsible for the deactivation of a catalyst, such as surface poisoning, morphology changes, and metal dissolution, can be missed by applying only electrochemical techniques. Ideally, the electrochemical catalyst stability studies are coupled in situ with analytical techniques, which are capable of quantifying dissolved metal components of the catalyst such as inductively coupled plasma mass spectrometry/optical emission spectroscopy (ICP-MS/OES).^267^ Electrochemical quartz crystal microbalance measurements (EQCM) can be useful to study mass changes of the electrode in situ.^268^ However, the EQCM response needs to be linear in order to avoid misinterpretations, as discussed by Moysiadou and Hu.^269^ Effort is being devoted to develop in situ techniques, such as in situ SEM/TEM or XRD, but these are far from being able to work under real operating conditions and are more suitable for the study of model catalysts. Therefore, the coupling of the electrochemical measurements with analytical techniques such as ICP-MS/OES is currently preferred.

The detection limits of ICP-MS are low [as low as 10 parts per trillion (ppt)], and hence, ICP-MS works best for solutions of low metal concentrations. ICP-MS is best-suited for acidic electrolytes,^270^ while the high cation concentration of alkaline electrolytes introduces calibration issues. The ICP-MS calibration becomes very challenging for cation concentrations exceeding 0.05 M,^271^ and ICP-OES, which is a less-sensitive instrument, is better suited for alkaline electrolytes. In the case of alkaline electrolytes, specific care needs to be taken to ensure complete metal dissolution in order to produce reliable ICP results.^272^ The additional step of acidifying the electrolyte is needed, hence presenting a challenge to in situ electrochemical–ICP measurements involving alkaline electrolytes. Some examples of catalyst-stability measurements, which included ICP-MS/OES measurements, were carried out for Co_2_P HER catalysts.^273^ Preferential dissolution of P over Co was shown to take place upon potential cycling, leaving a Co-rich surface of approximately twice the ESCA area after 2000 potential cycles. The dissolution of P resulted in an ECSA that doubled upon 2000 CV cycles.

**Figure 26:**
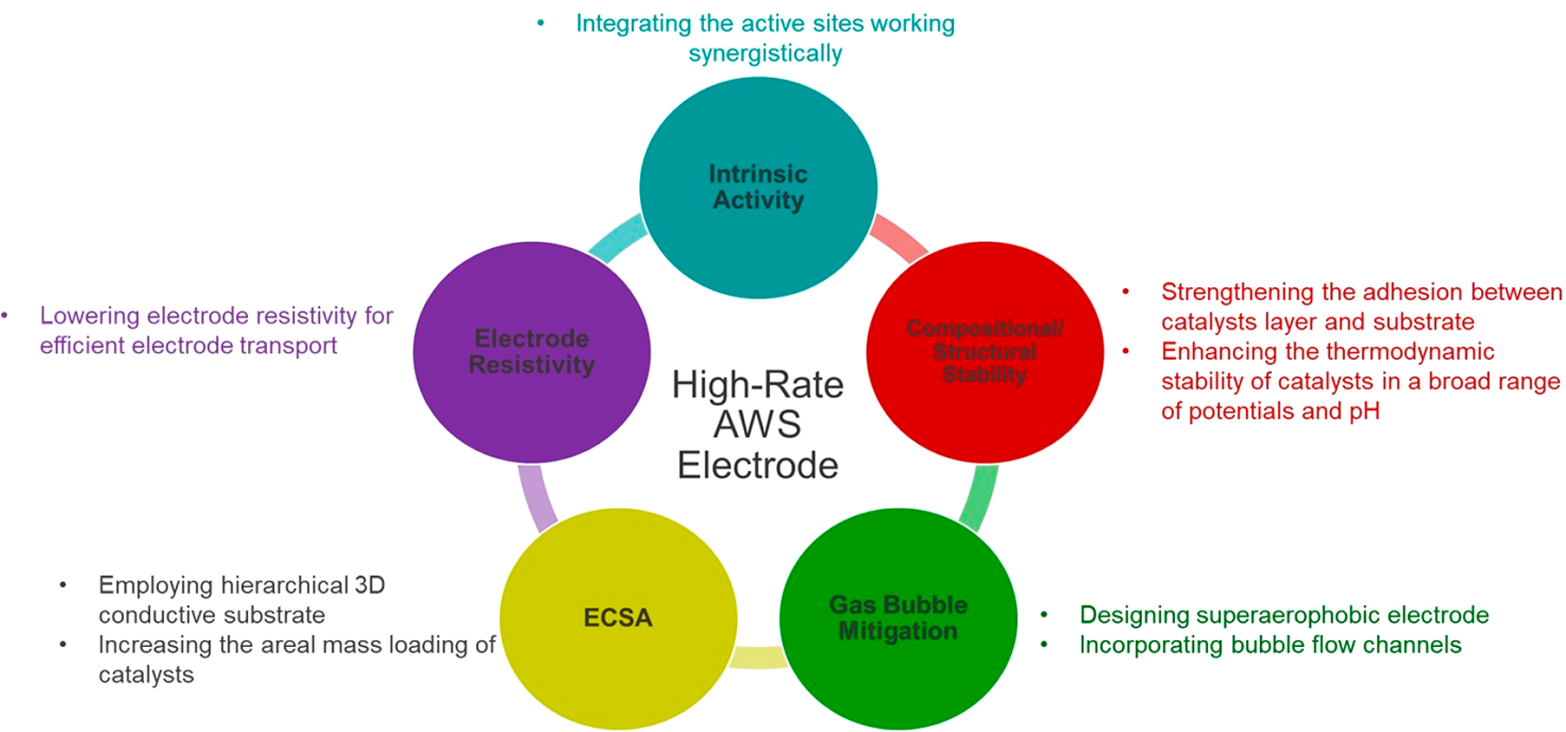
Schematic diagram summarizing the important factors in designing an electrode for high-rate water splitting. Reprinted with permission from ref (274). Copyright 2021 American Chemical Society.

The design of the electrochemical stability measurements reflecting conditions relevant to AEMWE operation is challenging. Many parameters such as the intrinsic activity, structure, composition, ECSA, conductivity of the catalyst, and feed solution of the AEMWE need to be considered for the design of the catalyst-stability experiments, as summarized in Figure 26 The formation of H_2_ and O_2_ gas bubbles is also a concern because they can block catalyst sites and consequently influence the stability measurements. In addition to physically blocking access to active sites, gas bubbles, if trapped in the structure of the catalyst and/or in a CL, can result in structural damage upon violent release.^274^ Gas bubble trapping on an electrode surface is specifically pronounced when a horizontal electrode, like a classic rotating ring disc electrode (RDE), is used and made worse upon rotating the electrode, as gas will be pulled into the center of the electrode by the rotational forces. A challenge is the time scale for the stability experiments, and catalysts capable of delivering high currents (>1 A/mg) for several thousands of hours are needed. It is evident that the design of accelerated stability tests (which reflect relevant AEMWE operating conditions and include information such as intermittent, startup, and shutdown operation and consider the development of potential hot spots and local pH fluctuations in a CL layer) is not trivial.

The electrochemical-stability measurements need to be complemented with a thorough characterization of the catalysts’ composition and structure (using XRD and XPS) at least before and after the measurement. Furthermore, electrochemical characterization of the electrode before and after the stability experiments is needed to show data such as CVs reflecting potential changes in the redox characteristics and ECSA value of the catalyst.

#### 3.4.1 HER and OER Catalyst Stability

The stability of the OER catalysts is generally of greater concern than that for the HER catalysts. However, HER catalysts also undergo activity changes during electrolysis and even during intermittent periods. These activity changes can be caused by decomposition of the catalyst, hydride formation (section 3.1), agglomeration of nanosized catalyst particles, adsorption of the ionomer (section 6), deposition of dissolved metal cations on the catalysts, and H_2_ gas blockage of catalyst sites.

Reasons for catalyst-activity loss during the OER can be manifold and as simple as resulting from physical loss of the catalyst particles. However, chemical and structural alterations of the catalysts can also take place during the OER, such as dissolution of catalyst components and structural and compositional changes, which can enhance or decrease the OER activity. Generally, an increase in the anode potential within the OER region leads to an exponential increase in metal dissolution, which is taken as confirmation that the OER and catalyst dissolution are correlated.^271^ Some key points can also be taken from the numerous OER catalyst studies carried out for dominantly Ir- and Ru-based catalysts in acidic electrolytes.^116^ For example, the formation of an insulating oxide layer favors stability. Also, the metal-dissolution rate for crystalline structures is generally lower than that for amorphous structures due to a stronger metal-to-metal bonding energy. However, a stability test may suggest that a catalyst is very stable, but the catalyst’s OER activity may be inferior.^271^

**Figure 27:**
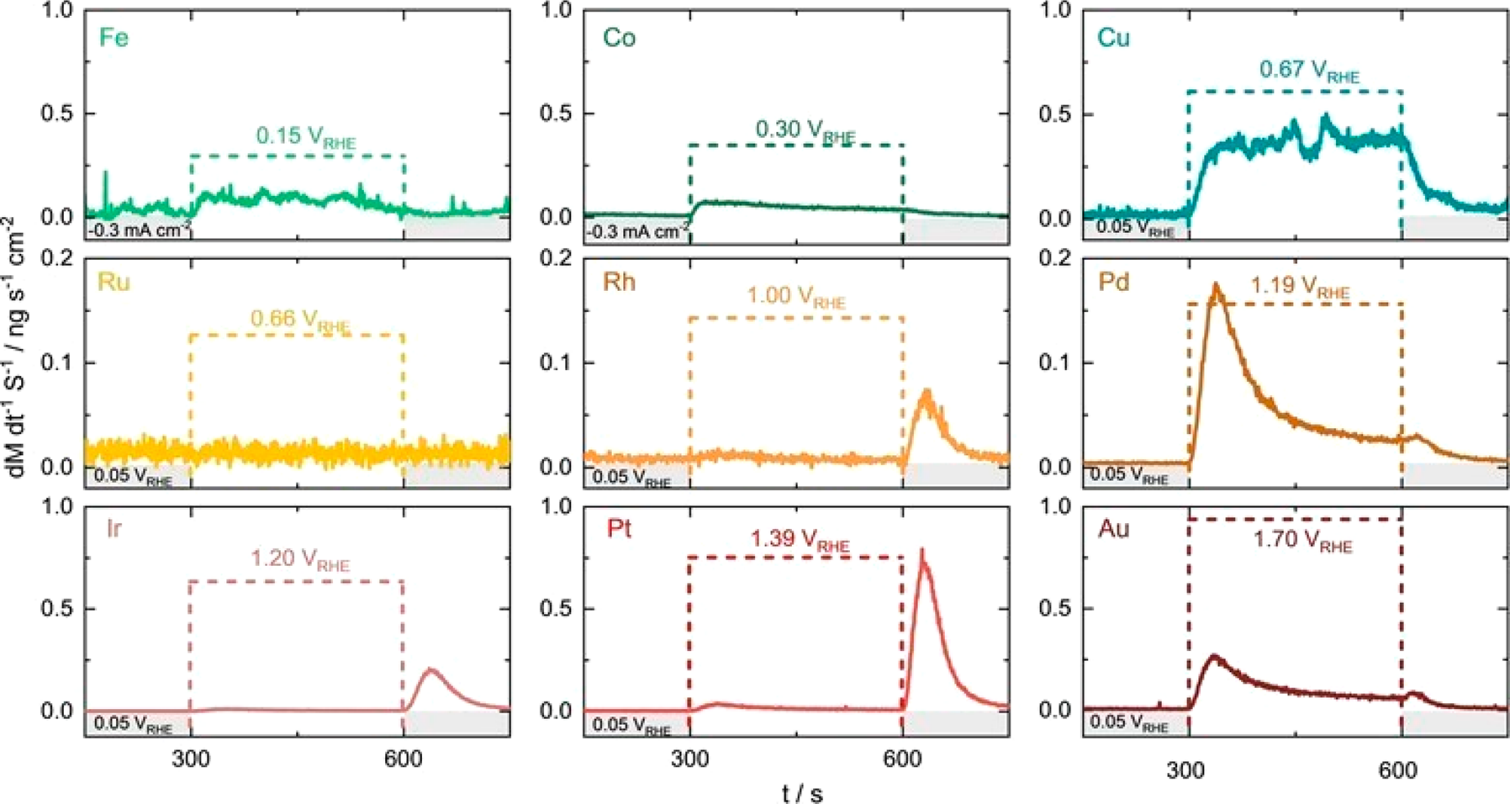
Dissolution rates during transient measurements for nine different metal electrodes (as indicated in the graphs). The metals are grouped according to their electronic structure, i.e., either 3d, 4d, or 5d. Three metals per group were selected. The studies were carried out in 0.05 M NaOH. Reprinted with permission from ref (275). Copyright 2021 Wiley.

Anodic dissolution is the most-probable cause of metal dissolution during the OER and is typically viewed as a continuous process, but catalysts can show instabilities during startup and shutdown periods, as demonstrated in a recent study by Speck et al.^275^ Metal dissolution of nine electrodes that were either 3d, 4d, or 5d transition metals was quantified (in acidic and alkaline electrolytes) using a flow cell and ICP-MS analysis. Either a low potential or a negative current was initially applied with the intent to reduce the metal surface. This was followed by a step to a potential where metal oxide formation was expected to occur, prior to reducing the metal surface again. The metal-dissolution rate was determined for the oxide formation and for the transient conditions, i.e., when the metal oxide surfaces were reduced. Results are shown in Figure 27. The study suggests that the rate of metal dissolution under oxide-formation conditions is proportional to the d-shell of the TM electrodes. For example, for Ir and Au metals that are within the same d-shell structure, metal dissolution is more pronounced for Au Ir. Transient dissolution was not observed for the 3d TMs, while transient metal dissolution occurred for 4d and 5d TMs at different rates. Overall, the results suggest that startup and shutdown should be avoided to decrease cathodic transient corrosion.

The study also suggested that the bonding energy of the metal–metal atoms and the affinity of the metal for oxygen are two determining descriptors of the catalyst’s stability during the OER. It was suggested that metals with higher cohesive energy (or metal–metal bonding energy) are less prone to dissolution, although this statement will need to be viewed in the context of the presence and nature of the electrolye. Additionally, a high oxygen-adsorption energy also favors the dissolution of metals with a higher affinity for oxygen because they are prone to incorporation of oxygen under oxidation conditions. Unlike the case of HER catalysts, the dissolution rate of OER catalysts in an open circuit has not been studied, but it would be useful for the selection of OER catalysts for AEMWEs.

**Figure 28:**
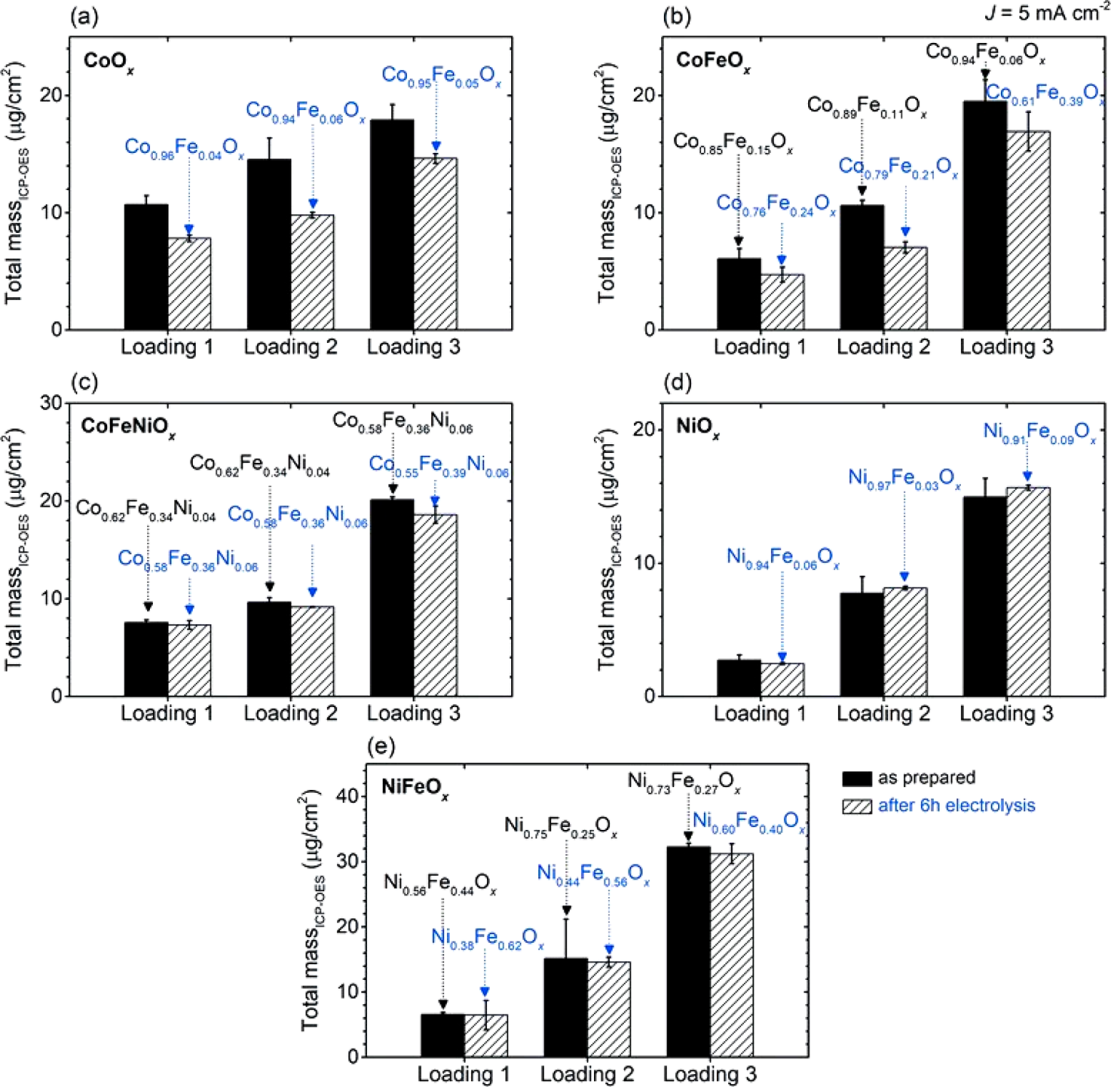
Changes in the masses of various thin-film catalysts before and after constant-current experiments at 5 mA/cm^2^ for 6 h in 1 M KOH. Different loadings of the catalyst were used, as indicated in the figure (loadings 1, 2, and 3). The names of the catalysts and the catalyst compositions (measured before and after 6 h of chronopotentiometric experiments) are also shown in the graphs.^269^

Another effect of metal dissolution is the change in catalyst composition causing changes in catalytic activity. A study focusing on CoO_*x*_, CoFeO_*x*_, CoFeNiO_*x*_, and NiO_*x*_ catalysts analyzed the total mass and composition of TM oxide catalysts before and after chronopotentiometric measurements in 1 M KOH for 6 h, as shown in Figure 28.^269^ All catalysts underwent noticeable compositional changes during the 6 h chronopotentiometric experiment (Figure 28). The first notable change was the incorporation of Fe if Fe was not already present in the catalyst structure [CoO_*x*_ (a) and NiO_*x*_ (c)]. Furthermore, the two catalysts without Co, namely, FeNiO_*x*_ (c) and NiO_*x*_ (d) showed minimal metal dissolution during the initial 6 h, while the Co-containing catalysts [CoO_*x*_, CoFeO_*x*_, and CoFeNiO_*x*_ (a–c)] showed mass losses in the 10–20% range. These results suggest that Co, at least in the catalyst used by this research group, induces a stability issue. Using electrochemical impedance spectroscopy (EIS), the authors determined that the dissolution rate varied over the initial 6 h, and afterward, the catalysts were considered stable. This time-dependent behavior may be due to the dissolution of very reactive catalyst components including defects and undercoordinated surface sites, followed by the establishment of an apparent stable state such as a protective oxide layer.^276^

Theoretical and experimental studies suggest that low-coordinated sites such as defects, edges, and steps are more likely corrosion sites.^276^ However, this descriptor is extremely difficult to quantify and replicate.

#### 3.4.2 Differences between Model Electrochemical Stability Studies Versus an AEMWE

**Figure 29:**
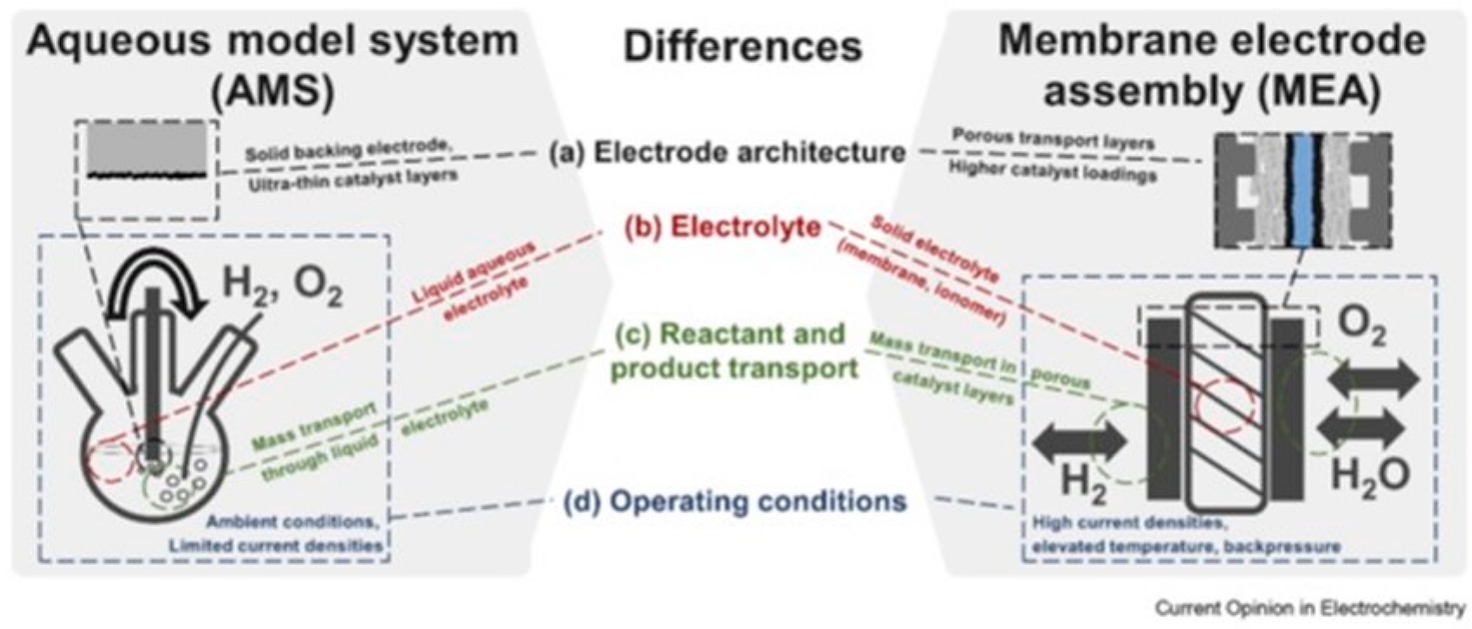
Differences a catalyst can experience in a traditional electrochemical experiment labeled as an aqueous model system (AMS) and in an MEA of an AEMWE cell. The differences can be the electrode architecture, the electrolyte, reactant and product transport, and the operating conditions. Reprinted with permission from ref (277). Copyright 2021 Elsevier.

Many stability measurements utilize traditional electrochemical cells such as an H-cell or a flow cell, where the catalyst is often immersed in an aqueous electrolyte. However, a catalyst experiences significantly different conditions in a thin-layer setup (which is typically an aqueous model system) than in an MEA of an operating AEMWE. A comparison between an aqueous model system and an MEA is presented in Figure 29. One of the main differences is the electrolyte/electrode interface. In a model system, the electrode is completely immersed in the electrolyte, while in an AEMWE, the catalyst layer experiences a higher exposure to the gaseous reactant environment. In an MEA, the catalyst is also surrounded with other components such as an ionomer enabling OH^–^ transport between anode and cathode (section 6). The electrode architecture in an aqueous model system can involve a powder catalyst but also compact thin films preferably deposited onto stable electrode surfaces of low HER and/or OER activity.

To close the gap between model studies and real operating systems, the use of a half-cell gas diffusion electrode (GDE) coupled to an ICP-MS/OES is favored.^278^ Researchers have studied Pt during the HER in a half-cell GDE and acidic conditions, and some findings apply to alkaline conditions. It was found that, contrary to what was observed for model systems, the dissolution of the Pt metal increased when the overall metal loading was decreased. Also, the metal dissolution in a half-cell GDE was lower because of the limited interactions with the electrolyte and the presence of the membrane. The ionomer also plays a role.^278^ For mass-transport-limitation reasons, one could also expect Ostwald ripening and local redeposition on existing particles to become more likely.^279^ Overall, one may speculate that the most general trends observed in a traditional electrochemical setup translate into a half-cell GDE system or an MEA. However, the intrinsic catalyst activities are expected to be different for evaluations carried out for a model versus a half-cell GDE system or an MEA due to the drastically different system architecture and operating conditions.

## 4 Anion-Exchange Membranes

So far, comparably low ionic conductivity and low durability of AEMs have been the major obstacles for the large-scale introduction of AEMWEs. However, recent advances, in increasing both the OH^–^ conductivity and the alkaline stability of AEMs, have fueled AEMWE development.^83,280−282^ Much of the research over the past decade has focused on developing AEMs for alkaline anion-exchange membrane fuel cells (AEMFCs); hence, in this section, reference to AEM performance in AEMFCs is made when adequate. AEMs are made from anion-exchange polymers (AEPs) consisting of cationic headgroups attached to the polymeric backbones. Extensive research is being carried out on these AEPs and many reviews exist, but a performance comparison is difficult due to the lack of consistent evaluation conditions. Therefore, there is a need to advance the understanding of the general performance-relevant parameters in an AEMWE cell. Critical characteristics of AEMs, such as the ion-exchange capacity (IEC), OH^–^ conductivity, chemical and mechanical stabilities, water uptake, and swelling of AEMs, depend on numerous factors including the AEP structure and operational parameters such as the electrolyte and humidification. A high ionic OH^–^ conductivity exceeding 0.1 S/cm is preferred.^32^ High chemical and mechanical stabilities at *j* >3 A/cm^2^ and *T* > 60 °C, in the presence of O_2_ and in alkaline conditions, are needed. Many AEMs break down at temperatures exceeding 60 °C. The early research view was that cationic headgroups are accountable for the IEC, ionic conductivity, and chemical stability, while the polymer backbones are responsible for the mechanical and thermal stability.^40^ It is now recognized that both the mechanism and the rate of degradation are influenced by the complete structure of the AEP, thus calling for studies of the entire polymer rather than individual headgroups and backbones. Previous reviews identified many possible degradation pathways in alkaline media:^40,283,284^ (1) nucleophilic substitution (S_N_2) benzyl substitution, (2) S_N_2 methyl substitution, (3) β-elimination substitution, (4) ylide intermediated rearrangements, (5) S_N_2 Ar aryl ether cleavage in the polymer backbone, (6) ring opening (e.g., imidazolium (IM)), (7) S_N_2 methyl substitution IM, (8) heterocycle deprotonation IM, (9) S_N_2 and ring opening (e.g., in piperidinium, pyrrolidinium, and morpholinium), (10) ring opening (N-spirocyclic ammonium), (11) dehydrofluorination (polymer backbone), (12) nucleophilic addition and displacement (pyridinium), and (13) nucleophilic degradation (guanidinium). Most quaternary amines (QAs) and IM groups are prone to degradation under alkaline conditions via the Hofmann degradation, S_N_2, or ring-opening reaction, especially at elevated temperatures and high-pH conditions. Recent research has also shown that the degradation pathways depend on the AEP structure and the test conditions. For example, β-elimination has been shown to take place for AEMs tested at 80 °C for NaOH concentrations below 4 M, while the methyl substitution reaction is found to be predominant at 120 °C for NaOH concentrations of 8 M and at 100 °C and a low relative humidity (RH) of 5%.^285^

### 4.1 AEM Structures and Their Impact on Alkaline Stability

#### 4.1.1 Cationic Headgroups

**Figure 30:**
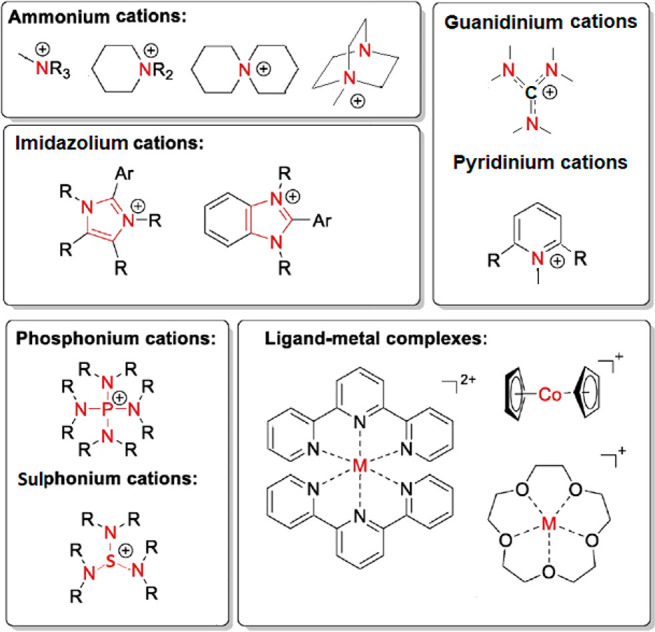
Examples of different types of common alkaline-stable cations for AEMs. Adapted with modification from ref (36).

The cationic headgroups provide the exchange sites for OH^–^ and are described by the IEC value. Many headgroups contain nitrogen, e.g., quaternary ammonium/tertiary diamines,^286−288^ (benz)imidazolium, guanidinium, and pyridinium.^289−292^ QAs are the most popular headgroups due to their promising ionic conductivity in an AEM, comparatively high stability, and ease of synthesis. Some nitrogen-free cationic headgroups have also shown promise with regards to ionic conductivity values and stability, e.g., sterically shielded phosphonium and sulphonium headgroups^293−296^ and ligand–metal complexes (Figure 30).^297,298^

**Figure 31:**
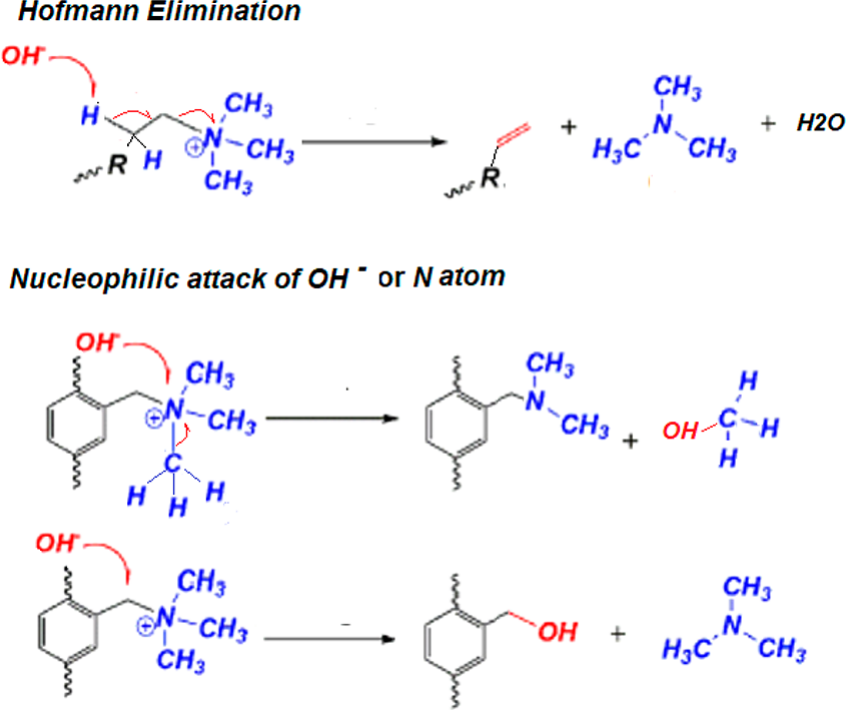
Hofmann elimination and nucleophilic degradation occurring via ammonium group displacement.

To improve the stability of cation groups, many researchers designed molecules that are devoid of β-hydrogen or have a minimal number of β-hydrogen to suppress preferential Hofmann elimination.^299^ However, AEMs without β-hydrogens still show degradation due to other mechanisms, e.g., the S_N_2 mechanism, which occurs via direct nucleophilic attack of OH^–^ anions on nitrogen atoms in the ammonium group, resulting in alcohol departure, or on the carbon atoms bonded with it, resulting in amine byproducts (Figure 31).^300^ Marino and Kreuer^301^ carried out an ex situ stability study on an extensive number of QA headgroups in their salt form using the same testing conditions, including controlling factors such as the temperature, solvent, and degree of solvation. The study reported that β-protons were less susceptible to nucleophilic attacks than previously suggested, whereas the presence of benzyl groups, nearby heteroatoms, or other electron-withdrawing species significantly promoted the degradation.^301^

Further approaches to increase the alkaline stability of cation headgroups are (i) to introduce groups that enable a steric hindrance and, hence, shield the AEP from OH^–^ attacks or (ii) to introduce groups with an electron-donor effect on or near the cations, countering the fact that electron-deficient cationic moieties appended to polymer backbones in AEMs are the most susceptible sites to OH^–^ attacks.

**Figure 32:**
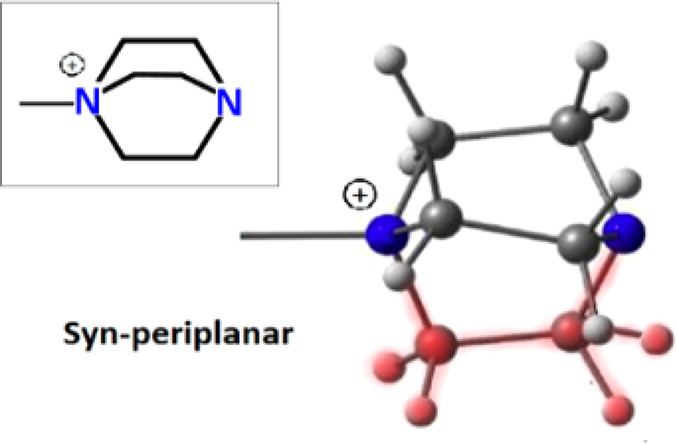
Conformational analysis of DABCO (one of the syn-periplanar structures is highlighted in red).

A simplified stability trend for common cations used in AEMs has been proposed: pentasubstituted IM > C_2-_aryl benzimidazolium > simple IM; N-spirocyclic piperidinium > piperidinium > pyridinium; tetrakisaminophosphonium > triarylphosphonium.^302^ Besides the steric hindrance effect, particular requirements of stereochemistry can also enhance the alkaline AEM stabilities. The following are examples of the latter: Bauer and Strathmann^303^ studied a monoquaternized 1,4-diazabicyclo[2.2.2]octane (DABCO) cation tethered to a poly(ether sulfone) (PES) and found that the resulting AEP was highly resilient to OH^–^ attacks. DABCO contains β-hydrogen, but the rigid cage structure in DABCO effectively hinders antiperiplanar conformation of the N atoms with β-hydrogen. Antiperiplanar conformations are a prerequisite for Hofmann elimination (Figure 32).

The introduction of electron-donating groups in close vicinity of the cations is also investigated. The goal is to hinder OH^–^ attacks by increasing the electron density of the cation. Bis-quaternary ammonium cross-linkers are more susceptible to degradation if two QA cations are close to each other, as two quaternized nitrogen centers strengthen the local environment for electron deficiency.^304^ The stability of alkyltrimethylammonium is higher than that for benzyltrimethylammonium (BTMA). The fact that imidazolium groups with electron-donating substituents improve the alkaline stability over conventional IM groups also supports the electron-donor strategy.^305^ Unsubstituted IMs generally exhibit poor chemical stability in strong alkaline conditions by ring-opening reactions such as S_N_2 reactions, heterocycle deprotonations, and substituent deprotonations.^306^ As mentioned, the stability could be increased by replacing the H in the β-position with electron-donating groups, such as methyl or butyl groups. Price et al.^307^ proposed that the effectiveness of increasing alkaline stability of imidazolium cations was higher for electronic stabilization of the C-2 position versus steric stabilization of the C-2 position. Some IM groups with large substituents can exceed the TMA (trimethylammonium) benchmark; however, the OH^–^ conductivity of IM-based AEPs is lower than that for QA-based AEPs.^308^ Diesendruck and Dekel found that the alkaline stability of BTMA groups, 6-azonia-spiro-[5.5]-undecane (ASU), and large-steric-hindrance imidazolium groups are affected by the λ value (λ = number of water molecules per OH^–^) at room temperature.^309^ The relationship between the λ value and the current density has not yet been revealed experimentally.^308^

Introducing resonance-stabilized structures in or near cationic groups opens another potential antidegradation pathway. The positive charges are delocated over more than one N (or P) atom by using aromatic diamine or multiple N or P systems, leading to resonance stabilization, as seen for, e.g., the heterocyclic imidazolium system.^35^ Another example of a resonance-stabilized AEM structure is *n*-alkylaminophosphonium in poly(ethylene) backbones.^294^ Compared to QA, quaternary phosphonium (QP) cations have attracted less attention. It was found that QP cations containing three trimethylphenyl groups exhibited extremely high alkaline stability exceeding 64 times that of benzyltrimethylammonium.^306^ Their alkaline stability is improved by introducing certain bulky groups. This is due to the strong electron-donating ability of the substitution groups, which can conjugate with the phosphonium cation. However, it is difficult to obtain AEMs with high IECs and good mechanical properties, as the compatibility of quaternary phosphonium with polymer matrixes can be poor. Guanidinium-based cations are also viewed to form resonance-stabilized structures due to charge delocalization along several moieties. Unfortunately, guanidinium does not seem to effectively increase the stability due to its high susceptibility to nucleophilic OH^–^ attacks.^310^

A recent approach to enhance alkaline stability is using cyclic cations as monoquaternized ammonium groups, but these cyclic ammonium groups still degrade mainly via nucleophilic substitution by a ring-opening mechanism in alkaline conditions. However, due to their ring strain, five- and six-ring cyclic ammonium groups have high alkaline stability, even higher than that of seven- or eight-membered rings.^301,311−314^ Some of these groups are low-cost and commercially available.

Metal-based cation groups with organic moieties have been researched and present promising stability at 80 °C in concentrated alkali solutions. Unlike the univalent cations, multivalent metal cations possess the ability to coordinate with more than one anion per cation center. Therefore, the incorporation of multivalent cations in AEMs facilitates the improvement of the IEC, resulting in higher ion conductivities.^306^ Examples of the latter are ruthenium, cobalt, and nickel, in bis(terpyridine) structure and permethyl cobaltocenium, but the corresponding materials can be costly and their synthesis tends to be complex. Tethering metal-based cation groups to a polymer backbone is difficult, and AEPs with metal-based cation groups do not yet show the ion conductivities and low water uptake needed for AEMWEs.

#### 4.1.2 Backbones

Polymeric AEM backbones are base polymers free of cationic moieties. Examples are poly(arylene ether)-based backbones [e.g., polyphenylene oxide, poly(arylene ether sulfone), poly(arylene ether ketone), and poly(arylene ether phosphine oxide)], polyolefin-based backbones [e.g., polyethylene, polystyrene, polynorborene, and polytetrafluoroethylene], polyphenylene-based backbones, and backbones containing cationic moieties [e.g., poly(benzimidazole) and poly(phosphazene)].^36^ Some show very promising alkaline stability; for example, polyphenylene-based AEMs with ketone tethers linked to guanidinium cations were stable in 0.5 M KOH at 80 °C for thousands of hours.^315^

### 4.2 Ionic Conductivity and Other Physical Properties

#### 4.2.1 Ionic-Conductivity Measurement Procedures

The ionic conductivity is a key parameter that is determined by a number of factors such as the IEC, water uptake, microphase separation, type and number of cationic groups, and spacing and clustering of the latter. Measurements of the ionic conductivity and other physical properties of AEMs need to be evaluated and standardized because current methodologies strongly vary between laboratories. The ionic conductivity depends on operation conditions such as the temperature and the viscosity of the electrolyte. Furthermore, the liquid-electrolyte compositions employed for the measurements often vary, such as using NaOH versus KOH solutions, and also in terms of the alkali concentrations. Furthermore, the measured ionic-conductivity value can be lower than the actual value of the OH^–^ conductivity because OH^–^ ions are quickly exchanged by CO_3_^2–^ and HCO_3_^–^ ions if present in the system.^316^ This can be enabled by measurements under ambient conditions where CO_2_ is part of the air.

**Figure 33:**
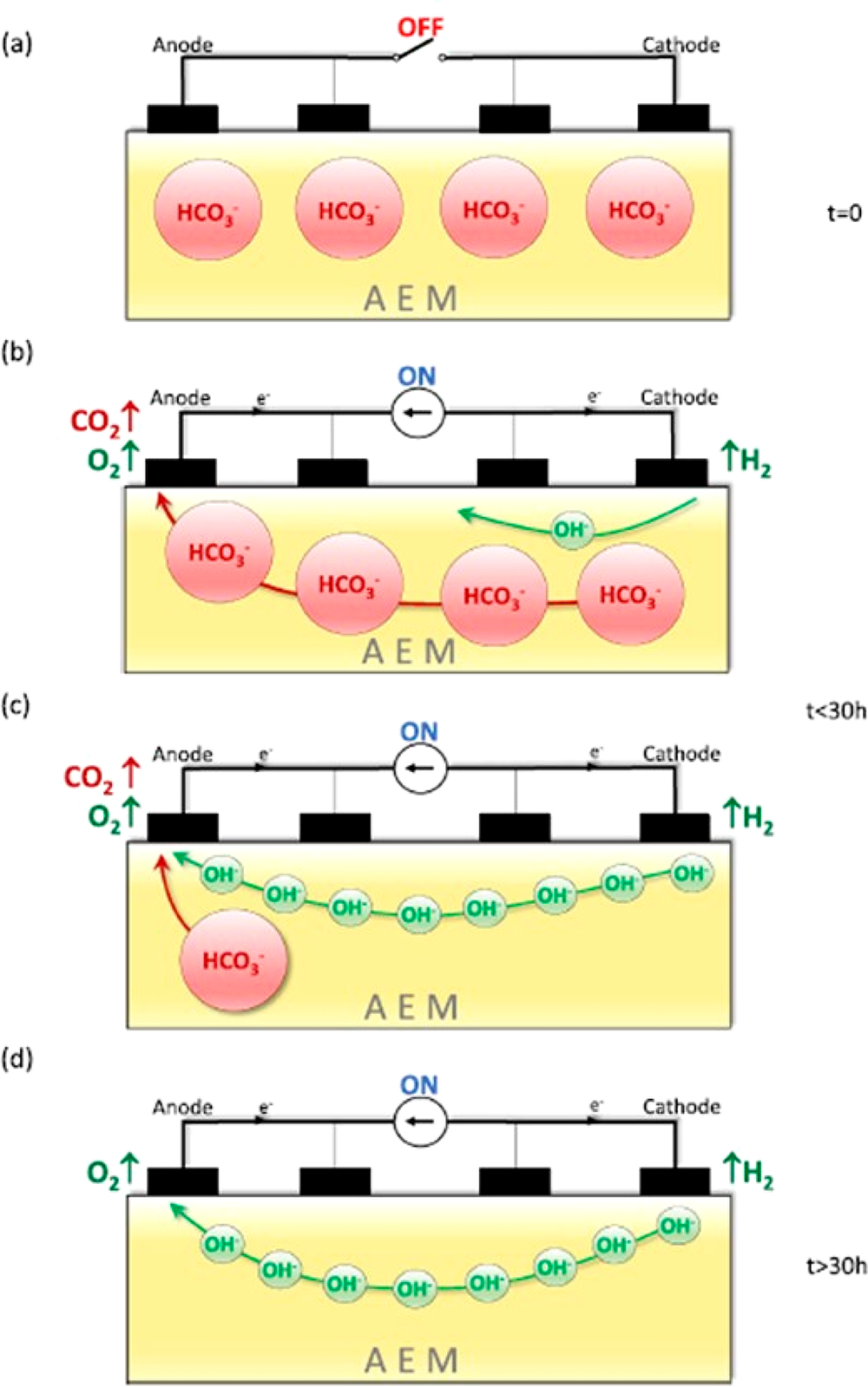
Schematic illustration of the processes taking place in the AEM while applying the direct current under the conditions of the conductivity measurement carried out under N_2_ and H_2_O atmospheres. Adapted from ref (316) with permission. The black rectangular boxes show the sensor electrodes and the anode and cathode for the two H_2_O splitting reactions. Closing the circuit turned the system on, allowing a low (typically 100 μA) current flow. The measurement setup shown in the figure yielded in-plane values.

Consequently, the need for systematic measurements has been highlighted to ensure the determination of the actual OH^–^ conductivity value rather than an apparently lower value resulting from carbonate infiltration into the material of interest.^317^ Therefore, prior to the ionic-conductivity measurements, the removal of air and CO_3_^2–^ and HCO_3_^–^ ions in the N_2_ and H_2_O measuring atmospheres is suggested (Figure 33).

In terms of the measurement procedure, the ionic conductivity is also often measured using four-point measurements determining in-plane values. However, only measurements across the membrane actually determine OH^–^ conductivity between the cathode and anode and, thus, the value that is relevant for an operating water electrolyzer. Therefore, measurements of ionic conductivity should be performed in the through-plane direction.

#### 4.2.2 Methods To Improve Critical AEM Properties

High ionic conductivities of AEMs can be achieved by increasing the IEC value, which is a measurement of fixed cation groups’ concentration. However, there is an optimum value, as high IECs result in high swelling ratios and large water uptake values, both of which are associated with the reduction of the membrane’s mechanical strength. This can be very pronounced, and a material with a very high IEC might be gel-like rather than a solid membrane, which is not desired for AEMWE applications. Therefore, an optimum value between ionic conductivity and mechanical membrane stability has to be achieved.

Strong swelling of the membrane material can lead to delamination of the AEM and catalyst layer. Gas and accompanied bubble evolution in operating AEMWEs was found to cause catalyst delamination upon extensive swelling, especially above 50 °C and for *j* values >0.5 A/cm^2^.^318^ It is very challenging to lower the swelling ratio of AEMs below the target values, which are dry/wet dimensional changes ≤1% in the machine direction and ≤4% in the transverse direction,^32^ without compromising the ionic-conductivity value. Most reported AEMs with ionic conductivities exceeding 0.05 S/cm at 20 °C show swelling ratios in the range of 20–40% and maximum tensile strengths of 16–34 MPa when fully hydrated.^284^ In addition to the ionic-conductivity value and dry/wet dimensions, other target values of AEMs for AEMWE applications are desired such as tensile strength >15 MPa, elongation at break >100%, area-specific resistance (ASR) ≤0.07 Ω cm^2^, and stability ≤0.07 Ω cm^2^ after 2000 h in an AEMWE.^32^

Material-design solutions at the nano- to microlevel are being developed to improve the ionic conductivity and reduce swelling. Currently, the commercial Tokuyama A201 is the AEM with the lowest reported swelling ratio of 6% TD (transverse direction) and 2% MD (machine direction). The ionic conductivity of this AEM is 0.042 S/cm at 90% RH at 20 °C.^36^ A parapolyphenylene-based AEM shows a remarkably low swelling ratio of 9.5%, a promising tensile strength of 35 MPa, and ionic conductivities of 0.049 S/cm at 30 °C and 0.137 S/cm at 80 °C.^319^

### 4.3 Performance-Enhancing Strategies

As discussed, in addition to alkaline stability, AEMs must simultaneously possess high OH^–^ conductivity while maintaining mechanical integrity. The following paragraphs discuss methodologies that have shown some promise to create such membrane properties.

#### 4.3.1 Cross-linking

Cross-linking creates chemical bonds between molecules contained in an ion-conducting polymer with the goal to reduce swelling; ideal cross-linking also maintains the high ionic conductivity of the AEP and AEM. Cross-linking is regarded as a straightforward way to improve thermal, mechanical, and physiochemical AEP properties. High mechanical stabilities are of extra high importance for thin (less than ∼50 μm) AEMs and for AEMWE operation at high differential pressure. Cross-linking can be physical or chemical. Physical cross-linking introduces ion–ion^320^ or van der Waals interactions^321^ between molecules. Chemical cross-linking refers to reagents covalently connected to the AEP. Such cross-linkable reagents can be small compounds, oligomers, or even end groups. Chemical cross-linking can be done as a one-step synthesis^322−324^ or as a post-cross-linking step. Cross-linking approaches have been explored as thiol–ene chemistry,^325,326^ Menshutkin reaction between halo-methylated polymer and commercially available diamines,^327^ ring-opening metathesis polymerization,^328^ olefin metathesis,^329^ and thermal cross-linking.^330^ Cross-linking has been shown to be beneficial, but swelling cannot be completely eliminated.^328,293,331^ The swelling and corresponding OH^–^ attacks on the AEP backbones and the functional groups can be reduced, but the ionic conductivity and processabiliy will be reduced if the linking occurs via the ion-conducting end groups. Multication side-chain or end-group cross-link strategies have been proven as effective; for example, Chen et al.^332^ reported a series of multication cross-linked membranes with high OH^–^ conductivity (0.155 S/cm at 80 °C) and good dimensional and alkaline stability. Lee et al.^333^ prepared a series of end-group cross-linked polysulfone (PSF) membranes by introducing a benzyne group at the end of the PSF polymer chain. The cross-linking improved the ionic conductivity (0.11 S/cm at 80 °C) and dimensional stability.

If not appropriately applied, cross-linking can result in poor AEP and AEM properties, for example, cross-linkers with long chains were shown to induce crystallinity into AEMs, compromising many physicochemical properties such as reducing the hydrophilicity.^334^ A study of poly(2,6-dimethyl-1,4-phenylene oxide) using hydrophilic cross-linkers that contained ethylene oxide (EO) showed that the presence of long EO cross-linkers increases the degree of crystallinity but reduces both the ionic conductivity and the alkaline stability.^334^ If not dosed correctly, cross-linking can be too strong, resulting in mechanically brittle AEMs and possibly poor alkaline stability. Furthermore, additional reaction steps complicate the processing of the membrane.^40^

Interaction of interpenetrating polymer networks (IPNs) allows for surpassing the mechanical strength of the original polymer with high IECs.^335^ Theoretically, IPN AEMs create networks made of a continuous ion-conductive phase, while non-ion-conductive networks maintain the mechanical stability. The networks interlace on the molecular scale without being covalently bonded. Examples are an IPN AEM based on poly(vinyl alcohol)/polyethylenimine and an IPN AEM cross-linked quaternized poly(epichlorohydrin)/polytetrafluoroethylene (PTFE).^336−339^ Reported IPN AEMs do not yet meet the mechanical strength requirements but show potential. For example, a cross-linked poly(vinyl alcohol)/cross-linked poly(vinyl benzyl-*N*-methyl piperidinium) IPN AEM yielded a high ionic conductivity of 0.258 S/cm at 80 °C, a moderate IEC value of 1.75 mmol/g, and an encouraging tensile strength of 9.3 MPa in the wet AEM state.^340^

#### 4.3.2 Microphase Separation

The ion-conducting polymers can contain wetting (typically the ion-conducting part) and nonwetting parts. When such a polymer is in contact with a liquid such as water or an electrolyte, the polymer molecules can reorient in a manner such that the wetting parts of the polymer are in contact with the liquid, resulting in the formation of liquid clusters. If the molecules contain specific spatial properties, the wetting and nonwetting parts create two phases. This effect is called microphase separation. The formation of a hydrophilic/hydrophobic microphase separation structure is relevant for the preparation of high-performance AEMs; percolating-liquid ionically conducting domains, which are called ion channels, can be created.^341^

**Figure 34:**
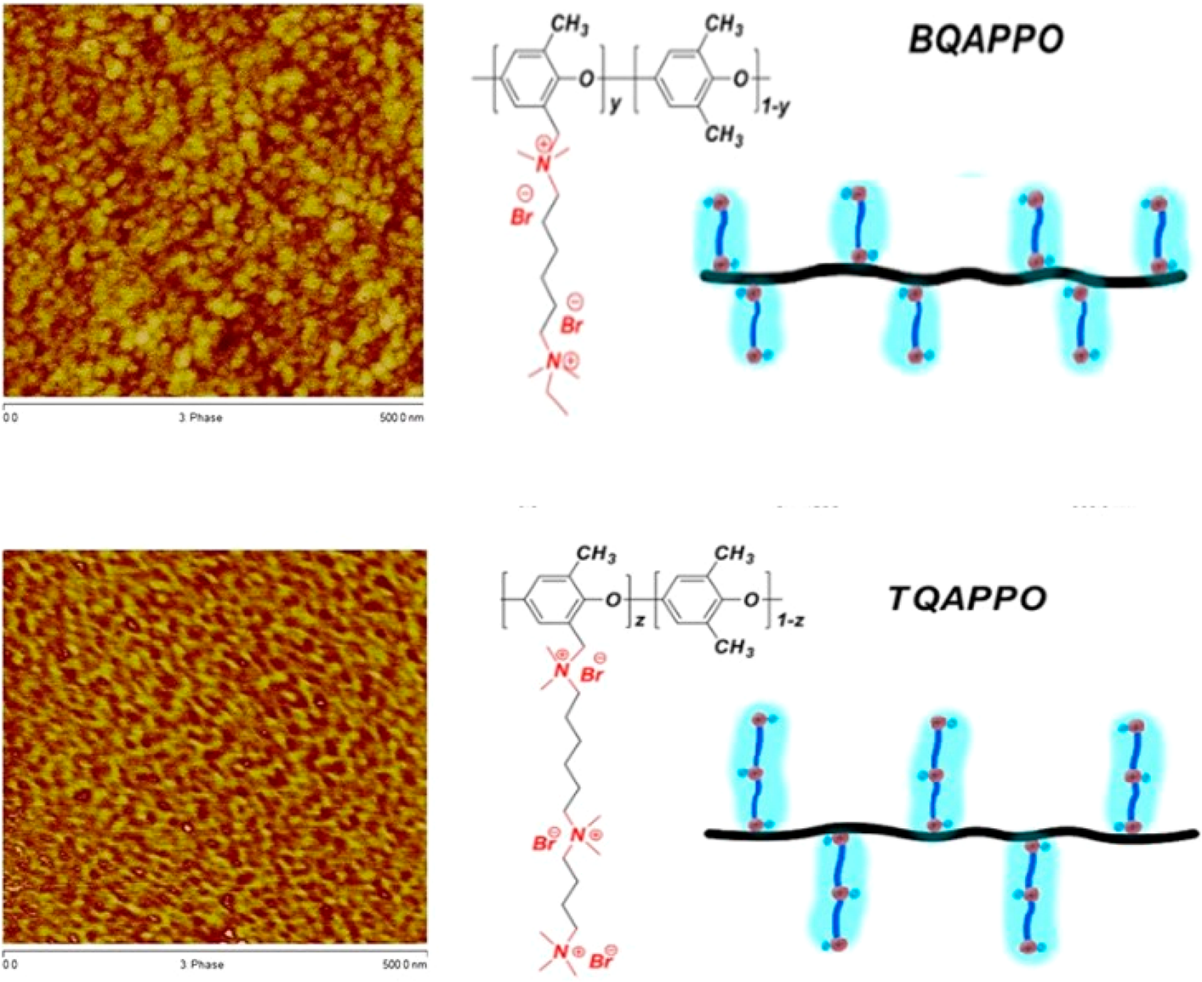
AFM tapping phase images revealing the architecture–morphology–properties relationship of AEMs (BQAPPO and TQAPPO). The *x*–*y* scales in the AFM images are 100 nm per square. The bright and dark domains in AFM images are designated as the hydrophobic and hydrophilic phases, respectively. Adapted with permission from ref (317). Copyright 2015 Springer Nature.

An important design criterion is to maximize the population of percolated ionic domains to enhance the ionic conductivity, although the ion-conductive domains need to be uniformly distributed across the AEM.^342^ Such a microphase-separation-structure control approach is promising to achieve both high ionic conductivity and high mechanical stability. The backbones, tethering chains, and molecular structures of the headgroups strongly influence the formation of the AEM microphase, altering the ionic conductivity and the water uptake.^343,344^ Correspondingly, the location, type, and concentration of cations and hydrophobic side chains need to be tuned in order to achieve optimum 3D phase-separation structures, and these are regularly investigated.^345^ Important strategies for optimum phase separations are the location of the wetting ion-conducting moiety in side chains or multiblock copolymers containing wetting and nonwetting alternating sections.^40,346−349^ In the aforementioned side-chain-type AEPs, the side-chain length, characterized by, e.g., the number of alkyl spacers, has been suggested to have a significant effect on the AEP’s performance, with five or six alkyl spacers being the optimum design.^350^ To reveal the morphology–properties relationship, atomic force microscopy (AFM) and transmission electron microscopy (TEM) have been employed. As an example, both the hydrophilicity and the flexibility of ionic side chains have been shown to play crucial roles in fabricating high-performance AEMs.^317^ This is shown in Figure 34, where the increased hydrophilicity and flexibility of ionic side chains (named TQAPPO) showed well-defined and well-distributed hydrophilic microphase separations.

#### 4.3.3 Organic/Inorganic Composite AEMs

**Figure 35:**
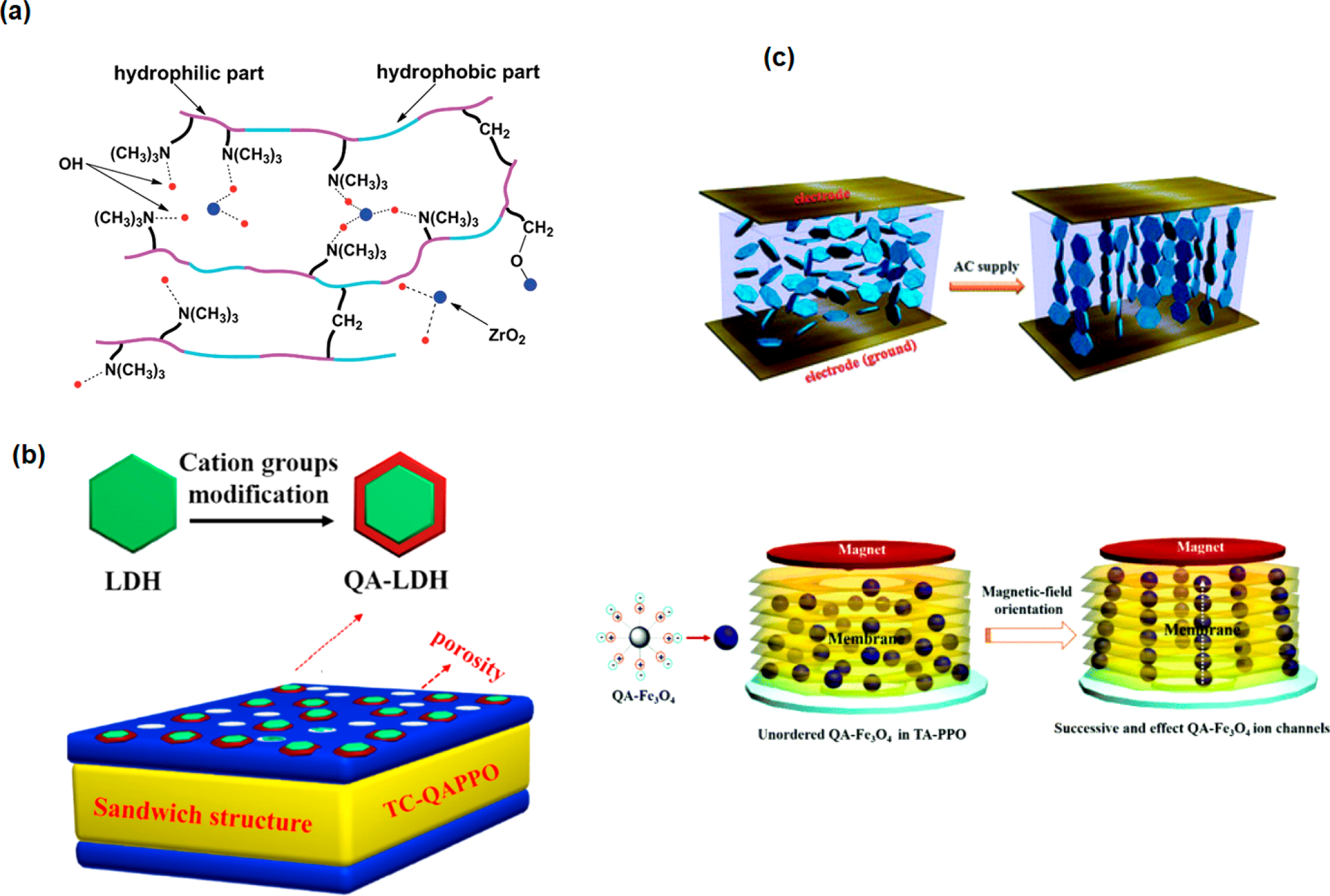
(a) Structure of a composite copoly(arylene ether sulfone)/nano-ZrO_2_ AEM designed to simultaneously achieve a high ionic conductivity, low water uptake, and improved thermal, mechanical, and chemical stabilities. Adapted with permission from ref (357). Copyright 2014 Royal Society of Chemistry. The blue dots are nano-ZrO_2_. (b) Porous-sandwich structure composite AEMs. Adapted with permission from ref (358). Copyright 2018 Elsevier. (c) Electric-field-oriented and magnetic-field-oriented composite AEMs. Adapted with permission from refs (359 and 360). Copyright 2014 and 2018 Royal Society of Chemistry, respectively.

Organic/inorganic composites are another strategy to improve AEM performance (Figure 35). Composite AEMs consist of two classes: mixed-matrix membranes embedding inorganic nanoparticles in organic AEPs and membranes made of an inert porous support filled with AEPs.^351^ Mixed-matrix membranes are gaining popularity due to a wide range of embedding materials such as metal ions, metal oxides, silica, functionalized nanoparticles, graphene oxide, and carbon nanotubes.^352^ The particles and the porous support membrane in composite AEMs are typically nonionic and curb water uptake, while the polycations provide high ionic loadings, facilitating ion conduction. Previous work encompassed composite AEMs that showed an increase in ionic conductivity as well as thermal, chemical, and mechanical stability while reducing the water uptake.^352−355^ However, the validation of the results in AEMWE cells is often missing.

The nanoparticles need to be uniformly dispersed in the organic phase, and they need to be alkaline-resistant.^356^ Correspondingly, particles such as silica and alumina are not recommended, while zirconia particles are promising. A poly(vinyl alcohol) (PVA)/PDDA/nano-ZrO_2_ composite AEM with 2.5 wt % ZrO_2_ showed properties such as a maximum tensile strength of 13.96 MPa and an elongation of 229%, while the 1.5 wt % nano-ZrO_2_ AEM yielded the highest ionic-conductivity value of 0.032 S/cm at 20 °C. Single-AEMWE-cell results using nano-ZrO_2_ incorporated into a commercial Sustainion membrane show promise and highlight the potential to increase H_2_ and O_2_ separation.^303^

Composite AEMs strengthened with porous, woven, or electrospun substrates have shown enhanced ionic conductivities and reduced swelling. The substrates are usually chemically inert, and mechanically stable AEPs such as high-density polyethylene, polypropylene, polystyrene, polyimide, or polyolefin are used. A 125-μm-thick noncomposite AEM showed a mechanical failure at 2000 h, while a thinner (60–90 μm) reinforced version did not fail over 4500 h in an AEMWE cell at 30 bar and 80 °C.^35^ Chen et al.^358,360^ designed a series of QA-functionalized LDH/poly(*p*-phenylene oxide) (PPO) composite membranes with a porous sandwich structure with a high ionic conductivity of 122 mS/cm at 80 °C. Another interesting approach is to design aligned composite membranes. Fan et al.^359^ and Chen et al.^360^ designed electric- and magnetic-field-oriented composite membrane series, respectively. The ionic conductivities of aligned composite membranes displayed improvements of 39% for electric-field-oriented composite membranes and 55% for magnetic-field-oriented composite membranes over the corresponding nonaligned composite membranes.

### 4.4 Promising AEM Examples and New Research Directions

**Figure 36:**
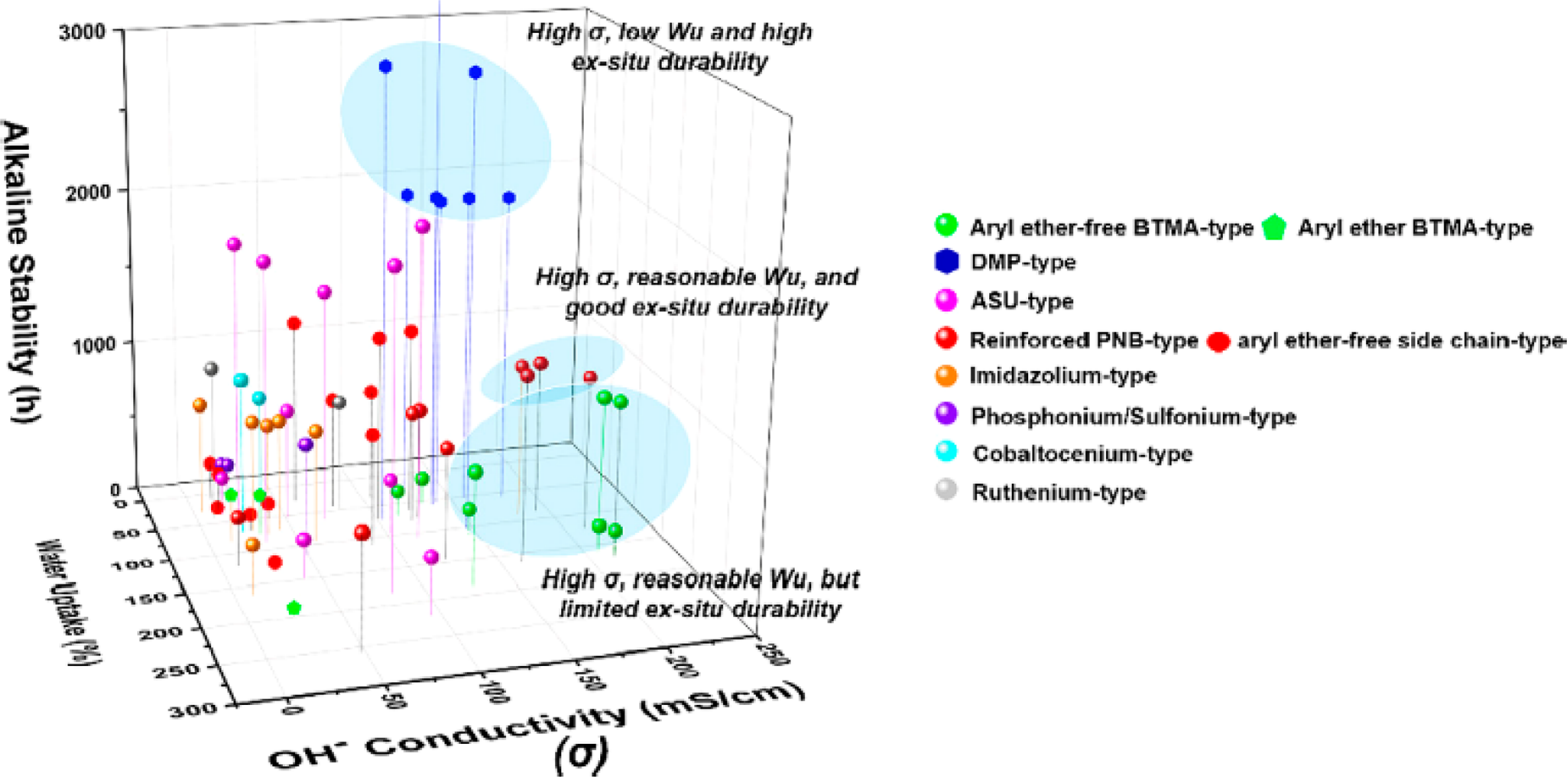
Comparison between the water uptake, OH^–^ conductivity (σ), and ex situ stability of typical BTMA-, DMP-, ASU-, side-chain-, imidazolium-, phosphonium/sulfonium-, cobaltocenium-, and ruthenium-type AEPs. The water uptake (*W*_u_) corresponds to the σ value at the same temperature (most AEPs are recorded at 80 °C, but some for the side-chain-, imidazolium-, sulfonium-, and ruthenium-type AEPs are plotted at room temperature and 60 °C due to insufficient information). The alkaline stability was recorded based on the temporal stability of AEPs in 1 M NaOH or KOH at 80 °C with degradation <10%, and some of the stable AEPs were evaluated at harsher conditions. Adapted with permission from ref (350). Copyright 2021 Elsevier.

In sections 4.1–4.3, critical AEP and AEM properties and methods of improving them were discussed, while in this section a summary highlighting promising AEM developments and research direction trends is presented. Before 2010, an ionic conductivity of 0.010 S/cm at 60–80 °C was the target for AEMs.^361^ Since then the target has increased 10-fold,^284^ and several AEMs have exceeded 0.2 S/cm.^362−367^ Current research on AEMs often aims on increasing the mechanical and chemical stabilities as well as breaking the operational temperature limits. Chen and Lee summarized the ex situ durability, OH^–^ conductivity, and water uptake of different types of AEPs (shown in Figure 36).^350^*N*,*N*-Dimethylpiperidinium (DMP)-type AEMs displayed an outstanding alkaline stability (ex situ durability) and a relatively high conductivity. Some reinforced polynorbornene (PNB)-, 6-azonia-spiro-[5.5]-undecane (ASU)-, and aryl ether-free BTMA-type AEMs also show overall high performances.^350^

On the basis of intensive research efforts achieved over the past decade, future trends in AEP designs are emerging. AEPs with noncyclic QAs are still a focus, mainly because they are easy to obtain and have stable AEP backbones, and the properly designed side chains avoid both Hofmann elimination and counterion condensation. Heterocycloaliphatic QAs will likely attract more attention because they show high stability in alkaline solutions.^301^ The alkaline stability of heterocycloaliphatic QA cations critically depends on their position in the AEP structure, the ring size, the presence of an additional heteroatom, and ring-substitution patterns.^368^ Spirocyclic QA cations are a special type of aliphatic heterocyclic QA with unique structures. This class of AEPs exhibit extraordinary alkaline stability because the spirocyclic structure has a high transition-state energy against degradation reactions.^369^ Reported studies include examples such as incorporating QA salts into the polymer backbone, attaching them directly onto the aliphatic or aromatic polymer backbone, and introducing them as a cross-linker to form a network.^312,313,370,371^ In terms of investigating the relationship between the alkaline stability and the cation structure of AEPs, in situ AEMWE cell tests are needed. The importance of in situ AEMWE cell testing of AEMs has been highlighted by a study carried out by Meek et al.,^372^ who also defined a testing protocol. Some AEPs exhibited excellent ex situ durability.^314^ However, in situ and ex situ results are generally not in agreement. For example, it was found that BTMA-PPO with poor alkaline stability showed acceptable in situ durability at 0.1 A/cm^2^, while side-chain-type PPO exhibited a significant voltage loss.^373^

With regards to AEP backbones, ether-free backbones are a preferred choice for structure design, as polybenzimidazole (PBI)-, polyphenylene-, and polyolefin-type AEMs have been widely explored. PBI- and polyphenylene-type AEPs have high thermal and good chemical stabilities.^374^ The unique benzimidazole repeating units in the backbone provide a high density of electronegative pyridine nitrogens (—N=) and can form hydrogen bonds to conduct OH^–^. However, their processability and ionic conductivity are too low for AEMWEs. Current research focuses on improving the ionic conductivity by alkali doping and enhancing the solubility by introducing ether bonds into the main chains. To improve the performance of doped PBI, several approaches have been developed, such as tuning the porosity, building sandwiched-porous PBI, and fabricating AEP blend systems.^375−378^

In Table 5 the structures of state-of-the-art AEPs associated with good ex situ ionic conductivity and alkaline stability are summarized to guide future AEP design. The examples shown in Table 5 exceed both ion-conductivity values of 0.1 S/cm at 80 °C and an ex situ stability of 500 h.^350^ AEMs tested specifically in AEMWE single cells are summarized in Table S19. Furthermore, protocols for AEM evaluation have been discussed in the literature, as mentioned earlier.^314^

**Table 5:**
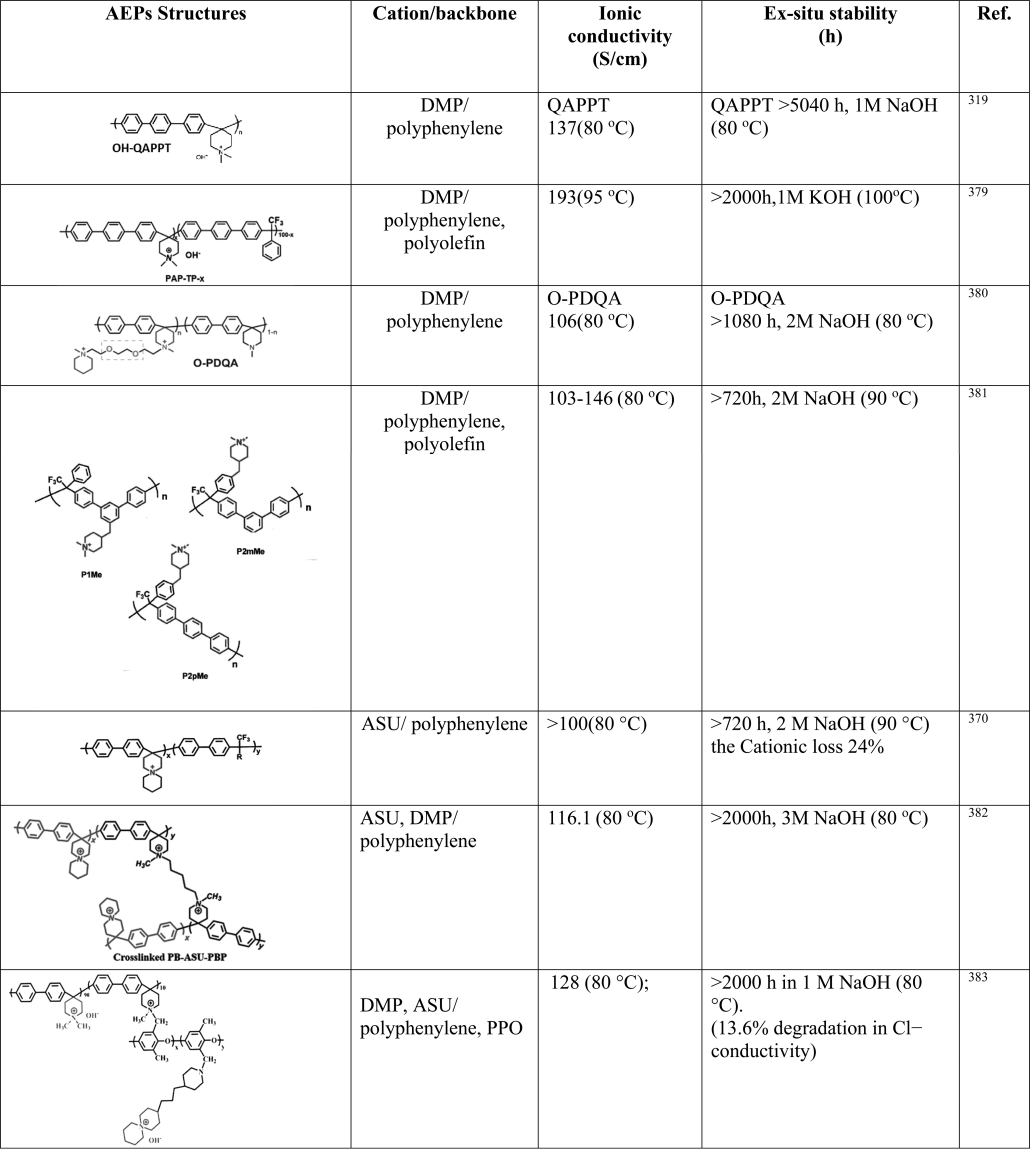

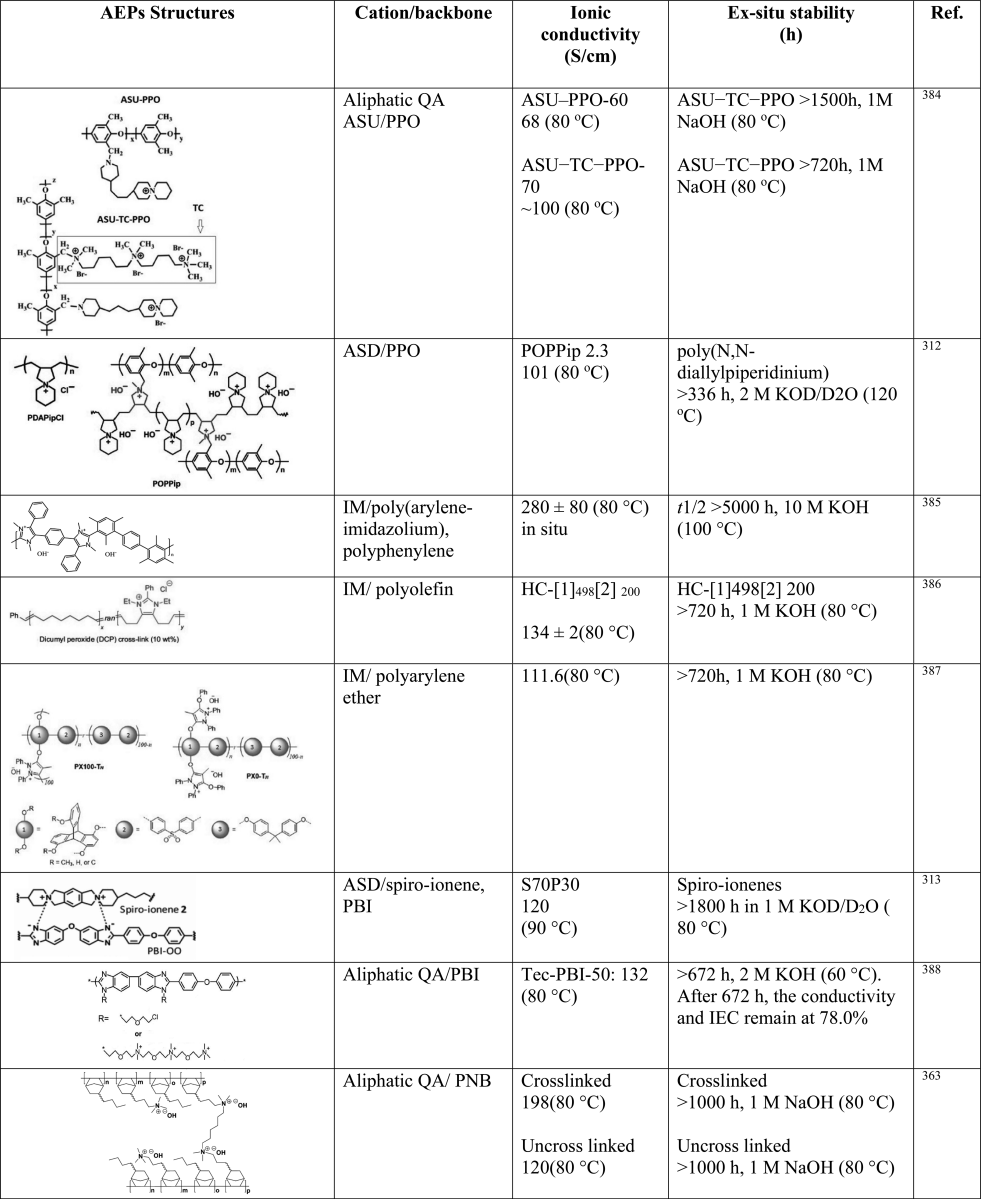

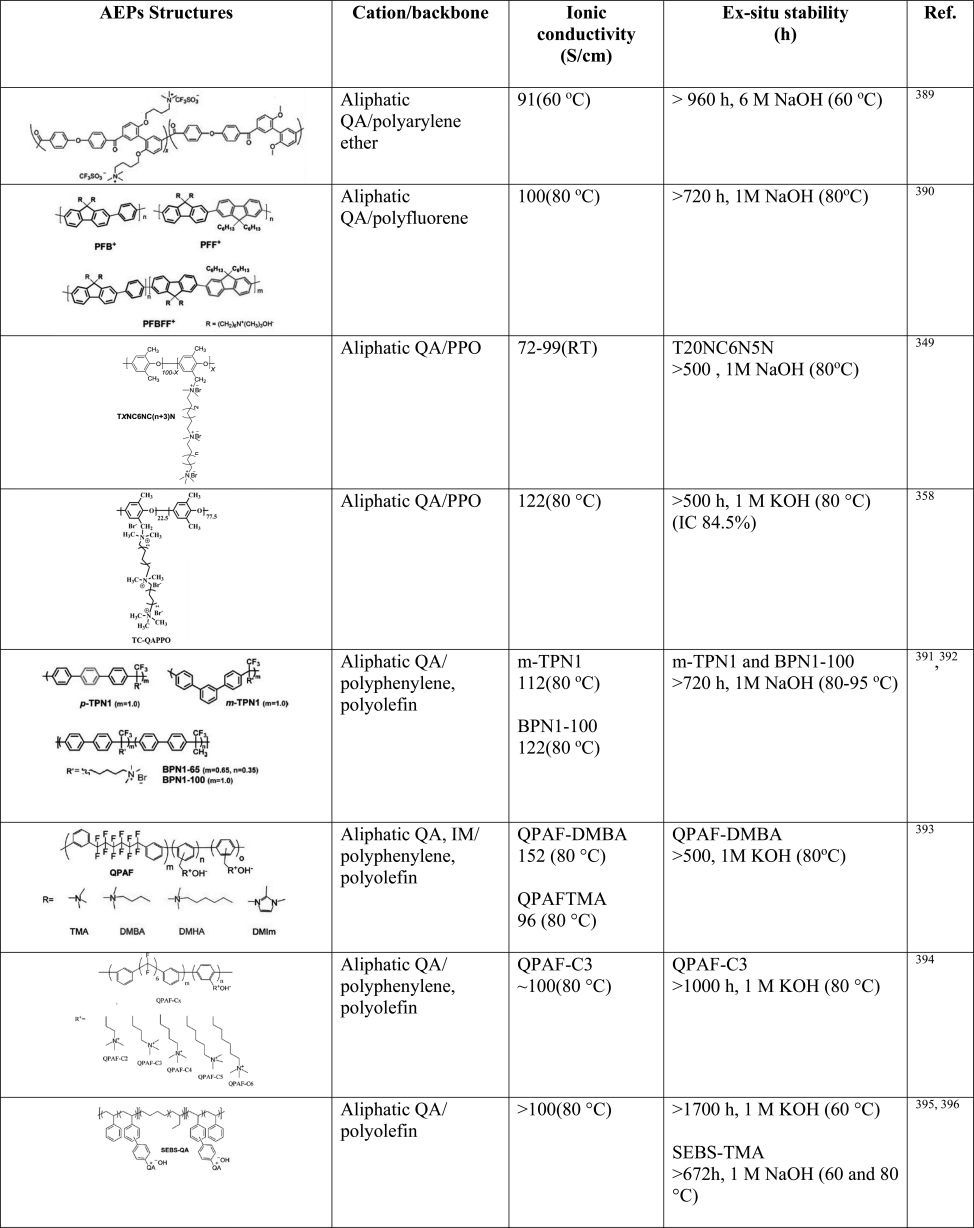

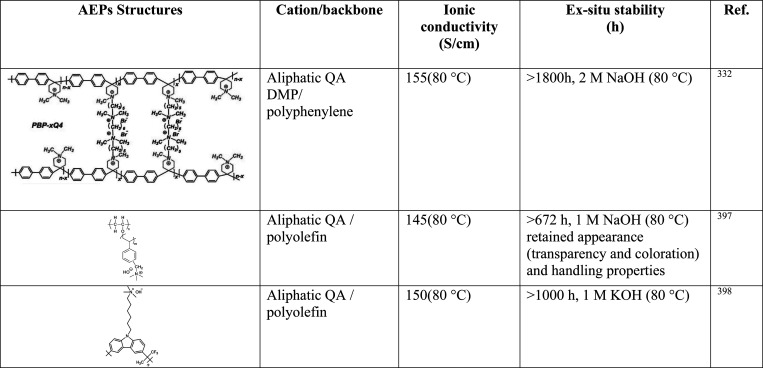
Summary of Recent Research Progress for AEPs of Ionic Conductivity Exceeding 0.1 S/cm at 80 °C and Ex Situ Stability Longer than 500 h

## 5 Membrane Electrode Assembly

Within the membrane electrode assembly (MEA), the membrane and catalysts are integrated into a functional unit. The membrane must provide mechanical stability during the compression of the MEA and the transport layers as well as enable ionic transport while inhibiting gas and electron crossover. The catalyst layer can be seen as the central interfacial layer in an MEA, where all transport pathways including chemical species, ions, and electrons need to come together at the reactive centers, which is the catalyst particle surface. In this section, we first discuss how to design optimum catalyst-layer structures. This is followed by a discussion on possible pathways for integration of the catalyst layer with the membrane and the manufacturing of MEAs.

### 5.1 Catalyst-Layer Design

**Figure 37:**
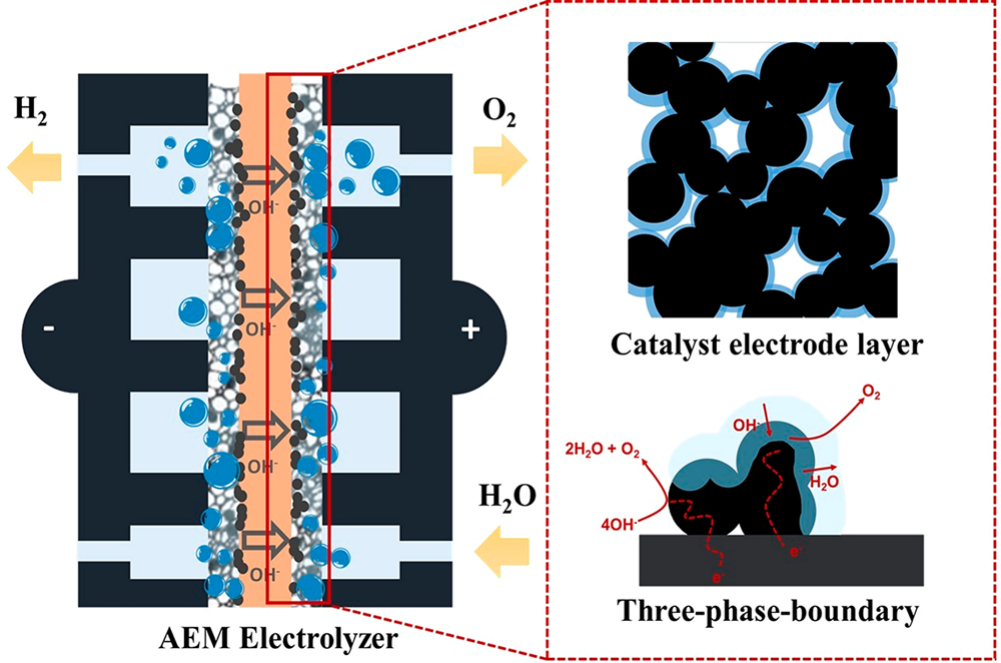
Simplified schematic of the triple-phase (gas, liquid, and solid) boundary for the OER showing the catalyst particles (black) that are in direct contact with the current collector (shown as a gray bar in the figure). The OH^–^-conducting AEI acting as an electrolyte and often also as a binder is shown in blue. In an actual MEA, the catalyst particles form up to several-micrometer-thick layers, and electronic conductions through the catalyst layer (from catalyst particle to adjunct catalyst particles) are needed.

Catalyst-layer design is about achieving optimum conditions for the transport pathways of the involved species. Here electrons need to be transported through electronically conducting pathways, which are the catalyst particles and the metals of the current collectors. Usually, the ions are transported via both the liquid and the solid electrolyte, and the reactants are liquid water and gases that are transported through the pores. These catalyst particles and the transport pathways are intertwined, which has to be considered for catalyst-layer design and which goes beyond the concept of catalyst activity.^399^ Catalyst-layer design seeks to find simple ways of manufacturing porous structures that have high-density reaction points within the so-called triple-phase (gas/solid/liquid) boundary (Figure 37).^400^ This is a three-dimensional area within the catalyst layer where the reactions (eqs 2 and 3) occur.^400^ Here the catalyst is the solid, the OH^–^ conductor is the “liquid”, and H_2_ and O_2_ are involved as gaseous species. To achieve a high number of these sites, the integration of the catalyst into the MEA in terms of the catalyst-layer structure is critical. The anion-exchange ionomer (AEI), an AEP, is the sole OH^–^ conductor in the case of a water-only feed, while OH^–^ conductivity is facilitated by an alkali electrolyte feed. The AEI needs to be integrated with the catalysts to provide high OH^–^ conductivity without blocking the catalyst sites and to allow for catalyst-layer porosity, facilitating the escape of the H_2_ and O_2_ products. Also the AEI acts as a binder to create a mechanically stable catalyst layer from the catalyst powder. In addition, the catalyst sites need to be electronically connected to the current collector for the electrochemical reactions to occur. High electronic conductivities of the catalysts and supports are needed, but the electronic conductivities on their own do not directly yield the highest-performing MEA, thus emphasizing the importance of the manufacturing methods and the catalyst integration into the MEA.^401^

It is crucial that the AEI is dispersed in a manner achieving maximal catalyst utilization and facilitating OH^–^ transport from the cathode to the anode catalyst sites through a continuous and highly conductive pathway. The OH^–^ conductivity of a catalyst layer can be 1 order of magnitude lower than that for the equivalent AEM and is influenced by the catalyst layer’s tortuosity. The latter can be seen as the mean deviation of traveling time from the shortest possible connection within a porous material.^31^ The optimal AEI loading typically ranges between 5 and 20 wt %, depends on numerous factors, and must be experimentally evaluated.^402^

**Figure 38:**
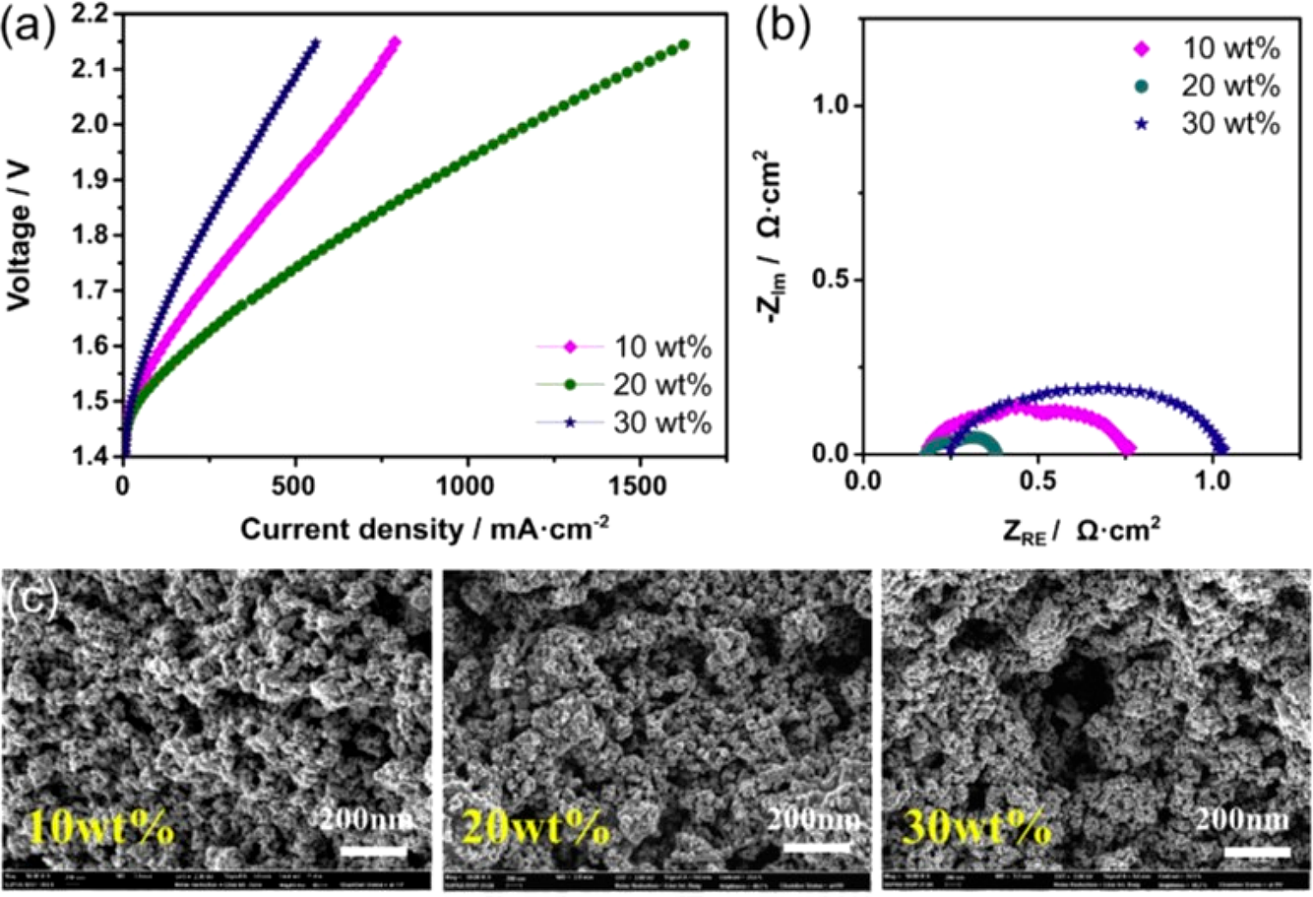
(a) Polarization curves and (b) Nyquist plots for AEMWEs with different (10, 20, and 30 wt %) AEI loadings at 50 °C and (c) field-emission (FE)-SEM images of the MEAs fabricated using the different AEI loadings. KOH (1 M) at 1 mL/min was fed to the anode and cathode. Reprinted with permission from ref (107). Copyright 2019 Elsevier.

An example of the influence of the AEI loading on various MEA properties and performance is shown in Figure 38. For these MEAs, tested in a single cell, the lowest voltage at a particular current density value is found for the 20 wt % AEI loading. The Nyquist plots (Figure 38b) further suggest that the high-frequency resistance (HFR) and the resistances for the anode and cathode charge-transfer reactions are the lowest for the 20 wt % AEI loading.^107^ The SEM images show differences in pore structures and the appearance of secondary pores for higher AEI loadings. The latter are suggested to lower the cell performance.

An understanding of how a specific AEI, which also acts as a binder for the catalyst particles, behaves in regards to factors such as its swelling and conductivity in feed electrolytes with different pH values will be crucial in understanding the AEI’s influence on transport in the pore phases.^403^

**Figure 39:**
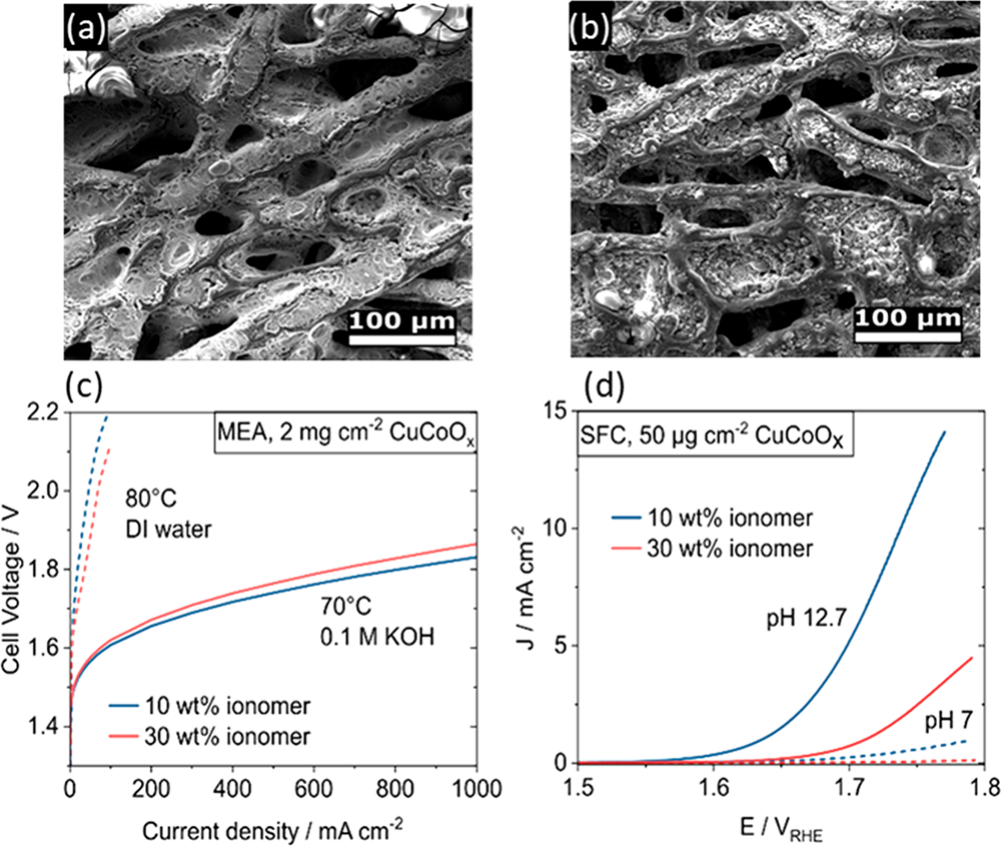
SEM images of 2 mg/cm^2^ CuCoO_*x*_ anode catalyst layers with (a) 10 wt % and (b) 30 wt % ionomer loadings.^404^ (c) Polarization curves of the single AEMWE cells for pure water (dashed lines) and for 0.1 M KOH (solid lines) feed. (d) *iR*-corrected linear sweep voltammograms of the CuCoO_*x*_ anode catalyst layers with varying ionomer contents at pH 12.7 (solid) and pH 7 (dashed) of a 0.05 M phosphate buffer solution in a scanning-flow-cell measurement. A Pt loading of 0.5 mg/cm^2^ was used at the cathode. (c, d) Reprinted with permission from ref (403). Copyright 2022 Elsevier.

Mayerhöfer et al. recently studied the effect of 10 and 30 wt % AEI loadings in the anode catalyst layer on the AEMWE performance in water and 0.1 M KOH (as illustrated in Figure 39a and b).^403^ It was found that the employed AEI by itself, even at 30 wt %, was not able to supply the required OH^–^ species and the basic environment for the PGM-free OER catalyst sites during pure water feed operations (Figure 39c). However, the performance increased by 20–45 times at a cell voltage of 1.8 V when a 0.1 M KOH feed was used.

In addition, a three-electrode scanning-flow-cell (SFC) experiment was conducted to investigate the anode catalyst layer. It was shown that a higher binder content can block the catalyst site and consequently lower the catalyst activity (Figure 39d) for the higher-pH feed. This effect was ascribed to the changes in the membrane- and contact resistances due to different swelling behaviors of the materials in the respective feed solutions. This shows that the role of catalyst-layer binders can differ significantly depending on the feed solutions.

The concept of not needing an AEI if an alkali electrolyte feed is applied has also been considered in a few studies.^31,80^ For this situation, the AEI may merely act as a catalyst particle binder and the liquid alkali electrolyte may be the vital OH^–^ provider.

**Figure 40:**
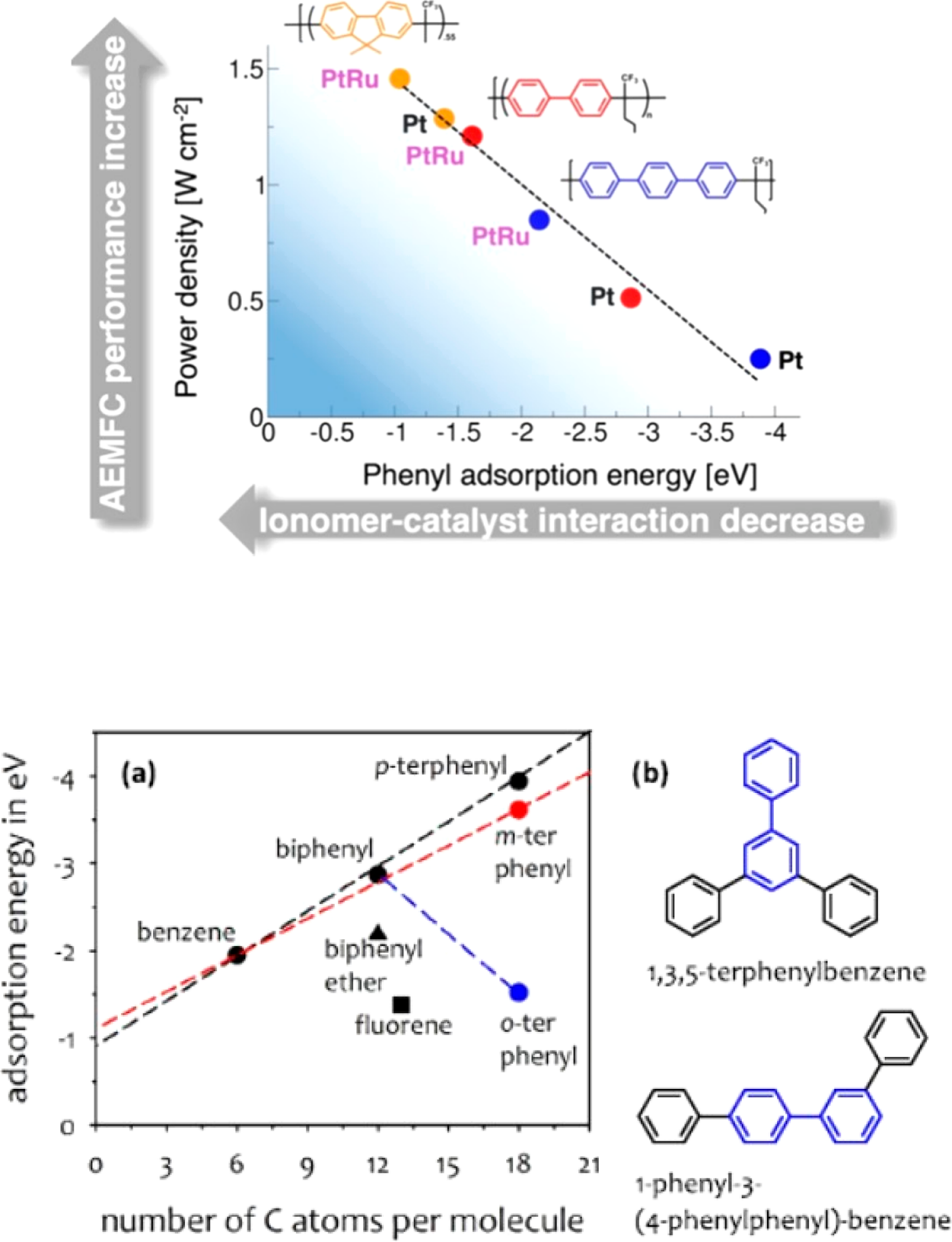
(Top) Dependency of the performance for an AEMFC (*y*-axes) on the adsorption of phenyl from the ionomer for different cathode (H_2_ oxidation) catalysts. The lower figure shows DFT-calculated adsorption energies for different substituted benzenes on Pt as a function of the system size (C atoms per molecule). Adapted with permission from ref (409). Copyright 2019 American Chemical Society.

The AEI also influences the pH and hydrophobicity of the catalyst layer. The AEI needs to have the appropriate chemical properties, which include maintaining a pH favoring the catalyst’s chemical stability and hydrophobicity and the need to be mechanically stable, to prevent catalyst detachment. For example, the p*K*_a_ of the conjugated acid of the QA cationic group or of some AEIs is lower than that for KOH, ∼10 versus 15.^405^ The choice of KOH (or other liquid alkali electrolytes) feeds also seems preferable for stability reasons in the case of, e.g., Ni-based catalysts. A high pH can also enhance the OER kinetics depending on the reaction order of the catalyst.^318^ Chemical similarity between the AEI and AEM allows for a low interfacial resistance and similar swelling, which helps to prevent delamination of the catalyst layer from the AEM.^32^ The functional groups and the backbone structure of the AEIs can be used to tune the hydrophobicity and chemical properties of the triple-phase boundary, which allows for pH adjustment and alters H_2_O availability at the catalyst sites. Chemical groups of the AEI can react and create negative side effects at both electrodes.^406^ In AEMFCs, aromatic AEI groups have been shown to adsorb on the Pt cathode catalysts, lowering the catalytic utilization (Figure 40).^294,406−408^ Experiments combined with DFT studies suggest that the adsorption of aromatic AEI fragments on Pt metal surfaces decreases as follows: *p*-terphenyl ≥ *m*-terphenyl > diphenyl ether > benzene ≥ *o*-terphenyl > 9,9-dimethyl fluorine, and the parallel adsorption of the adsorbed phenol ring on the Pt surface has a negative effect.^409^ The specific adsorption of QA cations and benzyl-group interactions with Pt can be lowered by utilizing large, rigid cations and nonrotatable phenyl groups,^410^ although the unsubstituted phenyl in polyaromatic backbones stays adsorbed on Pt well into positive potential regions.^411^ The adsorption energy depends on the catalyst. In the case of benzene, it may be lower for bimetallic surfaces such as Pt alloyed with Mo, Ni, or Ru.^412,413^

**Figure 41:**
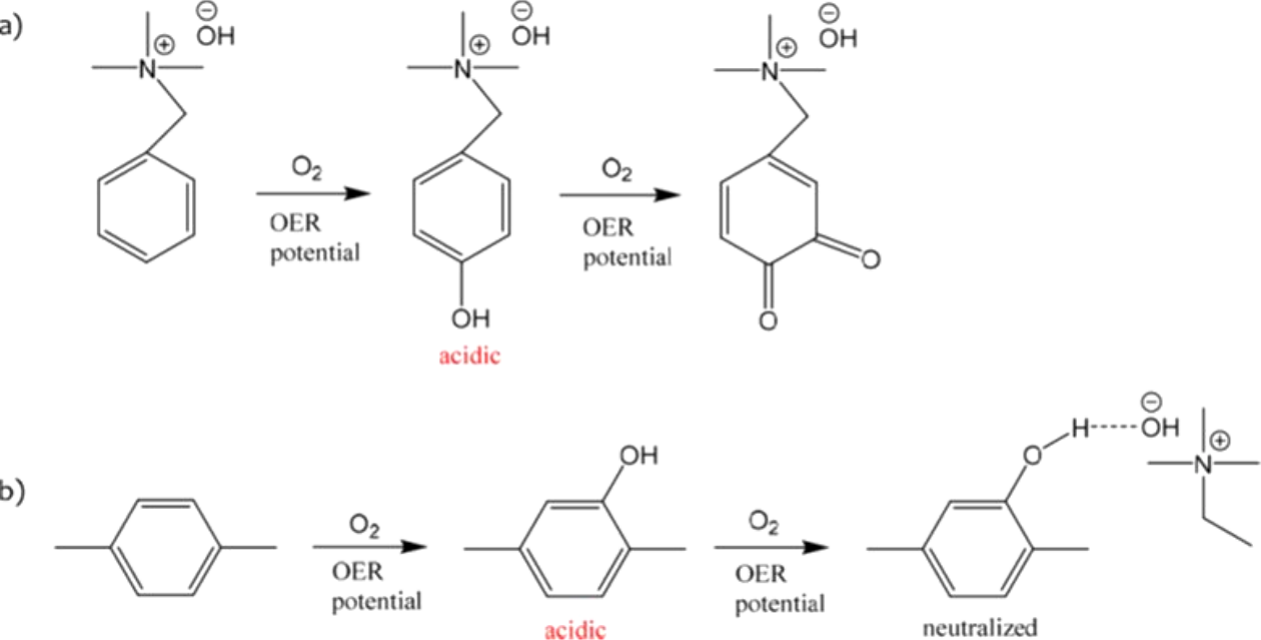
Phenyl oxidation of (a) benzyltrimethylammonium hydroxide and (b) polyaromatic AEI at OER potentials. Adapted with permission from ref (415). Copyright 2019 American Chemical Society.

In addition, catalyst-site blocking side effects such as lowering the pH, possibly causing dissolution of TM catalysts, can also occur.^414^ This has been proposed for AEIs containing phenyl groups in the backbone structure, which can be oxidized to acidic phenolic compounds (Figure 41).

**Figure 42:**
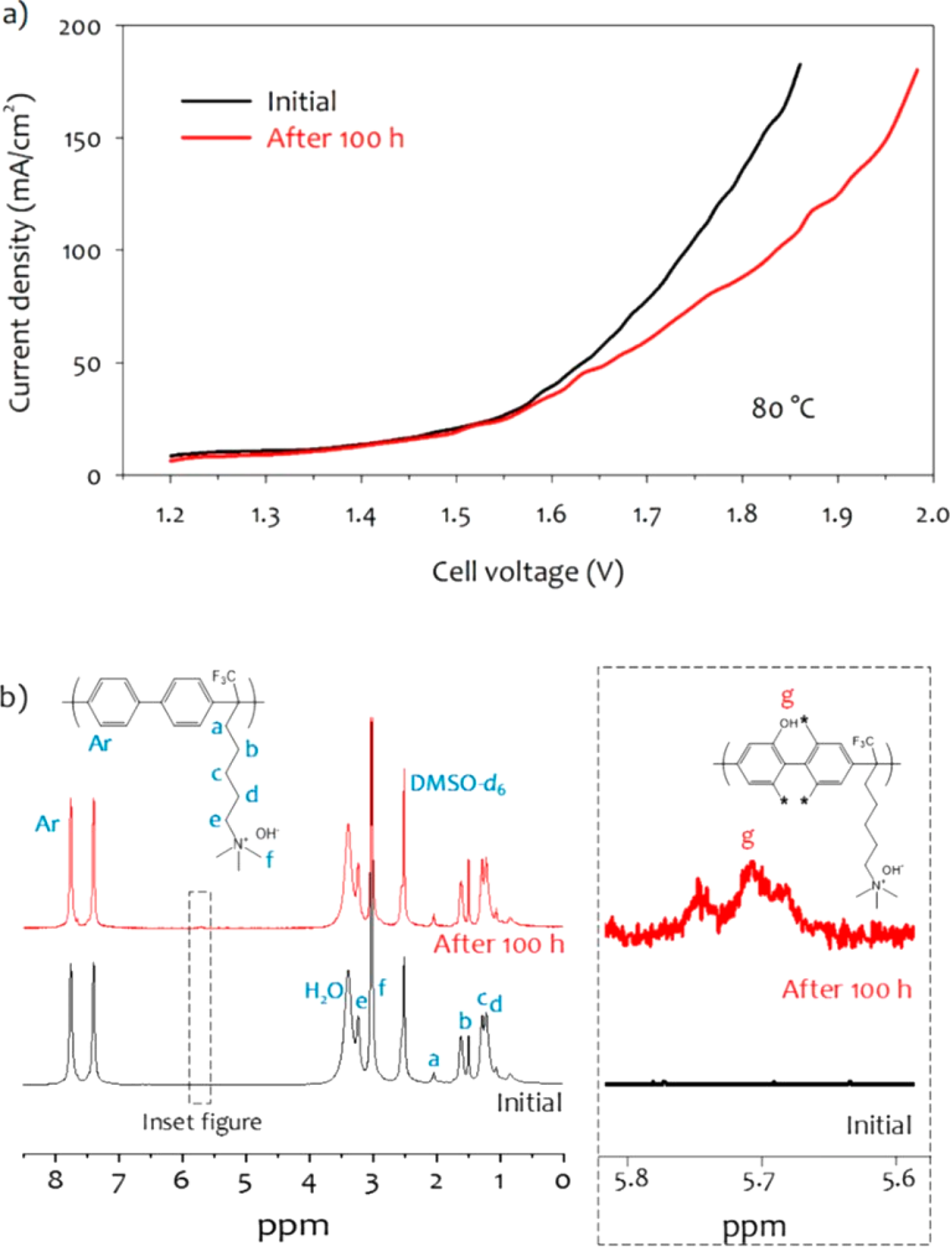
(a) Polarization curves of an AEM water electrolyzer before and after the 100 h test at 2.1 V at 80 °C. (b) ^1^H NMR spectra of the anode AEI before and after the durability test. The inset in (b) is the expanded view of the oxidized phenol peak in the ^1^H NMR spectra; * denotes other expected oxidation sites. Adapted with permission from ref (415). Copyright 2019 American Chemical Society.

DFT calculations suggest that phenyl adsorbed on the electrode surface in parallel or lying positions is most susceptible to oxidation, and both positions are observed at potentials as high as 1.6 V (Figure 42). The adsorption energy depends on the surface as follows: PtO_2_ (110) > IrO_2_ (110) > PtO (110) > IrO( 110) > La_0.85_Sr_0.15_CoO_3_ (001) > La_0.85_Sr_0.15_CoO_3_ (111).

Studies also involve the use of PTFE as a binder in catalyst layers.^85,416−419^ PTFE is nonionomeric; hence, in the absence of an AEI, a liquid alkaline electrolyte needs to be fed to provide OH^–^ conductivity. PTFE could play a role in tailoring the hydrophobicity/hydrophilicity of the catalyst layers to avoid flooding and gas blockage at critical locations. The PTFE loading needs to be sufficient to act as a binder but limited to avoid catalyst blockage and negative effects on the catalyst layer’s porosity.^420^ For FCs, many studies report on the optimal PTFE (and AEI) loading, including visualization experiments on water distribution within the MEA. However, an understanding of the effect of PTFE loading on the AEMWE performance is lacking, and studies of operando neutron scattering for water-distribution visualization could be useful.^421^ An attractive feature of PTFE is to allow electrode sintering at *T* values exceeding 300 °C, assisting in bonding the catalyst particles to the PTL and GDL, but care needs to be taken on how the catalyst-layer morphology is affected by high-temperature treatments.^422^

It can be summarized that the durability of an AEI in pure water-fed AEMWEs, especially at the anode under high operating potentials, is considered a limiting factor. Li et al.^31^ in a recent review distinguished between the durability-limiting factors for water-fed and concentrated KOH-fed AEMWEs, which among others included ionomer poisoning, ionomer detachment, and instability of the AEM. Alternative electrode-fabrication methods that exclude the use of an AEI in the catalyst layer are being reported more frequently, and such studies for AEMWE cells were recently reviewed by López-Fernández et al.^423^ The relevant AEMWE studies, which reported performance measurements for a minimum of 100 h, are listed in Table 6 and discussed further in section 7. These include reports for a unified electrode design where the catalyst layer was integrated within the GDL in a single component by means of growing the OER catayst directly on the substrate, such an Ni foam.^85,424^

**Table 6:** State-of-the-Art AEMWE Cells and Experimental Parameters^a^

	catalyst loading (mg/cm^2^)					operating conditions					
study	anode	cathode	AEI loading (wt %) anode/cathode	membrane, thickness (μm)	IEC_membrane_ (mmol/g or meq/g)	electrolyte	*j* (A/cm^2^)	*E*_cell_ (V)	time (h)	degradation rate (μV/h)	ref
1	IrO_2_, 3	Pt black, 3	F-PAE, 2.5/2.5	ATM-PP	1.7/(2.7 for F-PAE)	H_2_O	0.2	1.9	2000	350	(457)
2	Fe_*x*_Ni_*y*_OOH-20F, 4.8	Pt/C, 0.94	PAP-TP-85	PAP-TP-85, 30	2.4	H_2_O	0.2	1.63	150	560	(424)
3	Ir black, 2.7	Pt/C, 2.7	Sustainion XA-9, 5	Sustainion 37–50, 50	∼1.1	H_2_O	0.5	1.9	170	705	(428)
4	IrO_2_, 2.6	Pt black (HiSPEC 1000 TM), 2.4	A-Radel, 22/27	A-201 Tokuyama, 28	2.0	H_2_O	0.2	1.8	535	747	(83)
5	Ni_2_Fe_3_, 3	PtRu/C, 2	TMA-53, 20/20	HTMA-DAPP, 26	2.6/(2.6 for TMA-53)	H_2_O	0.2	1.75	170	1470	(414)
6	IrO_2_, 0.6	Pt/C, 0.3	BPN	quaternized polyphenylene	n/a^b^	H_2_O	0.2	2.1	100	1800	(415)
7	IrO_2_, 1.2	Pt/C, 0.3	PFOTFPh-TMA-C8, 25 wt %	PFOTFPh-TMA-C8, 23	2.7	H_2_O	200	1.65	120	2250	(458)
8	IrO_2_, 2.3–2.8	Pt black, 2.3–2.8	PiperION (TP-85), 10	PiperION (TP-85), 50	2.02–2.37	H_2_O	0.5	1.85	175	2571	(465)
9	Ni–Fe–O_*x*_, 5	Ni–Fe–Co, 5	Sustainion XB-7	PBI-based AEM (KOH doped)	n/a^b^	1 M KOH	1	2.09	100	–100	(461)
10a	NiFe_2_O_4_, 1.8	modified Raney nickel, 14.5	Nafion^c^	Sustainion grade T^d^, 50–80	∼1.1	1 M KOH	1	1.83	1920	0.7 ± 0.2	(453)
10b	NiFe_2_O_4_, 1.8	modified Raney nickel, 2.7	Nafion^c^	Sustainion X37-50, 50–80	∼1.1	1 M KOH	1	1.85	12180, 10100	0.7 ± 0.2	(453)
11	Raney Ni–Fe	Raney Ni–Fe	no AEI	PFTP-13 AEM, ∼30	2.82	1 M KOH	0.5	∼1.5	1000	±close to 0	(463)
12	NiFeOOH, electrodeposited	Pt/C, 0.4	Fumion, 30 (cathode only)	FAA-3, 50	1.4–1.6	1 M KOH	0.5	∼1.6	100	±close to 0	(108)
13	NiFeO_*x*_, 2	NiFeCo, 3	Nafion^c^	Sustainion 37–50, 50	∼1.1	1 M KOH	1	1.9	1920	5	(462)
14	IrO_2_, 1.2	Pt/C, 0.3	PFOTFPh-TMA-C8, 25 wt %	PFOTFPh-TMA-C8, 53	2.7	1 M KOH	200	1.6	135	370	(458)
15	IrO_2_, 2	Pt/C, 0.5	PFTP, 10/PFBP, 25	PFTP-13 AEM, ∼30	2.82	1 M KOH	0.5	1.6	1100	455	(463)
16	NiAl, 47.9 (plasma sprayed)	NiAlMo, 42.7 (plasma sprayed)	no AEI	HMT-PMBI, 50	2.52	1 M KOH	1	2.05	145	650	(464)
17	Raney Ni–Fe, 20	Raney Ni–Fe, 20	no AEI	Sustainion 37–50, 50	∼1.1	1 M KOH	0.5	∼1.93	250	1200	(463)
18	NiCo_2_O_4_, 8	Pt, 0.3	qPPO, 90/10 (catalyst/ionomer)	Dowex Marathon A and LDPE blend^e^	2.45	1.95 M KOH	0.3	1.8	100	200	(466)
19	Ni foam^f^	Ni foam^f^		PSEBS-CM-DABCO, 100	0.76	1.95 M KOH	0.3	2.26	160	20	(467)
20	NiCo_2_O_4_, 8	Pt, 0.3	qPPO, 90/10 (catalyst/polymer)	qPPO, TMA quaternized, 200	1.46	1.95 M KOH	0.3	1.8	400	100	(468)
21	NiCo_2_O_4_, 8	Pt, 0.3	PSEBS-CM-TMA/PTFE, ∼5 wt % or 95/5	PSEBS-CM-TMA, 100	0.75	1.95 M KOH	0.3	1.76	800	50	(469)
22	CuCoO_*x*_ (Acta 3030), 36	Ni/(CeO_2_–La_2_O_3_)/C (Acta 4030), 7.4	PTFE^c^, 10/10	A-201 Tokuyama, 28	1.8	1 wt % K_2_CO_3_	0.47	1.85	1000	200	(84)
23	Ni_0.7_Co_0.3_O_*x*,_ 2	Pt/C (40 wt %), 1	AS-4, 5	in-house prepared APE^g^	n/a^b^	1 wt % KHCO_3_	0.1	2.03	550	200	(470)
24a	CuCoO_*x*_ (Acta 3030), ∼30	Ni/(CeO_2_–La_2_O_3_)/C (Acta 4030), ∼7.4	alkaline ionomer (I_2_, Acta Spa), ∼9	A-201 Tokuyama, 28	1.8	1 wt % K_2_CO_3_	0.5	1.98	200	500	(235)
24b	CuCoO_*x*_ (Acta 3030), ∼30	Ni/(CeO_2_–La_2_O_3_)/C (Acta 4030), ∼7.4	alkaline ionomer (I_2_, Acta Spa), ∼9	FAA-3-PP-75	1.4–1.6	1 wt % K_2_CO_3_	0.5	2.05	200	2380	(235)

a All AEMWE cells were run for at least 100 h. Additional experimental conditions are given in Table S20.

b n/a indicates that information was not available in publication or corresponding Supporting Information. F-PAE, partially fluorinated poly(arylene ether); PAP, poly(aryl piperidinium); ATM-PP, poly(phenylene) with benzylic methylammonium groups; BPN, quaternized biphenylene AEI; TMA, trimethylammonium-functionalized polystyrenes; HTMA-DAPP, hexamethyltrimethylammonium-functionalized Diels–Alder polyphenylene; qPPO, quaternized polyphenylene oxide (with trimethylamine, TMA); PFOTFPh-TMA-C*x*, TMA-modified poly(fluorene-*alt*-tetrafluorophenylene) with *x* being the number of carbon atoms in the alkyl side chain; PFTP, poly(fluorenyl-*co*-terphenyl piperidinium-8); PFBP, poly(fluorenyl-*co*-biphenyl piperidinium-14); LDPE, low-density polyethylene; PSEBS, polystyrene-*block*-poly(ethylene-*ran*-butylene)-*block*-polystyrene, from technical data sheet.

c Nafion and PTFE may act as binders rather than AEIs’ in all of these cases, i.e., studies 6–8, OH^–^-conducting electrolytes were fed to the AEMWE cell.

d PTFE-reinforced Sustainion.

e Dowex Marathon A is milled anion-selective particles (Dow, predominant particles between 10 and 30 μm size, IEC = 3.90 mequiv/g) blended with LDPE and a water-soluble additive (3.4 wt %). They are press-molded between poly(ethylene terephthalate) (0.3 mm thick) films.

f Ni foams served as PTL and GDL; no catalyst was added onto the foams.

g No further information was supplied by the authors on the membrane other than in-house prepared solid alkaline polymer electrolyte (APE).

### 5.2 MEA Design

To form an MEA, the catalyst can be deposited directly either on the membrane, referred to as the catalyst-coated membrane (CCM) technique, or on a substrate, referred to as the catalyst-coated substrate (CCS) technique. Typical preparation methods for coating the substrate, which for AEMWEs is typically a choice of either a GDL or PTL, include wet routes whereby the catalyst powder and ionomer are mixed with a suitable solvent to create a stable ink or slurry. The latter are applied by spraying or painting onto the GDL support. These techniques, adopted from the fuel-cell field, have been optimized for PEMWEs^425^ and, more recently, for AEMWEs. To reduce waste and the use of large amounts of solvents typically associated with the wet-route MEA-fabrication methods, alternative thin-film deposition methods are being investigated. Chemical vapor deposition, atomic layer deposition, ion beam sputtering deposition, or magnetron sputtering are examples of such thin-film deposition methods.

The CCS approach allows easier control to fabricate robust and stable catalyst layers by depositing the catalyst inks and slurries directly onto an appropriate substrate. Alternatively, the CCM approach holds the benefit of improved contact of the catalyst layer with the membrane interface, resulting in improved ionic conductivity, which is seen in a decrease of the interfacial contact resistance. The main concerns are that the stability of the ionomer could be compromised and superficial changes of the membrane such as swelling can be introduced during the catalyst-deposition process. Comparisons between CCM- and CCS-fabricated MEAs reported in the literature are not simple because many factors such as the membrane stability, ionomer and membrane compatibility, and deposition technique may differ significantly.

A recent review by Miller et al.^30^ illustrated this exactly, as they showed the average current density recorded at 1.8 V^235,426^ was similar, namely, ∼200 mA/cm^2^, for CCS- and CCM-fabricated MEAs. The single-cell AEMWE performance was found to largely depend on the operating temperature, catalyst type (PGM versus non-PGM), and electrolyte for the different fabricated MEAs. Other studies have reported optimal performances with a CCM-cathode and CCS-anode electrode configuration, as the cell stability for the CCM approach was poor due to delamination of anode catalyst particles.^427^

**Figure 43:**
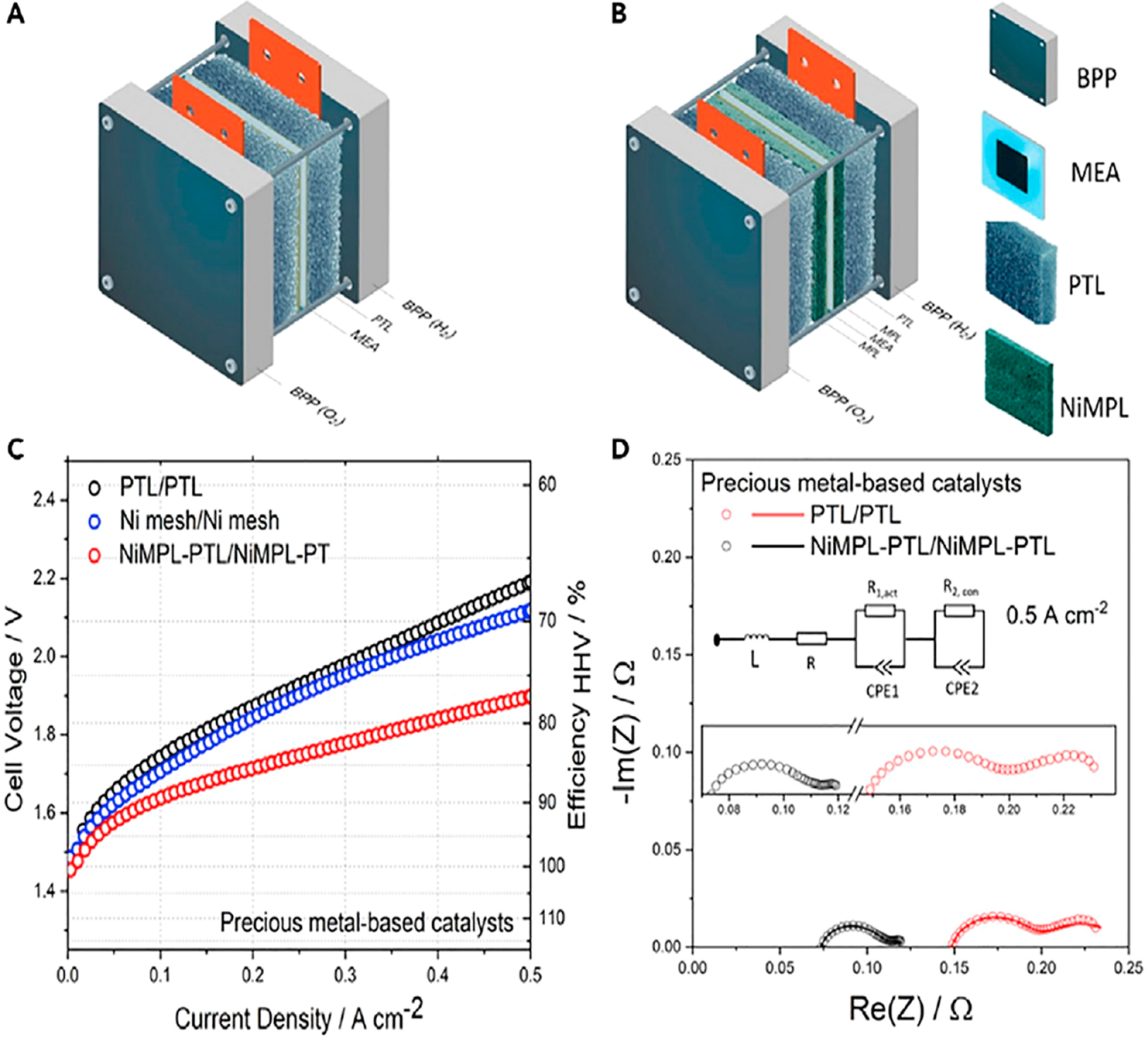
Schematics of different AEMWE cells including (A) only PTL and (B) the addition of a NiMPL-PTL on the anode and the cathode. (C) AEMWE-cell performances measured at 60 °C for water feed and for configured PTL/PTL (commercial Ni mesh) and NiMPL-PTL/NiMPL-PTL. (D) Electrochemical impedance spectroscopy measurements for the two cell configurations at 0.5 A/cm^2^ (from 50 kHz to 100 mHz). Reprinted with permission from ref (428). Copyright 2021 Elsevier.

Another strategy for reducing the interfacial contact resistance between the membrane and the CCS-formed anode is the inclusion of a microporous layer (MPL) between the PTL and MEA, as illustrated in Figure 43. Improved electrical connection and liquid/gas transport were achieved for a NiMPL-PTL while operating with a water feed.^428^

Determining the preparation parameters influencing the MEA and catalyst layers is somewhat of a trial approach due to the many variables involved. Some of the knowledge acquired for PEMWEs and FCs can be extrapolated to AEMWEs. In addition, molecular dynamic modeling of the MEA components in conjunction with experimental verification could advance the field more rapidly. In general, hot-pressing the MEA is favorable to increase the contact between the catalyst layer and the AEM, although the AEM may dry out. The *T*_g_ values of the AEM and AEI play an important role in determining the hot-pressing temperature. Control is needed to avoid AEM compression specifically for AEMWEs fed with liquids as compared to the gaseous feed of a FC, which benefits from pressures anywhere between 2 and 200 kg/cm^2^ at 120–195 °C for 50–300 s.^429^

The MEA components need to be optimized in tandem to address factors such as water management to avoid both drying out and flooding. In the case of AEMFCs, it is now believed that maximum performance cannot be achieved due to water flooding.^421^ Many of these issues have been addressed for PEM-based FCs and WEs, but in the case of alkaline conditions, the imbalance of water produced and consumed at the anode and cathode is larger than that for acidic conditions. The source of OH^–^ needed for the OER at the anode is in abundant supply when operated with a liquid alkali electrolyte as 1 M KOH, while the OER for the water-only feed depends on OH^–^ being supplied through the water splitting reaction taking place at the cathode. In the electrochemical reactions of an AEMWE, 1 mol of H_2_O is produced at the anode and 2 mol are consumed at the cathode, while for a PEMWE, 1 mol is consumed at the anode and 0 mol are consumed at the cathode. Even though H_2_O is produced at the anode and consumed at the cathode, the water feed at the anode seems to become the preferred feeding mode for AEMWEs. This mode reduces the need for H_2_O and H_2_ separation, thus delivering a higher-purity H_2_ from the cell.^430^ However, the best cell performance and highest operating current densities have been reached by feeding electrolyte to both the cathode and anode, which also reduces the risk of anode dehydration and increases water transport to the cathode.^83,107^ Future strategies to tailor defined MEA and transport-layer structures directing the liquid and gas feed to specifically defined areas are important for the design of novel and effective electrode architectures. This could include tailoring the hierarchical porosities of the catalyst layers along the in-plane (electrode-to-electrode) direction and utilizing modifiers that repel H_2_O or maybe even capture H_2_O. The optimization of flow rates and KOH concentrations are both relevant to the design of an actual AEMWE and have so far received limited attention in the literature.^430^

### 5.3 Current Collectors, Bipolar Plates, and Flow-Field Design

**Figure 44:**
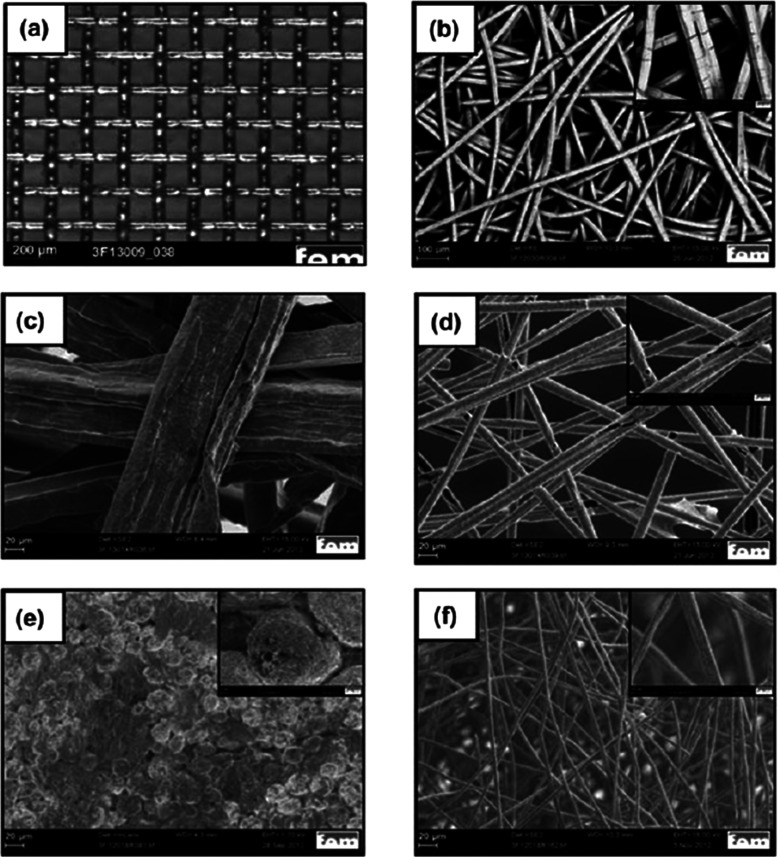
SEM and optical microscopy images for different metal substrates, as follows: (a) 0.065 mm Ni wire mesh, (b) nonwoven stainless steel, (c) nonwoven Ni, (d) nonwoven C–Ni conductive composite, (e) GDE, and (f) stainless steel web. Reprinted with permission from ref (35). Copyright 2020 Royal Society of Chemistry.

The current collector, which can be referred to as either PTL (such as porous metal framework) or GDL (such as woven carbon fibers) in an electrolyzer, serves to convey the electric current between bipolar plates and the respective anode and cathode CLs while mechanically supporting the membrane. It provides the pathway for electrolyte and reaction products between respective compartments and CLs. The support is either a fiber, foam, or woven metal network, as illustrated in Figure 44, and is designed with a large specific surface area for increased contact between the CL and membrane. Ideally it should have relatively small pore sizes (1–100 μm), a high porosity (>60%), and a thickness between 0.3 and 1 mm.^35^

At the interface between the electrodes, current collectors, and bipolar plates, contact resistances in the absence of passivation layers could lead to significant contributions to the cell resistance.^78^ Therefore, material selection and uniform contact between the former components are of high importance to ensure the long durability needed for WEs.

The thermodynamic stability of Ni foam^280,431^ and likewise of stainless steel (SS)^432,433^ felts, in combination with their ability to passivize at anodic potential in alkaline media, favor their use in AEMWE as anode substrates.^30^ However, common SS 316 is likely to leach Fe into the KOH electrolyte, specifically at elevated temperatures of 80 °C and over time.^98^ Therefore, SS 316 is likely not suitable as a long-term cell material, specifically at the anode. The carbon GDLs commonly used in FCs are restricted to use at the cathode in AEMWEs due to carbon corrosion in the presence of OH^–^ ions, which tend to operate as nucleophilic intermediates and can accelerate degradation in the highly oxidative environment of AEMWE anodes.^434^

**Figure 45:**
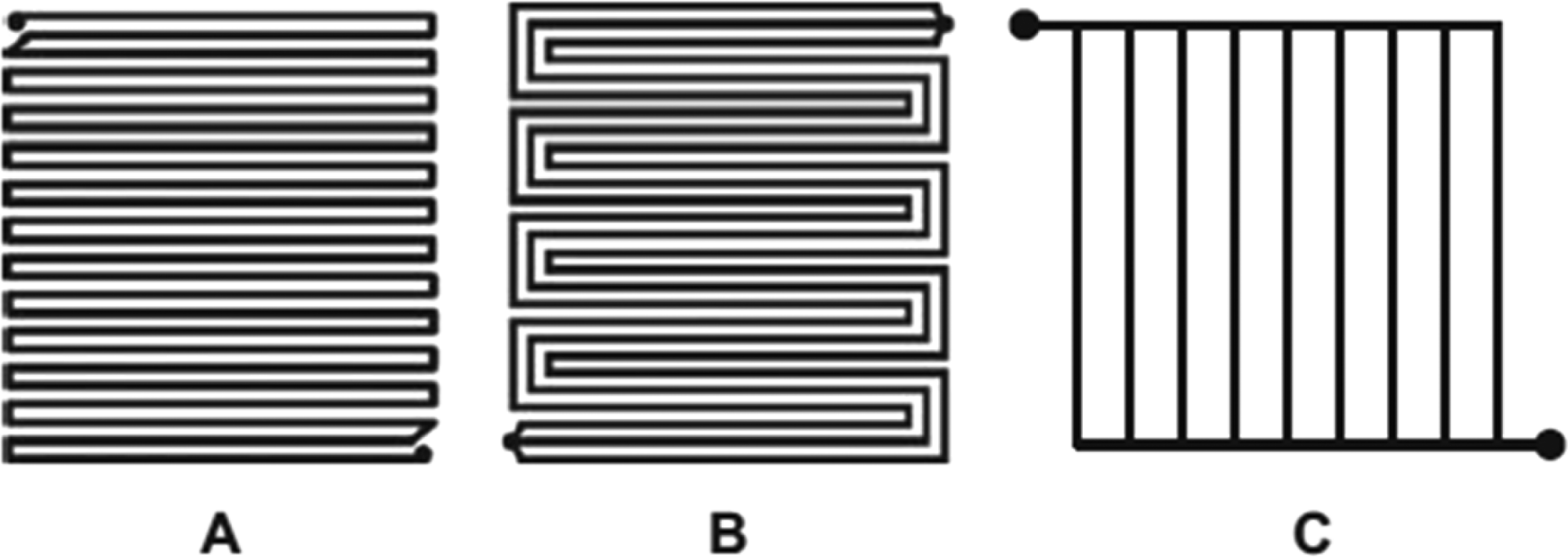
Flow-field designs commonly applied in PEMWEs: (A) single serpentine, (B) multiple serpentine, and (C) parallel column. Reprinted with permission from ref (435). Copyright 2019 Royal Society of Chemistry.

The bipolar plate’s role includes contacting cells and thus ensuring optimal reactant and product flow along a stack by means of manifolds incorporated in them. The flow-field design is closely connected to this aspect and affects the distribution of water as the reactant and the removal of produced gas and also needs to establish a firm electrical contact with the GDL and PTL. Different geometries exist, such as single and multiple serpentine, parallel column, and cascade pattern (as depicted in Figure 45), of which there is currently no optimal design.^435^ The optimal design is dependent on effective sealing of the cells for different pressures and operation at different cell sizes. Another consideration is the optimal supply of liquid water to the anode side of the cell and how this distribution effect can also serve as a temperature control of the cell or stack during operation. Most common for PEMWEs remains the use of the parallel channel design as it is proven to show a lower overpotential,^436^ although flow-distribution limitations at higher operating current densities are increasingly being investigated for WEs.^437,438^

## 6 Operational Modes and Performance

There are three operational modes in a WE. For today’s AEMWEs, the different modes are achieved at the following approximate conditions: (i) kinetic control for *j* < 0.3 A/cm^2^, (ii) cell electric resistance for 0.3 A/cm^2^ < *j* < 1.5 A/cm^2^, and (iii) mass-transport effect for *j* > 1.5 A/cm^2^. An AEMWE will operate at high *j* values, i.e., in the mass-transport zone.

A benefit of running at higher *j* values is the increase in the OH^–^ transport of the AEM due to the higher ratio of OH^–^ versus CO_3_^2–^ ions.^439^ However, the operating *j* values should always be below the critical current density (*j*_crt_), identified as the *j* value for which mass-transport losses and gas saturation limit the cell performance.^440^ Exceeding *j*_crt_ will lead to nonuniform gas distribution and nonuniform *j* values at the catalyst sites, resulting in the formation of undesired hot spots. This can result in the drying and, hence, degradation of the AEM. To limit gas-bubble formation at the surface of the electrode, limiting *j* is a good strategy. A few more suitable strategies can be considered such as releasing gas bubbles, including passive and active approaches like optimizing the catalyst layer, GDL, and PTL geometries and pore sizes;^416,441,442^ applying coatings;^417^ and the addition of surfactants.^418^ The electrolyte and reactant flow rates always need to allow fast transport, reducing the risk of creating mass-transport limitations and other failures.^72,99,100^ At higher *j* values, a higher flow rate is needed to match the consumption rate of the reactants.^440^

Electrolytes used in AEMWEs can be categorized into 3 groups: hydroxide solutions, carbonate solutions, and pure water plus the solid electrolyte. An advantage of the water-only feed is the absence of OH^–^, thus allowing the device to run at higher temperatures without a loss of the mechanical stability of the AEM.^414^ The ionic conductivity of the AEM often exceeds 0.08 S/cm, which in theory is sufficient to enable OH^–^ transport from the cathode to the anode.^35^ However, using pure water in combination with the AEI as electrolyte presents a few hurdles. This includes the need to develop stable AEIs of high ionic conductivities for neutral and mildly alkaline pH. The overall cell resistance is also higher compared to the combination of thin AEMs and liquid alkali electrolytes.

**Figure 46:**
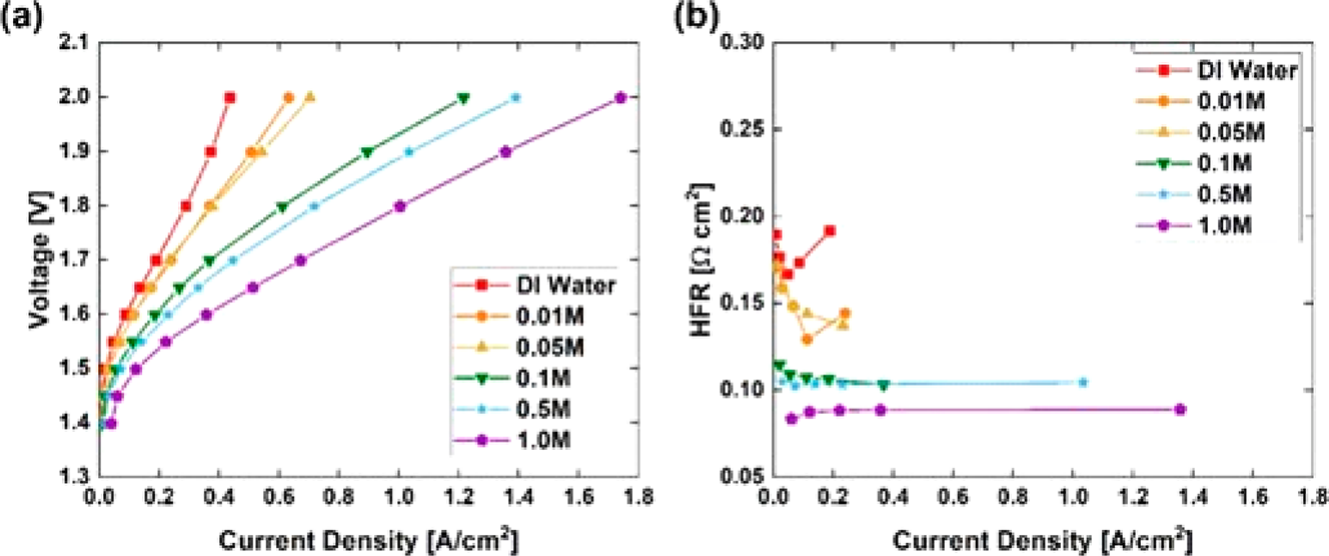
(a) Voltage (*E*_cell_) and (b) high-frequency resistance (HFR) versus *j* curves. The catalysts are at the cathode, PtRu/C 0.36 mgPt/cm^2^, and at the anode, IrO_2_ 0.75 mgIr/cm^2^. Dilute KOH or deionized (DI) water serves as the liquid electrolyte. Hexamethyltrimethylammonium-functionalized Diels–Alder polyphenylene (HTMA-DAPP) is used as the AEM and AIE. The AEM wet thickness is 50 μm. All the measurements were conducted at 60 °C and ambient pressure. Reprinted with permission from ref (446). Copyright 2021 IOP Publishing.

Diluted KOH solutions, typically between 3 and 10 wt %, are preferred to ease the nucleophilic OH^–^ attack on the AEM and AEI, but milder alkaline electrolytes are less effective in assisting in the OH^–^ transport within the catalyst layer. The KOH conductivity drops ∼1 order of magnitude from 0.178 to 0.02 S/cm when changing from 5 to 0.5 wt % KOH.^101^ For low electrolyte concentrations, there is no buffer effect; hence, rapid and undesired pH changes in the AEM and the catalyst layers can take place, and small CO_2_ concentrations dissolved in the electrolyte increase the *E*_cell_ value. Effects of the H_2_O and different alkali electrolyte concentrations on the *E*_cell_–*j* performance and also the high-frequency resistance (HFR) of single MEA cells are known.^318^Figure 46 shows the benefit of a higher KOH concentration in lowering the *E*_cell_ value. The lower *E*_cell_ values are partially due to lowering the resistance reflected in the HFR in Figure 46b but also due to additional benefits such as an increase in the effective ECSA of the catalysts using liquid alkali electrolyte feeds. Depending on the reaction order of the catalyst with respect to the pH, the catalyst kinetics can be enhanced and the catalyst stability can be altered with pH.^419,443−445^ In fact, the development of catalysts of zero reaction order may allow operation with H_2_O-only feed.^318^

**Figure 47:**
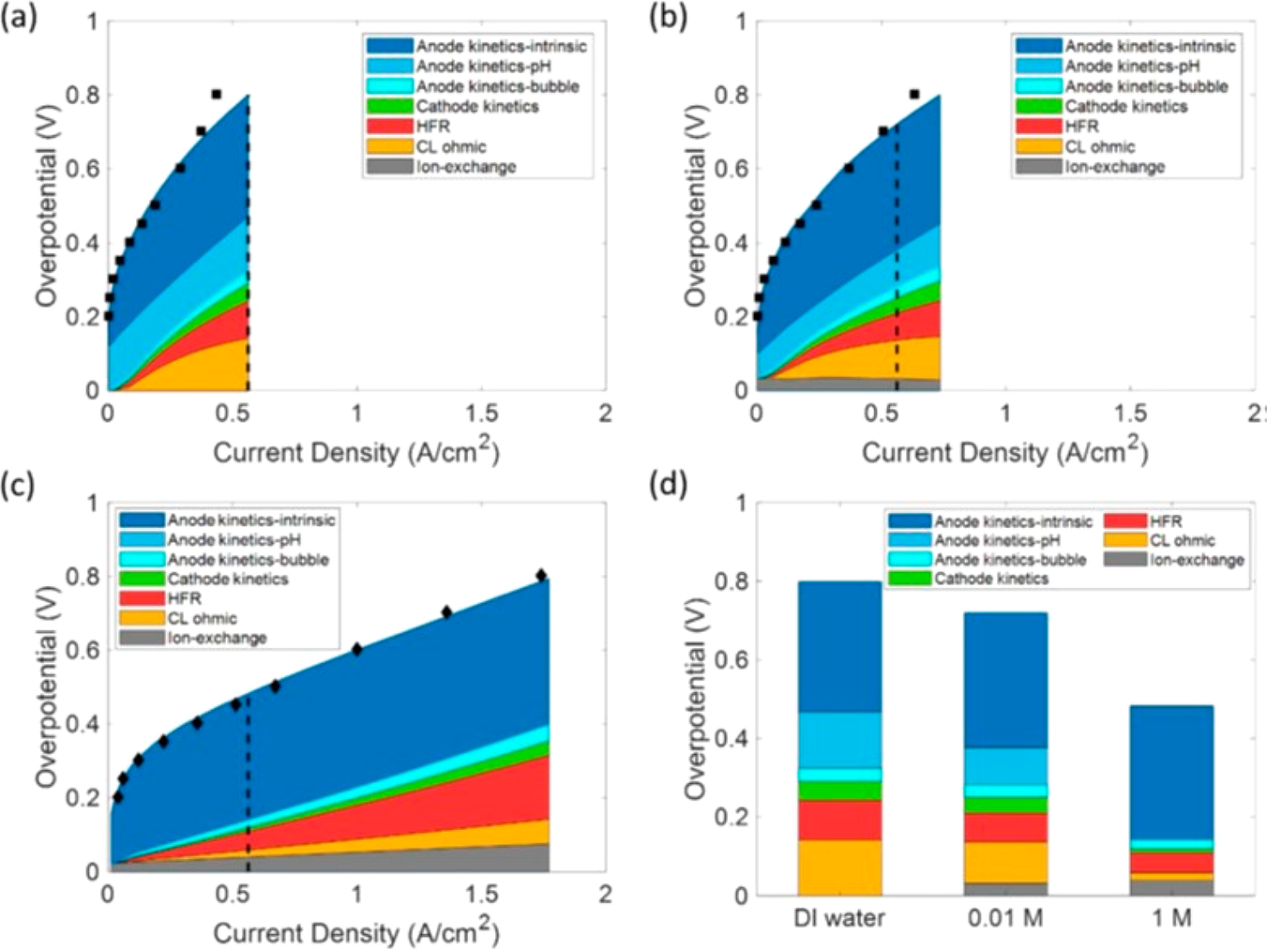
Applied voltage breakdown for (a) water, (b) 0.01 M KOH, and (c) 1 M KOH. The dashed line shows the location corresponding to the largest *j* in (a). (d) Bar graph of the applied-voltage breakdowns at 0.56 A/cm^2^ (indicated by the dashed lines). The cell overpotential is broken down into the following: anode kinetic losses (blue), cathode kinetic losses (green), high-frequency resistance (HFR) loss (red), catalyst-layer (CL) ohmic loss (yellow), and ion-exchange loss (gray). The anode kinetic losses are further broken down into three parts: anode kinetic losses due to gas-bubble coverage (light blue), anode kinetic losses due to low pH (medium blue), and intrinsic kinetics loss (dark blue). Reprinted with permission from ref (446). Copyright 2021 IOP Publishing.

Figure 47 shows the breakdown for the individual overpotential components depending on the electrolyte concentration. Figure 47d shows the enhancement of the anode and cathode kinetics and the decrease in the HFR for higher KOH concentrations.

There is a threshold for which an increase in the catalyst loading leads to a decrease in conductivity due to the resistance to mass transport created by the catalyst-layer thickness.^318^ Mass-transport limitations are prominent at higher loadings and when operating at high *j* values. The short-term *E*_cell_ versus *j* curves in Figures 46 and 47 were obtained for relatively low catalyst loadings, specifically for the anode catalyst layer. Anode catalyst loadings of 2 mg/cm^2^ are more typical, and depending on the catalyst, loadings as high as 10 mg/cm^2^ have been employed.

The (Bi)carbonate solutions pH values range between neutral and pH 12, and they have been used as an alternative to NaOH or KOH feeds. The conductivity of a (bi)carbonate solution compared with KOH is lower; thus, more-concentrated electrolytes are often employed. For example, a comparison is made of the *E*_cell_–*j* and *R*_cell_–*j* of two identical cells, both fed with electrolytes of pH ∼12, but with one being 0.01 M KOH and the other being 0.72 M K_2_CO_3_; resistances of 0.3–0.4 Ω/cm^2^ and 0.1–0.2 Ω/cm^2^ were measured.^447^ Dilute (bi)carbonate electrolytes may reduce AEM and AEI stability issues, but the site blockage through carbonate deposition remains a problem and the long-term impact on catalyst utilization (and membrane blockage) needs to be determined.^30^ This is supported by a mathematical model, which suggests that the increased voltages result from the Nernstian voltage difference across the AEM (and also AEI) when OH^–^ is replaced with CO_3_^–^ rather than from a less-conductive K_2_CO_3_ electrolyte.^448^

The inclusion of a solid-state membrane in the AEMWE also has the benefit of allowing pressurization of the cathode and compression of the H_2_, up to 30 bar.^449^ This facilitates hydrogen storage as compared to the traditional alkaline electrolysis.^59^ Additionally, the separation of electrodes by means of the AEM ensures that H_2_ crossover from the cathode to the anode is limited and the risk of forming explosive gas mixtures (>4% of H_2_ in O_2_) is reduced.^450,451^ Factors such as operating current, membrane thickness, pressure, and temperature influence the gas crossover, which, if increased, affects the efficiency of the cell negatively and leads to H_2_ loss. It is known that for higher current densities and thicker membranes the gas crossover decreases.^78^ However, the H_2_ crossover increases linearly with an increase in the H_2_ partial pressure and could become an issue for pressured systems in the operating range of 30–60 bar.^35^ Studies reporting H_2_ crossover measurements include one by Ito et al.^452^ carried out at 8.5 bar using a Tokyama A201 as the AEM and Pt/C and CuCoO_*x*_ cathode and anode catalysts, respectively. The Ito et al. study showed that the H_2_ crossover was 0.16 times that of a PEMWE system. Motealleh et al.^453^ performed a long-term study at atmospheric pressure and were able to decrease the H_2_ crossover by 56% by reinforcing a Sustainion membrane with 2% zirconia.

Limited by long-term stability, typically low-temperature PEMWEs and AEMWEs operate below 80 °C. A recent review by Lohmann-Richters et al.^454^ considered the challenges to increase the operating temperature for AEMWEs in order to increase the current density and the possibilities to capitalize on this unique advantage by means of thermal management. Thus far, it has been demonstrated that a performance with current densities of 3.75 A/cm^2^ at 1.75 V and 200 °C is obtainable by the implementation of mixed Ni, Fe, and/or Co and Raney-Ni–Mo as anode and cathode catalysts, respectively, albeit with porous zirconia as a diaphragm. It is concluded that further improvements with regards to components costs and possibly stability would be required to advance the latter technology.

## 7 Single-Cell AEMWEs Exceeding 100 h of Operation

Optimization of single AEMWE cells has resulted in significant stability and activity improvements in less than a decade. A comparison of the most-significant AEMWE single-cell performance and durability limiting factors for different operation modes (varying liquid electrolytes) using both PGM and PGM-free catalysts was provided by Li et al.^31^ To date, high current densities in the several A/cm^2^ range have been reported, e.g., 5.3 A/cm^2^ at 1.8 V in 1 M NaOH and 2.7 A/cm^2^ at 1.8 V in pure-water feed.^414^ These current densities and *E*_cell_ values are comparable to the single-cell results of PEMWEs. However, the biggest challenge remaining is achieving good durability at sufficiently high current density (most work currently aims for 1 A/cm^2^) to achieve acceptable *E*_cell_ values below 1.9 V for run times >100 h. Until recently, only a few studies have reported AEMWE single-cell measurements exceeding 100 h. These studies are listed in Table 6 and grouped in the order of the electrolyte, first for water and then for liquid alkali electrolytes, followed by the voltage-degradation rate (μV/h) in increasing order. Additional information is given in Table S20.

Due to the many different conditions such as catalyst, catalyst loading, AEI, AEM, and electrolyte, a direct comparison is not straightforward, but a number of observations can be made. On average, AEMWE cells operated on a water feed show a higher voltage degradation than liquid alkali electrolytes when operated at higher constant current (0.2 A/cm^2^ compared to minimum 0.5 and 1 A/cm^2^ for carbonate- and hydroxide-based electrolytes, respectively).^424^ From the eight durability studies found that operated on pure water, only two reported the use of PGM-free anodes (studies 2 and 5, Table 6), while all of the others include the use of baseline state-of-the-art (SoA) PEMWE catalysts, IrO_2_ and Pt/C. Both observations may be related to the fact that the pH in an AEMWE run on pure water is close to neutral or perhaps even drops below 7 in the anode catalyst layer. The pH drop is due to the consumption of OH^–^ during the OER, possibly facilitating degradation of the anode catalysts such as NiFe.^455,456^ Furthermore, high IECs of the AEIs likely provide a higher-pH environment, benefiting the OER kinetics. However, high IECs also lead to a higher water uptake, which can result in the detachment of the catalysts and, hence, higher degradation rates (e.g., studies 4, 5, 7, and 8).

The goal of studies 1 and 6 was to understand the cell durability and performance by studying different cation-functionalized polyaromatic AEMs and AEIs.^415,457^ The AEM [made using poly(phenylene) as the backbone of the AEP with benzylic methylammonium groups (ATM-PP)] of study 1 shows only a gradual cell-performance loss. This suggests that the backbone degradation of AEM and AEI is delayed by slowing the cation degradation when using benzyltrimethylammonium (BTMA)-functionalized polyaromatics (study 1^457^). However, this AEM needs to be operated in the absence of caustic solutions and below 60 °C to limit backbone degradation.^457^ The tendency of phenyl groups in the AEP backbone to be oxidized under OER potentials has been found to detrimentally influence the performance by forming acidic phenols at the anode, as determined in study 5 for the quaternized biphenylene ionomer (BPN).^414^ The phenol-formation rate of unsubstituted phenyl groups was found to be much higher in comparison to ammonium-substituted phenyl groups.^457^ More recently, Soni et al. (study 7) demonstrated that an acceptable durability with high IEC (2.9 mequiv/g) can be achieved by introducing long alkyl side chains (C_*x*_, *x* = 8) to the AEP backbone and effectively reducing the phenyl fraction that is susceptible to electrochemical oxidation.^411,458^ In addition, the poly(fluorene-*alt*-tetrafluorophenylene) (PFOTFPh) polymer backbone contains nonrotatable fluorene moieties, which are less likely to be absorbed on the Pt catalyst surface and therefore further serve to suppress phenyl absorption of the AEP backbone.^410^ The same PFOTFPh-TMA-C8 (only thicker membrane, 53 μm) prepared MEA in 1 M KOH (study 13) approached near 100% in faradaic efficiency due to a lower measured H_2_ permeability (8 times lower as compared with Nafion 211) and only 120 mV higher for current densities measured up to 2 A/cm^2^.

In study 5, 0.2 A/cm^2^ at 1.75 V in H_2_O was achieved.^414^ However, the large voltage degradation indicates limitations. It was found that, even in the presence of a high IEC ionomer (TMA-70, IEC = 3.3 mequiv/g), the non-PGM anode catalyst particles were washed out. Exchanging the AEI with a lower IEC TMA-53 (IEC = 2.6 mequiv/g) showed an increased binding strength for long-term operation. The combination of the same IEC (2.6 mequiv/g) AEIs and AEMs (HTMA-DAPP AEM and TMA-53 ionomer) and, hence, similar swelling proved to be more stable in cell tests.^414^

Xiao et al. (study 2) demonstrated the advantages obtainable with a non-PGM, self-supported, fluoride-incorporated nickel–iron oxy/hydroxide (Fe_*x*_Ni_*y*_OOH-nF) catalyst directly grown onto a compressed Ni foam support.^424^ Increased catalyst utilization and an improved contact between the catalyst, PTL, and membrane ensured better long-term durability.

Razmjooei et al.^428^ (study 3) demonstrated that, by introducing a nickel-based microporous layer (MPL), an improved contact at the interface between MEA and PTL resulted in a reduced ohmic resistance and ultimately improved cell performance with Sustainion (IEC of ±1.1 mmol/g). Furthermore, an MPL designed with the appropriate pore size and distribution could serve to decrease mass-transport losses and enable operating AEMWEs at higher current densities (comparable to a PEMWE).^459,460^

Vincent et al.^461^ (study 9) demonstrated cell performances of 2.09 V at 1 A/cm^2^ (1.88 V at *j* = 0.6 A/cm^2^) in 1 M KOH at 60 °C. The maintained *E*_cell_ (first 100 h) compares well with that of study 12, which also used a Ni-based catalyst in the anode and cathode and 1 M KOH at 60 °C but with Sustainion as the AEI in the catalyst layers and an in-house prepared PBI AEM. Detailed information about the preparation or thickness of the AEM itself is not given, but the work illustrates the successful preparation of a low-cost Ni-based catalyst with apparent high stability and performance.

The most impressive performances regarding low voltage degradation have been achieved by PGM-catalyst-free cells implementing the use of a Sustainion membrane (studies 10a, 10b, and 13).^453,462^ According to data extracted from a peer-reviewed technical report published by Dioxide Materials, these membranes show by far the lowest degradation, indicated in the voltage increase of only 5 μV/h (study 13), although a 1 M KOH electrolyte was circulated to achieve an *E*_cell_ of 1.9 V at a *j* of 1 A/cm^2^.^462^ The Sustainion membrane is based on a polystyrene-based membrane with a quaternized imidazolium headgroup. This membrane is argued to achieve its high OH^–^ conductivity through the K^+^ of the electrolyte in combination with its high water uptake; hence, it is suggested that it is not an actual AEM.^32^ The small degradation rate of the Sustainion membrane was confirmed by others (study 10).^453^ In fact, the latter group reported the longest-stability single-cell AEMWE performances to date of over 10 000 and 12 000 h for Sustainion XC37-50 and grade-T membranes (a PTFE- reinforced membrane), respectively, in 1 M KOH and at 1 A/cm^2^.^453^

The MEAs, using commercial Tokuyama and Sustainion membranes, employed either Nafion or PTFE in the catalyst layer, i.e., no actual AEIs, indicating that the OH^–^ conductivity in the catalyst layers is provided by the alkali electrolyte feed. Chen et al.^463^ achieved a record density for AEMWEs of 7.68 A/cm^2^ at 2 V with 1 M KOH at 60 °C by using PGM catalysts (study 15). By using the same high IEC AEM (>2.8 mmol/g), namely, poly(fluorenyl-*co*-terphenyl piperidinium-13) (PFTP-13), and Ni–Fe composite catalysts (study 11), a current density of 1.62 A/cm^2^ at 2 V was measured in 1 M KOH. More importantly, the AEMWE study was based on running a dry cathode for applications where high-purity hydrogen is of importance. The authors found that an AEM with high IEC (∼2.8 mmol/g) and diffusivity (9–11 × 10 ^–8^ cm^2^/s) was required for ensuring the high cell performance with only an anode feed. Furthermore, including a high-IEC ionomer (3.43 mmol/g) in the cathode electrode (25 wt % loading) had a subsequent high water uptake. This serves to secure water molecules diffusing through the AEM from the anode to the cathode electrode for electrochemical reactions. The durability of PGM-free electrodes, Ni–Fe/Ni foam, and PFTP AEM (study 15) demonstrated superb stability at an applied current 0.5 A/cm^2^ in 1 M KOH and can be regarded as one of the best-performing and durable AEMWE single-cell results so far along with commercial Sustainion (studies 10 and 13).

Another ultrahigh current density of 4 A/cm^2^ for 12 h measuring a stable cell voltage of ∼2.05 V was reported by Park et al.^107^ At 0.5 A/cm^2^ (study 12) the measured cell voltage showed not even a slight decrease over the 100 h measurement. The authors demonstrated an improved (2-fold) and durable performance of their in-house prepared three-dimensional unified electrode whereby the catalyst NiFeOOH was directly formed by electrodeposition onto the current collector as compared to conventional electrodes (0.5 mg/cm^2^ IrO_2_ anode). The atomic Fe/Ni ratio was optimized (3:3) to obtain an optimum balance between catalytic activity and electrical conductivity, which resulted in an improved cell performance from the reduced ohmic resistance. Beyond the enhanced catalyst utilization, the larger pore-size distribution of the unified electrode design also serves to improve the mass transport of the reactant and product.

Another longer-term study (study 16) employing electrodes without the addition of an AEI was presented by Wang et al.^464^ They managed to obtain a performance comparable to that of a PEM electrolyzer at 60 °C (1 A/cm^2^ at 1.8 V) with their atmospheric-plasma-sprayed (APS) NiAlMo layer anode, a 1 M KOH feed, and a hexamethyl-*p*-terphenyl poly(benzimidazolium) (HMT-PMBI) AEM. The performance was stable for 145 h.

Study 18 reports the use of a heterogeneous AEM, which consists of anion-exchange particles (Dowex Marathon particles, 10–30 μm, IEC of 3.9 mequiv/g) blended with a low-LDPE matrix and water-soluble additive for the purpose of increasing the conductivity of the membrane.^466^ The conductivity of the heterogeneous membrane was improved by 75% for an optimized water-soluble additive content of 3.4 wt %. However, this performance is limited to the use of a liquid alkali electrolyte to ensure efficient OH^–^ transport through the membrane to provide contact between the anion-selective particles. A trimethylamine quaternized PPO was employed as the AEI in study 20.^468^ An identical MEA was tested in 1 M KOH at 70 °C, and the impact of membrane degradation (an IEC decrease of 5.7% after 100 h of operation) was observed, resulting in a more pronounced cell-voltage degradation of 400 μV/h. It was concluded that such a heterogeneous membrane’s performance highly depends on the liquid electrolyte’s conductivity, and to ensure an enhanced lifetime (>100 h) of the membrane in 1 M KOH electrolyte, a cell temperature of 50 °C could be considered limiting.

In a follow-up study by Hnát et al.^467^ (study 19), the stronger base DABCO was investigated as the quaternization agent for the chosen backbone polymer, a poly(styrene–ethylene–butylene–styrene). Increases in the operation temperature and electrolyte concentration ensured higher electrolysis efficiency but increased the degradations of the AEM and AEI. Therefore, the strategy to decrease the weak spots in the AEP structure was to lower the IEC and use 10 wt % KOH as the electrolyte. The heterogeneous membrane proved promising, albeit with a higher *E*_cell_ of 2.25 V, which was a consequence of the bare Ni foam used as a current collector and catalyst for the long-term measurements. It was concluded by the research group of Žitka et al.^469^ that the polymer matrix PPO quaternized with either DABCO or TMA eventually (after 400 h) suffered from degradation of the backbone hydrolysis mechanism and the performance deteriorated at operation temperatures of 60 °C in 10 wt % KOH. They found more promise in combining their TMA-quaternized PSEBS in both membrane and AEI (study 21), which operated for 800 h and showed a low voltage degradation.

Study 22 used a catalyst-coated-substrate preparation method using PTFE instead of an actual OH^–^-conducting AEI at the anode.^84^ The cell was stable for 1000 h at 0.47 A/cm^2^ on a 1 wt % K_2_CO_3_ feed.

Chi et al.^470^ (study 23) focused on evaluating the durability of nickel/cobalt oxide as non-PGM and compared the cell performance with commercial Acta 3030 as the OER catalysts. Their prepared Ni_0.7_Co_0.3_O_*x*_ showed good stability and outperformed (0.2 versus 0.1 A/cm^2^ at 2 V) the cell with a commercial anode catalyst. Detailed information about the membrane besides that it was prepared in-house were not presented.

Study 24 by Vincent et al.^235^ also reported the use of commercial Acta 3030 and 3040 as OER and HER catalysts, respectively. Very high catalyst loadings (∼30 mg/cm^2^) were used, and it is the first study to report the use of Acta supplied ionomer (I_2_) for longer-term studies, compared to Tokoyama (24a) and (24b) FAA-3-PP-75 membranes in 1 wt % K_2_CO_3_ at 60 °C.

## 8 Establishing Protocols for Single-Cell AEMWE Evaluation

Electrochemical characterization of suitable AEMWE materials in full cells typically entails performance and durability measurements. Due to varying operating conditions, care needs to be taken when comparing the AEMWE cell performances. The comparison of AEMWE test results remains difficult due to the absence of uniform testing protocols.^101^ Typical reports of high-performing AEMWE cells include short-term tests recording slow-sweep polarization (*j*–*E*_cell_) curves. The recording of *j*–*E*_cell_ curves is most often preceded by a break-in procedure that serves to activate the catalyst layer and conditions the AEM. This procedure may vary depending on the AEM and catalyst used. Such a break-in typically entails applying a constant *E*_cell_ in the range of 1.6 and 2.2 V,^35,98,471^ which is incrementally (in 0.2 V steps) increased for varying holding times to reach pseudostable current densities at the applied *E*_cell_. In other cases, slow-sweep *j*–*E*_cell_ curves are repeatedly recorded until pseudostable curves are obtained.

Short-term tests can show promising results, but the durability of the cell and the components require a longer evaluation (>1000 h) and even intermittent tests to allow for a dynamic durability assessment. So far, the long-term tests vary between operating at a constant *j* of 0.5 or 1 A/cm^2^ and a few intermittent-mode studies entailing cycling between a high and low (e.g., between 1 and 1.8 V) *E*_cell_ limit.^84,472^ Frensch et al.^473^ discussed the limits of past AEMWE durability studies typically conducted under continuous feed at constant *j* or by cycling within narrow *E*_cell_ ranges. They suggested a dynamic evaluation of the stability for ∼1000 h. A voltage bias of 1.95 V was applied by interrupting the steady operation at varying time intervals (such as every 2–10 h and every 100 h). The effect on the cell performance was evaluated from electrochemical impedance spectroscopy (EIS) data recorded before and after each rest period. The frequency of the rest times was found to significantly affect the cell stability, and recoverable and irreversible losses associated with either nonpermanent gas-bubble formation or AEM degradation (likely due to drying during more frequent rest times) were distinguished.

There remains the need to properly define protocols for full-cell stability tests while keeping the end application in mind (e.g., intermittent operation when coupled with renewable energy storage). The inclusion of characterization techniques such as high-frequency resistance (HFR) or full EIS in support of *j*–*E*_cell_ curves allows for a breakdown of the associated (kinetic, ohmic, and mass-transport region) losses and identifies the limitations of the AEM, interfaces, catalyst layers, and other MEA characteristics. Slow-sweep voltammetry yields information on the catalytic activity specifically when studied in half-cell MEAs. Floating electrode and gas diffusion electrode (GDE) half-cell setups have been developed. The floating electrode best suits electrochemical reactions with gas as the reactant but does not allow one to change parameters such as temperature, pressure, and feed flow rate.^474^ The GDE half-cell setup seems more suitable for comparing catalyst activity in an AEM or BPM system, and parameters such as feed flow rate, *T*, and *P*, as well as different components such as PTL, GDL, AEI, and catalyst loading, can be tuned to mimic the real system.^475−477^ On the basis of this information, a testing protocol for the evaluation of single-cell AEMWEs is proposed in the Supporting Information.

## 9 Developments on AEMWE Stack Designs

Operational modes of single AEMWE cells will need to be tuned to optimize the performance of AEMWE stacks. An initial AEMWE stack study by Bouzek and co-workers^430^ was aimed at verifying suitable MEA design parameters in order to ensure that high-purity product gases were produced with their developed gas-separator system. A 3-cell stack consisting of 5 × 5 cm^2^ nickel foam electrodes (no catalyst was added) and the commercial heterogeneous anion-selective membrane Ralex reinforced with PP mesh (Mega, Czech Republic) was studied. To decrease the oxygen contamination due to H_2_ crossover, the electrolyte feed arrangement was adjusted to feed electrolyte to the anode compartment only. This allowed for water molecules to diffuse from the anode to the cathode due to the membrane’s hydrophilic nature while facilitating the pressurization of the H_2_ produced at the cathode without the need for separation from the liquid phase. In addition, a sufficiently high pH at the anode was maintained withstanding dilution from produced water, thus preventing the dissolution of the Ni electrode, which can take place upon positive electrode polarization at pH < 9.^478^ The authors developed a mathematical model allowing for stack-performance validation, thus aiding in the scale-up of future zero-gap stack developments and the understanding of the impact of limiting factors.

Apart from the AEMWE single-cell studies, studies of commercial-size stack operation of active areas exceeding 60 cm^2^ in the water electrolysis literature are few.^479^ The first study thus far to apply their own developed electrocatalysts to an AEMWE stack system was recently reported by Park et al.^480^ They demonstrated the activity and durability of their own developed NiCoO and NiCo alloys as the HER and CuCoO as the OER electrocatalysts at a commercial scale. The catalysts were incorporated in a 5-cell AEMWE stack system (active area of 64 cm^2^). Interestingly, their 5-cell stack performed better than a similar material’s single-cell measurement, achieving 740 mA/cm^2^ at 1.65 V per cell as compared to the measured 540 mA/cm^2^ at 1.85 V cell voltage for the single AEMWE cell. It is important to understand the influence of the electrolyte behavior as the stacks increase in number and size and the impact of design that allows for optimum laminar and turbulent flow of electrolyte, which directly affects the cell performance.^481^ Attention on fluid mechanical analysis to better understand and further improve performance remains an important topic as the AEMWE technology develops. Furthermore, cell degradation for the 5-cell stack was measured at a voltage degradation per stack of 2 mV/h with an initial cell efficiency calculated at 69% and a H_2_ purity of 99.995% measured by gas chromatography.

## 10 Summary and Outlook

Much progress in AEMWE single-cell performance has been made, and PGM-free anodes are being incorporated. AEMWE cells frequently approach *j* values exceeding 1 A/cm^2^ that are needed for large-scale applications, and one long-term study has shown a high durability in combination with a low cell voltage (<2 V) for 10 000 h. Concrete conclusions regarding the most promising compositions of an MEA cannot be made at this point due to the many different variables and inconsistent evaluation protocols used. However, higher performances for cells run on dilute alkali electrolytes rather than H_2_O and equipped with PGM-free anodes are observed. The dilute alkaline-fed cells (1 M KOH) in combination with PGM-free anodes have shown improvements in the stability (voltage-degradation rates of ≤5 μV/h were observed) for minimal measurement times of 1000 h.^453−463^ The stability of MEA components, specifically the catalysts and AEMs, are still limiting the widespread implementation of AEMWEs. To increase the stability, it is key to understand the degradation mechanism employing a diverse range of characterization techniques. The development of in situ characterization techniques to identify the mechanism leading to polymer degradation, including in situ studies at higher temperatures, would be helpful. This includes the need to understand the interactions between the catalysts with the AEM and AEIs. The in situ identification of chemical and physical changes specifically for the sluggish anode catalysts when operating at high *j* values and *E*_cell_ values would also be of value.^415^

Changes in the *R*_cell_ and, hence, also the *E*_cell_ value over time need to be understood. Many factors can increase *R*_cell_ including blockage by H_2_ and O_2_, carbonate deposition, and corrosion of the current collectors. The overall *R*_cell_ value can be measured in situ using HFR,^83^ but a resistance increase can originate from many sources, making it difficult, but necessary, to decouple the various sources. EIS could provide insight into the ohmic resistance of the AEM as well as monitoring in situ changes taking place in, e.g., catalyst-layer characteristics such as the ionic conductivity and catalytic activities.

The alkaline stability (specifically at >60 °C) is still a concern for AEMs. Systematic investigation into different microphase-separated structures at the molecular level, prediction of species transport, and water solvation within microphase-separated materials by multiscale molecular simulation will assist in moving the field of AEM design forward.^284^ The peralkylammoniums will likely continue to be among the most studied and stable classes of cations, while all-carbon backbones, aryl ether-free, and PBI structure will likely continue as a research focus. The materials’ processability and cost will need to be considered. More extensive and standardized device testing is needed.^36^

The most active and stable catalysts for the HER include various types of Pt–Ni and Pt/C nanosized systems. Well-dispersed nanosized PtRu and Ru catalysts also show promise, although a full characterization of the latter, including the estimation of the Ru content and stability studies including intermittent conditions, are needed because Ru and RuO_2_ are known to show poor stability in alkaline media at more-positive potentials. It appears that much of the progress for the latter has been made by utilizing high-surface-area carbon supports featuring N-groups and layered structures to allow the dispersion (on the <3 nm scale) and the embedment of the ruthenium catalysts. Such approaches, i.e., the use of modified carbon supports other than the typically used Vulcan XC-72, may be of benefit to other catalysts to achieve a smaller particle size (in the case of, e.g., Mo-based catalysts) and to possibly avoid agglomeration of the nanosized catalyst particles during electrolysis. PGM-free catalyst options such as the combination of Ni and Mo have shown some promise. The addition of Mo to Ni has been shown to increase the HER activity over Ni-only catalysts. However, the intrinsic activities for these Ni–Mo catalysts are still below the activity of Pt/C catalysts, and the stability of Mo needs to be increased. It appears that the particle sizes of many of the PGM-free HER catalysts are larger than those for the state-of-the-art Pt/C catalysts, which in combination with lower intrinsic activities reduces the mass activity of the resulting catalysts.

The most promising OER catalysts in terms of high mass activity and a low OER onset potential include Ni–Fe-based catalysts. These catalysts typically show nanoscale features and are present as supported particles, core–shell type structures, or high-surface-area layered structures of hydrous oxides such as NiFeO_*x*_H_*y*_ and NiCoFeO_*x*_H_*y*_. Many Ni–Fe–Co catalysts have slightly higher activities than Ni–Fe catalysts, which may be related to an increase in conductivity introduced by Co. Ni–Fe–Mo-based catalysts have also been reported; however, the stability of Mo reflecting real operating conditions needs to be addressed. CoCu catalysts have attracted interest for early AEMWE single-cell studies, and the addition of Cu into the Co-oxide spinel lattice was shown to improve the intrinsic OER activity. However, mass OER activities and the performance in single AEMWE cells are not as high as for Ir-oxides and Ni–Fe-based catalysts. This could be at least partially due to a larger particle size and size distribution of the CoCu catalysts. Ir-oxides are the state-of-the-art OER catalyst in acidic electrolytes, but the stability of Ir-oxides is poorer in alkaline versus acidic media. Many different forms of Ir-oxide exist, and significant differences in the OER activity and stability are observed. The intrinsic OER activity of Ir-based catalysts follows the order Ir-metal > amorphous IrO_*x*_ > rutile IrO_2_, while the stability order is reversed, i.e., Ir-metal < amorphous IrO_*x*_ < rutile IrO_2_. More care needs to be taken when selecting baseline catalysts, such as, e.g., an Ir-oxide for the evaluation of newly developed catalysts. This includes the need for careful physical and electrochemical characterization of the baseline catalyst before and after electrocatalytic-activity measurements. Ni–Fe- and Ni–Co–Fe-based catalysts have shown higher activities than Ir-oxides in both thin-layer cells and single-cell AEMWE tests. The stabilities of these Ni–Fe- and Ni–Co–Fe-based catalysts need to be proven, but promising results have been revealed in single-cell AEMWE tests. Nevertheless, careful studies of the performance and stability of specifically the high-surface-area and hydrous NiFeO_*x*_H_*y*_ and NiCoFeO_*x*_H_*y*_ catalysts are needed that also reflect real AEMWE conditions. Studies shedding light on the stability and potential degradation mechanism of the Ni–Fe-based catalysts under AEMWE conditions will further advance this technology.

The questions of increasing the anode catalyst activity and overcoming catalyst dissolution remain. Metal dissolution of PGM catalysts has been extensively studied in a standard 3-electrode setup or using in situ detection techniques, but only a few reports for metal dissolution of PGM-free catalysts exist. To the best of our knowledge, no studies exist discussing metal dissolution in an AEMWE cell. Higher *j* values are typically applied in an MEA (as compared to thin-layer catalyst evaluation), and a catalyst experiences different conditions (such as being flooded) in a thin-layer setup versus an MEA setup.

It is difficult to compare the activities of different catalysts due to the different experimental conditions used. These include catalyst loading, catalyst surface area, measurement methods, temperature, purity and concentration of the electrolyte, and purity of the salts used for the catalyst synthesis, specifically considering Fe impurities. Comparing activities based on *j* values normalized for the geometric area or the ECSA can be unreliable if the sample is, for example, highly porous or if the normalization is carried out using different methods to measure the ECSA. However, the extraction of ECSA-related data for the catalysts being studied is needed. Unfortunately, reliable methods to determine ECSA values for oxide and/or oxide-covered catalysts do not exist. However, trends can be established using a combination of well-described electrochemical methods such as *C*_dl_, Cu_upd_, and charge values extracted from redox reactions. Catalyst stability needs to be examined more rigorously using analytical methods such as ICP-MS/OES analyses to quantify the catalyst dissolution. Uniform protocols should be used to evaluate the catalyst activity as well as the stability of the catalysts. The catalyst-stability studies also need to include measurements that consider real AEMWE conditions such as fluctuations that a catalyst can experience in the CL of an MEA as well as startup and shutdown conditions. In all cases, i.e., for activity and stability measurements, the catalysts need to be well-characterized before and after the measurements using electrochemical methods as well as physical-characterization methods. The use of steady-state measurements to extract mass and intrinsic HER and OER information and to construct a valid Tafel plot is of high importance. Currently, large discrepancies for both the HER and OER catalysts reported in the literature exist, which are likely at least partially due to using nonsteady-state methods as polarization curves.

The actual Pt amounts on the cathode in an AEMWE approach small values such as 0.5 mg Pt/C/cm^2^,^482^ and Pt–Ni alloys and potentially also Ru-based catalysts may further lower the cathode Pt loadings. Anode catalyst loadings in the 2 mg/cm^2^ and higher range are still typically used due to the sluggish OER kinetics.^482^ The use of non-PGM catalysts and possibly high-surface-area and multimetal Ni-based catalysts seems the most promising route for OER catalysts for AEMWE applications. To achieve this, as well as to achieve reductions of the OER catalyst loadings, innovative designs of the anode catalyst layer in addition to the use of highly active and stable catalysts could help. Developing novel catalyst-layer and catalyst designs that allow for efficient water and counterion transport while using highly active catalysts in small amounts seems to be an effective strategy to enhance the performance of AEMWEs.^102,483^ This might include designs similar to the thin OER catalyst-layer structure developed by 3M, which has shown promise for high performance and long-term stability for low loading of PGM anode catalysts in PEMWEs.^102^ Other CL designs explored for AEMWEs are the direct formation of the HER and OER catalysts on the porous current collector, instead of transforming catalyst powder catalysts into CLs, that subsequently need to be applied to either the porous current collector substrate or the membrane, known as CCS and CCM methods, respectively. Direct formation of catalysts on the porous and high-surface-area current collectors potentially offers a higher catalyst utilization and improved catalyst/electrode interactions. Such designs are in line with a proposal to consider a PGM-free catalyst with less sensitivity to local pH fluctuations and less interaction with the AEI. Recent studies have demonstrated promising durability advances with self-supported PGM-free electrodes and omitting the inclusion of an AEI but with a supporting electrolyte.^453,463^ Also missing are PTL and GDL studies where the effect of pore size and distribution of different support designs on mass- and charge-transport limitations are compared and optimized for AEMWEs by modeling and are validated experimentally. Besides the optimum electrode design, the AEM and AEIs still require adequate ion-exchange capacity and water diffusivity for high-performance AEMWEs.^463,483^ The earlier-mentioned considerations must be tailored for the chosen cell operating mode to ensure high performance while keeping in mind the relevant durability-limiting factors.^31^

## Supporting Information Available

Tables for HER activity measurements, tables for OER activity measurements, protocols to evaluate HER and OER catalysts, protocol to evaluate HER and OER catalyst stability, ECSA measurements for HER/OER activity reporting, summary of AEMs evaluated in single AEMWE cells, additional data for state-of-the-art single-cell AEMWE tests, and protocol for single-cell AEMWE testing abbreviations and symbols (PDF)
