# Supplementary material for: Anion-Exchange Membrane Water Electrolyzers
Source: Chem Rev. 2022 Apr 20;122(13):11830–95. doi: 10.1021/acs.chemrev.1c00854 (PMC9284563; doi:10.1021/acs.chemrev.1c00854)
Supplement: Supplementary file 1 — cr1c00854_si_001.pdf [file cr1c00854_si_001.pdf]

Supporting Information (SI):
Anion Exchange Membrane Water Electrolyzers

Naiying Du[a,d], Claudie Roy[b,d], Retha Peach[c], Matthew Turnbull[a,d], Simon Thiele[c,e], Christina Bock[a,d*]

[a]: National Research Council of Canada, 1200 Montreal Road, Ottawa, ON, Canada, K1A 0R6

[b]:National Research Council of Canada, 2620 Speakman Drive, Mississauga, ON, Canada, L5K 1B1

[c]: Forschungszentrum Jülich GmbH, Helmholtz Institute Erlangen-Nürnberg for Renewable Energy (IEK-11), Cauerstaße. 1, 91058 Erlangen, Germany

[d]: Energy, Mining and Environment Research Centre

[e]: Friedrich-Alexander-Universität Erlangen-Nürnberg, Department Chemie- und Bioingenieurwesen, Egerlandstr. 3, 91058 Erlangen, Germany

[*]: Corresponding author: Christina.Bock1@outlook.com

CONTENTS

1 TABLES FOR HER ACTIVITY MEASUREMENTS....................S2
2 TABLES FOR OER ACTIVITY MEASUREMENTS....................S9
3 PROTOCOLS TO EVALUATE HER AND OER CATALYSTS S27
3.1 Electrode Preparation....................S29
3.2 Electrolyte....................S31
3.3 Counter and Reference Electrodes S31
3.4 Activity Measurements S32
4 PROTOCOL TO EVALUATE HER AND OER CATALYST STABILITY S33
4.1 Stability Measurements....................S33
5 ECSA MEASUREMENTS FOR HER/OER ACTIVITY REPORTING S36
6 SUMMARY OF AEMS EVALUATED IN AEMWE SINGLE CELLS S38
7 ADDITIONAL DATA TO STATE OF THE ART AEMWE SINGLE CELL TESTS...S50
8 PROTOCOL FOR SINGLE CELL AEMWE TESTING S51
ABBREVIATIONS AND SYMBOLS....................S55
REFERENCES S62

1 TABLES FOR HER ACTIVITY MEASUREMENTS

Table S1. HER data for Pt/C catalysts measured in 1 M KOH

This Table contains HER data used for Figures 10 and 12 in the paper. The majority of the data were extracted from non-steady state measurements.

Catalyst	Supplier	Loading (mg_{Pt}/cm^2)	η^{1} (mV)	$j_{mass}{}^{1}$ (A/mg_{Pt})	"T.S."[2] (mV/dec)	η region[3] (mV)	Ref.
20 wt.% Pt/C	Alfa-Aesar	0.0051	110	1.961	134	75-125	[1]

20 wt.% Pt/C	Fuel Cell Store	0.01	61	1	-	-	[2]
20 wt.% Pt/C	Alfa-Aesar	0.0204	77	0.49	46	20-40	[3]
Pt/C	-	0.255	49	0.039	39	20-40	[4]
Pt/C	-	0.2	49	0.05	39	0-50	[5]
Pt/C	-	0.283	40	0.035	39	0-20	[6]
40 wt.% Pt/C	-	0.204	53	0.049	30	-	[7]
20 wt.% Pt/C	Alfa-Aesar	-	70	1.122	117	0-90	[8]
20 wt.% Pt/C	Alfa-Aesar	0.59	16	0.085	43	12-25	[9]
Pt/C	-	0.285	372	0.035	-	0-50	[10]
20 wt.% Pt/C	-	0.0216	-	0.463	-	-	[11]
20 wt.% Pt/C	Johnson-Matthew	0.0153	-	0.653	-	-	[12]
20 wt.% Pt/C	-	0.14	-	0.071	46	30-50	[13]
Pt/C	-	-	-		-	0-20	[14]
PtNW	home-made	0.0175	-	0.571	-	-	[11]

PtBP	home-made	0.0186	-	0.538	43	20-40	2

[1]: measured at 10 mA/cm$^2_{geom}$.

[2]: "T.S." stands for "Tafel-slope". The values are as reported. Quotation marks are used as the majority of the reported "Tafel-slopes" were measured at a η region, i.e., below η < RT/F as indicated in column 7 in the Table.

[3]: η region used by the authors to extract the "Tafel-slope".

Table S2. HER data for Pt-Co and Pt-Ni based catalysts measured in 1 M KOH

This Table contains HER data used for Figure 12 in the paper. The majority of the data were extracted from non-steady state measurements.

Catalyst	Loading (mg_{Pt}/cm^2)	η[1] (mV)	j_{mass}[1] (A/mg_{Pt})	j_{mass}[1,2] (A/mg_{cat})	"T.S."[3] (mV/dec)	η region[4] (mV)	Ref.
PtCo-Co/TiM	0.043	28	0.233	-	35	10-50	13
$PtCo(OH)_2$/CC	*0.39*	32	0.026	-	70	-	15
Pt NW/SL-$Ni(OH)_2$	0.016	59	0.625	-	-	-	11
Pt NP/SL-$Ni(OH)_2$	0.0218	78	0.459	-	-	-	11
5Pt/Ni-SP	0.204	32	0.049	-	30	-	7

Pt_3Ni_3 NWs/C-air	0.015	40	0.667	0.512	-	-	[12]
Pt_3Ni_3 NWs/C-air[5]	0.015	45	0.667	0.512	-	-	[12]
Pt_3Ni_2-NWs-S/C	0.015	42	0.667	0.512	-	-	[16]
Pt_3Ni_2-NWs-S/C[5]	0.015	42	0.667	0.512	-	-	[16]
hcp-Pt-Ni alloys	0.00764	65	1.309	-	78	0-70	[8]
PtNi-O	0.0051	40	1.961	-	79	60-100	[1]
PtNi	0.0051	42	1.961	-	85	50-80	[1]

[1]: measured at 10 mA/cm^2_{geom}.

[2]: j_{mass} per total catalyst mass, i.e., A/mg_{cat}

[3]: “T.S.” stands for “Tafel-slope”. The values are as reported. Quotation marks are used as the majority of the reported “Tafel-slopes” were measured at a η region, i.e., below $\eta < RT/F$ as indicated in column 7 in the Table.

[4]: η region used by the authors to extract the “Tafel-slope”.

[5]: 0.1 M instead of 1 M KOH was used.

Table S3. HER data for Pt/C and Pt-Ni catalysts measured in 1 M KOH and at η of 70 mV

This Table contains HER data used for Figure 12b in the paper. The majority of the data were extracted from non-steady state measurements.

Catalyst	Supplier	j_{mass} (A/m_{Pt})	j_{int} (A/cm^2_{Pt})	ECSA (m^2/g_{Pt})	Ref.
20 wt.% Pt/C	Alfa-Aesar	52.5	0.28	70.9	[1]
Pt/NW	home-made	40.7	0.46	40.7	[11]
Pt_3Ni_3 NWs/C-air	home-made	27.6	3	22.8	[12]
Pt_3Ni_2-NWs-S/C	home-made	22.8	1.1	27.6	[12]
hcp-Pt-Ni alloys	home-made	27.3	11.1	27.3	[8]
PtNi-O	home-made	49.4	14.8	48.8	[1]
PtNi	home-made	48.8	10.8	49.4	[1]

Table S4. HER data for Ru-based catalysts measured in 1 M KOH

This Table contains HER data used for Figure 13 in the paper. The majority of the data were extracted from non-steady state measurements.

Catalyst	Loading (mg_{PGM}/cm^2_{geom})	Loading (mg_{cat}/cm^2_{geom})	η^{1} (mV)	j_{mass}^{1} (A/cm^2_{geom})	"T.S."[2] (mV/dec)	η region[3] (mV)	Ref.
$Pt_1Ru_{1.54}$ NP/BP	0.0148	-	22	0.676	19	10-20	[2]

20 wt.% PtRu (Fuel Cell Store)	0.01	-	40	0.2	-	-	[3]
Ru@CN	-	0.273	32	0.037	53	-	[17]
Ru_2P	-	0.233	52	0.043	69	-	[18]
$Ru@C_2N$	-	0.285	17	0.035	38	0-50	[10]
Ru/C-300	-	0.59	14	0.017	33	-	[9]
Ru-NC-700	-	0.2	12	0.05	14	0-20	[5]
Co-substituted Ru nano-sheet	-	0.153	13	0.065	29	10-40	[19]
RuCo@NC	-	0.275	28	0.036	31	-	[20]
RuCoP	-	0.3	23	0.033	37	6-20	[21]
RuCo@NC-600	-	0.255	34	0.039	36	20-40	[4]
Ru_1Ni_1-NCNFs	-	0.612	35	0.016	30	-	[22]
NiRu@N–C	-	0.273	32	0.037	64	-	[23]
NiRu@MWCNTs	-	0.283	14	0.035	32	0-20	[6]
R-NiRu	-	-	16		40	0-50	[14]
$Ru/C_3N_4/C$	-	0.2	80	0.05	-	-	[4]

RuNi nano-sheets	0.027	-	15	0.372	28	0-20	24

[1]: measured at 10 mA/cm^2_{geom}.

[2]: "T.S." stands for "Tafel-slope". The values are as reported. Quotation marks are used as the majority of the reported "Tafel-slopes" were measured at a η region, i.e., below η < RT/F as indicated in column 7 in the Table.

[3]: η region used by the authors to extract the "Tafel-slope".

Table S5. HER data for other, mainly non-PGM catalysts measured in 1 M KOH

The majority of the data were extracted from non-steady state measurements.

Catalyst	Loading (mg_{cat}/cm^2_{geom})	η[1] (mV)	j_{mass}[1] (A/cm^2_{geom})	"T.S."[2] (mV/dec)	Ref.
IrCo@NC-500	0.285	45	0.0351	80	25
Co/Co_3O_4-nano-sheets	0.85	100	0.0118	44	26
CoP films	2.71	94	0.0037	42	27
CoP/Carbon Cloth	0.92	250	0.0109	51	28
MoC_x	0.8	151	0.0125	59	11
Mo_2C@NC	0.28	60	0.0357	60	29
MoP	0.86	140	0.0116	54	30
Mo_2C NP	0.102	176	0.098	58	31

Mo_2C	1.4	190	0.00714	54	[32]
Ni_3S_2/Ni foam	1.6	223	0.00625	-	[33]
Ni_5P_4 pellet	177	49	0.0000566	98	[34]
Ni_2P pellet	177	69	0.0000564	118	[34]
NiO@Ni	2	105	0.005	-	[35]
np-Ni_3N	0.16	68	0.0625	32	[3]

^{1}: measured at 10 mA/cm^2_{geom}.

^{2}: "T.S." stands for "Tafel-slope". The values are as reported. Quotation marks are used as the majority of the reported "Tafel-slopes" were measured at a η region, i.e., below $\eta <$ RT/F as indicated in column 7 in the Table.

2 TABLES FOR OER ACTIVITY MEASUREMENTS

Table S6. OER data for Ir-oxide catalysts measured in 1 M KOH

This Table contains HER data used for Figure 19 in the paper. The majority of the data were extracted from non-steady state measurements.

Catalyst	Supplier	Loading (mg_{cat}/cm^2_{geom})	η^1 (V)	$j_{mass}{}^1$ (A/cm^2_{geom})	"T.S."^{2} (mV/dec)	Ref.
IrO_x (SA-100)	Tanaka Kikinzoku Kogyo	0.2	0.281	0.05	51	[36]
IrO_x	Proton Onsite	0.01	0.393	1.01	47	[37]
IrO_2	-	0.07	0.338	0.143	47	[38]

IrO_2[3]	-	-	0.427	-	49	[39]
IrO_2	-	-	0.32	-	-	[40]
IrO_2	Sigma-Aldrich	0.51	0.378	0.02	98	[41]
IrO_2	-	0.32	0.297	0.031	63	[42]
IrO_2	Commercial	0.142	0.338	0.07	49	[43]
IrO_2	-	0.2	-	0.05	54	[44]
IrO_2	Sigma-Aldrich	0.7	0.32	0.014	76	[45]
IrO_2	-	0.15	0.256	0.067	70	[46]
IrO_2[4]	-	0.204	0.467	0.049	80	[47]
IrO_2	Commercial	0.14	0.39	0.071	79	[48]
IrO_2	Alfa Aesar	0.2	0.339	0.05	59	[49]
IrO_2	Commercial	0.15	0.34	0.067	78	[50]
IrO_2	Sigma-Aldrich	1	0.338	0.01	50	[51]
IrO_2	Alfa Aesar	2.2	0.219	0.005	89	[52]
IrO_2	-	0.14	0.47	0.071	61	[53]
IrO_2	-	0.28	0.39	0.036	78	[54]

[1]: measured at 10 mA/cm^2_{geom}.

[2]: "T.S." stands for "Tafel-slope". The values are as reported. Quotation marks are used as the majority of the measurements used non-steady state methods for the evaluation.

[3]: 1 M NaOH was used as electrolyte.

[4]: 0.1 M KOH was used as electrolyte.

Table S7. OER data for NiFe-based catalysts measured in 1 M KOH

The majority of the data were extracted from non-steady state measurements.

Catalyst	Loading (mg_{cat}/cm^2_{geom})	η^{1} (V)	$j_{mass}^{1,2}$ (A/cm^2_{geom})	"T.S."[3] (mV/dec)	Ref.
NiFe/C	0.36	0.22	0.0278	30	[55]
FeNi/$NiFe_2O_4$ @NC−800	0.131	0.316	0.0763	91	[56]
FeNi@N-CNT	2	0.3	0.005	48	[57]
$Ni_{0.9}Fe_{0.1}$/NC	2	0.27	0.005	45	[58]
Fe-Ni nano-particles	0.029	0.311	0.3448	65	[59]
$NiFe_2O_4$ QDs	0.21	0.262	0.0476	37	[60]
$Ni_{2/3}Fe_{1/3}$-rGO	0.25	0.21	0.04	40	[61]
$Ni_{2/3}Fe_{1/3}$-GO	0.25	0.23	0.04	42	[61]
$Ni_{2/3}Fe_{1/3}$-NS	0.25	0.31	0.04	-	[61]
Ni_3FeN-NPs	0.35	0.28	0.0286	46	[62]
Ni_2Fe_1-O	0.15	0.244	0.0667	39	[50]
Ni-Fe-Se cages	0.1	0.24	0.1	24	[63]
Glassy $Ni_{40}Fe_{40}P_{20}$	1	0.27	0.01	35	[51]

Crystallized $Ni_{40}Fe_{40}P_{20}$	1	0.288	0.01	41	[51]
FeNiP-NP	4.12	0.18	0.0024	76	[64]
n-NiFe LDH /NGF[4]	0.25	0.337	0.04	45	[65]
FeNi-rGO LDH	0.25	0.206	0.04	39	[66]
FeNi LDH	0.25	0.232	0.04	48	[66]
$Fe_6Ni_{10}O_x$	0.1	0.286	0.01	48	[67]
NiFeMn-LDH	0.2	0.262	0.05	47	[68]
Ni_1Fe_2-250	0.17	0.31	0.059	42	[69]
NiFe LDH/C[4]	0.1	0.36	0.1	51	[70]
Fe-Ni hydroxide/GMC	0.147	0.32	0.068	57	[71]
$Na_{0.08}Ni_{0.9}Fe_{0.1}O_2$	0.13	0.26	0.077	44	[72]
$NaNi_{0.9}Fe_{0.1}O_2$	0.13	0.29	0.077	44	[72]
NiFe LDH@ Cu foam	2.2	0.199	0.005	28	[46]
NiFe-B	0.07	0.347	0.143	67	[38]
NiFe-NS	0.07	0.302	0.143	40	[38]
$NiFe_2O_4$	0.8	0.51	0.0125	-	[73]

NiFe-MoO_x NS	0.2	0.276	0.05	55	[74]
$NiFeO_x$ /CoN_y –C	0.196	0.31	0.051	60	[75]
S-NiFe-700@C	0.286	0.281	0.035	53	[76]
3D NiFe LDH/Ni foam[4]	1	0.249	0.01	50	[77]
Ni-Fe/Au	0.14	0.331	0.071	58	[53]
Ni-Fe/2D-ErGO	0.14	0.278	0.071	42	[53]
Ni-Fe film[4]	0.027	0.28	0.374	40	[78]
NiFe-LDH/CNT	0.25	0.247	0.04	31	[79]
NiFe-LDH/CNT[4]	0.25	0.308	0.04	35	[79]
NiFe LDH/oGSH[4]	0.25	0.35	0.04	54	[80]
(Ni–Fe)-LDH/3D-ErGO (8:2)	0.14	0.259	0.071	39	[53]
$Ni_{0.9}Fe_{0.1}O_x$	0.0012	0.336	8.55	30	[39]

RGO-Ni-Fe LDH[4]	0.2	0.25	0.05	33	[81]
NiFeMo	0.28	0.28	0.036	40	[82]
NiFe	0.28	0.315	0.036	40	[82]
NiFe/NF[4]	0.032	0.24	0.312	33	[83]
NiFe/NF	0.032	0.215	0.312	28	[83]
$NiFeO_x$/CFP	1.6	0.23	0.006	32	[84]
$NiFeO_x$/CFP[4]	1.6	0.25	0.006	-	[84]
NiFe LDH[5]	0.05	0.26	0.2	21	[85]
24:0-RT NiFe-LDH[4]	0.1	0.27	0.1	34	[86]
$Ni_{0.75}Fe_{0.25}OOH$ – GCE	0.21	0.286	0.048	-	[87]
$Ni_{0.71}Fe_{0.29}(OH)_x$[4]	0.317	0.296	0.032	58	[88]
$Ni_{0.89}Fe_{0.11}(OH)_x$[4]	0.402	0.348	0.025	78	[88]
HPGC@NiFe LDHs	0.285	0.265	0.035	56	[89]
$Ni_{0.75}Fe_{0.25}HO$-np	0.135	0.23	0.074	24	[90]
NiFe-LDH	0.2	0.247	0.05	37	[36]

FeNi-rGO hybrids	0.25	0.195	0.04	39	66

[1]: measured at 10 mA/cm^2_{geom}.

[2]: j_{mass} is per total catalyst mass

[3]: "T.S." stands for "Tafel-slope". The values are as reported. Quotation marks are used as the majority of the measurements used non-steady state methods for the evaluation.

[4]: 0.1 M KOH.

[5]: 1 M NaOH.

Table S8. OER data for NiCo-based catalysts measured in 1 M KOH

The majority of the data were extracted from non-steady state measurements. The majority of papers do not specify if Fe-free chemicals and electrolytes were used for the synthesis and extraction of OER data.

Catalyst	Loading (mg_{cat}/cm^2_{geom})	η^{1} (V)	$j_{mass}^{1,2}$ (A/cm^2_{geom})	"T.S."[3] (mV/dec)	Ref.
$NiCo_{2.7}(OH)_x$	0.2	0.35	0.05	65	[91]
Ni_2Co_1@$Ni_2Co_1O_x$	0.4	0.32	0.025	-	[92]
NiCoP/rGO	0.15	0.27	0.06667	45	[93]
NiCo diselenide/CC	2.8	0.258	0.00357	42	[94]
NiCo-B	0.07	0.385	0.143	65	[38]
$NiCo_2S_4$	0.07	0.337	0.143	64	[95]

$NiCo_2O_4$	0.07	0.377	0.143	91	[95]
$NiCo_2O_4$@C[4]	1	0.267	0.01	63	[96]
NiCo LDH nano-sheets	0.17	0.367	0.059	53	[97]
Ni-Co-OH[4]	0.82	0.337	0.0122	40	[98]
NiCoP/NF	1.6	0.28	0.0062	75	[99]
$Ni_{0.7}Co_{0.3}$/NC	0.1	0.337	0.1	37	[100]
$Ni_{0.5}Co_{0.5}$/NC	0.1	0.3	0.1	>120	[100]
$Ni_{0.4}Co_{0.6}$/NC	0.1	0.328	0.1	>120	[100]
$Ni_{0.3}Co_{0.7}$/NC	0.1	0.342	0.1	>120	[100]
$Ni_{0.2}Co_{0.8}$/NC	0.1	0.349	0.1	>120	[100]
CoNi hydroxide	0.2	0.324	0.05	-	[49]
CoNi LDH/CoO nano-sheets	0.265	0.3	0.038	-	[101]
CoNi LDH/CoO/GO nano-sheet	0.265	0.315	0.038	-	[101]
$NiCo_2O_4$	0.4	0.36	0.025	55	[102]
$Ni_{0.69}Co_{0.31}$-P	3.5	0.266	0.00286	47	[103]
$NiCoO_2$[4]	0.8	0.39	0.0125	42	[73]
Co_3O_4@Ni	0.83	0.265	0.012	65	[104]

NiCo-NS	0.07	0.334	0.14286	41	[38]
$Ni_{3.5}Co_{5.5}S_8$	0.21	0.333	0.04762	49	[105]
Co_4Ni_1P NTs	0.19	0.245	0.05263	61	[106]

[1]: measured at 10 mA/cm^2_{geom}.

[2]: j_{mass} is per total catalyst mass.

[3]: "T.S." stands for "Tafel-slope". The values are as reported. Quotation marks are used as the majority of the measurements used non-steady state methods for the evaluation.

[4]: 1 M NaOH.

Table S9. OER data for NiCoFe-based catalysts measured in 1 M KOH

The majority of the data were extracted from non-steady state measurements. The majority of papers do not specify if Fe-free chemicals and electrolytes were used for the synthesis and extraction of OER data.

Catalyst	Loading (mg_{cat}/cm^2_{geom})	η^1 (V)	$j_{mass}^{1,2}$ (A/cm^2_{geom})	"T.S."[3] (mV/dec)	Ref.
FeCoNi-2 CP	1	0.288	0.01	-	[107]
FeCoNi-2	0.32	0.325	0.031	60	[107]
NiCoFe LTHs/CFC	0.4	0.239	0.025	32	[108]
O–NiCoFe–LDH[4]	0.12	0.34	0.083	93	[109]
$FeNi_8Co_2$ LDH	0.25	0.224	0.04	42	[110]
Co-Ni-Fe511	0.12	0.288	0.083	55	[30]

Co-Fe-P-1.7/Ni foam	1	0.244	0.01	-	[111]

[1]: measured at 10 mA/cm^2_{geom}.

[2]: j_{mass} is per total catalyst mass.

[3]: "T.S." stands for "Tafel-slope". The values are as reported. Quotation marks are used as the majority of the measurements used non-steady state methods for the evaluation.

[4]: 0.1 M KOH.

Table S10. OER data for Ni-based catalysts measured in 1 M KOH

The majority of the data were extracted from non-steady state measurements. The majority of papers do not specify if Fe-free chemicals and electrolytes were used for the synthesis and extraction of OER data.

Catalyst	Loading (mg_{cat}/cm^2_{geom})	η^1 (V)	j_{mass}^1 (A/cm^2_{geom})	"T.S."[2] (mV/dec)	Ref.
$Ni(OH)_2$/NF	2.9	0.17	0.0034	>120	[112]
Ni@NC-800	0.31	0.28	0.032	45	[113]
$Ni_{2.7}Zn(OH)_x$	0.05	0.29	0.2	43	[114]
$Ni(OH)_2$	0.14	0.331	0.071	-	[115]
NiO	0.14	0.364	0.071	-	[115]
Ni	0.14	0.377	0.071	-	[115]
NiO	0.2	0.43	0.05	81	[116]
$Ni(OH)_2$	0.2	0.36	0.05	111	[116]
NiO-(i)[3]	0.8	0.43	0.012	62	[52]

NiO/Ni-350	0.5	0.345	0.02	53	[117]
Ni/NC	0.1	0.42	0.1	>120	[100]

[1]: measured at 10 mA/cm$^2_{geom}$.

[2]: "T.S." stands for "Tafel-slope". The values are as reported. Quotation marks are used as the majority of the measurements used non-steady state methods for the evaluation.

[3]: 1 M NaOH was used as electrolyte.

Table S11. OER data for Co-based catalysts measured in 1 M KOH

The majority of the data were extracted from non-steady state measurements. The majority of papers do not specify if Fe-free chemicals and electrolytes were used for the synthesis and extraction of OER data.

Catalyst	Loading (mg_{cat}/cm^2_{geom})	η^{1} (V)	$j_{mass}^{1,2}$ (A/cm^2_{geom})	"T.S."[3] (mV/dec)	Ref.
NC@CuCo$_2$N$_x$/CF	2	0.23	0.005	84	[44]
Cu$_{0.3}$Co$_{2.7}$P/NC	4	0.19	0.025	44	[118]
Cu$_{0.5}$Co$_{2.5}$O$_4$	0.5	0.285	0.02	79	[41]
CuCo$_2$S$_4$	0.7	0.31	0.0143	86	[45]
Cu@CoS$_x$/CF	3.9	0.16	0.003	61	[119]
Co$_2$P NW	0.17	0.26	0.059	52	[120]
CoP NWs	0.17	0.32	0.059	64	[120]
CoP NS/C	0.71	0.277	0.014	70	[121]

CoP NR/C	0.71	0.32	0.014	86	[121]
Sandwich-like CoP/C	0.36	0.33	0.028	53	[122]
CoP-MNA	6.2	0.29	0.002	65	[123]
Co_2P@N, P-PCN/CNTs	0.36	0.28	0.028	72	[124]
CoP_2/RGO	0.285	0.3	0.035	96	[125]
CoPh/NG	2.5	0.262	0.004	54	[46]
CoPh/NG GC	0.15	0.31	0.067	98	[46]
CoPs/NG	2.5	0.289	0.004	68	[46]
CoPh/G	2.5	0.292	0.004	-	[46]
CoP/TM	2.1	0.31	0.005	87	[126]
Co-P foam	1.52	0.3	0.007	46	[127]
Co_2P/CNT-900	1	0.292	0.01	68	[128]
Co_5Mn–LDH/MWCNT	0.283	0.3	0.035	74	[129]
Mn-Co oxyphosphide multi-shelled particles	0.25	0.32	0.04	52	[130]
Mn@CoMnO NPs	0.3	0.246	0.033	46	[131]

CoMn LDH	0.142	0.324	0.07	43	[43]
$Co_{0.5}Mn_{0.5}WO_4$ [4]	0.2	0.4	0.05	84	[47]
gelled FeCoW	0.21	0.191	0.048	-	[87]
$Co_6Mo_6C_2$/NCRGO	0.14	0.26	0.071	50	[48]
$CoMoS_4$/β-$Co(OH)_2$	1	0.342	0.01	105	[132]
CoS-$Co(OH)_2$@MoS_{2+x}	0.2	0.38	0.05	68	[133]
$ZnCo_2O_4$ spindle	0.24	0.389	0.042	60	[134]
$Zn_xCo_{3-x}O_4$-3:1 RP arrays	1	0.32	0.01	51	[135]
ZnO-CoO_x/CPEC	0.45	0.276	0.022	59	[136]
NG-$CoSe_2$ [4]	0.2	0.366	0.05	40	[137]
$CoSe_2$/CF	2.9	0.297	0.003	41	[138]
a-CoSe/Ti	3.8	0.292	0.003	-	[139]
peapod-like $Co(S_{0.71}Se_{0.29})_2$	1	0.283	0.01	68	[140]
$Co_2B/CoSe_2$	0.4	0.32	0.025	56	[141]

$Co_{1-x}S$/N–S–G[4]	0.5	0.371	0.02	58	[142]
N–Co_9S_8/G[4]	0.2	0.409	0.05	83	[143]
Co_9S_8/CNS/CNT	0.24	0.267	0.042	48	[144]
Co_9S_8/CNS	0.24	0.294	0.042	51	[144]

[1]: measured at 10 mA/cm^2_{geom}.

[2]: j_{mass} is per total catalyst mass.

[3]: "T.S." stands for "Tafel-slope". The values are as reported. Quotation marks are used as the majority of the measurements used non-steady state methods for the evaluation.

[4]: 0.1 M KOH.

Table S12. OER data for FeCo-based catalysts measured in 1 M KOH

The majority of the data were extracted from non-steady state measurements.

Catalyst	Loading (mg_{cat}/cm^2_{geom})	η^1 (V)	j_{mass}^1 (A/cm^2_{geom})	"T.S."[2] (mV/dec)	Ref.
CoFe LDH-F	0.2	0.3	0.05	40	[145]
Co-Fe-P-1.7	0.424	0.26	0.024	58	[111]
CoFePO	2.2	0.274	0.005	52	[146]
Fe-Co-2.3Ni-B	0.3	0.274	0.033	38	[147]
$Fe_3O_4Co_9S_8$/rGO-2	0.25	0.32	0.04	55	[148]

$Co_{0.7}Fe_{0.3}P$/CNTs	0.5	0.243	0.02	36	[149]
$CoFe_2O_4$/C NRAs	1.03	0.24	0.01	45	[150]
CoFe-LDH[3]	0.204	0.34	0.049	-	[151]
Co–Fe LDH/rGO[3]	0.204	0.325	0.049	-	[151]
NiFeMn-LDH	0.2	0.262	0.05	47	[68]
Fe-Co_3O_4 (32/1)[3]	0.12	0.486	0.083	-	[152]
CoFe35 LDH[3]	0.25	0.35	0.04	47	[153]
V-Co-Fe-343	0.28	0.307	0.036	36	[154]
$Fe_{0.4}Co_{0.6}$	1.2	0.283	0.008	34	[155]
$CoFe_2O_4$-np/PANIMWCNTs	0.285	0.314	0.035	31	[156]
$Co_3Fe_7O_x$/NPC-450	0.36	0.328	0.028	31	[157]
α-$Co_4Fe(OH)_x$	0.28	0.295	0.036	52	[54]
$Fe_{0.5}Co_{0.5}$@NC/NCNS-800	0.306	0.27	0.033	50	[158]
Fe_3O_4/Co(OH$_2$ NSs(Co/Fe 15)	0.111	0.39	0.09	-	[159]

Fe_3O_4/Co(OH$_2$ NSs(Co/Fe 15)^{3}	0.111	0.37	0.09	61	[159]
Fe_3O_4-Co_3S_4 NS	0.672	0.27	0.015	56	[160]
G-FeCoW - Au foam	0.21	0.191	0.048	-	[87]
LDH FeCo - Au foam	0.21	0.279	0.048	-	[87]
G-FeCo - Au foam	0.21	0.215	0.048	-	[87]
A-FeCoW - Au foam	0.21	0.232	0.048	-	[87]
LDH FeCo – GCE	0.21	0.331	0.048	-	[87]
G-FeCo – GCE	0.21	0.277	0.048	-	[87]
G-FeCoW – GCE	0.21	0.223	0.048	-	[87]
A-FeCoW – GCE	0.21	0.301	0.048	-	[87]
$Co_{0.54}Fe_{0.46}OOH^{3}$	0.2	0.39	0.05	47	[161]

^{1}: measured at 10 mA/cm$^{2}_{geom}$.

[2]: "T.S." stands for "Tafel-slope". The values are as reported. Quotation marks are used as the majority of the measurements used non-steady state methods for the evaluation.

[3]: 0.1 M KOH was used as electrolyte.

Table S13. OER data for Fe-based catalysts measured in 1 M KOH

The majority of the data were extracted from non-steady state measurements.

Catalyst	Loading (mg_{cat}/cm^2_{geom})	η^1 (V)	$j_{mass}{}^1$ (A/cm^2_{geom})	"T.S."[2] (mV/dec)	Ref.
Fe_3C/Fe@NC NTs-NCNFs	0.56	0.284	0.018	56	[162]
$Fe_{0.5}V_{0.5}$	0.143	0.39	0.07	38	[163]
$Fe(TCNQ)_2$/Fe	0.49	0.36	0.02	110	[164]
Fe_2O_3[3]	0.8	1.24	0.01	-	[73]

[1]: measured at 10 mA/cm^2_{geom}.

[2]: "T.S." stands for "Tafel-slope". The values are as reported. Quotation marks are used as the majority of the measurements used non-steady state methods for the evaluation.

[3]: 1 M NaOH was used as electrolyte.

Table S14. OER data for NiP-based catalysts measured in 1 M KOH

The majority of the data were extracted from non-steady state measurements. The majority of papers do not specify if Fe-free chemicals and electrolytes were used for the synthesis and extraction of OER data.

Catalyst	Loading (mg_{cat}/cm^2_{geom})	η^{1} (V)	$j_{mass}{}^{1}$ (A/cm^2_{geom})	"T.S."[2] (mV/dec)	Ref.
Ni-P/NF	3.2	0.309	0.003	58	[165]
Ni_2P	0.14	0.29	0.071	47	[115]
Ni-P	0.2	0.3	0.05	77	[116]
C@Ni_8P_3	1.9	0.267	0.005	51	[166]
Ni_xP_y-325	0.15	0.32	0.067	107	[167]
$Ni_{12}P_5$/NF	3	0.24	0.003	106	[168]
Ni_2P/NF	3	0.26	0.003	112	[168]
$Ni_{12}P_5$/NF	1	0.295	0.01	-	[168]
Ni_2P/NF	1	0.33	0.01	-	[168]
CP@Ni-P	25.8	0.19	0.0004	73	[169]
MoS_2/Ni_3S_2 Heterostructures	9.7	0.218	0.001	83	[170]
Ni_2P@NF-6	5.6	0.142	0.002	109	[171]
NiCuP	6.7	0.292	0.001	49	[172]

[1]: measured at 10 mA/cm^2_{geom}.

[2]: "T.S." stands for "Tafel-slope". The values are as reported. Quotation marks are used as the majority of the measurements used non-steady state methods for the evaluation.

Tables 2, 3, 5, 7-14 contain some information provided by Kibgsaard et al.[173] Additional metrics and various catalysts are added in this publication.

3 PROTOCOLS TO EVALUATE HER AND OER CATALYSTS

As discussed throughout the review paper, consistent catalytic activity and stability measurements for HER and OER electro-catalysts at conditions mimicking AEMWE operation are needed. Therefore, in the following sections recommendations for measurement procedures are made.

The need for accurate HER and OER catalysts measurements has already been discussed throughout the catalyst section in the review paper. As discussed, steady-state measurements need to be carried out to measure mass activities, intrinsic activities and Tafel-slopes in valid η regions. The measurement of the HER characteristics is more challenging than for the OER as the HER and its corresponding back reaction, namely the oxidation of adsorbed hydrogen (the HOR) are fast electrochemical reactions and the reaction can be kinetically controlled. The end goal of the measurements should be a plot showing the steady-state data as a Tafel plot, i.e., η vs. the logarithm of the current density and a Table showing metrics relevant to the HER and OER as suggested in the following Table S15.

Table S15. Template suggesting a format to report HER and OER catalyst metrics*[1]*

Catalyst	Catalyst loading[2] (µg/cm^2)	η at 10 mA/cm$^2_{geom}$ (mV)	j_{mass} at η = 70 mV	j_{int} at η = 70 mV	Tafel slope[3] (mV /dec)	ECSA[4] /m^2 g^{-1}

"Catalyst of interest"	….	….	….	….	….	….
"Commercial baseline catalyst"[5]	….	….	….	….	….	….

[1]: The electrolyte solution needs to be given as well as the temperature used for the measurements.

[2]: The catalyst loading needs to be listed. Ideally, the wt.% of the active component of the catalyst, which typically is a metal is measured.

[3]: The Tafel plots need to be extracted in a η region exceeding RT/F, i.e., > 50 mV. Some catalysts show more than one Tafel slope. In this case, both slopes and the corresponding η regions need to be published.

[4]: ESCA is the electrochemical surface area. It is not possible to determine the ECSA accurately for every catalysts but trends can be established. The method used to determine or approximate the ECSA value needs to be stated.

[5]: The same data (as for the catalyst of interest) need to be measured and reported for at least one baseline catalyst from a commercial supplier. The supplier of the baseline catalyst needs to be stated. In case of the HER, a commercial Pt/C catalysts (20 or 40 wt.% Pt/C, C being Vulcan XC-72 or Ketjenblack) is recommended. In case of the OER, a commercial unsupported IrO_x powder catalyst is recommended.

In addition to reporting the electrochemical characteristics, a full physical characterisation of in-house developed as well as commercial baseline catalysts is needed

using methods as XRD, SEM, TEM and XPS. The study by Mahmood et al., is a good example demonstrating an appropriate variety of physical and analytical characterisation techniques.[10]

3.1 Electrode Preparation

In case of catalyst powders, a catalyst ink, from which an aliquot is pipetted onto a flat and inert electrode surface, is typically formed and left to dry on air and room temperature. Afterwards, a thin layer of a (in water or in lower alcohols soluble) ionomer is deposited onto the catalyst layer. Again, this is done by pipetting a small volume of a very dilute ionomer solution across the entire catalyst layer surface. This ionomer layer serves the purpose of holding the catalyst on the electrode surface, i.e., acting as a glue during the electrochemical measurements. Often, a solution of approx. 0.025 wt.% Nafion in H_2O is used and typically 5-10 µLs are pipetted onto e.g., a 0.196 cm^2_{geom} (0.25 mm diameter) circular electrode surface area of a rotating disc electrode (RDE). Reports exist (specifically in the case of the HER) of ionomers altering the catalyst activity (see section 6 in the review paper) hence, the ionomer is not added to the ink and very dilute ionomer solution are used to minimise any possible interactions. In case of doubt, catalytic activity results can be compared to results obtained from electrodes made using different ionomers, e.g., without sulfonic acid or phenolic groups or using different amounts of ionomers. It is of high importance that a thin ionomer (glue) layer is applied on top of the thin catalyst layer to ensure rapid product and reactant flow from and to the catalyst sites. This has been extensively discussed for the evaluation of the O_2 reduction reaction (ORR), which is mass

transport controlled.[174] Mass-transport limitations are of lower concern for the OER in thin layer electrodes, however, the guidelines established for the ORR should be followed.

The catalyst needs to be uniformly distributed on an inert electrode surface of known area. RDEs are well suited as electrodes for thin layer measurements as they have smooth and defined surface areas. In case, of the HER measurements, rotating the electrodes is also needed to rule out mass transport limitations. For the HER, glassy carbon electrodes and for the OER, gold or even glassy carbon and smooth nickel electrodes can be used. It needs to be kept in mind that these electrodes can exhibit HER and OER activities. Therefore, establishing the background catalytic activities (i.e., HER or OER currents) of the electrode substrates is essential prior to any activity measurement. The catalyst loading (measured in mg/cm^2_{geom}) influences the activity, although not in a uniform manner. Therefore, measurements of the activity at different catalyst loadings are needed to ensure that a catalyst loading range, which yields a constant mass activity as explained by Anantharaj et al.[175] is used. For example, catalyst loadings in the range of 5 to 15 $\mu g/cm^2_{geom}$ are typical for very active OER catalysts. The amount of the active component in the catalyst, specifically for supported catalysts, needs to be identified.

For accurate measurements, a real ink needs to be formed, which can be challenging. A known amount of catalyst, which depends on the activity of the catalyst, is weight into water or a lower alcohol-based solution of known volume and sonicated for 30 to 60 min. Catalysts can also be directly formed on the electrode. Again, in order to determine the catalyst activity, the catalyst loading and the ECSA need to be known for the determination of the mass and the intrinsic activities, respectively.

3.2 Electrolyte

Most commonly 1 M KOH is used as electrolyte for thin layer catalyst activity measurements. Some reports exist for NaOH or 0.1 M KOH electrolytes, however for consistency, it is recommended to carry out the measurements in 1 M KOH. If another electrolyte is of interest as feed for single cell AEMWE measurements, it is recommended to carry out activity measurements in 1 M KOH as well as in the other electrolyte for the catalysts of interest as well as the baseline catalyst. The alkali electrolyte cannot be stored in glass. In case, of Fe-free measurements, high purity KOH needs to be used. Fe can be removed from KOH using either a chemical or an electrochemical method.[176,177]

3.3 Counter and Reference Electrodes

High surface area counter electrodes need to be used in order to avoid dissolution of the counter electrode and avoid erroneous capacitive contribution. In case of the HER, a high surface area electrode such as a carbon rod is recommended. Pt cannot be used for the HER, as it has a high HER activity and dissolves upon potential cycling into the Pt-oxide formation region. This can lead to deposition of Pt onto the working electrode, i.e., onto the catalyst of interest, thus resulting in erroneous HER activity measurements.

A Hg/HgO or reversible hydrogen electrode (RHE) needs to be used for the measurements in alkaline electrolytes. The reference electrode needs to be calibrated before and after each experiment. Reference electrodes such as Ag/AgCl combinations are not suitable as the AgCl is transformed into silver-hydroxide in alkaline solutions. The reference electrode will need to be placed close to the working electrode surface to reduce the IR-drop, however a distance of two times the diameter of the reference electrode needs

to be maintained to ensure uniform potential and current distribution. Ideally, a Luggin capillary is used and can be made out of e.g., Teflon tubing. The IR drop should be less than a few mV, otherwise an IR drop correction need to be applied.

3.4 Activity Measurements

The electrochemical steps recommended for the performance assessment are as follows: Carbonates are removed by N_2 or Argon bubbling through the alkaline electrolyte in order to prevent a drop in the pH. The HER, is carried out in saturated H_2 electrolyte, while the OER is carried out in a saturated O_2 electrolyte at a constant temperature such as 20 ^oC. Carrying out CVs at least before and after the Tafel slope measurements is strongly recommended to allow comparison between different electrodes and understand if the catalyst is altered and/or physically detached during the measurements. Removal of H_2 or O_2 gas bubbles from the electrode surface is needed to ensure that the electrode surface is not blocked. Some research group rotate the RDE with the purpose to remove gas bubbles. However, rotating a horizontal surface has the opposite effect and gas bubbles will be pulled to the center of the electrode.

Steady-state Tafel plots can be obtained by gradually stepping the working electrode potential in 10 or 25 mV steps and measuring the current after a 5 min. period. Repeats of the current measurements on the same electrode need to be included to ensure that the catalyst is not altered during the experiment. At least three electrodes need to be measured. The procedures of measuring HER and OER activities in this manner are described in the literature by e.g., Lyons et al.[178]

4 PROTOCOL TO EVALUATE HER AND OER CATALYST STABILITY

A few protocols to measure HER and OER catalysts stability have been suggested with the goal to increase the accuracy of the measurements. Many stability measurements are solely based on electrochemical experiments.[179] Electrochemical stability measurements should last at least a few (10-24) h, but run for several hundreds of hours for catalysts of high interest. Measurements over longer periods allow to probe the catalyst stability when the catalyst has reached a thermodynamically stable state under relevant conditions. Care needs to be taken in selecting the substrate on which the catalyst is deposited and studied. A substrate needs to be stable under the conditions of interest and not display significant catalytic activity for the reaction of interest. A common substrate for HER studies outside of an AEMWE cell (e.g., using an H-cell) is glassy carbon, while gold, fluorine-doped tin-oxides and sometimes also glassy carbon is used as substrate for the OER. Nickel metal could be a substrate for OER catalyst stability studies. It needs to be remembered that nickel on its own has some OER activity, which could be enhanced during the stability study by Fe incorporation and/or possible surface alterations.

4.1 Stability Measurements

CVs should be performed before and after the stability tests. OER and HER stability measurements can be performed at 1.6 V and at -0.1 V vs. RHE, respectively. However, measurements at additional potentials and the addition of cycling the potential within a narrow region also provide meaningful information (see Tables S16 and S17) and[73,180]. Measurements at a constant potential (chrono-amperometric) provide thermodynamic control, while potential cycling experiments reflect changing conditions and fluctuations,

which can occur in a CL during AEMWE operation. In addition, experiments from a specific η value to open-circuit conditions (transient experiments) are also recommended to mimic start-up and shut-down operation (Table S18). The bare electrode substrate should also be tested using the same experimental conditions to establish the baseline. The stability measurements need to be carried out at constant temperature conditions. Higher temperatures such as 60-80 °C can be a benefit as it could reflect accelerated tests and also real operating conditions but the stability of other components such as the ionomer needs to be considered.

Table S16. Suggested testing conditions and template for reporting catalyst stability measurements by chrono-amperometry

**E at η / V**	**Activity initial (A/mg_{cat})**	**Activity final (A/mg_{cat})**	**Activity changes (%)**	**ECSA initial (m^2/g_{cat})**	**ECSA final (m^2/g_{cat})**	**ECSA changes (%)**
0.1	….	….	….	….	….	….
0.2	….	….	….	….	….	….
0.3	….	….	….	….	….	….
0.5	….	….	….	….	….	….
0.7	….	….	….	….	….	….

Table S17. Suggested testing conditions and template for reporting catalyst stability measurements by potential cycling within a small voltage range

**Potential cycle[1] (V)**	**Activity initial (A/mg_{cat})**	**Activity final (A/mg_{cat})**	**Activity changes (%)**	**ECSA initial (m^2/g_{cat})**	**ECSA final (m^2/g_{cat})**	**ECSA changes (%)**
0.1-0.2	….	….	….	….	….	….
0.1-0.3	….	….	….	….	….	….
0.1-0.4	….	….	….	….	….	….
0.1-0.5	….	….	….	….	….	….
0.1-0.7	….	….	….	….	….	….

[1]: The values in column 1 are given as η.

Table S18. Suggested testing conditions and template for reporting catalyst stability measurements mimicking start-up an shut-down conditions: Transient cycling

**Transient cycle[1]**	**Activity initial (A/mg_{cat})**	**Activity final (A/mg_{cat})**	**Activity changes (%)**	**ECSA initial (m^2/g_{cat})**	**ECSA final (m^2/g_{cat})**	**ECSA changes (%)**
1	….	….	….	….	….	….
2	….	….	….	….	….	….
N_{th}[2]	….	….	….	….	….	….

[1]: A transient cycle consist of a potential step and holding at a defined η value of e.g., 0.1, 0.2 or 0.3 V followed by exposure for a defined period of time to open-circuit conditions. Between 10 to a 100 transient cycles are recommended.

[2]: N_{th} equals the transient cycle number.

The electrochemical stability measurements need to be coupled with an analytical technique such as ICP-MS/OES to quantify metal ion dissolution during the course and/or at end of the stability test. Calibration for the ICP-MS/OES is needed. KOH electrolyte samples require acidification using HNO_3 prior to the ICP-MS/OES measurement to ensure that metal ions in the electrolyte are completely dissolved. The catalysts need be thoroughly characterized before and after the stability measurements, as also suggested in section 3 above. Details for the selection of counter and reference electrodes are also given in section 3 above.

5 ECSA MEASUREMENTS FOR HER/OER ACTIVITY REPORTING

As mentioned throughout the catalysts section of the review paper consistent measurements of HER and OER activities that allow comparison and validation among studies are needed. Intrinsic activities are important to understand a catalyst, thus the knowledge of the ECSA of a catalyst is crucial. Unfortunately, reliable ECSA methods for many of the catalysts discussed in the review paper are not available. Therefore, the ECSA methods and its challenges are briefly discussed below.

For Pt and Pt-based catalyst, the ECSA can be determined from the $H_{ads/des}$ charge as described in the literature.[181] For polycrystalline Pt, the $H_{ads/des}$ charge is converted to the Pt surface area using a conversion factor of 210 C/cm^2_{Pt}.[181]

In case of catalyst sites in the metallic state, CO_{ads} stripping voltammetry can also be used. CO does not adsorb as a monolayer on catalyst sites present in the oxide form and similarly the under potential deposition (upd) method of metals like the Cu_{upd} is specific to catalyst in the metallic state.[181]

Other electrochemical methods have been used to estimate ECSA values such as C_{dl} measurements and charges observed for the oxidation and/or reduction of the catalyst sites. These methods can be of value to establish trends but they often do not yield accurate ECSA values.[182]

Some researchers have applied different methods to extract ECSA values and for some catalysts similar ECSA values extracted from different methods have been reported. An example is a study for Ru nano-particle catalysts embedded in a functionalised carbon matrix (as already mentioned in section 3.2 in the review paper), where the agreement between the $H_{ads/des}$, CO_{ads} and Cu_{upd} methods allowed the extraction of the number of metallic Ru sites in the catalyst.[10] Anderson et al. also used such methods to obtain estimates of ECSA values for powder catalysts by extrapolating from ECSA values determined for the corresponding bulk metal electrodes.[180]

In summary, accurate ECSA values and therefore, also accurate intrinsic catalytic activities can be extracted for some catalysts, while for a large number of catalysts only trends can be determined.

6 SUMMARY OF AEMS EVALUATED IN AEMWE SINGLE CELLS

Table S19. Summary of the various developed to date AEMs with their performances in AEMWE single cells

Backbone	Ref.	Membrane Electrode Assembly				AEMWE Cell Performance				
		**Membrane**	**Anode Catalyst loading mg/cm²**	**Cathode Catalyst loading mg/cm²**	**Ionomer**	**Voltage (V)**	**Current density (mA/cm²)**	**T (°C)**	**Electrolyte**	**Time (h)**
**Polyphenylene based AEMs**										
Polyphenylene	183	ATM-PP	IrO_2,3	Pt black, 3	F-PAE	2.2-2.5	200	50	H_2O	2000
	184	Quaternized PP	IrO_2, 0.6	Pt Ru/C, 0.3	BPN	1.8	150	80	H_2O	-
	185	HTMA-DAPP	Ni-Fe, 3	Pt-Ru/C, 2	TMA-70 or TMA-53	1.8	2700	85	H_2O	-
		HTMA-DAPP	Ni-Fe, 3	Pt-Ru/C, 2	TMA-70	~1.6-2.5	200	60	H_2O	14
		HTMA-DAPP	Ni-Fe, 3	Pt-Ru/C, 2	TMA-53	~1.75-2.1	200	60	H_2O	170
	186	HTMA-DAPP	IrO_2, 0.75	Pt-Ru/C, 0.36	HTMA-DAPP	2.0	400	60	H_2O	-
	187	HTMA-DAPP	Co_3O_4, 3	Pt/C, 3	Aemion	1.9-2.1	500	50	1 wt.% K_2CO_3	750
	188	PTP-90	IrO_2, 2.5	Pt/C, 0.5	-	2.2	1000	75	1 M NaOH	-
		PTP-90	IrO_2, 2.5	Pt/C, 0.5	-	2.13-2.28	400	55	1 M NaOH	120
	189	BPN1-100	PGM , 2	PGM, 2	AS-4	2.0-2.13	200	50	H_2O	6
		TPN1-100	PGM , 2	PGM, 2	AS-4	2.15-2.21	200	50	H_2O	6
	190	PAP-TP-85	Fe_xNi_yOOH-20F, 4.8	Pt/C, 0.94	PAP-TP-85 and PAP-TP-85 MQN	1.74	1500	80	1 M KOH	-

		PAP-TP-85	Fe_xNi_yOOH-20F, 4.8	Pt/C, 0.94	PAP-TP-85 and PAP-TP-85 MQN	1.8	1020	90	H_2O	-
		PAP-TP-85	Fe_xNi_yOOH-20F, 4.8	Pt/C, 0.94	PAP-TP-85 and PAP-TP-85 MQN	1.62-1.68	200	80	H_2O	>160
		PAP-TP-85	Fe_xNi_yOOH-20F, 4.8	Pt/C, 0.94	PAP-TP-85 and PAP-TP-85 MQN	1.71-1.81	500	80	H_2O	70
**Polyfluorene**	[191]	PFTP-13	IrO_2, 2	Pt/C, 0.5	PFTP-8/PFBP-14	2.0	7680	80	1 M KOH	-
		PFTP-13	IrO_2, 2	Pt/C, 0.5	PFTP-8/PFBP-14	~2.1	500	60	1 M KOH	~1100
		PFTP-8	IrO_2, 2	Pt/C, 0.5	PFTP-8/PFBP-14	2.0	4880	80	1 M KOH	-
		x-PFTP	IrO_2, 2	Pt/C, 0.5	PFTP-8/PFBP-14	2.0	3600	80	1 M KOH	-
		PFTP-13	IrO_2, 2	Pt/C, 0.5	PFTP-8/PFBP-14	2.0	1800	80	H_2O	-
		PFTP-13	Ni-Fe, 20	Ni-Fe, 20	-	2.0	1600	80	1 M KOH	-
		PFTP-13	Ni-Fe, 20	Ni-Fe, 20	-	~1.5	500	60	1 M KOH	~1000
**Poly arylene ether based AEMs**										
**Polyarylene ether**	[192]	$PPO\text{-}TMA^+OH^-$	IrO_2, 2.5	Pt black, 2.5	$PPO\text{-}TMA^+$ OH^-	1.6-1.7	100	50	H_2O	5
		$PPO\text{-}ABCO^+OH^-$	IrO_2, 2.5	Pt black, 2.5	$PPO\text{-}ABCO^+$ OH^-	1.8-2.0	100	50	H_2O	5
	[193]	PPO24-BIM	IrO_2, 3	Pt/C, 1.5	PTFE	1.8	300	50	0.5 M KOH	900 cycles
	[194]	qPPO-TMA	$NiCo_2O_4$, 8	Pt/C,0.3	qPPO	2.0	1000	70	10 wt.% KOH	-

		qPPO-TMA	$NiCo_2O_4$, 8	Pt/C,0.3	qPPO	1.79-1.84	300	50		400
	195	LSCPi	IrO_2, 8	Pt/C, 0.4	PTFE	1.8	300	50	H_2O	-
		LSCPi	IrO_2, 8	Pt/C, 0.4	PTFE	1.74-2.3	200	50	H_2O	35
**Poly arylene ether ketone**	196	PAEK-APMP	Ni foam, -	Ni foam,-	-	2.0	~25	60	10 wt.% KOH	-
**Poly sulfone**	197	xQAPs	Ni-Fe, -	Ni-Mo, 40	xQAPS	1.8-1.85	400	70	H_2O	8
	198	PSf-DABCO	$NiCO_2O_4$, 5	$NiFe_2O_4$, 1.5	PTFE	2.0	180	50	10 wt.% KOH	-
	199	$PSF\text{-}TMA^+OH^-$	$Pb_2Ru_2O_{6.5}$, 2.5	Pt black, 2.5	$PSF\text{-}TMA^+ OH^-$	1.8	400	50	H_2O	-
		$PSF\text{-}TMA^+OH^-$	$Pb_2Ru_2O_{6.5}$, 2.5	Pt black, 2.5	$PSF\text{-}TMA^+ OH^-$	1.51-2.33	200	50	H_2O	6
**Polybenzimidazole based AEMs**										
200		p-PBI	Ti_nO2_n-1-supported nano Pt, 0.2	Ti_nO2_n-1-supported nano Pt, 0.2	-	2.0	100	80	H_2O	-
201		linear, crosslinked, and thermal cured PBI	Ni, -	Ni, -	-	2.0	100 -210	80	30 wt.% KOH	-
202		linear, crosslinked, and thermal cured PBI	Ni, -	Ni, -	-	1.8	<100	80	30 wt.% KOH	-
		C-ABPBI	Ni Foam, -	Ni Foam, -	-	2.0	335	70	3 M KOH	-

	203									
		C-ABPBI	Ni Foam, -	Ni Foam, -	-	2.0	180	50	1.9 M KOH	-
		L-ABPBI	Ni Foam, -	Ni Foam, -	-	2.0	280	70	3 M KOH	-
		L-ABPBI	Ni Foam, -	Ni Foam, -	-	2.0	155	50	1.9 M KOH	-
	204	PBI based AEM	Ni-Fe-O_x, 5	Ni-Fe-Co, 5	Sustainion® XB-7	2.09-2.08	1000	60	1 M KOH	100
		PBI based AEM	Ni-Fe-O_x, 5	Ni-Fe-Co, 5	Sustainion® XB-7	1.89-1.88	600	60	1 M KOH	100
	205	m-PBI	NiAl, -	NiAlMo, -	-	1.8	1700	80	24 wt.% KOH	-
	206	HMT-PMBI	NiAlMo, 42.7	NiAlMo, 42.7	-	2.086	2000	60	1 M KOH	-
		HMT-PMBI	NiAl, 47.9	NiAlMo, 42.7	-	2.1	1000	60	1 M KOH	154
	207	HMT-PMBI	Pt, 0.5	Pt, 0.5	HMT-PMBI (Cl^-)	2.2-2.5	25	60	1 M KOH	195 (+50 h conditioning)
	208	m-PBI	Ni Foam, -	Ni Foam, -	-	2.4	1500	80	20 wt.% KOH	-
	209	Mes-PBI	None noble metals, -	None noble metals, -	-	2.0	200	80	25 wt.% KOH	~80
**Polyolefin-based AEMs**										
**Polyethylene**	210	LDPE-g-VBC-DABCO	Proprietary non PGM, ACTA SpA catalyst	Proprietary non PGM, ACTA SpA catalyst	AS4	2.08-2.25	460	45	1 wt.% K_2CO_3	500
	211	Aminated poly (LDPE-co-VBC)	$Cu_xMn_{0.9-x}Co_2.1O_4$, 3	Pt/C, 1	Develop-mental ionomer	1.82	100	40	1 M KOH	-

	212	LDPE-g-VBC-TMA	$NiCo_2O_4$, 10	Pt/C, 0.4	PSEBS-CM-TMA	1.65	100	60	0.1 M KOH	-
		LDPE-g-VBC-TMA	$NiCo_2O_4$, 10	Pt/C, 0.4	PSEBS-CM-TMA	1.72	100	20	1 M NaOH	-
**Polystyrene**	213	PSEBS-CM-TMA/PTFE	$NiCo_2O_4$, 8	Pt/C, 0.3	PSEBS-CM-TMA or PTFE	1.76 - 1.84	300	50	10 wt.% KOH	800
	214	SEBS-Pi	IrO_2, 2	Pt/C, 2	CMSEBS	2.08	400	50	5.6 wt.% KOH	105
	215	PSEBS-CM-DABCO	$NiCo_2O_4$, 10	$NiFe_2O_4$, 10	PSEBS-CM-DABCO	2.0	120	40	10 wt.% KOH	-
		PSEBS-CM-DABCO	$NiCo_2O_4$, 10	$NiFe_2O_4$, 10	PSEBS-CM-DABCO	2.26	300	50	10 wt.% KOH	160
		PSEBS-CM-DABCO	$NiCo_2O_4$, 10	$NiFe_2O_4$, 10	PSEBS-CM-DABCO	2.0	150	40	15 wt.% KOH	-
	216	poly (ST-*co*-VBC)	Ni, -	Ni, -	-	2.5	100	25	H_2O	~ 1.3
	187	SES-TMA, 53 µm	Co_3O_4, 3	Pt/C, 3	Aemeon	1.9-2.0	500	50	1 wt.% K_2CO_3	~ 500
	217	mm-qPVBz/Cl^-	$Cu_{0.7}CO_{2.3}O_4$, 3	Nano Ni, 2	QPVB/Cl^-	1.99	100	55	H_2O	-
**Polytetrafluoroethylene**	218	Cranfield-membrane	$Cu_{0.7}CO_{2.3}O_4$, 3	Nano Ni, 2	QPDTB	1.9	100	22	H_2O	-
		Cranfield-membrane	$Cu_{0.7}CO_{2.3}O_4$, 3	Nano Ni, 2	QPDTB	1.8	50	20-30	H_2O	~5
	219	Cranfield-membrane	$Li_{0.21}CO_{2.79}O_4$, 2.5	Nano Ni, 2	QPDTB-OH^-	2.05	300	45	H_2O	-
		Cranfield-membrane	$Li_{0.21}CO_{2.79}O_4$, 2.5	Nano Ni, 2	QPDTB-OH^-	2.15	300	30	H_2O	10

**Polycarbazole**	220	QPC-TMA	IrO_2, 2	Pt/C, 0.4	QPC-TMA	1.9	3500	70	1 M KOH	-
		QPC-TMA	IrO_2, 2	Pt/C, 0.4	QPC-TMA	1.6	780	70	1 M KOH	<3
**Composite AEMs**										
221		PBI /FAA-3 – 20% PF-41	IrO_2 3.5	Pt/C, 1.5	PTFE	2.0	200	60	20 wt.% KOH	100
222		C-PVA-ABPBI 4:1	Ni foam, -	Ni foam, -	-	2.0	450	50	15 wt.% KOH	-
			Ni foam, -	Ni foam, -	-	~2.10	200	50	15 wt.% KOH	1
			Ni foam, -	Ni foam, -	-	2.0	900	70	15 wt.% KOH	-
			Ni foam, -	Ni foam, -	-	~1.95	200	70	15 wt.% KOH	1
		L-PVA-ABPBI 4:1	Ni foam, -	Ni foam, -	-	2.0	220	50	15 wt.% KOH	-
			Ni foam, -	Ni foam, -	-	~2.55	200	50	15 wt.% KOH	1
			Ni foam, -	Ni foam, -	-	2.0	290	70	15 wt.% KOH	-
			Ni foam, -	Ni foam, -	-	~2.25	200	70	15 wt.% KOH	1
		L-PVA-PBI 4:1	Ni foam, -	Ni foam, -	-	2.0	180	50	15 wt.% KOH	-

		Ni foam, -	Ni foam, -	-	~2.3	200	50	15 wt.% KOH	1
		Ni foam, -	Ni foam, -	-	2.0	430	70	15 wt.% KOH	-
		Ni foam, -	Ni foam, -	-	~2.15	200	70	15 wt.% KOH	1
223	Dowex Marathon A+ LDPE blend and press-molded between poly(ethylene terephthalate) films	$NiCo_2O_4$, 8	Pt/C, 0.3	qPPO	1.70-1.75	300	70	1 M KOH	100
		$NiCo_2O_4$, 8	Pt/C, 0.3	qPPO	1.97-2.0	300	70	0.5 M Na_2CO_3	100
		$NiCo_2O_4$, 8	Pt/C, 0.3	qPPO	~1.8	300	50	1.95 M KOH	100h
224	PTFE/QPDTB	$Cu_{0.6}Mn_{0.3}Co_{2.1}O_4$, 3	Pt/C, 0.1	qPDTB-OH^-	1.61-1.75	100	22	H_2O	175
225	PISPVA46	IrO_2, 2.01	Pt/C, 0.5	Nafion EW1100	2.0	547.7	60	0.5 M KOH	-
	PISPVA46	IrO_2, 2.01	Pt/C, 0.5	Nafion EW1100	1.8	354.1-224	60	0.5 M KOH	80
226	120 µm PSU/PVP(25:75)	Ni foam, -	Proprietary cathode, -	-	2.0	500	80	20 wt.% KOH	700
227	PFSA/PVP	Ni, -	Ni, -	-	2.0	~100	80	30 wt. % KOH	-
228	LDPE-PEG-PPG-ANEX	$NiCo_2O_4$, 10	Ni, -	MEA-qPPO	1.85	135	50	10 wt. % KOH	-
	LDPE-PEG-PPG-ANEX	$NiCo_2O_4$, 10	Ni, -	PTFE	2.02-2.05	225	50	10 wt. % KOH	135
**Commercial AEM**									
229	A-201 Tokuyama	IrO_2, 2.9	Pt black, 3.2	As-4	1.8	399	50	1 M KOH	-
		IrO_2, 2.6	Pt black, 2.4	A-Radel	2.0-2.15	200	50	H_2O	>535

230	A-201 Tokuyama	$CuCoO_x$ (Acta 3030), 36	Ni/(CeO_2-La_2O_3)/C (Acta 4030), 7.4	PTFE	1.75	470	43	1 M KOH	-
	A-201 Tokuyama	$CuCoO_x$ (Acta 3030), 36	Ni/(CeO_2-La_2O_3)/C (Acta 4030), 7.4	PTFE	1.93	470	43	1 wt.% K_2CO_3/$KHCO_3$	1000
	A-201 Tokuyama	$CuCoO_x$ (Acta 3030), 36	Ni/(CeO_2-La_2O_3)/C (Acta 4030), 7.4	PTFE	1.85	470	43	1 wt.% K_2CO_3	1000
231	A-201 Tokuyama	Ni/CP, 0.017	Ni/CP, 0.017	-	1.9	150	50	1 M KOH	-
232	A-201 Tokuyama	IrO_2, -	Pt/C, -	PTFE	1.8	299	50	0.5 M KOH	-
	A-201 Tokuyama	IrO_2, -	Pt/C, -	PTFE	2.2	~800	50	0.5 M KOH	100 cycles
233	A-201 Tokuyama	IrO_2, -	Pt/C, -	PTFE	1.8	1070	50	0.5 M KOH	-
	A-201 Tokuyama	IrO_2, -	Pt/C, -	PTFE	2.2	~1500	50	0.5 M KOH	1600 cycles
234	A-201 Tokuyama	$CuCoO_x$ (Acta 3030), 30	Ni/(CeO_2-La_2O_3)/C (Acta 4030), 7.4	Ionomer I_2/ Teflon AF	1.98-2.08	500	60	1 wt.% K_2CO_3	200
	FAA-3-PP-75	$CuCoO_x$ (Acta 3030), 30	Ni/(CeO_2-La_2O_3)/C (Acta 4030), 7.4	Ionomer I_2/ Teflon AF	2.05 - 2.43	500	60	1 wt.% K_2CO_3	200
235	A-901 Tokuyama	Acta 3030® ($CuCoO_x$), 30	Acta 4030® (Ni/(CeO_2-La_2O_3)/C), 7.4	Ionomer I_2/Teflon AF	1.94	400	50	1 wt.% K_2CO_3	-
	A-901 Tokuyama	Acta 3030® ($CuCoO_x$), 30	Acta 4030® (Ni/(CeO_2-	Ionomer I_2/Teflon AF	2.13 - 2.17 V	500	50	1 wt.% K_2CO_3	180

			La_2O_3)/C), 7.4						
236	FAA3-PK-130	$Ce_{0.2}MnFe_{1.8}O_4$, 3.5	Ni, 3.5	FAA3-PK-130	1.8	300	25	H_2O	>100
237	FAA-3-PK-130	NiFe-BTC-GNPs/NF, 2.5	$NiMO_4/MoO_2$/NF, 2.5	FAA-3-PK-130	1.85	540	70	H_2O	-
	FAA-3-PK-130	NiFe-BTC-GNPs/NF, 2.5	$NiMO_4/MoO_2$/NF, 2.5	FAA-3-PK-130	1.85	~450	50	H_2O	72
238	FAA3-50	$NiMn_2O_4$, 3	Pt/C, 0.5	FAA-3	2.0	530	80	1 M KOH	-
	FAA3-50	$NiMn_2O_4$, 3	Pt/C, 0.5	FAA-3	1-1.8	~300	50	1 M KOH	1000
239	FAA-3-50	g-CN-CNF-800, 6	Pt/C, 0.4	Nafion	1.9	734	60	1 M KOH	-
240	FAA 3-PE	Ir black, 3	NiMo/X72, 5	Fumion FAA-3-SOLUT-10	1.9	1000	50	1 M KOH	-
	FAA 3-PE	Ir black, 3	Pt/C, 1	Fumion FAA-3-SOLUT-10	1.8	1000	50	1 M KOH	-
241	Fumasep FAA-3-PE-30	$Cu_{0.81}Co_{2.19}O_4$, -	Pt/C, 1	-	1.68	100	30	0.1 M KOH	100
242	FAA3-50	$NiCo_2O_4$, 1.2	Pt/C, 1	FAA3-50	1.8	303	50	6 M KOH	-
		$NiMn_2O_4$, 0.5	Pt/C, 1	FAA3-50	1.8	181	50	6 M KOH	-
243	FAA-3-50	IrO_2, 4	Pt /C, 0.4	FAA-3-Br	1.9	1500	70	1 M KOH	-
244	Selemion AMV	GO-NiO, -	Ni/Zn/S, -	-	1.9	513	80	5.36 M KOH	20
245	Selemion AMV	NiO, -	Pt, -	Proprietary ionomer	1.9	400	80	1 M KOH	-
246, 247, 248	Sustainion X37-50	$NiFe_2O_4$, 2	NiFeCo, 3 or 2	Sustainion X37-50 or Nafion	1.9	1000	60	1 M KOH	~2000
249	Sustainion® X37-50	NiMo-NH_3/H_2, 3	Fe-NiMo-NH_3/H_2, 3	Nafion	1.57	1000	80	1 M KOH	-
	Sustainion® X37-50	NiMo-NH_3/H_2, 3	Fe-NiMo-NH_3/H_2, 3	Nafion	1.69	500	20	1 M KOH	25

	Sustainion® X37-50	NiMo-NH_3/H_2, 3	Fe-NiMo-NH_3/H_2, 3	Nafion	1.52	50	20	1 M KOH	25
	Sustainion® X37-50	NiMo-N_2/H_2, 3	Fe-NiMo-N_2/H_2, 3	Nafion	1.68	1000	80	1 M KOH	-
	Sustainion® X37-50	NiMo-NH_3, 3	Fe-NiMo-NH_3, 3	Nafion	1.62	1000	80	1 M KOH	-
36	Sustainion ® X37-50	NiFe-LDH, 2.5	Pt/C, 1.3	Nafion	1.59	1000	80	1 M KOH	-
250	Sustainion® Grade T	$NiFe_2O_4$, 1.8	Raney Ni, 14.5	-	1.8	837	60	1 M KOH	-
	Sustainion ® Grade T	$NiFe_2O_4$, 1.8	Raney Ni, 14.5	-	1.83	1000	60	1 M KOH	12180
	Sustainion™ X37-50	$NiFe_2O_4$, 1.8	Raney Ni, 2.7	-	1.8	744	60	1 M KOH	-
	Sustainion™ X37-50	$NiFe_2O_4$, 1.8	Raney Ni, 2.7	-	1.85	1000	60	1 M KOH	~10100
251	X37-50 Grade T	CE-CCO, 3.8	Pt,1	-	1.8	1390	45	1 M KOH	
	X37-50 Grade T	CE-CCO, 3.8	Pt,1	-	1.66-1.74	500	45	1 M KOH	64
41	X37-50 Grade T	CCO-11, 10	Pt,1	PTFE	1.63-1.69	400	45	1 M KOH	100
	X37-50 Grade T	$Cu_{0.5}Co_{2.5}O_4$, 10	Pt,1	PTFE	1.8	1300	45	1 M KOH	-
252	X37-50 Grade T	$Ni_{0.75}$ $Fe_{2.25}O_4$, -	Pt, 1	PVDF	1.9	2000	45	1 M KOH	-
	X37-50 Grade T	$Ni_{0.75}$ $Fe_{2.25}O_4$, -	Pt, 1	PVDF	1.6-1.8	500	45	1 M KOH	21
	X37-50 Grade T	IrO_2, 4	Pt, 1	Nafion	1.8	870	45	1 M KOH	-
191	PTFE-Sustainion	Ni-Fe, 20	Ni-Fe, 20	PFTP-8/PFBP	2.0	620	60	1 M KOH	-

	PTFE-Sustainion	Ni-Fe, 20	Ni-Fe, 20	PFTP-8/PFBP	1.9-2.1 V	500	60	1 M KOH	240
	PFTP -13	Pt/C,0.5	IrO_2, 2	PFTP-8/PFBP-14	2	7680	80	1 M KOH	-
	PFTP -13	Pt/C,0.5	IrO_2, 2	PFTP-8/PFBP-14	2.2	500	60	1 M KOH	1100
	PFTP -8	Pt/C,0.5	IrO_2, 2	PFTP-8/PFBP-14	2.0	4880	80	1 M KOH	-
	x- PFTP	Pt/C,0.5	IrO_2, 2	PFTP-8/PFBP-14	2.0	3600	80	1 M KOH	-
	PFTP -13	Pt/C,0.5	IrO_2, 2	PFTP-8/PFBP-14	2.0	1800	80	H_2O	-
	PFTP -13	Ni-Fe, 20	Ni-Fe, 20	-	2.0	1600	80	1 M KOH	-
	PFTP -13	Ni-Fe, 20	Ni-Fe, 20	-	1.5	500	60	1 M KOH	1000
[253]	a Zirfon Perl 500 UTP (AGFA)	SS316L	Raney Ni	-	1.75	300	75	6 M KOH	720
[254]	Aemion™AF1-HNN8-50	Ir black, 3.5-3.8	Pt/C, 1	FAA-3	1.82	2000	60	1 M KOH	-
		Ir black, 3.5-3.8	Pt/C, 1	Aemion™ , AP1-HNN8	1.73-1.76V	500	50	0.1 M KOH	~17
	Aemion™AF1-HNN8-25	Ir black, 3.5-3.8	Pt/C, 1	Aemion™ , AP1-HNN8	1.68-1.73V	500	50	0.1 M KOH	~17
[121]	YAB membrane, Foma Co.	CoP NS, 5	CoP NS, 5	PTFE	1.74-1.78V	300	50	1 M KOH	~24
[255]	Commercial AEM	IrO_2, 3	Pt black, 3	-	1.87	200	35	1 wt.% $KHCO_3$	~190
		$Pb_2Ru_2O_{6.5}$, 3	Pt black, 3	-	1.75	200	35	1 wt.% $KHCO_3$	~190
**Other AEMs**									

256	AEM with quaternary ammonium	$Cu_{0.7}Co_{2.3}O_4$, 3	Pt/C, 1	-	1.8	1000	25	1 M KOH	-
257	Membrane from ITM power plc	$NiFe(OH)_2$, -	Pt, -	-	2.10 V to 2.25 V	1000	60	4 M NaOH	~240
258	in-house prepared APE	$Ni_{0.7}Co_{0.3}O_x$, 2	Pt/C, 1	AS-4	1.94 V to 2.05 V	100	50	1 wt.% $KHCO_3$	550

7 ADDITIONAL DATA TO STATE OF THE ART AEMWE SINGLE CELL TESTS

Table S20 provides additional experimental and operational information for the AEMWE single cell tests discussed in section 6. Table S20 complements Table S6 in the review paper. The study numbers shown in the two tables are identical.

Table S20. Experimental and operational data for State of the Art AEMWE single cell tests

This table provides supporting information to Table 6 presented in the review paper.

Study	Anode: PTL	Cathode: GDL	T [°C]	Feed mode	Ref.
1	Ti	Carbon paper	50	n/a	[259]
2	Ni foam	Carbon paper	80	Anode	190
3	NiMPL-PTL (stainless steel)	NiMPL-PTL (stainless steel)	60	Both	[260]
4	Ti foam	Carbon paper, untreated	50	cathode (first 2 h) then anode only	[229]
5	Platinized Ti	Carbon paper (SGL BC 29)	60	Anode	[185]
6	Platinized Ti	Carbon paper (SGL BC29)	80	Cathode	[184]
7	Ni foam	carbon paper (SGL 29AA)	80	Anode	[261]
8	Stainless steel (400 µm), on sintered Ti plate for back support	carbon paper (Toray 090)	55	anode and cathode	[262]
9	Ni foam	Ni foam	60	Anode	[204]
10a	stainless steel	Ni fiber paper	60	anode and cathode	[250]
10b	stainless steel	Ni fiber paper	60	anode and cathode	[250]
11	Ni foam	Ni foam	60	Anode	[191]
12	stainless steel	carbon paper	70	Anode and cathode	[263]

13	Ni	Ni	60	anode and cathode	[246]
14	Ni foam	carbon paper (SGL 29AA)	80	Anode	[261]
15	Titanium felt	carbon paper (SGL 38 BC)	60	Anode	191
16	stainless steel	stainless steel	60	anode and cathode	[206]
17	Ni foam	Ni foam	60	Anode	[191]
18	Ni foam	Ni foam	50	n/a	[223]
19	Ni foam	Ni foam	50	n/a	[215]
20	Ni foam	Ni foam	50	n/a	[194]
21	Ni foam (pore size 580 mm)	Ni foam (pore size 580 mm)	50	anode and cathode	[213]
22	Porous Ni	C-cloth coated with hydrophobic MPL[1], on Ni	43	Anode	[230]
23	Porous Ti, Pt plated	PTFE treated carbon paper	50	Anode	[258]
24a	Ni foam	carbon paper	60	Anode	[234]
24b	Ni foam	carbon paper	60	Anode	[234]

[1]: MPL: Microporous layer

8 PROTOCOL FOR SINGLE CELL AEMWE TESTING

In the absence of defined testing protocols it is difficult to reliably reproduce and compare single cell AEMWE performance data between studies. Furthermore, the lack of baseline materials for AEM systems contributes to the complexity of comparing results across studies. Therefore, in this section a testing protocol to evaluate the performance and durability measurements of single cell AEMWEs is proposed.

The conditioning of the membrane usually entails soaking the membrane in 1-3 M KOH for 24- 48 h according to the manufacturers guidelines.[264] This allows for the exchange of the counter ion (typically I^- or Cl^-) with OH^- before assembling the cell. Complete OH^- exchange may require the use of different hydroxide concentrations

depending on how strongly the hydrated ions interact with the charged end-groups of the AEM.[265] A similar behaviour is expected for the ionomers in the catalyst layer, but these ionomers are seldomly pre-doped before electrolyzer operation and a steady improvement in cell voltage, in the "conditioning" phase of electolyzer operation, may well be attributed to the exchange of ionomer with KOH.[266,262,267]

The MEA also needs to be preconditioned in the AEMWE cell prior to performance measurements. First a stable cell temperature is established, while circulating pure water or supporting electrolyte to the anode and/or cathode. Usually after one hour, conditioning is applied as follows: i) Either by current stepping (typically 100 mA/cm² to 1A/cm², in 100 mA steps, for holding time 2-5 min. at each step) or by ii) applying a constant voltage (typically in the range of 1.6-2 V) until the current is stabilized. A steady state is typically reached after 30-60min. This step also allows to identify possible pinholes in the MEA, before starting with the performance assessment of the electrolyzer cell.

Lindquist et al. recently showed the impact of insufficient cell conditioning on the low-current-density performance of polarization curves (up to 200 mV) obtained before and after conditioning for commercial PiperION in water feed.[262]

Some studies have been found to condition AEMWE cells in alkaline electrolyte, followed by purging with pure water and measuring performance thereafter.[185,261] Lindquist et al. found that insufficient purging with water after conditioning in KOH could result in an enhanced performance attributed to residual KOH.[262] They recorded linear sweep voltammograms (LSVs) accompanied with conductivity measurements of the effluent electrolyte to conduct the study.

Table S21. Conditioning and testing protocols for AEMWE single cells summarized from the literature

Pre-cell conditioning of membrane	KOH doping, exchanging of $I^-/Br^-/Cl^-$ groups	
Activation of catalyst	Investigated studies on GDL scale[190]	
Pre-conditioning of cell	i) Current steps, e.g. 100 mA/cm² to 1 A/cm², in 100 mA steps, for holding time 2-5 min at each step.[262]<br>ii) Constant current of 0.2 A/cm² applied for 30 min, while recording iV-curves before and after to compare steady state for recorded cell voltage.[260]	Compare polarization curves before and after cell conditioning step to confirm conditioning is complete.<br><br>Once steady cell voltage is achieved for applied current steps, conditioning is considered effective.<br><br>If still unstable or the measured voltage difference between iV-curves is > 50 mV, the conditioning step could be repeated. Once the cell voltage keeps increasing it could be a sign the cell is unstable and either membrane or ionomer degradation has started.

Performance assessment	Polarization curves (voltage measured at current density increments) are recorded, supported with EIS	j_{max} measured up to the cut-off cell voltage of 2.2-2.4 V. This is to limit the probability of oxidation/corrosion of cell components at higher cell potentials. (PEWME at 1.8 and 2.0 V).[268]
Short and long term stability	Galvanostatic, constant current applied for a minimum of 100 hours.<br>H_2O feed: 0.2-0.5 A/cm²<br>KOH/K_2CO_3: 0.3, 0.5 or 1 A/cm²<br><br>Alternatively, intermittent operation (e.g. voltage cycling) allows for cycling between a high and low cell voltages with varying rest times.[269,238]	The voltage degradation rate over time is calculated (μV/h) for comparison between studies
Post-characterization/ex-situ tests	Most commonly performed to assess the degradation of electrode materials. This includes SEM-EDX measurements, XRD or XPS to establish a change in structure or intensity of characteristic signals as compared to pristine samples.[190,262]	

ABBREVIATIONS AND SYMBOLS

List of abbreviations and symbols used throughout the manuscript and SI:

a: intercept in Tafel plot

A: surface area

ads: adsorbed

AEI: anion exchange ionomer

AEM: anion exchange membrane

AEMFC: anion exchange membrane fuel cell

AEMWE: anion exchange membrane water electrolyzer

AEP: anion exchange polymers

AFM: atomic force microscopy

ALD: atomic layer deposition

AMS: aqueous model system

ASU: 6-azonia-spiro [5.5] undecane

at.: atomic

b: Tafel-slope value [mV/dec]

BP: bipolar plates

BPM: bipolar membranes

BPN: quaternized biphenylene ionomer

BTMA: benzyltrimethylammonium

C_{dl}: double layer capacitance

CL: catalyst layer

cm^2_{geom}: geometrical electrode surface area in cm^2;

CO_{ads}: adsorbed CO

CV: cyclic voltammogram

CVD: chemical vapor deposition

Cu_{upd}: underpotential deposition of Copper

E_{act}: activation energy

CAPEX: capital investment cost

CCM: catalyst coated membrane

CEM: cation exchange membrane

CCS: catalyst coated substrate

CNT: carbon nanotubes

DABCO: 1,4-diazabicyclo[2.2.2]octane

dec: decade

DFT: density functional theory

dl: double layer

DMP: N,N-dimethylpiperidinium

E_{act}: activation energy

E_{an}: anode potential

E_{cat}: cathode potential

E_{Cell}: cell potential

E^o: reversible potential

E^o_{rev}: standard reversible potential

E^o_{th}: thermoneutral voltage

ECSA: electrochemical surface area

EIS: electrochemical impedance spectroscopy

EO: ethylene oxide

EQCM: electrochemical quartz crystal microbalance

ES: energy storage

F: Faraday's constant [96485 C/mol]

FC: fuel cell

FE-SEM: field emission scanning electron microscope

GC: gas chromatography

GDE: gas diffusion electrode

GDL: gas diffusion layer

$H_{ads/des}$: adsorbed/desorbed molecular hydrogen

HBE: hydrogen binding energy

HER: hydrogen evolution reaction

HOR: hydrogen oxidation reaction

HFR: high frequency resistance

HHV: high heating value

IC: initial capital cost (initial cost of the water electrolysers)

ICP-MS: inductively coupled plasma mass spectrometry

ICP-OES: inductively coupled optical emission spectrometry

IEC: ion exchange capacity

IM: imidazolium

IPNs: interpenetrating polymer networks

IR: voltage drop (current multiplied by the resistance)

iV: current-potential curves

j: current density

j_{crt}: critical current density

j_{int}: intrinsic current density

j_{mass}: mass current density

j_{max}: maximum current density

j_o: exchange current density

$j_{o-int:}$ intrinsic exchange current density

j_{o-mass}: mass exchange current density

LDH: layered double helix

LSVs: linear sweep voltammograms

LT: lifetime

MEA: membrane electrode assembly

M_{H2}: molecular hydrogen weight

MMT: million metric tons

MPL: micro-porous layer

MS: mass-spectrometry

n: number of catalyst atoms

NC: nano-carbons

n_e: number of electrons

np: nano-particles

N_{th}: transient cycle number

OER: oxygen evolution reaction

OPEX: operating investment cost

ORR: oxygen reduction reaction

P: Pressure

PBI: polybenzimidazole

pc: polycrystalline

PEMWE: proton exchange membrane water electrolyser

PES: polyethersulfone

PFOTFPh: poly(fluorene-alt-tetrafluorophenylene)

PGM: platinum group metal

PTFE: polytetrafluoroethylene

PTL: porous transfer layer

PNB: polynorbornene

PPO: polypropylene oxide

PSF: polysulfone

QA: quaternary amines

QP: quaternary phosphonium

R: gas constant [8.314 $kg \cdot m^2 \cdot s^{-2} \cdot K^{-1} \cdot mol^{-1}$] or resistance

R_{Cell}: cell resistance

RDE: rotating ring disc electrode

RH: relative humidity

RHE: reversible hydrogen electrode

SHE: standard hydrogen electrode

S: geometrical electrode surface area

SEM: scanning electron microscopy

SFC: scanning flow cell

SI: supporting information

SMR: steam methane reforming

SoA: State of the Art

SS: stainless steel

T: Temperature

T_g: glass temperature

TEM: transmission electron microscopy

TM: transmission metal

TOF: turn over frequency

T.S.: Tafel-slope

upd: under-potential deposition

WE: water electrolyzer

wt.: weight

W_u: water uptake

XRD: x-ray diffraction

XPS: X-ray photon spectroscopy

Symbols:

*: surface adsorbed species

λ: number of water molecules per OH^-

η: overpotential

Ω: resistance / ohm

REFERENCES

(1) Zhao, Z.; Gao, W.; Xue, W.; Liu, Z.; Huang, J.; Pan, X.; Huang, Yu. Surface-Engineered PtNi-O Nanostructure with Record-High Performance for Electrocatalytic Hydrogen Evolution Reaction. *JACS* **2018**, *140*, 9046−9050.

(2) Li, Y.; Pei, W.; He, J.; Liu, K.; Qi, W.; Gao, X.; Zhou, S.; Xie, H.; Ying, K.; Gao, Y. Hybrids of PtRu Nanoclusters and Black Phosphorus Nanosheets for Highly Efficient Alkaline Hydrogen Evolution Reaction. *ACS Catal*. **2019**, *9*, 10870−10875.

(3) Wang,T.; Wang, M.; Yang, H.; Xu, M.; Zuo, C.; Feng,K.; Xie, M.; Deng, J.; Zhong, J.; Zhou, W.; Cheng, T. and Li, Y. Weakening hydrogen adsorption on nickel via interstitial nitrogen doping promotes bifunctional hydrogen electrocatalysis in alkaline solution. *Energy Environ. Sci.* **2019**, *12*, 3522−3529.

(4) Zhang, F.; Zhu, Y.; Chen, Y.; Lu, Y.; Lin, Q.; Zhang, L.; Tao, S.; Zhang, X. and Wang, H. RuCo alloy bimodal nanoparticles embedded in N-doped carbon: a superior pH-universal electrocatalyst outperforms benchmark Pt for the hydrogen evolution reaction. J. *Mater. Chem. A* **2020**, *8*, 12810−12820.

(5) Li, B.; Guo, L.; Wu, F.; Peng, Y.; Lu, E. J.; Smart, T. J.; Wang, N.; Finfrock, Y. Z.; Morris, D.; Zhang, P.; Li, N.; Gao, P.; Ping, Y.; Chen, S. Ruthenium atomically dispersed in carbon outperforms platinum toward hydrogen evolution in alkaline media, *Nat. Commun*. **2019**, *10*, 631−742.

(6) Peng, Z.; Liu, J.; Hu, B.; Yang, Y.; Guo, Y.; Li, B.; Li, L.; Zhang, Z.Vui, B.; He, L. and Du, M. Surface Engineering on Nickel–Ruthenium Nanoalloys Attached Defective Carbon Sites as Superior Bifunctional Electrocatalysts for Overall Water Splitting. ACS *Appl. Mater. Interfaces* **2020**, *12*, 13842−13851

(7) Abbas, S.A.; Kim, S-H.; Iqbal. M.I.; Muhammad. S.; Yoon, W-S.; Jung, K-D.: Synergistic effect of nano-Pt and Ni spine for HER in alkaline solution: hydrogen spillover from nano-Pt to Ni spine. *Sci. Reports* **2018**, *8*, 2986−2995.

(8) Cao, Z.; Chen, Q.; Zhang, J.; Li, H.; Jiang, Y.; Shen, S.; Fu, G.; Lu, B.; Xie, Z.; Zheng, L. Platinum-nickel alloy excavated nano-multipods with hexagonal close-packed structure and superior activity towards hydrogen evolution reaction. *Nat. Commun*. **2017**, *8*, 15131−15138.

(9) Xu, C.; Ming, M.; Wang, Q.; Yang, C.; Fan, G.; Wang, Y.; Gao, D.; Bia, J.; Zhang, Y. Facile synthesis of effective Ru nanoparticles on carbon by adsorption-low temperature pyrolysis strategy for hydrogen evolution. *J. Mater. Chem. A* **2018**, *6*, 14380−14386.

(10) Mahmood, J.; Li, F.; Jung, S.-M.; Okyay, M. S.; Ahmad, I.; Kim, S.-J.; Park, N.; Jeong H.Y. and Baek J.-B. An efficient and pH-universal ruthenium-based catalyst for the hydrogen evolution reaction. *Nat. Nanotech.* **2017**, *12*, 441–446.

(11) Yin, H.; Zhao, S.; Zhao, K.; Muqsit, A.; Tang, H.; Chang, L.; Zhao, H.; Gao, Y.; Tang, Z. Ultrathin platinum nanowires grown on single-layered nickel hydroxide with high hydrogen evolution activity. *Nat. Commun*. **2015**, *6*, 6430−6438.

(12) Wang, P.; Jiang, K.; Wang, G.; Yao, J.; Huang, X. Phase and Interface Engineering of Platinum–Nickel Nanowires for Efficient Electrochemical Hydrogen Evolution. *Angew. Chem. Int. Ed.* **2016**, *55*, 12859–12863.

(13) Wang, Z.; Ren, X.; Luo, Y.; Wang, L.; Cui, G.; Xie, F.; Wang, H.; Xie, Y. and Sun, X. An ultrafine platinum–cobalt alloy decorated cobalt nanowire array with superb activity toward alkaline hydrogen evolution. *Nanoscale*. **2018**, *10*, 12302–12307.

(14) Chen, X.; Wan, J.; Wang, J.; Zhang, Q.; Gu, L.; Zheng,L.; Wang, N.; Yu, R. Atomically Dispersed Ruthenium on Nickel Hydroxide Ultrathin Nanoribbons for Highly Efficient Hydrogen Evolution Reaction in Alkaline Media. *Adv. Mater.* **2021**, *33*, 2104764−2104773.

(15) Xing, Z.; Han, C.; Wang, D.; Li, Q.; Yang, X. Ultrafine Pt Nanoparticle-Decorated $Co(OH)_2$ Nanosheet Arrays with Enhanced Catalytic Activity toward Hydrogen Evolution. *ACS Catal*. **2017**, *7*, 7131−7135.

(16) Wang, P.; Zhang, X.; Zhang, J.; Wan, S.; Guo, S.; Lu, G.; Yao, J.; Huang, X. Precise tuning in platinum-nickel/nickel sulfide interface nanowires for synergistic hydrogen evolution catalysis. *Nat. Commun*. **2017**, *8*, 14580−14589.

(17) Wang, J.; Wei, Z.; Mao, S.; Li, H.; Wang, Y. Highly uniform Ru nanoparticles over N-doped carbon: pH and temperature-universal hydrogen release from water reduction. *Energy Environ. Sci*. **2018**, *11*, 800−806.

(18) Pu, Z.; Amiinu, I. S.; Kou, Z.; Li, W.; Mu, S. RuP_2-Based Catalysts with Platinum-like Activity and Higher Durability for the Hydrogen Evolution Reaction at All pH Values. *Angew. Chem. Int. Ed*. **2017**, *129*, 11717−11722.

(19) Mao, J.; He, C.-T.; Pei, J.; Chen, W.; He, D.; He, Y.; Zhuang, Z.; Chen, C.; Peng, Q.; Wang, D. and Li, Y. Accelerating water dissociation kinetics by isolating cobalt atoms into ruthenium lattice. *Nat. Commun*. 2018, *9*, 4958−4966.

(20) Su, J.; Yang, Y.; Xia, G.; Chen, J.; Jiang, P.; and Chen, Q. Ruthenium-cobalt nanoalloys encapsulated in nitrogen-doped graphene as active electrocatalysts for producing hydrogen in alkaline media. *Nat. Commun.* **2017**, *8*, 14969−14979.

(21) Xu, J.; Liu, T.; Li, J. Li, B.; Liu, Y.; Zhang, B.; Xiong, De.; Amorim, I.; Li. W.; Liu, L. Boosting the hydrogen evolution performance of ruthenium clusters through synergistic coupling with cobalt phosphide. *Energy Environ. Sci.* **2018**, *11*, 1819−1827.

(22) Li, M.; Wang, H.; Zhu, W.; Li, W.; Wang, C.; Lu, X. RuNi Nanoparticles Embedded in N-Doped Carbon Nanofibers as a Robust Bifunctional Catalyst for Efficient Overall Water Splitting. *Adv. Sci.* 2020, *7*, 1901833−1901843.

(23) Xu, Y.; Yin, S.; Li, C.; Deng, K.; Xue, H.; Li, X.; Wang, H.; Wang, L. Low-ruthenium-content NiRu nanoalloys encapsulated in nitrogen-doped carbon as highly efficient and pH-universal electrocatalysts for the hydrogen evolution reaction. *J. Mater. Chem. A* **2018**, *6*, 1376−1381.

(24) Liu, H.; Yan, Z.; Chen, X.; Li, J.; Zhang, L.; Liu, F.; Fan, G.; Cheng, F. Electrodeposition of Pt-Decorated $Ni(OH)_2/CeO_2$ Hybrid as Superior Bifunctional Electrocatalyst for Water Splitting. *Research*. **2020**, *1*, 9068270−9068281.

(25) Jiang, P.; Chen, J.; Wang, C.; Yang, K.; Gong, S.; Liu, S.; Lin, Z.; Li, M.; Xia, G.; Yang, Y.; Su, J.; Chen, Q. Tuning the Activity of Carbon for Electrocatalytic Hydrogen Evolution via an Iridium-Cobalt Alloy Core Encapsulated in Nitrogen-Doped Carbon Cages. *Adv. Mater.* **2018**, *30*, 1705324−1705334.

(26) Yan, X.; Tian, L.; He, M.; Chen, X. Three-Dimensional Crystalline/Amorphous Co/Co_3O_4 Core/Shell Nanosheets as Efficient Electrocatalysts for the Hydrogen Evolution Reaction. *Nano Lett.* **2015**, *15*, 6015–6021.

(27) Jiang, N.; You, B.; Sheng, M.; Sun, Y.; Electrodeposited Cobalt-Phosphorous-Derived Films as Competent Bifunctional Catalysts for Overall Water Splitting. *Angew. Chem. Int. Ed.* **2015**, *54*, 6251–6254.

(28) Tian, J.; Liu, Q.; Asiri, A. M.; Sun, X.; Self-Supported Nanoporous Cobalt Phosphide Nanowire Arrays: An Efficient 3D Hydrogen-Evolving Cathode over the Wide Range of pH 0–14. *JACS* **2014**, *136*, 7587–7590.

(29) Liu, Y.; Yu, G.; Li, G. D.; Sun, Y.; Asefa, T.; Chen, W.; Zou, X. Coupling Mo_2C with Nitrogen-Rich Nanocarbon Leads to Efficient Hydrogen-Evolution Electrocatalytic Sites. *Angew. Chem. Int. Ed.* **2015**, 54, 10752–10757.

(30) Xiao, P.; Sk, M. A.; Thia, L.; Ge, X.; Lim, R. J.; Wang, J.-Y.; Lima, K. H.; Wang, X. Molybdenum phosphide as an efficient electrocatalyst for the hydrogen evolution reaction. *Energy Environ. Sci.* **2014**, *7*, 2624–2629.

(31) Ma, L.; Ting, L. R. L.; Molinari, V.; Giordano, C.; Yeo, B. S. Efficient hydrogen evolution reaction catalyzed by molybdenum carbide and molybdenum nitride nanocatalysts synthesized via the urea glass route. *J. Mater. Chem. A* **2015**, *3*, 8361–8368.

(32) Vrubel, H.; Hu, X.; Molybdenum Boride and Carbide Catalyze Hydrogen Evolution in both Acidic and Basic Solutions. *Angew. Chem. Int. Ed.* **2012**, *51*, 12703–12706.

(33) Feng, L. L.; Yu, G.; Wu, Y.; Li, G. D.; Li, H.; Sun, Y.; Asefa, T.; Chen, W. High-index faceted Ni_3S_2 nanosheet arrays as highly active and ultrastable electrocatalysts for water splitting. *JACS* **2015**, *137*, 14023–14026.

(34) Laursen, A. B.; Patraju, K. R.; Whitaker, M. J.; Retuerto, M.; Sarkar, T.; Yao, N.; K. V. Ramanujachary, K. V.; Greenblatt, M.; Dismukes, G. C. Nanocrystalline Ni_5P_4: a hydrogen evolution electrocatalyst of exceptional efficiency in both alkaline and acidic media. *Energy Environ. Sci.* **2015**, *8*, 1027–1034.

(35) Chen, Z. J.; Cao, G. X.; Gan, L. Y.; Dai, H.; Xu, N.; Zang, M-J.; Dai, H-B.; Wu, H.; Wang, P. Highly Dispersed Platinum on Honeycomb-like NiO@Ni Film as a Synergistic Electrocatalyst for the Hydrogen Evolution Reaction. *ACS Catal.* **2018**, *8*, 8866–8872.

(36) Koshikawa, H.; Murase, H.; Hayashi, T.; Nakajima, K.; Mashiko, H.; Shiraishi, S. Tsuji, Y. Single Nanometer-Sized NiFe-Layered Double Hydroxides as Anode Catalyst in Anion Exchange Membrane Water Electrolysis Cell with Energy Conversion Efficiency of 74.7% at 1.0 A cm^{-2}. *ACS Catal.* **2020**, *10*, 1886–1893.

(37) Xu, D.; Stevens, M. B.; Cosby, M. R.; Oener, S. Z.;Smith, A. M.; Enman, L. J.; Ayers, K. E.; Capuano, C. B.; Renner, J. N.; Danilovic, N. et al. Earth-Abundant Oxygen Electrocatalysts for Alkaline Anion-Exchange-Membrane Water Electrolysis: Effects of Catalyst Conductivity and Comparison with Performance in Three-Electrode Cells. *ACS Catal.* **2019**, *9*, 1, 7–15.

(38) Song, F.; Hu, X. Exfoliation of layered double hydroxides for enhanced oxygen evolution catalysis. *Nat. Commun.* **2014**, *5*, 4477−4486.

(39) Trotochaud, L.; Ranney, J. K.; Williams, K. N.; Boettcher, S. W. Solution-cast metal oxide thin film electrocatalysts for oxygen evolution. *JACS* **2012**, *134*, 17253−17261.

(40) McCrory, C. C. L.; Jung, S.; Peters, J. C. and Jaramillo, T. F. Benchmarking heterogeneous electrocatalysts for the oxygen evolution reaction. *JACS* **2013**, *135*, 16977–16987.

(41) Jang, M. J.; Yang, J.; Lee, J.; Park, Y. S.; Jeong, J.; Park, S. M.; Jeong, J.-Y.; Yin, Y.; Seo, M.-H.; Choi, S. M.; et al. Superior performance and stability of anion exchange membrane water electrolysis: pH-controlled copper cobalt oxide nanoparticles for the oxygen evolution reaction. *J. Mater. Chem. A* **2020**, *8*, 4290–4299.

(42) Cui, X.; Ren, P.; Deng, D.; Deng, J. and Bao, X. Single layer graphene encapsulating non-precious metals as high-performance electrocatalysts for water oxidation. *Energy Environ.Sci.* **2016**, *9*, 123−129.

(43) Song, F.; and Hu, X. Ultrathin Cobalt–Manganese Layered Double Hydroxide Is an Efficient Oxygen Evolution Catalyst. *JACS* **2014**, *136*, 16481–16484.

(44) Zheng, J.; Chen, X.; Zhong, X.; Li, S.; Liu, T.; Zhuang, G.; Li, X.; Deng, S.; Mei, D.; Wang, J.-G. Hierarchical Porous NC@CuCo Nitride Nanosheet Networks: Highly Efficient Bifunctional Electrocatalyst for Overall Water Splitting and Selective Electrooxidation of Benzyl Alcohol. *Adv. Funct. Mater.* **2017**, *27*, 1704169−1704180.

(45) Chauhan, M.; Reddy, K. P.; Gopinath, C. S.; Deka, S. Copper Cobalt Sulfide Nanosheets Realizing a Promising Electrocatalytic Oxygen Evolution Reaction. *ACS Catal.* **2017**, *7*, 5871–5879.

(46) Yu, X.; Zhang, S.; Li, C.; Zhu, C.; Chen, Y.; Gao, P.; Qi, L.; Zhang, X.. Hollow CoP nanoparticle/N-doped graphene hybrids as highly active and stable bifunctional catalysts for full water splitting. *Nanoscale* **2016**, *8*, 10902–10907.

(47) Karkera, G.; Sarkar, T.; Bharadwaj, M. D.; Prakash, A. S. Design and Development of Efficient Bifunctional Catalysts by Tuning the Electronic Properties of Cobalt–Manganese Tungstate for Oxygen Reduction and Evolution Reactions. *ChemCatChem.* **2017**, *9*, 3681–3690.

(48) Tang, Y.-J.; Liu, C.-H.; Huang, W.; Wang, X.-L.; Dong, L. –Z.; Li, S.-L.; Lan, Y.-Q. Bimetallic Carbides-Based Nanocomposite as Superior Electrocatalyst for Oxygen Evolution Reaction. *ACS Appl. Mater. Interfaces* **2017**, *9*, 16977–16985.

(49) He, K.; Cao, Z.; Liu, R.; Miao, Y.; Ma, H.; Ding, Y. In situ decomposition of metal-organic frameworks into ultrathin nanosheets for the oxygen evolution reaction. *Nano Res*. **2016**, *9*, 1856–1865.

(50) Dong, C.; Kou, T.; Gao, H.; Peng, Z.; Zhang, Z. Eutectic-Derived Mesoporous Ni-Fe-O Nanowire Network Catalyzing Oxygen Evolution and Overall Water Splitting. *Adv. Energy Mater.* **2018**, *8*, 1701347−1701355.

(51) Tan, Y.; Zhu, F.; Wang, H.; Tian, Y.; Hirata, A.; Fujita, T.; Chen, M. Noble-Metal-Free Metallic Glass as a Highly Active and Stable Bifunctional Electrocatalyst for Water Splitting. *Adv. Mater. Interfaces* **2017**, *4*, 1601086−1601082.

(52) Yu, L.; Zhou, H.; Sun, J.; Qin, F.; Yu, F.; Bao, J.; Yu, Y.; Chen, S.; Ren, Z. Cu nanowires shelled with NiFe layered double hydroxide nanosheets as bifunctional electrocatalysts for overall water splitting. *Energy Environ. Sci.* **2017**, *10*, 1820–1827.

(53) Yu, X.; Zhang, M.; Yuan, W.; Shi, G. A high-performance three-dimensional Ni–Fe layered double hydroxide/graphene electrode for water oxidation. *J. Mater. Chem. A* **2015**, *3*, 6921–6928.

(54) Jin, H.; Mao, S.; Zhan, G.; Xu, F.; Bao, X.; Wang, Y. Fe incorporated α-$Co(OH)_2$ nanosheets with remarkably improved activity towards the oxygen evolution reaction. *J. Mater. Chem. A* **2017**, *5*, 1078–1084.

(55) Feng, Y.; Zhang, H.; Zhang, Y.; Li, X.; Wang, Y. Ultrathin Two-Dimensional Free-Standing Sandwiched NiFe/C for High-Efficiency Oxygen Evolution Reaction. *ACS Appl. Mater. Interfaces* **2015**, *7*, 9203–9210.

(56) Ma, Y.; Dai, X.; Liu, M.; Yong, J.; Qiao, H.; Jin, A.; Li, Z.; Huang, X.; Wang, H.; Zhang, X. Strongly Coupled FeNi Alloys/$NiFe_2O_4$@Carbonitride Layers-Assembled Microboxes for Enhanced Oxygen Evolution Reaction. *ACS Appl. Mater. Interfaces* **2016**, *8*, 34396–34404.

(57) Tao, Z.; Wang, T.; Wang, X.; Zheng, J.; Li, X. MOF-Derived Noble Metal Free Catalysts for Electrochemical Water Splitting. *ACS Appl. Mater. Interfaces* **2016**, *8*, 35390–35397.

(58) Zhang, X.; Xu, H.; Li, X.; Li, Y.; Yang, T.; Liang, Y. Facile Synthesis of Nickel–Iron/Nanocarbon Hybrids as Advanced Electrocatalysts for Efficient Water Splitting. *ACS Catal.* **2016**, *6*, 580–588.

(59) Candelaria, S. L.; Bedford, N. M.; Woehl, T. J.; Rentz, N. S.; Showalter, A. R.; Pylypenko, S.; Bunker , B. A.; Lee, S.; Reinhart, B.; Ren, Y. et al. Multi-Component Fe–Ni Hydroxide Nanocatalyst for Oxygen Evolution and Methanol Oxidation Reactions under Alkaline Conditions. *ACS Catal.* **2017**, *7*, 365–379.

(60) Yang, H.; Liu, Y.; Luo, S.; Zhao, Z.; Wang, X,; Luo, Y.; Wang, Z.; Jin, J.; Ma, J. Lateral-Size-Mediated Efficient Oxygen Evolution Reaction: Insights into the Atomically Thin Quantum Dot Structure of $NiFe_2O_4$. *ACS Catal.* **2017**, *7*, 5557–5567.

(61) Ma, W.; Ma, R.; Wang, C.; Liang, J.; Liu, X.; Zhou, K.; Sasaki, T. A Superlattice of Alternately Stacked Ni–Fe Hydroxide Nanosheets and Graphene for Efficient Splitting of Water. *ACS Nano* **2015**, *9*, 1977–1984.

(62) Jia, X.; Zhao, Y.; Chen, G.; Shang, L.; Shi, R.; Kang, X.; Waterhouse, G. I. N.; Wu, L.-Z.; Tung, C.-H.; Zhang, T. Ni_3FeN Nanoparticles Derived from Ultrathin NiFe-Layered Double Hydroxide Nanosheets: An Efficient Overall Water Splitting Electrocatalyst. *Adv. Energy Mater.* **2016**, *6*, 1502585−1502590.

(63) Nai, J.; Lu, Y.; Yu, L.; Wang, X.; Lou, X. W. (David), Formation of Ni–Fe Mixed Diselenide Nanocages as a Superior Oxygen Evolution Electrocatalyst. *Adv. Mater.* **2017**, *29*, 1703870−1703877.

(64) Qian, M.; Cui, S.; Jiang, D.; Zhang, L.; Du, P. Highly Efficient and Stable Water-Oxidation Electrocatalysis with a Very Low Overpotential using FeNiP Substitutional-Solid-Solution Nanoplate Arrays. *Adv. Mater.* **2017**, *29*, 1704075−1704080.

(65) Tang, C.; Wang, H.-S.; Wang, H.-F.; Zhang, Q.; Tian, G.-L.; Nie, J.-Q.; Wei, F. Spatially Confined Hybridization of Nanometer-Sized NiFe Hydroxides into Nitrogen-Doped Graphene Frameworks Leading to Superior Oxygen Evolution Reactivity. *Adv. Mater.* **2015**, *27*, 4516–4522.

(66) Long, X.; Li, J.; Xiao, S.; Yan, K.; Wang, Z.; Chen, H.; Yang, S. A Strongly Coupled Graphene and FeNi Double Hydroxide Hybrid as an Excellent Electrocatalyst for the Oxygen Evolution Reaction. *Angew. Chem. Int. Ed.* **2014**, *53*, 7584–7588.

(67) Kuai, L.; Geng, J.; Chen, C.; Kan, E.; Liu, Y.; Wang, Q.; Geng, B. A reliable aerosol-spray-assisted approach to produce and optimize amorphous metal oxide catalysts for electrochemical water splitting. *Angew. Chem. Int. Ed.* **2014**, *53*, 7547–7551.

(68) Lu, Z.; Qian, L.; Tian, Y.; Li, Y.; Sun, X.; Duan, X. Ternary NiFeMn layered double hydroxides as highly-efficient oxygen evolution catalysts. *Chem. Commun.* **2016**, *52*, 908–911.

(69) Chen, H.; Huang, X.; Zhou, L.-J.; Li, G.-D.; Fan, M.; Zou, X. Electrospinning Synthesis of Bimetallic Nickel–Iron Oxide/Carbon Composite Nanofibers for Efficient Water Oxidation Electrocatalysis. *ChemCatChem*. **2016**, *8*, 992–1000.

(70) Dionigi, F.; Reier, T.; Pawolek, Z.; Gliech, M.; Strasser, P. Design Criteria, Operating Conditions, and Nickel–Iron Hydroxide Catalyst Materials for Selective Seawater Electrolysis. *ChemSusChem* **2016**, *9*, 962–972.

(71) Wang, L.; Huang, X.; Xue, J. Graphitic Mesoporous Carbon Loaded with Iron–Nickel Hydroxide for Superior Oxygen Evolution Reactivity. *ChemSusChem* **2016**, *9*, 1835–1842.

(72) Weng, B.; Xu, F.; Wang, C.; Meng, W.; Grice, C. R.; Yan, Y. A layered Na1−xNiyFe1−yO_2 double oxide oxygen evolution reaction electrocatalyst for highly efficient water-splitting. *Energy Environ. Sci.* **2017**, *10*, 121–128.

(73) Jung, S.; McCrory, C.C.L.; Ferrer, I.M.; Peters, J.C.; Jaramillo, T.F. Benchmarking nanoparticulate metal oxide electrocatalysts for the alkaline water oxidation reaction. *J. Mater. Chem. A* **2016**, *4*, 3068−3076.

(74) Xie, C.; Wang, Y.; Hu, K.; Tao, L.; Huang, X.; Huo, J.; Wang, S. In situ confined synthesis of molybdenum oxide decorated nickel–iron alloy nanosheets from MoO42− intercalated layered double hydroxides for the oxygen evolution reaction. *J. Mater. Chem. A* **2017**, *5*, 87–91.

(75) Zhong, H.; Tian, R.; Gong, X.; Li, D.; Tang, P.; Alonso-Vante, N.; Feng, Y. Advanced bifunctional electrocatalyst generated through cobalt phthalocyanine tetrasulfonate intercalated Ni2Fe-layered double hydroxides for a laminar flow unitized regenerative micro-cell. *J. Power Sources* **2017**, *361*, 21–30.

(76) Feng, Y.; Yu, X.-Y.; Paik, U. N-doped graphene layers encapsulated NiFe alloy nanoparticles derived from MOFs with superior electrochemical performance for oxygen evolution reaction. *Sci. Rep.* **2016**, *6*, 34004−34012.

(77) Lu, Z.; Xu, W.; Zhu, W.; Yang, Q.; Lei, X.; Liu, J.; Li, Y.; Sun, X.; Duan, X. Three-dimensional NiFe layered double hydroxide film for high-efficiency oxygen evolution reaction. *Chem. Commun.* **2014**, *50*, 6479–6482.

(78) Louie, M. W.; Bell, A. T. An Investigation of Thin-Film Ni–Fe Oxide Catalysts for the Electrochemical Evolution of Oxygen. *JACS.* **2013**, 135, 12329–12337.

(79) Gong, M.; Li, Y.; Wang, H.; Liang, Y.; Wu, J. Z.; Zhou, J.; Wang, J.; Regier, T.; Wei, F.; Dai, H. An Advanced Ni–Fe Layered Double Hydroxide Electrocatalyst for Water Oxidation. *JACS* **2013,** *135*, 8452–8455.

(80) Zhu, X.; Tang, C.; Wang, H.-F.; Zhang, Q.; Yang, C.; Wei, F. Dual-sized NiFe layered double hydroxides in situ grown on oxygen-decorated self-dispersal nanocarbon as enhanced water oxidation catalysts. *J. Mater. Chem. A* **2015**, *3*, 24540–24546.

(81) Xia, D.; Zhou, L.; Qiao, S.; Zhang, Y.; Tang, D.; Liu, J.; Huang, H.; Liu, Y.; Kang, Z. Graphene/Ni–Fe layered double-hydroxide composite as highly active electrocatalyst for water oxidation. *Mater. Res. Bull.* **2016**, *74*, 441–446.

(82) Han, N.; Zhao, F.; Li, Y. Ultrathin nickel–iron layered double hydroxide nanosheets intercalated with molybdate anions for electrocatalytic water oxidation. *J. Mater. Chem. A* **2015**, *3*, 16348–16353.

(83) Lu, X.; Zhao, C.; Electrodeposition of hierarchically structured three-dimensional nickel–iron electrodes for efficient oxygen evolution at high current densities. *Nat. Commun.* **2015**, *6*, 6616-6623.

(84) Wang, H.; Lee, H.-W.; Deng, Y.; Lu, Z.; Hsu, P.-C.; Liu, Y.; Lin, D.; Cui, Y. Bifunctional non-noble metal oxide nanoparticle electrocatalysts through lithium-induced conversion for overall water splitting. *Nat. Commun.* **2015**, *6*, 7261−7269.

(85) Chen, R.; Sun, G.; Yang, C.; Zhang, L.; Miao, J.; Tao, H.; Yang, H.; Chen, J.; Chen, P.; Liu, B. Achieving stable and efficient water oxidation by incorporating NiFe layered double hydroxide nanoparticles into aligned carbon nanotubes. *Nanoscale Horiz.* **2016**, 1, 156–160.

(86) Xu, Y.; Hao, Y.; Zhang, G.; Lu, Z.; Han, S.; Li, Y.; Sun, X. Room-temperature synthetic NiFe layered double hydroxide with different anions intercalation as an excellent oxygen evolution catalyst. *RSC Adv.* **2015**, *5*, 55131–55135.

(87) Zhang, B.; Zheng, X.; Voznyy, O.; Comin, R.; Bajdich, M.; García-Melcho, M.; Han, L; Xu, J.; Liu, M.; Zheng, L. Homogeneously dispersed multimetaloxygen-evolving catalysts. *Science* **2016**, *352*, 333−337.

(88) Gao, Y. Q.; Liu, X. Y.; Yang, G. W. Amorphous mixed-metal hydroxide nanostructures for advanced water oxidation catalysts. *Nanoscale* **2016**, 8, 5015–5023.

(89) Ni, Y.; Yao, L.; Wang, Y.; Liu, B.; Cao, M.; Hu, C. Construction of hierarchically porous graphitized carbon-supported NiFe layered double hydroxides with a core–shell structure as an enhanced electrocatalyst for the oxygen evolution reaction. *Nanoscale* **2017**, *9*, 11596–11604.

(90) Wang, C.; Moghaddam, R. B.; Brett, M. J.; Bergens, S. H. Simple Aqueous Preparation of High Activity and Stability NiFe Hydrous Oxide Catalysts for Water Oxidation. *ACS Sustainable Chem. Eng.* **2017**, *5*, 1106−1112.

(91) Nai, J.; Yin, H.; You, T.; Zheng, L. Zhang, J.; Wang, P.; Jin, Z.; Tian, Y.; Liu, J.; Tang, Z.; et al. Efficient Electrocatalytic Water Oxidation by Using Amorphous Ni–Co Double Hydroxides Nanocages. *Adv. Energy Mater.* **2015**, *5*, 1401880−1401886.

(92) He, J.; Hu, B.; Zhao, Y. Superaerophobic Electrode with Metal@Metal-Oxide Powder Catalyst for Oxygen Evolution Reaction. *Adv. Funct. Mater.* **2016**, 26, 5998–6004.

(93) Li, J.; Yan, M.; Zhou, X.; Huang, Z.-Q.; Xia, Z.; Chang, C.-R.; Ma, Y.; Qu, Y. Mechanistic Insights on Ternary $Ni_{2-x}Co_xP$ for Hydrogen Evolution and Their Hybrids with Graphene as Highly Efficient and Robust Catalysts for Overall Water Splitting. *Adv. Funct. Mater.* **2016**, *26*, 6785–6796.

(94) Wang, X.; Zheng, Y.; Yuan, J.; Shen, J.; Hu, J.; Wang, A.;Wu, L.; Niu, L. Porous NiCo diselenide nanosheets arrayed on carbon cloth as promising advanced catalysts used in water splitting. *Electrochim. Acta* **2017**, *225*, 503–513.

(95) Jiang, J.; Yan, C.; Zhao, X.; Luo, H.; Xue, Z.; Mu, T. A PEGylated deep eutectic solvent for controllable solvothermal synthesis of porous $NiCo_2S_4$ for efficient oxygen evolution reaction. *Green Chem.* **2017**, *19*, 3023–3031.

(96) Deng, J.; Zhang, H.; Zhang, Y.; Luo, P.; Liu, L.; Wang, Y. Striking hierarchical urchin-like peapoded $NiCo_2O_4$@ C as advanced bifunctional electrocatalyst for overall water splitting. *J. Power Sources* **2017**, *372*, 46–53.

(97) Liang, H.; Meng, F.; Cabán-Acevedo, M.; Li, L.; Forticaux, A.; Xiu, L.; Wang, Z.; Jin, S. Hydrothermal Continuous Flow Synthesis and Exfoliation of NiCo Layered Double Hydroxide Nanosheets for Enhanced Oxygen Evolution Catalysis. *Nano Lett.* **2015**, *15*, 1421–1427.

(98) Niu, K.-Y.; Lin, F.; Jung, S.; Fang, L.; Nordlund, D.; McCrory, C. C. L.; Weng, T-C.; Erius, P.; Doeff, M.; Zheng, H. Tuning complex transition metal hydroxide nanostructures as active catalysts for water oxidation by a laser–chemical route. *Nano Lett.* **2015**, *15*, 2498–2503.

(99) Liang, H.; Gandi, A. N.; Anjum, D. H.; Wang, X.; Schwingenschlögl, U.; Alshareef, H. N.; Plasma-Assisted Synthesis of NiCoP for Efficient Overall Water Splitting. *Nano Lett.* **2016**, *16*, 7718–7725.

(100) Bayatsarmadi, B.; Zheng, Y.; Russo, V.; Ge, L.; Casari, C. S.; Qiao, S.-Z.; Highly active nickel–cobalt/nanocarbon thin films as efficient water splitting electrodes. Nanoscale **2016**, *8*, 18507–18515.

(101) Wu, J.; Ren, Z.; Du, S.; Kong, L.; Liu, B.; Xi, W.; Zhu, J.; Fu, H. A highly active oxygen evolution electrocatalyst: Ultrathin CoNi double hydroxide/CoO nanosheets synthesized via interface-directed assembly. *Nano Res.* **2016**, *9*, 713–725.

(102) Xiao, Y.; Feng, L.; Hu, C.; Fateev, V.; Liu, C.; Xing, W. $NiCo_2O_4$ 3 dimensional nanosheet as effective and robust catalyst for oxygen evolution reaction. *RSC Adv.* **2015**, *5*, 61900–61905.

(103) Yin, Z.; Zhu, C.; Li, C.; Zhang, S.; Zhang, X.; Chen, Y. Hierarchical nickel–cobalt phosphide yolk–shell spheres as highly active and stable bifunctional electrocatalysts for overall water splitting. *Nanoscale* **2016**, *8*, 19129–19138.

(104) Li, R.; Zhou, D.; Luo, J.; Xu, W.; Li, J.; Li, S.; Cheng, P.; Yuan, D. The urchin-like sphere arrays Co_3O_4 as a bifunctional catalyst for hydrogen evolution reaction and oxygen evolution reaction. *J. Power Sources* **2017**, *341*, 250–256.

(105) Ganesan, V.; Ramasamy, P.; Kim, J. Hierarchical $Ni_{3.5}Co_{5.5}S_8$ nanosheet-assembled hollow nanocages: Superior electrocatalyst towards oxygen evolution reaction. *Int. J. Hydrogen Energ.* **2017**, *42*, 5985–5992.

(106) Yan, L.; Cao, L.; Dai, P.; Gu, X.; Liu, D.; Li, L.; Wang, Y.; Zhao, X. Metal-Organic Frameworks Derived Nanotube of Nickel–Cobalt Bimetal Phosphides as Highly Efficient Electrocatalysts for Overall Water Splitting. Adv. *Funct. Mater.* **2017**, *27*, 1703455-1703464.

(107) Yang, Y.; Lin, Z.; Gao, S.; Su, J.; Lun, Z.; Xia, G.; Chen, J.; Zhang, R.; Chen, Q. Tuning electronic structures of nonprecious ternary alloys encapsulated in graphene layers for optimizing overall water splitting activity. *ACS Catal.* **2017**, *7*, 469–479.

(108) Wang, A.-L.; Xu, H.; Li, G.-R. NiCoFe layered triple hydroxides with porous structures as high-performance electrocatalysts for overall water splitting. *ACS Energy Lett.* **2016**, *1*, 445–453.

(109) Qian, L.; Lu, Z.; Xu, T.; Wu, X.; Tian, Y.; Huo, L.; Sun, X.; Duan, X. Trinary Layered Double Hydroxides as High-Performance Bifunctional Materials for Oxygen Electrocatalysis. *Adv. Energy Mater.* **2015**, *5*, 15002451−15002460.

(110) Long, X.; Xiao, S.; Wang, Z.; Zheng, X.; Yang, S.; Co intake mediated formation of ultrathin nanosheets of transition metal LDH-an advanced electrocatalyst for oxygen evolution reaction. *Chem. Commun.* **2015**, *51*, 1120–1123.

(111) Zhang, T.; Du, J.; Xi, P.; Xu, C. Hybrids of Cobalt/Iron Phosphides Derived from Bimetal–Organic Frameworks as Highly Efficient Electrocatalysts for Oxygen Evolution Reaction. *ACS Appl. Mater. Interfaces* **2017**, *9*, 362–370.

(112) Rao, Y.; Wang, Y.; Ning, H.; Li, P.; Wu, M. Hydrotalcite-like $Ni(OH)_2$ Nanosheets in Situ Grown on Nickel Foam for Overall Water Splitting. *ACS Appl. Mater. Interfaces* **2016**, *8*, 33601–33607.

(113) Xu, Y.; Tu, W.; Zhang, B.; Yin, S.; Huang, Y.; Kraft, M.; Xu, R. Nickel Nanoparticles Encapsulated in Few-Layer Nitrogen-Doped Graphene Derived from Metal–Organic Frameworks as Efficient Bifunctional Electrocatalysts for Overall Water Splitting. *Adv. Mater.* **2017**, *29*, 1605957−1605964.

(114) Wang, S.; Nai, J.; Yang, S.; Guo, L. Synthesis of amorphous Ni− Zn double hydroxide nanocages with excellent electrocatalytic activity toward oxygen evolution reaction. *ChemNanoMat*. **2015**, *1*, 324–330.

(115) Stern, L.-A.; Feng, L.; Song, F.; Hu, X. Ni_2P as a Janus catalyst for water splitting: the oxygen evolution activity of Ni_2P nanoparticles. *Energy Environ. Sci.* **2015**, *8*, 2347–2351.

(116) Yu, X.-Y.; Feng, Y.; Guan, B.; Lou, X. W.; Paik, U. Carbon coated porous nickel phosphides nanoplates for highly efficient oxygen evolution reaction. *Energy Environ. Sci.* **2016**, *9*, 1246–1250.

(117) Liang, J.; Wang, Y.-Z.; Wang, C.-C.; Lu, S.-Y. In situ formation of NiO on Ni foam prepared with a novel leaven dough method as an outstanding electrocatalyst for oxygen evolution reactions. *J. Mater. Chem. A* **2016**, *4*, 9797–9806.

(118) Song, J.; Zhu, C.; Xu, B. Z.; Fu, S.; Engelhard, M. H.; Ye, R.; Du, D.; Beckman , S. P.; Lin, Y. Bimetallic Cobalt-Based Phosphide Zeolitic Imidazolate Framework: CoP_x Phase-Dependent Electrical Conductivity and Hydrogen Atom Adsorption Energy for Efficient Overall Water Splitting. *Adv. Energy Mater.* **2017**, *7*, 1601555-1601563.

(119) Liu, Y.; Li, Q.; Si, R.; Li, G.-D.; Li, W.; Liu, D.-P.; Wang, D.; Sun, L.; Zhang, Y.; Zou, X. Coupling Sub-Nanometric Copper Clusters with Quasi-Amorphous Cobalt Sulfide Yields Efficient and Robust Electrocatalysts for Water Splitting Reaction. *Adv. Mater.* **2017**, *29*, 1606200−1606207.

(120) Jin, Z.; Li, P.; Xiao, D. Metallic Co_2P ultrathin nanowires distinguished from CoP as robust electrocatalysts for overall water-splitting. *Green Chem.* **2016**, *18*, 1459–1464.

(121) Chang, J.; Liang, L.; Li, C.; Wang, M.; Ge, J.; Liu, C.; Xing, W. Ultrathin cobalt phosphide nanosheets as efficient bifunctional catalysts for a water electrolysis cell and the origin for cell performance degradation. *Green Chem.* **2016**, *18*, 2287–2295.

(122) Bai, Y.; Zhang, H.; Feng, Y.; Fang, L.; Wang, Y. Sandwich-like CoP/C nanocomposites as efficient and stable oxygen evolution catalysts. *J. Mater. Chem. A* **2016**, *4*, 9072–9079.

(123) Zhu, Y.-P.; Liu, Y.-P.; Ren, T.-Z.; Yuan, Z.-Y. Self-supported cobalt phosphide mesoporous nanorod arrays: a flexible and bifunctional electrode for highly active electrocatalytic water reduction and oxidation. *Adv. Funct. Mater.* **2015**, *25*, 7337–7347.

(124) Li, X.; Fang, Y.; Li, F.; Tian, M.; Long, X.; Jin, J.; Ma, J. Ultrafine Co_2P nanoparticles encapsulated in nitrogen and phosphorus dual-doped porous carbon nanosheet/carbon nanotube hybrids: high-performance bifunctional electrocatalysts for overall water splitting. *J. Mater. Chem. A* **2016**, *4*, 15501–15510.

(125) Wang, J.; Yang, W.; Liu, J. CoP_2 nanoparticles on reduced graphene oxide sheets as a super-efficient bifunctional electrocatalyst for full water splitting. *J. Mater. Chem. A* **2016**, *4*, 4686–4690.

(126) Yang, L.; Qi, H.; Zhang, C.; Sun, X. An efficient bifunctional electrocatalyst for water splitting based on cobalt phosphide. *Nanotechnology* **2016**, *27*, 23LT01−23LT07.

(127) Oh, S.; Kim, H.; Kwon, Y.; Kim, M.; Cho, E.; Kwon, H. Porous Co–P foam as an efficient bifunctional electrocatalyst for hydrogen and oxygen evolution reactions. *J. Mater. Chem. A* **2016**, *4*, 18272–18277.

(128) Das, D.; Nanda, K. K. One-step integrated fabrication of Co_2P nanoparticles encapsulated N, P dual-doped CNTs for highly advanced total water splitting. *Nano Energy* **2016**, *30*, 303–311.

(129) Jia, G.; Hu, Y.; Qian, Q.; Yao, Y.; Zhang, S.; Li, Z.; Zou, Z. Formation of Hierarchical Structure Composed of (Co/Ni)Mn-LDH Nanosheets on MWCNT Backbones for Efficient Electrocatalytic Water Oxidation. *ACS Appl. Mater. Interfaces* **2016**, *8*, 14527–14534.

(130) Guan, B. Y.; Yu, L.; Lou, X. W. General Synthesis of Multishell Mixed-Metal Oxyphosphide Particles with Enhanced Electrocatalytic Activity in the Oxygen Evolution Reaction. *Angew. Chem. Int. Ed.* **2017**, *56*, 2386–2389.

(131) Hu, C.; Zhang, L.; Zhao, Z.-J.; Luo, J.; Shi, J.; Huang, Z.; Gong, J. Edge Sites with Unsaturated Coordination on Core–Shell Mn_3O_4@MnxCo$_3$−xO$_4$ Nanostructures for Electrocatalytic Water Oxidation. *Adv. Mater.* **2017**, *29*, 1701820−1701826.

(132) Sun, Y.; Wang, C.; Ding, T.; Zuo, J.; Yang, Q. Fabrication of amorphous CoMoS 4 as a bifunctional electrocatalyst for water splitting under strong alkaline conditions. *Nanoscale* **2016**, *8*, 18887–18892.

(133) Yoon, T.; Kim, K. S. One-Step Synthesis of CoS-Doped β-Co(OH)$_2$@Amorphous MoS_{2+x} Hybrid Catalyst Grown on Nickel Foam for High-Performance Electrochemical Overall Water. *Adv. Funct. Mater.* **2016**, *26*, 7386–7393.

(134) Zhang, J.; Zhang, D.; Yang, Y.; Ma, J.; Cui, S.; Lia, Y.; Yuan, B. Facile synthesis of $ZnCo_2O_4$ mesoporous structures with enhanced electrocatalytic oxygen evolution reaction properties. *RSC Adv.* **2016**, *6*, 92699–92704.

(135) Liu, X.; Chang, Z.; Luo, L.; Xu, T. Lei, X.; Liu, J.; Sun, X. Hierarchical $Zn_xCo_{3-x}O_4$ Nanoarrays with High Activity for Electrocatalytic Oxygen Evolution. *Chem. Mater.* **2014**, *26*, 1889–1895.

(136) Xiong, S.; Li, P.; Jin, Z.; Gao, T.; Wang, Y.; Guo, Y.; Xiao, D. Enhanced catalytic performance of ZnO-CoO_x electrode generated from electrochemical corrosion of Co-Zn alloy for oxygen evolution reaction. *Electrochim. Acta* **20**16, *222*, 999–1006.

(137) Gao, M.-R.; Cao, X.; Gao, Q.; Xu, Y.-F.; Zhang, Y.-R.; Jiang, J.; Yu, S.-H. Nitrogen-Doped Graphene Supported $CoSe_2$ Nanobelt Composite Catalyst for Efficient Water Oxidation. *ACS Nano* **2014**, 8, 3970–3978.

(138) Sun, C.; Dong, Q.; Yang, J.; Dai, Z.; Lin, J.; Chen, P.; Huang, W.; Dong, X. Metal–organic framework derived $CoSe_2$ nanoparticles anchored on carbon fibers as bifunctional electrocatalysts for efficient overall water splitting. *Nano Res.* **2016**, *9*, 2234–2243.

(139) Liu, T.; Liu, Q.; Asiri, A. M.; Luo, Y.; Sun, X. An amorphous CoSe film behaves as an active and stable full water-splitting electrocatalyst under strongly alkaline conditions. *Chem. Commun*. **2015**, *51*, 16683–16686.

(140) Fang, L.; Li, W.; Guan, Y.; Feng, Y. Zhang, H.; Wang, S.; Wang, Y. Tuning Unique Peapod-Like $Co(S_xSe_{1-x})_2$ Nanoparticles for Efficient Overall Water Splitting. *Adv. Funct. Mater.* **2017**, *27*, 1701008−1701016.

(141) Guo, Y.; Yao, Z.; Shang, C.; Wang, E. Amorphous Co_2B Grown on $CoSe_2$ Nanosheets as a Hybrid Catalyst for Efficient Overall Water Splitting in Alkaline Medium. *ACS Appl. Mater. Interfaces* **2017**, *9*, 39312–39317.

(142) Qiao, X.; Jin, J.; Fan, H.; Li, Y.; Liao, S. In situ growth of cobalt sulfide hollow nanospheres embedded in nitrogen and sulfur co-doped graphene nanoholes as a highly active electrocatalyst for oxygen reduction and evolution. *J. Mater. Chem. A* **2017**, *5*, 12354–12360.

(143) Dou, S.; Tao, L.; Huo, J.; Wang, S.; Dai, L. Etched and doped Co_9S_8/graphene hybrid for oxygen electrocatalysis. *Energy Environ. Sci.* **2016**, *9*, 1320–1326.

(144) Al-Mamun, M.; Wang, Y.; Liu, P.; Zhong, Y. L.; Yin, H.; Su, X.; Zhang, H.; Yang, H.; Wang, D.; Tan, Z.; Zhao, H. One-step solid phase synthesis of a highly efficient and robust cobalt pentlandite electrocatalyst for the oxygen evolution reaction. *J. Mater. Chem. A* **2016**, *4*, 18314–18321.

(145) Liu, P. F.; Yang, S.; Zhang, B.; Yang, H. G. Defect-Rich Ultrathin Cobalt–Iron Layered Double Hydroxide for Electrochemical Overall Water Splitting. *ACS Appl. Mater. Interfaces* **2016**, *8*, 34474–34481.

(146) Duan, J.; Chen, S.; Vasileff, A.; Qiao, S. Z. Anion and Cation Modulation in Metal Compounds for Bifunctional Overall Water Splitting. *ACS Nano* **2016**, *10*, 8738–8745.

(147) Nsanzimana, J. M. V.; Peng, Y.; Xu, Y. Y.; Thia, L. Wang, C.; Xia, B. Y.; Wang, X. An efficient and earth-abundant oxygen-evolving electrocatalyst based on amorphous metal borides. *Adv. Energy Mater.* **2018**, *8*, 1701475−1701481.

(148) Yang, J.; Zhu, G.; Liu, Y.; Xia, J.; Ji, Z.; Shen, X.; Wu, S. Fe_3O_4-Decorated Co_9S_8 Nanoparticles In Situ Grown on Reduced Graphene Oxide: A New and Efficient Electrocatalyst for Oxygen Evolution Reaction. *Adv. Funct. Mater.* **2016**, *26*, 4712–4721.

(149) Zhang, X.; Zhang, X.; Xu, H.; Wu, Z.; Wang, H.; Liang, Y. Iron-Doped Cobalt Monophosphide Nanosheet/Carbon Nanotube Hybrids as Active and Stable Electrocatalysts for Water Splitting. *Adv. Funct. Mater.* **2017**, *27*, 1606635−1606646.

(150) Lu, X.-F.; Gu, L.-F.; Wang, J.-W.; Wu, J.-X.; Liao, P.-Q.; Li, G.-R. Bimetal-Organic Framework Derived $CoFe_2O_4$/C Porous Hybrid Nanorod Arrays as High-Performance Electrocatalysts for Oxygen Evolution Reaction. *Adv. Mater.* **2017**, *29*, 1604437−1604443.

(151) Han, X.; Yu, C.; Yang, J.; Zhao, C.; Huang, H.; Liu, Z,; Ajauan, P. M.; Qiu, J. Mass and Charge Transfer Coenhanced Oxygen Evolution Behaviors in CoFe-Layered Double Hydroxide Assembled on Graphene. *Adv. Mater. Interfaces* **2016**, *3*, 1500782−1500789.

(152) Grewe, T.; Deng, X.; Tüysüz, H. Influence of Fe Doping on Structure and Water Oxidation Activity of Nanocast Co_3O_4. *Chem. Mater.* **2014**, *26*, 3162–3168.

(153) Yang, F.; Sliozberg, K.; Sinev, I.; Antoni, H.; Bähr, A.; Ollegott, K.; Xia, W.; Masa, J.; Grünert, W.; Roldan, R. C. Synergistic effect of cobalt and iron in layered double hydroxide catalysts for the oxygen evolution reaction. *ChemSusChem* **2017**, *10*, 156–165.

(154) Gao, T.; Jin, Z.; Liao, M.; Xiao, J.; Yuan, H.; Xiao, D. A trimetallic V–Co–Fe oxide nanoparticle as an efficient and stable electrocatalyst for oxygen evolution reaction. *J. Mater. Chem. A.* **2015**, *3*, 17763–17770.

(155) Liu, W.; Du, K.; Liu, L.; Zhang, J.; Zhu, J.; Shao, Y.; Li, M. et al.; One-step electroreductively deposited iron-cobalt composite films as efficient bifunctional electrocatalysts for overall water splitting. *Nano Energy* **2017**, *38*, 576–584.

(156) Liu, Y.; Li, J.; Li, F.; Li, W.; Yang, H.; Zhang, X.; Liu, Y.; Ma, J. A facile preparation of $CoFe_2O_4$ nanoparticles on polyaniline-functionalised carbon nanotubes as enhanced catalysts for the oxygen evolution reaction. *J. Mater. Chem. A* **2016**, *4*, 4472–4478.

(157) Lin, X.; Li, X.; Li, F.; Fang, Y. Yian, M. An X.; Fu, Y.; Jin, J.; Ma, J. Precious-metal-free Co–Fe–O_x coupled nitrogen-enriched porous carbon nanosheets derived from Schiff-base porous polymers as superior electrocatalysts for the oxygen evolution reaction. *J. Mater. Chem. A* **2016**, *4*, 6505–6512.

(158) Li, M.; Liu, T.; Bo, X.; Zhou, M.; Guo, L. A novel flower-like architecture of FeCo@NC-functionalized ultra-thin carbon nanosheets as a highly efficient 3D bifunctional electrocatalyst for full water splitting. *J. Mater. Chem. A* **2017**, *5*, 5413–5425.

(159) Sun, F.; Li, L.; Wang, G.; Lin, Y. Iron incorporation affecting the structure and boosting catalytic activity of β-$Co(OH)_2$: exploring the reaction mechanism of ultrathin two-dimensional carbon-free Fe_3O_4-decorated β-$Co(OH)_2$ nanosheets as efficient oxygen evolution electrocatalysts. *J. Mater. Chem. A* **2017**, *5*, 6849–6859.

(160) Du, J.; Zhang, T.; Xing, J.; Xu, C.; Hierarchical porous Fe_3O_4/Co_3S_4 nanosheets as an efficient electrocatalyst for the oxygen evolution reaction. *J. Mater. Chem. A* **2017**, *5*, 9210–9216.

(161) Zhang, X.; An, L.; Yin, J.; Xi, P.; Zheng, Z.; Du, Y. Effective Construction of High-quality Iron Oxy-hydroxides and Co-doped Iron Oxy-hydroxides Nanostructures: Towards the Promising Oxygen Evolution Reaction Application. *Sci. Rep.* **2017**, *7*, 43590−43600.

(162) Zhao, Y.; Zhang, J.; Guo, X.; Fan, H.; Wu, W.; Liu, H.; Wang, G. Fe_3C@nitrogen doped CNT arrays aligned on nitrogen functionalized carbon nanofibers as highly efficient catalysts for the oxygen evolution reaction. *J. Mater. Chem. A* **2017**, 5, 19672–19679.

(163) Fan, K.; Ji, Y.; Zou, H.; Zhang, J.; Zhu, B,; Chen, H.; Daniel, Q.; Luo, Y.; Yu, J.; Sun, L. Hollow Iron-Vanadium Composite Spheres: A Highly Efficient Iron-Based Water Oxidation Electrocatalyst without the Need for Nickel or Cobalt. *Angew. Chem. Int. Ed.* **2017**, *56*, 3289–3293.

(164) Xie, M.; Xiong, X.; Yang, L.; Shi, X.; Asiri, A. M.; Sun, X. An $Fe(TCNQ)_2$ nanowire array on Fe foil: an efficient non-noble-metal catalyst for the oxygen evolution reaction in alkaline media. *Chem. Commun.* **2018**, *54*, 2300–2303.

(165) Tang, C.; Asiri, A. M.; Luo, Y.; Sun, X.; Electrodeposited Ni-P Alloy Nanoparticle Films for Efficiently Catalyzing Hydrogen- and Oxygen-Evolution Reactions. *ChemNanoMat*. **2015**, *1*, 558–561.

(166) Yu, J.; Li, Q.; Chen, N.; Xu, C.-Y. Zhen, L.; Wu, J.; Dravid, V. P. Carbon-Coated Nickel Phosphide Nanosheets as Efficient Dual-Electrocatalyst for Overall Water Splitting. *ACS Appl. Mater. Interfaces* **2016**, *8*, 27850–27858.

(167) Li, J.; Li, J.; Zhou, X.; Xia, Z.; Gao, W.; Ma, Y.; Qu, Y. Highly Efficient and Robust Nickel Phosphides as Bifunctional Electrocatalysts for Overall Water-Splitting. *ACS Appl. Mater. Interfaces* **2016**, *8*, 10826–10834.

(168) Menezes, P. W.; Indra, A.; Das, C.; Walter, C.; Göbel, C.; Gutkin, V.; Schmeiβer, D.; Driess, M. Uncovering the nature of active species of nickel phosphide catalysts in high-performance electrochemical overall water splitting. *ACS Catal.* **2017**, *7*, 103–109.

(169) Wang, X.; Li, W.; Xiong, D.; Petrovykh, D. Y.; Liu, L. Bifunctional nickel phosphide nanocatalysts supported on carbon fiber paper for highly efficient and stable overall water splitting. *Funct. Mater.* **2016**, *26*, 4067–4077.

(170) Zhang, J.; Wang, T.; Pohl, D.; Rellinghaus, B.; Dong, R.; Liu, S.; Zhuang, X.; Feng, X. Interface Engineering of MoS_2/Ni_3S_2 Heterostructures for Highly Enhanced Electrochemical Overall-Water-Splitting Activity. *Angew. Chem. Int. Ed.* **2016**, *55*, 6702–6707.

(171) Zheng, J.; Zhou, W.; Liu, T.; Liu, S.; Wang, C.; Guo, L. Homologous NiO//Ni_2 P nanoarrays grown on nickel foams: a well matched electrode pair with high stability in overall water splitting. *Nanoscale* **2017**, *9*, 4409–4418.

(172) Wei, L.; Goh, K.; Birer, Ö.; Karahan, H. E. Chang, J.; Zhai, S.; Chen, X.; Chen, Y.A hierarchically porous nickel–copper phosphide nano-foam for efficient electrochemical splitting of water. *Nanoscale* **2017**, *9*, 4401–4408.

(173) Kibsgaard, J.; Chorkendorff, I. Considerations for the Scaling-up of Water Splitting Catalysts. *Nature Energy* **2019**, *4*, 430–433.

(174) Mayrhofer, K. J. J.; Hartl, K.; Juhart, V.; Arenz, M. Degradation of Carbon-Supported Pt Bimetallic Nanoparticles by Surface Segregation. *JACS* **2009**, *131*, 16348–16349.

(175) Anantharaj, S.; Noda, S.; Driess, M.; Menezes, P. W. The Pitfalls of Using Potentiodynamic Polarization Curves for Tafel Analysis in Electrocatalytic Water Splitting. *ACS Energy Lett.* **2021**, *6*, 1607−1611.

(176) Trotochaud, L.; Young, S.L.; Ranney, J.K.; Boettcher, S.W. Journal of the American Chemical Society Nickel–Iron Oxyhydroxide Oxygen-Evolution Electrocatalysts: The Role of Intentional and Incidental Iron Incorporation. *JACS* **2014**, *136*, 6744−6753.

(177) Spanos, I.; Tesch, M.F.; Yu, M.; Tüysüz, H.; Zhang, J.; Feng, X.; Müllen, K.; Schlögl, R.; Mechler, A.K. Facile Protocol for Alkaline Electrolyte Purification and Its Influence on a Ni–Co Oxide Catalyst for the Oxygen Evolution Reaction. *ACS Catal.* **2019**, *9*, 8165−8170.

(178) Lyons, M.E.G.; Floquet, S. Mechanism of oxygen reactions at porous oxide electrodes. Part 2-Oxygen evolution at RuO_2, IrO_2 and $Ir_xRu_{1-x}O_2$ electrodes in aqueous acid and alkaline solution. *Phys. Chem. Chem. Phys.* **2011**, *13*, 5314–5335.

(179) Kasian, O.; Geiger, S.; Mayrhofer, K. J. J.; Cherevko, S. Electrochemical On-Line ICP-MS in Electrocatalysis Research. *Chem. Rec*. **2019**, *1*, 2130–2142.

(180) Anderson, G.C.; Pivovar, B.S.; Alia, S.M. Establishing Performance Baselines for the Oxygen Evolution Reaction in Alkaline Electrolytes Establishing Performance Baselines for the Oxygen Evolution Reaction in Alkaline Electrolytes. *J. Electrochem. Soc*. **2020**, *167*, 044503−044515.

(181) Bock, C.; MacDougall, B. Novel Method for the Estimation of the Electroactive Pt Area. *J. Electrochem. Soc*. **2003**, 150, E377−E383.

(182) Anantharaj, S.; Sugime, H.; Suguru, N. Why shouldn't double-layer capacitance (C_{dl}) be always trusted to justify Faradaic electrocatalytic activity differences? *J. Electroanal. Chem.* **2021**, *903*, 115842−115852.

(183) Choe, Y. K.; Fujimoto, C.; Lee, K. S.; Dalton, L. T.; Ayers, K.; Henson, N. J.; Kim, Y. S. Alkaline Stability of Benzyl Trimethyl Ammonium Functionalized Polyaromatics: A Computational and Experimental Study. *Chem. Mater.* **2014**, *26*, 5675–5682.

(184) Li, D.; Matanovic, I.; Lee, A. S.; Park, E. J.; Fujimoto, C.; Chung, H. T.; Kim, Y. S. Phenyl Oxidation Impacts the Durability of Alkaline Membrane Water Electrolyzer. *ACS Appl. Mater. Interfaces* **2019**, *11*, 9696 –9701.

(185) Li, D.; Park, E. J.; Zhu, W.; Shi, Q.; Zhou, Y.; Tian, H.; Lin, Y.; Serov, A.; Zulevi, B.; Baca, E. D. et al. Highly quaternized polystyrene ionomers for high performance anion exchange membrane water electrolysers. *Nat. Energy*. **2020**, *5*, 378–385.

(186) Liu, J.; Kang, Z.; Li, D.; Pak, M.; Alia, S. M.; Fujimoto, C.; Bender, G.; Kim, Y. S.; Weber, A. Z. Elucidating the Role of Hydroxide Electrolyte on Anion-Exchange Membrane Water Electrolyzer Performan. *J. Electrochem. Soc.* **2021**, *168*, 054522−054531.

(187) Li, D.; Motz, A. R.; Bae, C.; Fujimoto, C.; Yang, G.; Zhang, F.; Ayers, K. E.; and Kim, Y. S. Durability of anion exchange membrane water electrolyzer. *Energy Environ. Sci.* **2021**, *14*, 3393–3419.

(188) Hu, X.; Huang, Y.; Liu, L.; Ju, Q.; Zhou, X. Qiao, X.; Zheng, Z.; Li, N. Piperidinium functionalized aryl ether-free polyaromatics as anion exchange membrane for water electrolysers: Performance and durability. *J. Membr. Sci.* **2021**, *621*, 118964–118975.

(189) Park, E. J.; Capuano, C. B.; Ayers, K. E.; Bae, C. Chemically durable polymer electrolytes for solid-state alkaline water electrolysis. *J. Power Sources* **2018**, *375*, 367–372.

(190) Xiao, J.; Oliveira, A. M.; Wang, L.; Zhao, Y.; Wang, T.; Wang, J.; Setzler, B. P.; Yan Y. Water-Fed Hydroxide Exchange Membrane Electrolyzer Enabled by a Fluoride-Incorporated Nickel–Iron Oxyhydroxide Oxygen Evolution Electrode. *ACS Catal. **2021**,* *11*, 264–270.

(191) Chen, N. J.; Paek, S. Y.; Lee, J. Y.; Park, J.H. Lee, S. Y.; Lee, Y. M. High-performance anion exchange membrane water electrolyzers with a current density of 7.68 A cm^{-2} and a durability of 1000 hours. *Energy Environ. Sci.* **2021**, *14*, 6338–6348.

(192) Parrondo, J.; Ramani, V. Stability of Poly(2,6-dimethyl 1,4-phenylene)Oxide-Based Anion Exchange Membrane Separator and Solubilized Electrode Binder in Solid-State Alkaline Water Electrolyzers. *J. Electrochem. Soc.* **2014**, *161*, F1015–F1020.

(193) Marinkas, A.; Struźyńska-Piron, I.; Lee, Y. Lim, A.; Park, H. S.; Jang, j. H.; Kim, H. J.; Kim, J.; Maljusch, A.; Conradi, O. et al. Anion-conductive membranes based on 2-mesityl-benzimidazolium functionalised poly(2,6-dimethyl-1,4-phenylene oxide) and their use in alkaline water electrolysis. *Polymer* **2018**, *145*, 242 –251.

(194) Schauer, J.; Hnát, J.; Brožová, L.; Žitka, J.; Bouzek, K. Anionic Catalyst Binders Based on Trimethylamine-Quaternized Poly(2,6-Dimethyl-1,4-Phenylene Oxide) for Alkaline Electrolyzers. J. Membr. Sci. **2015**, *473*, 267–273.

(195) Chu, X.; Shi, Y.; Liu, L.; Huang, Y.; Li, N. Piperidinium-functionalized anion exchange membranes and their application in alkaline fuel cells and water electrolysis. *J. Mater. Chem. A* **2019**, *7*, 7717–7727.

(196) Lee, N.; Duong, D. T.; Kim, D. Cyclic ammonium grafted poly (arylene ether ketone) hydroxide ion exchange membranes for alkaline water electrolysis with high chemical stability and cell efficiency. *Electrochim. Acta.* **2018**, *271*, 150–157.

(197) Xiao, L.; Zhang, S.; Pan, J.; Yang, C.; He, M.; Zhuang, L.; Lu, J. First implementation of alkaline polymer electrolyte water electrolysis working only with pure water. *Energy Environ. Sci.* **2012**, *5*, 7869–7871.

(198) Schauer, J.; Žitka, J.; Pientka, Z.; Křivčík, J.; Hnát, J.; Bouzek, K. Polysulfone–based anion exchange polymers for catalyst binders in alkaline electrolyzers. *J. Appl. Polym. Sci.* **2015**, *132*, 42581– 42587.

(199) Parrondo, J.; Arges, C.G; Niedzwiecki, M.; Anderson, E. B.; Ayersb, K. E.; Ramani, V. Degradation of anion exchange membranes used for hydrogen production by ultrapure water electrolysis. *RSC Adv.* **2014**, *4*, 9875–9879.

(200) Penchev, H.; Borisov, G.; Petkucheva, E.; Ublekov, F.; Sinigersky, V.; Radev, I.; Slavcheva, E. Highly KOH doped para–polybenzimidazole anion exchange membrane and its performance in Pt/$TinO_{2n-1}$ catalyzed water electrolysis cell. *Mater. Lett*. **2018**, *221*, 128–130.

(201) Jensen, J. O.; Aili, D.; Hansen, M. K.; Li, Q.; Bjerrum, N. J.; Christensen, E. A Stability Study of Alkali Doped PBI Membranes for Alkaline Electrolyzer Cells. *ECS Trans*. **2014**, *64*, 1175–1184.

(202) Aili, D.; Hansen, M. K.; Renzaho, R. F.; Li, Q.; Christensen, E.; Jensen, J. O.; Bjerrum, N. J. Heterogeneous anion conducting membranes based on linear and crosslinked KOH doped polybenzimidazole for alkaline water electrolysis. *J. Membr. Sci.* **2013**, *447*, 424–432.

(203) Diaz, L. A.; Hnát, J.; Heredia, N.; Bruno, M. M.; Viva, F. A.; Paidar, M.; Corti, H. R.; Bouzek, K.; Abuin, G.C. Alkali doped poly (2,5-benzimidazole) membrane for alkaline water electrolysis: Characterization and performance. *J. Power Sources* **2016**, *312*, 128 –136.

(204) Vincent, I.; Lee, E. C.; Kim, H. M. Highly Cost-Effective Platinum-Free Anion Exchange Membrane Electrolysis for Large Scale Energy Storage and Hydrogen Production. *RSC Adv*. **2020**, 10, 37429–37438.

(205) Kraglund, M. R.; Carmo, M.; Schiller, G.; Ansar, S. A.; Aili, D.; Christensen, E.; Jensen, J. O. Ion-solvating membranes as a new approach towards high rate alkaline electrolyzers. *Energy Environ. Sci.* **2019**, *12*, 3313–3320.

(206) Wang, L.; Weissbach, T.; Reissner, R.; Ansar, A.; Gago, A. S.; Holdcroft, S.; Friedrich, K. A. High Performance Anion Exchange Membrane Electrolysis Using Plasma-Sprayed, Non-Precious-Metal Electrodes. *ACS Appl. Energy Mater.* **2019**, *2*, 7903–7912.

(207) Wright, A. G.; Fan, J.; Britton, B.; Weissbach, T.; Lee, H.-F.; Kitching, E. A.; Peckhama, T. J.; Holdcroft, S. Hexamethyl-p-Terphenyl Poly(benzimidazolium): A Universal Hydroxide-Conducting Polymer for Energy Conversion Devices. *Energy Environ. Sci.* **2016**, *9*, 2130–2142.

(208) Kraglund, M. R.; Aili, D.; Jankova, K.; Christensen, E.; Li, Q.; Jensen, J. O. Zero-Gap Alkaline Water Electrolysis Using Ion-Solvating Polymer Electrolyte Membranes at Reduced KOH Concentrations. *J. Electrochem. Soc.* **2016**, *163*, F3125–F3131.

(209) Aili, D.; Wright, A. G.; Kraglund, M. R.; Jankova, K.; Holdcroft, S.; Jensena, J. O. Towards a stable ion-solvating polymer electrolyte for advanced alkaline water electrolysis. *J. Mater. Chem. A* **2017**, *5*, 5055–5066.

(210) Faraj, M.; Boccia, M.; Miller, H.; Martini, F.; Borsacchi, S.; Geppi, M.; Pucci, A. New LDPE based anion-exchange membranes for alkaline solid polymeric electrolyte water electrolysis. *Int. J. Hydrog. Energy* **2012**, *37*, 14992–15002.

(211) Wu, X.; Scott, K. A non-precious metal bifunctional oxygen electrode for alkaline anion exchange membrane cells. *J. Power Sources* **2012**, *206*, 14–19.

(212) Gupta, G.; Scott, K.; Mamlouk, M. Performance of polyethylene based radiation grafted anion exchange membrane with polystyrene-b-poly (ethylene/butylene)-b-polystyrene based ionomer using $NiCo_2O_4$ catalyst for water electrolysis. *J. Power Sources* **2018**, *375*, 387–396.

(213) Žitka, J.; Peter, J.; Galajdová, B.; Pavlovec, L.; Pientka, Z.; Paidar, M.; Hnát, J.; Bouzek, K. Anion exchange membranes and binders based on polystyrene-block-poly (ethylene-ran-butylene)-block-polystyrene copolymer for alkaline water electrolysis. *Desalin. Water Treat.* **2019**, *142*, 90–97.

(214) Su, X.; Gao, L.; Hu, L.; Qaisrani, N. A.; Yan, X.; Zhang, W.; Jiang, X.; Ruan, X.; He, G. Novel piperidinium functionalized anionic membrane for alkaline polymer electrolysis with excellent electrochemical properties. *J. Memb. Sci.* **2019**, 581, 283–292.

(215) Hnát, J.; Plevová, M.; Žitka, J.; Paidar, M.; Bouzek, K. Anion-selective materials with 1,4-diazabicyclo[2.2.2]octane functional groups for advanced alkaline water electrolysis. *Electrochim. Acta* **2017**, *248*, 547–555.

(216) Vengatesan, S.; Santhi, S.; Jeevanantham, S.; Sozhan, G. Quaternized poly (styrene-co-vinylbenzyl chloride) anion exchange membranes for alkaline water electrolysers. *J. Power Sources* **2015**, *284*, 361–368.

(217) Cao, Y. C.; Wu, X.; Scott, K. A quaternary ammonium grafted poly vinyl benzyl chloride membrane for alkaline anion exchange membrane water electrolysers with no-noble-metal catalysts. *Int. J. Hydrog. Energ.* **2012**, *37*, 9524–9528.

(218) Wu, X.; Scott, K. A polymethacrylate-based quaternary ammonium OH^- ionomer binder for non-precious metal alkaline anion exchange membrane water electrolysers. *J. Power Sources* **2012**, *214*, 124–129.

(219) Wu, X.; Scott, K. A Li-doped Co_3O_4 oxygen evolution catalyst for non-precious metal alkaline anion exchange membrane water electrolysers. *Int. J. Hydrog. Energ*. **2013**, *38*, 3123–3129.

(220) Cha, M. S.; Park, J. E.; Kim, S.; Han, S.-H.; Shin, S.-H.; Yang, S. H.; Kim, T.-H.; Yu, D. M.; So, S.; Hong, Y. T. et al. Poly(carbazole)-based anion-conducting materials with high performance and durability for energy conversion devices. *Energy Environ. Sci.* **2020**, *13*, 3633–3645.

(221) Konovalova, A. Kim, H.; Kim, S.; Lim, A.; Park, H. S.; Kraglund, M. R.; Aili, D.; Jiang, J. H.; Kim, H.-J.; Henkensmeier, D. Blend membranes of polybenzimidazole and an anion exchange ionomer (FAA3) for alkaline water electrolysis: Improved alkaline stability and conductivity. *J. Membr. Sci.* **2018**, *564*, 653–662.

(222) Diaz, L. A.; Coppola, R. E.; Abuin, G. C.; R.Escudero-Cid, R.; Herranz, D.; Ocón, P. Alkali-doped polyvinyl alcohol – Polybenzimidazole membranes for alkaline water electrolysis. *J. Membr. Sci.* **2017**, *535*, 45–55.

(223) Hnát, J.; Paidar, M.; Schauer, J.; Bouzek, K. Polymer anion-selective membrane for electrolytic water splitting: The impact of a liquid electrolyte composition on the process parameters and long-term stability. *Int. J. Hydrog. Energ.* **2014**, *39*, 4779–4787.

(224) Wu, X.; Scott, K.; Xie, F.; Alford, N. A reversible water electrolyser with porous PTFE based OH^- conductive membrane as energy storage cells. *J. Power Sources* **2014**, *246*, 225-231.

(225) Park, H. J.; Lee, S. Y.; Lee, T. K.; Kim, H.-J.; Lee, Y. M. N_3-butyl imidazolium-based anion exchange membranes blended with Poly(vinyl alcohol) for alkaline water electrolysis. *J. Membr. Sci.* **2020**, *611*, 118355–118365.

(226) Aili, D.; Kraglund, M. R.;Tavacoli, J.; Chatzichristodoulou, C.; Jensen, J. O. Polysulfone-polyvinylpyrrolidone blend membranes as electrolytes in alkaline water electrolysis. *J. Membr. Sci.* **2020**, *598*, 117674–117683.

(227) Aili, D.; Hansen, M. K.; Andreasen, J. W.; Zhang, J.; Jensen, J. O.; Bjerrum, N. J.; Li, Q. Porous poly(perfluorosulfonic acid) membranes for alkaline waterelectrolysis *J. Memb. Sci.* **2015**, *493*, 589–598.

(228) Chanda, D.; Hnát, J.; Bystron, T.; Paidar, M.; Bouzek, K. Optimization of synthesis of the nickel-cobalt oxide based anode electrocatalyst and of the related membrane-electrode assembly for alkaline water electrolysis. *J. Power Sources* **2017**, 347, 247–258.

(229) Leng, Y.; Chen, G.; Mendoza, A. J.; Tighe, T. B.; Hickner, M. A.; Wang, C. Y. Solid-State Water Electrolysis with an Alkaline Membrane. *JACS* **2012**, *134*, 9054–9057.

(230) Pavel, C. C.; Cecconi, F.; Emiliani, C.; Santiccioli, S.; Scaffidi, A.; Catanorchi, S.; Comotti, M. Highly efficient platinum group metal free based membrane-electrode assembly for anion exchange membrane water electrolysis. *Angew. Chem. Int. Ed.* **2014**, *53*,1378–1381.

(231) Ahn, S. H.; Lee, B.-S.; Choi, I.; Yoo, S. J.; Kim, H.-J.; Cho, E. A.; Henkensmeier, D.; Nam, S. W.; Kim, S.-K.; Jang, J. H. Development of a membrane electrode assembly

for alkaline waterelectrolysis by direct electrodeposition of nickel on carbon papers. *Appl. Catal. B* **2014**, *197*, 154–155.

(232) Cho, M. K.; Park, H.-Y.; Choe, S.; Yoo, S. J.; Kim, J. Y.; Kim, H.-J.; Henkensmeier, D.; Lee, S. Y.; Sung, Y.-E.; Park, H. S.; Jang, J. H. Factors in electrode fabrication for performance enhancement of anion exchange membrane water electrolysis. J. *Power Sources* **2017**, *347*, 283–290.

(233) Cho, M. K.; Park, H.-Y.; Lee, H. J.; Kim, H.-J.; Lim, A.; Henkensmeier, D.; Yoo, S. J.; Kim J. Y.; Lee, S. Y.; Park, H. S.; Jang, J. H. Alkaline anion exchange membrane water electrolysis: Effects of electrolyte feed method and electrode binder content. J. *Power Sources* **2018**, *382*, 22–29.

(234) Vincent, I.; Kruger, A.; Bessarabov. D. Development of Efficient Membrane Electrode Assembly for Low Cost Hydrogen Production by Anion Exchange Membrane Electrolysis. *Int. J. Hydrog. Energ.* **2017**, *42*, 10752–10761.

(235) Vincent, I.; Kruger, A.; Bessarabov, D. Hydrogen Production by water Electrolysis with an Ultrathin Anion-exchange membrane (AEM). *Int. J. Electrochem. Sci.* **2018**, *13*, 11347 – 11358.

(236) Pandiarajan, T.; Berchmans, L. J.; Ravichandran, S. Fabrication of spinel ferrite based alkaline anion exchange membrane water electrolysers for hydrogen production. *RSC Adv.* **2015**, *5*, 34100–34108.

(237) Thangavel, P.; Ha, M.; Kumaraguru, S.; Meena, A.; Singh, A. N.; Harzandi, A. M.; Kim, K. S. Graphene-nanoplatelets-supported NiFe-MOF: high-efficiency and ultra-stable oxygen electrodes for sustained alkaline anion exchange membrane water electrolysis. *Energy Environ. Sci.* **2020**, *13*, 3447– 3458.

(238) Carbone, A.; Zignani, S. C.; Gatto, L.; Trocino, S.; Aricò, A. S. Assessment of the FAA3-50 polymer electrolyte in combination with a $NiMn_2O_4$ anode catalyst for anion exchange membrane water electrolysis. *Int. J. Hydrog. Energ*. **2020**, *45*, 9285–9292.

(239) Park, J. E.; Kim, M.-J.; Lim, M. S.; Kang, S. Y.; Kim, J. K.; Oh, S.-H.; Her, M.; Cho, Y.-H.; Sung, Y.-E. Graphitic carbon nitride-carbon nanofiber as oxygen catalyst in anion-exchange membrane water electrolyzer and rechargeable metal–air cells. *Appl. Catal. B* **2018**, *237*, 140–148.

(240) Faid, A. Y.; Barnett, A. O.; Seland, F.; Sunde, S. Highly Active Nickel-Based Catalyst for Hydrogen Evolution in Anion Exchange Membrane Electrolysis. *Catalysts* **2018**, *8*, 614–616.

(241) Choi, W.-S.; Jang, M. J.; Park, Y. S.; Lee, K. H.; Lee, J. Y.; Seo, M.-H.; Choi, S.M. Three-Dimensional Honeycomb-Like $Cu_{0.81}Co_{2.19}O_4$ Nanosheet Arrays Supported by Ni Foam and Their High Efficiency as Oxygen Evolution Electrodes. *ACS Appl. Mater. Interfaces* **2018**, *10*, 38663–38668.

(242) Busacca, C.; Zignani, S. C.; Blasi, A. D.; Faro, M. L.; Antonucci, V.; Aricò, A. S. Electrospun $NiMn_2O_4$ and $NiCo_2O_4$ spinel oxides supported on carbon nanofibers as electrocatalysts for the oxygen evolution reaction in an anion exchange membrane-based electrolysis cell. *Int. J. Hydrog. Energ.* **2019**, *44*, 20987-20996.

(243) Park, J. E.; Kang, S.Y.; Oh, S.-H.; Kim, J. K.; Lim, M. S.; Ahn, C.-Y.; Cho, Y.-H.; Sung, Y.-E. High-performance anion-exchange membrane water electrolysis. *Electrochim. Acta* **2019**, *295*, 99–106.

(244) Seetharaman, S.; Balaji, R.; Ramya, K.; Dhathathreyan, K. S.; Velan, M. Graphene oxide modified non-noble metal electrode for alkaline anion exchange membrane water electrolyzers. *Int. J. Hydrog. Energ.* **2013**, *38*, 14934–14942.

(245) Joe, J. D.; J. Kumar, B. D. S.; Sivakumar, P. Production of hydrogen by anion exchange membrane using AWE. *Int. J. Sci .Technol. Res.* **2014**, 3, 38–42.

(246) Kaczur, J. J.; Yang, H.; Liu, Z.; Sajjad, S. D.; Masel, R. I. Carbon Dioxide and Water Electrolysis Using New Alkaline Stable Anion Membranes. *Front. Chem.* **2018**, *6*, 1–16.

(247) Liu, Z.; Sajjad, S. D.; Gao, Y.; Kaczur, J.; Masel, R. An Alkaline Water Electrolyzer with Sustainion™ Membranes: 1 A/cm² at 1.9V with Base Metal Catalysts. *ECS Trans.* **2017**, *77*, 71–73.

(248) Liu, Z.; Sajjad, S. D.; Gao, Y.; Yang, H.; Kaczur, J. J.; Masel, R. I. The effect of membrane on an alkaline water electrolyzer. *Int. J. Hydrog. Energ.* **2017**, 42, 29661-29665.

(249) Chen, P.; Hu, X. High-Efficiency Anion Exchange Membrane Water Electrolysis Employing Non-Noble Metal Catalysts. *Adv. Energy Mater.* **2020**, *10*, 2002285-2002291.

(250) Motealleh, B.; Liu, Z.; Masel, R. I.; Sculley, J. P.; Richard Ni, Z.; Meroueh, L. Next-generation anion exchange membrane water electrolyzers operating for commercially relevant lifetimes. *Int. J. Hydrog. Energ.* **2021**, *46*, 3379–3386.

(251) Park, Y. S.; Yang, J.; Lee, J.; Jang, M. J.; Jeong, J.; Choi, W. S.; Kim, Y.; Yin, Y.; Seo, M. H.; Chen, Z.; Choi, S. M. Superior performance of anion exchange membrane water electrolyzer: ensemble of producing oxygen vacancies and controlling mass transfer resistance. *Appl. Catal. B* **2020**, *278*, 119276–119287.

(252) Lee, J.; Jung, H.; Park, Y. S.; Woo, S.; Kwon, N.; Xing, Y.; Oh, S. H.; Choi, S. M.; Han, J. W.; Lim, B. Corrosion-engineered bimetallic oxide electrode as anode for high-efficiency anion exchange membrane water electrolyzer. *Chem. Eng. J.* **2021**, *420*, 127670–127677.

(253) Colli, A. N.; Girault, H. H.; Battistel, A. Non-Precious Electrodes for Practical Alkaline Water Electrolysis. *Mater.* **2019**, *12*, 1336–1352.

(254) Fortin, P.; Khoza, T.; Cao, X.; Martinsen, S. Y.; Barnett, A. O.; Holdcroft, S. High-performance alkaline water electrolysis using Aemion™ anion exchange membranes. *J. Power Sources* **2020**, *451*, 227814–227824.

(255) Parrondo, J.; George, M.; Capuano, C.; Ayers, K. E.; Ramani, V. Pyrochlore electrocatalysts for efficient alkaline water electrolysis. *J. Mater. Chem. A* **2015**, *3*, 10819–10828.

(256) Wu, X.; Scott, K. $Cu_xCo_{3-x}O_4$ ($0 \leq x < 1$) nanoparticles for oxygen evolution in high performance alkaline exchange membrane water electrolysers. *J. Mater. Chem.* **2011**, *21*, 12344–12351.

(257) Li, X.; Walsh, F. C.; Pletcher, D. Nickel based electrocatalysts for oxygen evolution in high current density, alkaline water electrolysers. *Phys. Chem. Chem. Phys.* **2011**, *13*, 1162–1167.

(258) Chi, J.; Yu, H.; Li, G.; Fu, L.; Jia, J.; Gao, X.; Yi, B.; Shao, Z. Nickel/Cobalt Oxide as a Highly Efficient OER Electrocatalyst in an Alkaline Polymer Electrolyte Water Electrolyzer. *RSC Adv.* **2016**, *6*, 90397–90400.

(259) Choe, Y. K.; Fujimoto, C.; Lee, K. S.; Dalton, L. T.; Ayers, K.; Henson, N. J.; Kim, Y. S. Alkaline Stability of Benzyl Trimethyl Ammonium Functionalized Polyaromatics: A Computational and Experimental Study. *Chem. Mater.* **2014**, *26*, 5675–5682.

(260) Razmjooei, F.; Morawietz, T.; Taghizadeh, E.; Hadjixenophontos, E.; Mues, L.; Gerle, M.; Wood, B.D.; Harms, C.; Gago, A.S.; Ansar, S.A.; et al. Increasing the performance of an anion- exchange membrane electrolyzer operating in pure water with a nickel-based microporous layer. *Joule* **2012**, *5*, 1776–1799.

(261) Soni, R.; Miyanishi, S.; Kuroki, H.; Yamaguchi, T. Pure Water Solid Alkaline Water Electrolyzer Using Fully Aromatic and High-Molecular-Weight Poly(fluorene-*alt*-tetrafluorophenylene)-trimethyl Ammonium Anion Exchange Membranes and Ionomers. *ACS Appl. Energy Mater.* **2021**, *4*, 1053−1058.

(262) Lindquist, G.A.; Oener, S.Z.; Krivina, R.; Motz, A.R.; Keane, A.; Capuano, C.; Ayers, K.E.; Boettcher, S.W.; Performance and Durability of Pure-Water-Fed Anion Exchange Membrane Electrolyzers Using Baseline Materials and Operation. *ACS Appl. Mater. Interfaces* **2021**, *13*, 51917−51924.

(263) Park, J. E.; Park, S.; Kim, M.-J.; Shin, H.; Kang, S. Y.; Cho, Y.-H.; Sung, Y.-E. Three-Dimensional Unified Electrode Design Using a NiFeOOH Catalyst for Superior Performance and Durable Anion-Exchange Membrane Water Electrolyzers. *ACS Cat.* **2022**, *12*, 135–145.

(264) Wang, J.; Zhao, Y.; Setzler, B. P.; Rojas-Carbonell, S.; Yehuda, C. B.; Amel, A.; Page, M.; Wang, L.; Hu, K.; Shi, L.; et al. Poly(aryl piperidinium) membranes and ionomers for hydroxide exchange membrane fuel cells. *Nat. Energy.* **2019**, *4*, 392−398.

(265) Epsztein, R.; Shaulsky, E.; Qin, M.; Elimelech, M. Activation behavior for ion permeation in ion-exchange membranes: Role of ion dehydration in selective transport. *J. Mem. Science* **2019**, *15*, 316−326.

(266) Zhegur-Khais, A.; Kubannek. F.; Krewer. U.; Dekel. D.R. Measuring the true hydroxide conductivity of anion exchange membranes. *J. Mem. Science* **2020**, *612*, 118461−118470.

(267) Mayerhöfer, B.; Ehelebe, K.; Speck, F.D.; Bierling, M.; Bender, J.; Kerres, J.A.; Mayrhofer, K.J.J.; Cherevko, S.; Peach, R.; Thiele, S. On the effect of anion exchange ionomer binders in bipolar electrode membrane interface water electrolysis. *J. Mater. Chem. A* **2021**, *9*, 14285−14295.

(268) Tsotridis, G.; Pilenga. A.; EU harmonised protocols for testing of low temperature water electrolysers. *JRC Technical report* **2021**, 1−161.

(269) Frensch, S. H.; Fouda-Onana, F.; Serre, G.; Thoby, D.; Araya, S. S.; Kær, S. K. Influence of the Operation Mode on PEM Water Electrolysis Degradation. *Int. J. Hydrogen Energ.* **2019**, *44*, 29889–29898.
